# Rhodium‐Catalyzed Intermolecular Arylative [2 + 2 + 1] Annulation–Oxidation to Produce Electron‐Deficient Azulene‐Embedded Polycyclic Aromatic Hydrocarbons

**DOI:** 10.1002/anie.202505622

**Published:** 2025-05-30

**Authors:** Yoshinobu Kamiya, Yu Sato, Tomohiro Oriki, Yuko Kishida, Haruki Sugiyama, Waner He, Kexiang Zhao, Tsuyoshi Michinobu, Hidehiro Uekusa, Ken Tanaka

**Affiliations:** ^1^ Department of Chemical Science and Engineering Institute of Science Tokyo Ookayama, Meguro‐ku Tokyo 152–8550 Japan; ^2^ Department of Chemistry Institute of Science Tokyo Ookayama, Meguro‐ku Tokyo 152–8550 Japan; ^3^ Neutron Industrial Application Promotion Center Comprehensive Research Organization for Science and Society 162‐1 Shirakata, Tokai‐mura, Naka‐gun, Ibaraki Naka 319–1106 Japan; ^4^ Department of Materials Science and Engineering Institute of Science Tokyo Ookayama, Meguro‐ku Tokyo 152–8550 Japan

**Keywords:** [2 + 2 + 1] annulation, Alkynes, Azulenes, Near‐infrared absorption, Rhodium, π–π stacking interactions

## Abstract

Azulene derivatives have attracted much attention for their application in organic electronic materials and devices because of their large dipole moment and small HOMO–LUMO energy gap. As these physical properties of azulene depend on its substitution and condensation patterns, developing methods to synthesize functionalized and π‐extended azulenes is desirable. However, synthesizing π‐extended azulenes requires harsh reaction conditions, making it hard to achieve both functionalization and π‐extension. Here, we report the synthesis of electron‐deficient azulene‐embedded polycyclic aromatic hydrocarbons (PAHs) with two alkoxycarbonyl groups by the rhodium‐catalyzed intermolecular arylative [2 + 2 + 1] annulation of teraryl diynes with dialkyl acetylenedicarboxylates followed by oxidation at room temperature. Interestingly, for the electron‐rich diyne, prolonged oxidation time after the arylative [2 + 2 + 1] annulation yields a helicene‐like bis(azulene‐embedded PAH) in good yield. Thus, obtained electron‐deficient fused azulenes have small HOMO–LUMO energy gaps (up to *E*_g_^elec^ = 1.52 and *E*_g_^theo^ = 2.06), resulting in long‐wavelength absorption extending into the near‐infrared region. Due to bulky electron‐withdrawing groups and π‐extension, the molecule becomes saddle‐shaped and highly polarized, and strong π–π stacking interactions are observed in both the solid and solution states.

## Graphical abstract

The synthesis of electron‐deficient azulene‐embedded polycyclic aromatic hydrocarbons has been achieved by the rhodium‐catalyzed intermolecular arylative [2 + 2 + 1] annulation of teraryl diynes with dialkyl acetylenedicarboxylates followed by oxidation at room temperature. For the electron‐rich diyne, prolonged oxidation time after the arylative [2 + 2 + 1] annulation yields a helicene‐like bis(azulene‐embedded PAH) with a small HOMO–LUMO energy gap and long‐wavelength absorption extending into the near‐infrared region.

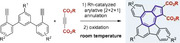

## Introduction

Azulene is a non‐alternating hydrocarbon with a large dipole moment^[^ ^1^ ^]^ and a small HOMO–LUMO energy gap.^[^ ^2^ ^]^ These physical properties of azulene depend on its substitution and condensation patterns, and thus developing synthetic methods for functionalized and π‐extended azulenes is valuable for their application in organic electronic materials and devices.^[^ ^3^, ^4^, ^5^, ^6^, ^7^, ^8^, ^9^, ^10^, ^11^, ^12^, ^13^, ^14^ ^]^ Due to the lack of versatile methods for synthesizing azulene cores from pre‐functionalized precursors, the general strategy for substituted azulene synthesis was functionalization after constructing the azulene backbone. Incorporating azulene units into polycyclic aromatic hydrocarbons (PAHs) was also difficult due to challenges in forming heptagons and their incorporation into polyarenes.^[^ ^15^ ^]^

**Figure 1:**
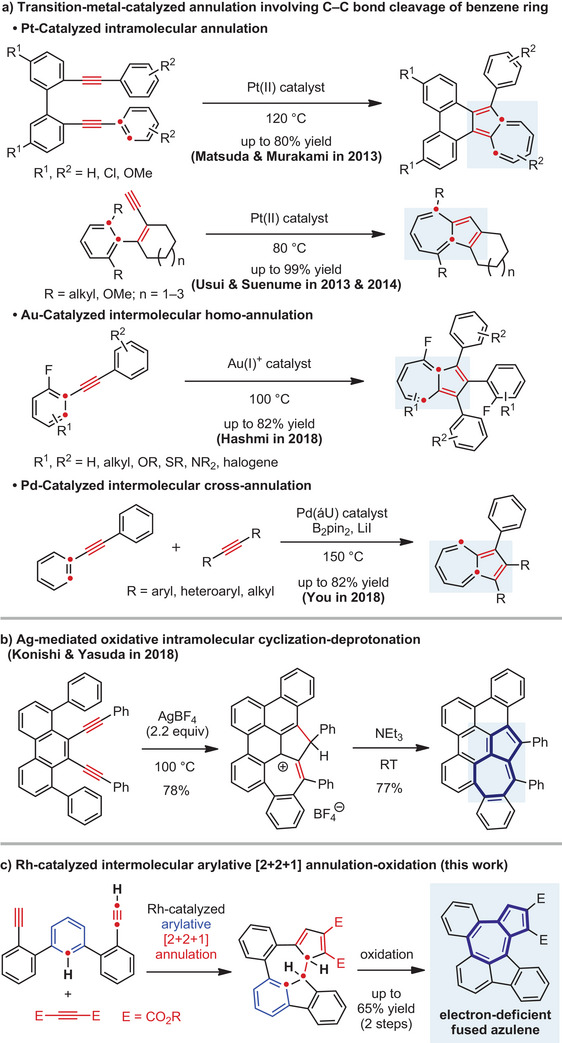
Syntheses of azulene derivatives via transition‐metal‐mediated annulations of alkynes.

Following the pioneering works by Ziegler–Hafner^[^ ^16^ ^]^ and Nozoe,^[^ ^17^ ^]^ many efforts have been made to develop concise methods for synthesizing functionalized and π‐extended azulene derivatives.^[^ ^18^, ^19^, ^20^ ^]^ Several effective strategies have been developed that utilize the cyclization reactions of alkynes, enabling the straightforward design of stable pre‐functionalized precursors. Initially, the homodimerization of alkynes was explored, but this approach encountered challenges, including low yields and the requirement for stoichiometric amounts of transition metals.^[^ ^21^, ^22^, ^23^ ^]^ Subsequently, since 2013, synthetic methods for substituted azulenes involving carbon–carbon bond cleavage of the benzene ring by transition metal‐catalyzed skeletal rearrangement have been developed. In 2013 and 2014, Matsuda–Murakami^[^ ^24^ ^]^ and Usui–Suenume^[^ ^25^, ^26^ ^]^ reported platinum‐catalyzed intramolecular cyclizations of diynes and enynes, respectively (Figure 1a, top); in 2018, Hashmi reported cationic gold‐catalyzed homo‐cyclizations of diarylalkynes (Figure 1a, middle)^[^ ^27^, ^28^, ^29^ ^]^; in 2018, You reported the palladium‐catalyzed cross‐[3 + 2] cyclization and simultaneous aromatic ring expansion with B_2_pin_2_ (pin = pinacol borane) and LiI (Figure 1a, bottom).^[^ ^30^ ^]^ This reaction applies to both homo‐ and cross‐cyclization and exhibits a broad substrate range. However, the above methods involve cleavage of the carbon–carbon bond of the benzene ring and are therefore not suitable for π‐expansion of the seven‐membered ring moiety of the azulene skeleton.

For the π‐extension of the azulene, intramolecular cyclization of substrates with pre‐incorporated π‐systems has been reported. Following the on‐surface dehydrocyclization synthesis of azulene‐embedded PAHs reported by Peña–Pascual^[^ ^31^ ^]^ and Feng and coworkers,^[^ ^32^, ^33^ ^]^ Mastalerz,^[^ ^34^, ^35^ ^]^ Zhang,^[^ ^36^ ^]^ Chi,^[^ ^37^ ^]^ Konishi–Yasuda,^[^ ^38^ ^]^ and Takasu^[^ ^39^ ^]^ reported the synthesis of unfunctionalized azulene‐embedded PAHs in solution via the intramolecular cyclization, such as the Scholl and Friedel–Crafts reactions. Würthner reported the synthesis via the palladium‐catalyzed intermolecular double [5 + 2] annulation from a 3,9‐diboraperylene precursor.^[^ ^40^ ^]^ As for the synthesis by cyclization of alkynes, Konishi–Yasuda reported the synthesis via silver‐mediated oxidative intramolecular diyne cyclization followed by deprotonation (Figure 1b).^[^ ^41^ ^]^ However, these methods have not been applied for synthesizing functionalized azulene‐embedded PAHs, presumably due to harsh reaction conditions using strong Brønsted or Lewis acids and/or high temperatures.

Here, we report the synthesis of electron‐deficient azulene‐embedded PAHs with two alkoxycarbonyl groups by the rhodium‐catalyzed intermolecular arylative [2 + 2 + 1] annulation^[^ ^42^, ^43^, ^44^ ^]^ followed by oxidation at room temperature (Figure 1c). We also report the structural (saddle‐shaped structures and strong π–π stacking interactions in both the solid and solution states) and physical properties (large dipole moments, small HOMO–LUMO energy gaps, and near‐infrared absorption) of azulene‐embedded PAHs due to bulky electron‐withdrawing groups and π‐extension.

## Results and Discussion

Recently, we reported the synthesis of highly strained, substituted *o*,*m*,*o*,*p*‐tetraphenylene **3aa** by the cationic rhodium(I)/H_8_‐binap complex‐catalyzed intermolecular [2 + 2 + 2] cycloaddition of teraryl diyne **1a** with dimethyl acetylenedicarboxylate (**2a**).^[^ ^45^ ^]^ In this reaction, we found that five to seven ring fused by‐product **5aa** is generated as a mixture of double bond regioisomers via the arylative [2 + 2 + 1] annulation, along with *o*,*m*,*o*,*m*‐tetraphenylene by‐product **4aa**. Air oxidation of **5aa** gradually proceeded upon isolation by silica gel chromatography to generate electron‐deficient azulene‐embedded PAH **6aa**. Thus, the crude reaction mixture was treated with 2,3‐dichloro‐5,6‐dicyano‐1,4‐benzoquinone (DDQ) at room temperature to yield **6aa** in 14% NMR yield (Table 1, entry 1). Screening of axially chiral biarylbisphosphine ligands (entries 1–4) showed that the yield of **6aa** tends to improve with decreasing the dihedral angle of the ligand^[^ ^46^ ^]^ (dihedral angle: H_8_‐binap > binap > MeO‐biphep, yield of **6aa**: H_8_‐binap < binap < MeO‐biphep). However, further decreasing the dihedral angle of the ligand (segphos and biphep) lowered the yield of **6aa** (entry 5). Testing electron‐deficient MeO‐biphep ligands (difluorphos and P‐phos) (entries 6 and 7) revealed that the use of P‐phos affords **6aa** in the highest yield of 60% and **3aa** in the lowest yield of 1% (entry 7). Sterically demanding segphos derivatives decreased the yield of **6aa** (entries 8 and 9). P‐phos was the best ligand because it outperformed MeO‐biphep on other less reactive substrates.

**Table 1:** Screening of reaction conditions.^a)^

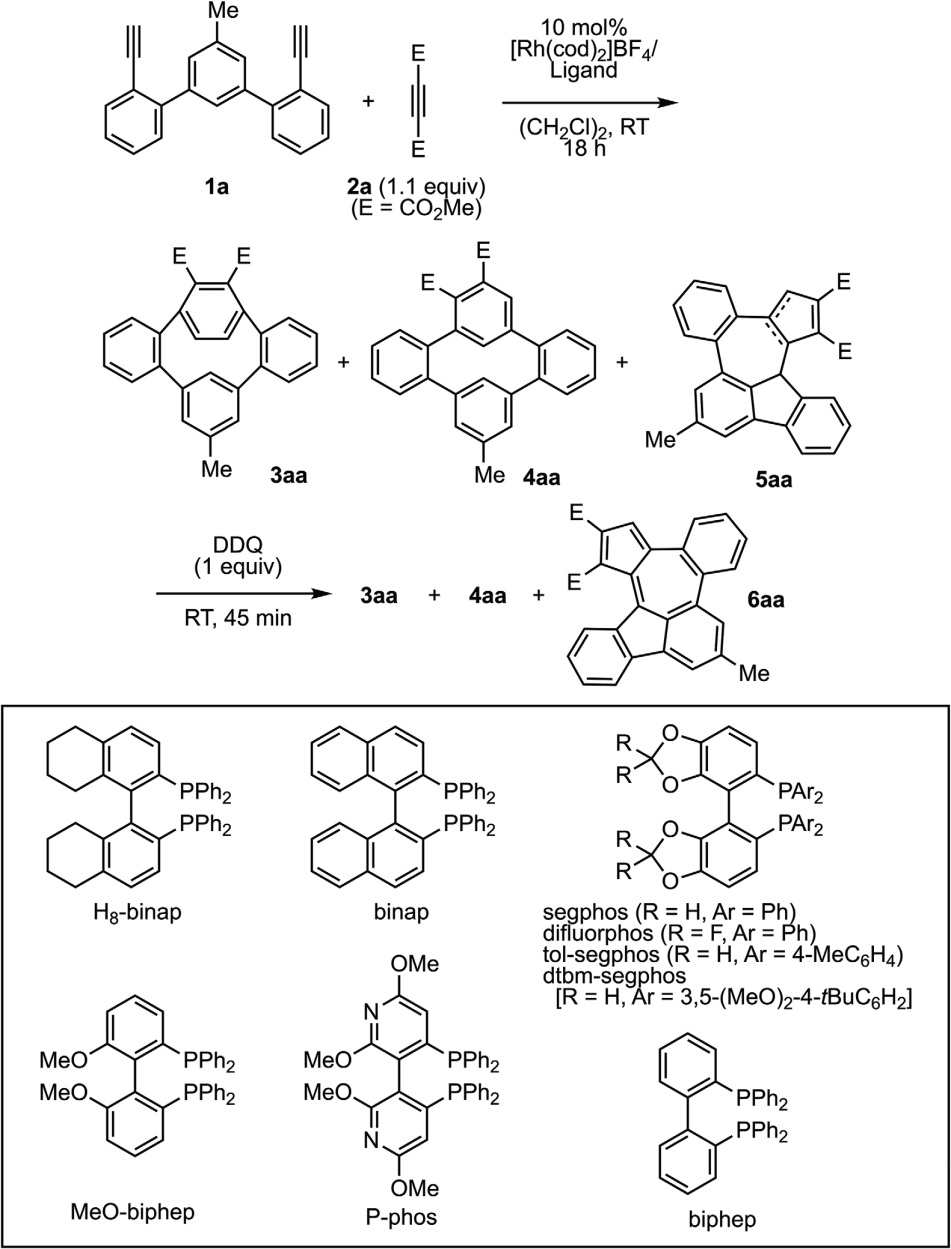				
Entry	Ligand	**3aa** / % Yield^b)^	**4aa** / % Yield^b)^	**6aa** / % Yield^b)^
1	H_8_‐binap	24	5	14
2	binap	22	7	24
3	MeO‐biphep	5	9	60 (48^c)^)
4	segphos	2	8	55 (47^c)^)
5	biphep	6	12	51
6	difluorphos	3	12	48
7	P‐phos	1	12	60 (48^c)^)
8	tol‐segphos	6	6	40
9	dtbm‐segphos	11	12	27

^a)^ [Rh(cod)_2_]BF_4_ (0.010 mmol), ligand (0.010 mmol, difluorphos: *S*‐isomer and others: *R*‐isomers), **1a** (0.10 mmol), **2a** (0.11 mmol), and (CH_2_Cl)_2_ (10 mL) were used.

^b)^ Determined by ^1^H NMR using tetrachloroethane as an internal standard.

^c)^ Isolated yield. cod = 1,5‐cyclooctadiene.

**Figure 2:**
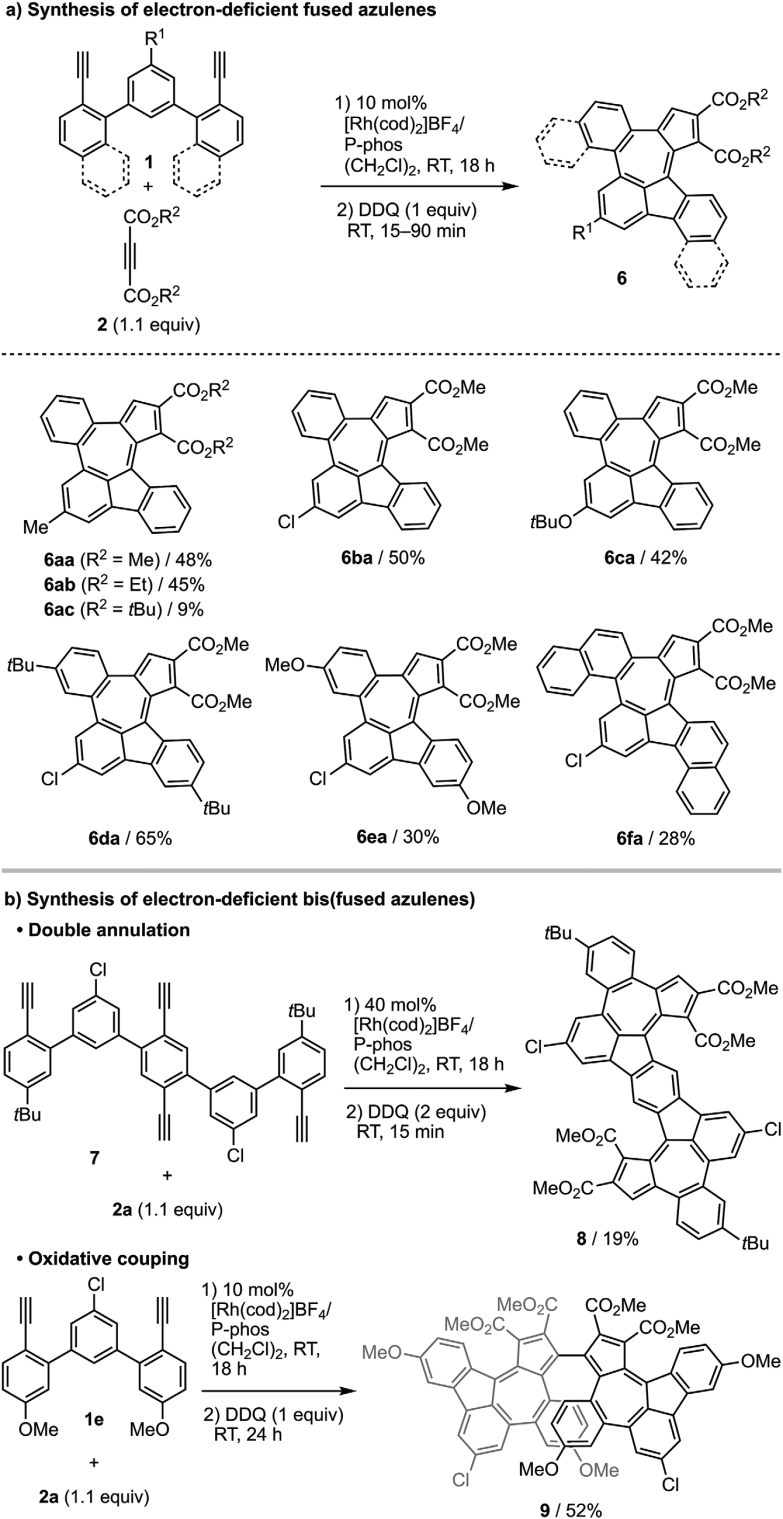
Scope of azulene‐embedded PAH synthesis. Yields are of the isolated products. [Rh(cod)_2_]BF_4_ (0.0050—0.010 mmol), P‐phos (0.0050—0.010 mmol), **1** (0.050—0.10 mmol), **2** (0.055—0.11 mmol), and (CH_2_Cl)_2_ (5—10 mL) were used.

With the optimized reaction conditions, we investigated the scope of arylative [2 + 2 + 1] annulation–oxidation to produce azulene‐embedded PAHs (Figure 2a). Not only dimethyl (**2a**) but also diethyl (**2b**) acetylenedicarboxylates could be used to give the corresponding azulene‐embedded PAHs **6aa** and **6ab** in 48% and 45% yield, respectively. However, the use of sterically demanding di‐*tert*‐butyl acetylenedicarboxylate (**2c**) much decreased the product yield (**6ac**, 9%) due to reducing the reaction rate. Other than acetylenedicarboxylates, methyl propiolate, methyl but‐2‐ynoate, methyl 3‐phenylpropiolate, 1‐ethynyl‐4‐methylbenzene, and ethynyltrimethylsilane were also tested, but the corresponding azulene‐embedded PAHs were not generated. Next, we examined the substituents on teraryl diyne **1**. Not only methyl‐ but also chloro‐ and *tert*‐butoxy‐substitution on the central benzene ring was amenable to yield **6ba** and **6ca** in good yields. Substitution on the terminal benzene rings with *tert*‐butyl groups was also amenable to yield **6da** in good yield, but methoxy‐substituted diyne **1e** and naphthalene‐derived diyne **1f** yielded the corresponding azulene‐embedded PAHs **6ea** and **6fa** in low yields. The double arylative [2 + 2 + 1] annulations of tetrayne **7** with **2a** yielded the corresponding fused double azulene‐embedded PAH **8**, although the yield was low at 19% (Figure 2b, top). Interestingly, for the electron‐rich diyne **1e**, prolonged oxidation time after the arylative [2 + 2 + 1] annulation with **2a** led to the oxidative homo‐coupling to yield helicene‐like bis(azulene‐embedded PAH) **9** in good yield (Figure 2b, bottom).^[^ ^47^, ^48^ ^]^ In these reactions, in addition to producing cyclophanes (**3** and **4**), insoluble substances, which may be oligomers from intermolecular reactions, were also formed.

**Figure 3:**
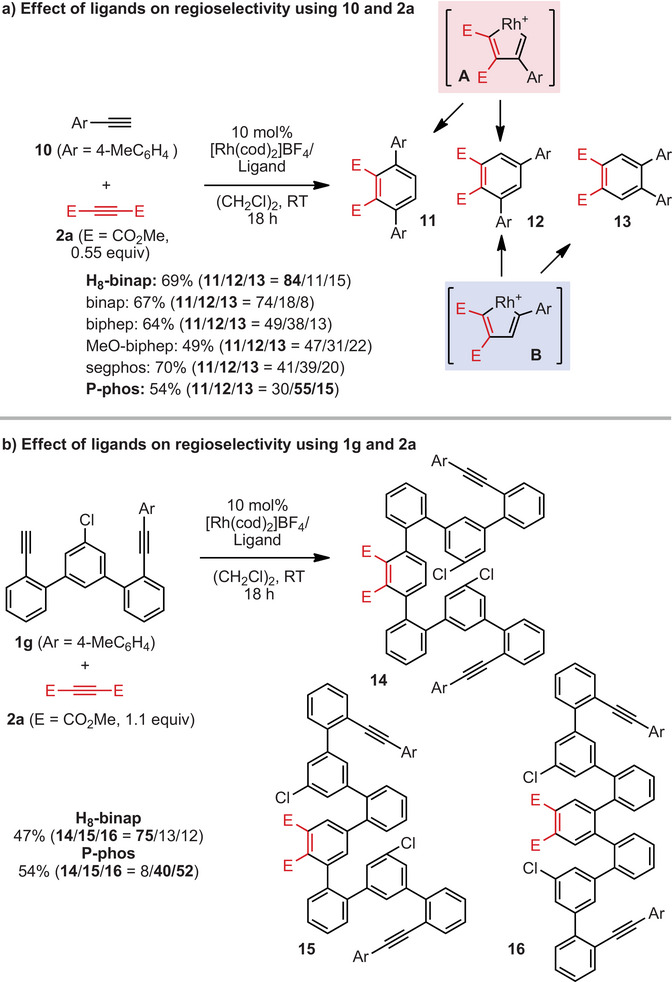
Experimental mechanistic studies. Yields were determined by ^1^H NMR using tetrachloroethane as an internal standard. a) Yields are based on **10**. b) Yields are based on **1g**.

To clarify the mechanism of this reaction, we investigated the ligand effect on the regioselectivity of the intermolecular [2 + 2 + 2] cycloaddition^[^ ^49^, ^50^, ^51^, ^52^ ^]^ of arylacetylene **10** with **2a** (Figure 3a).^[^ ^53^, ^54^ ^]^ The regioselectivity of the resulting rhodacycle and the subsequent coordination direction of the alkyne determine the ratio of the products **3aa**, **4aa**, and **5aa**. As anticipated, the yield of **11** correlated with the yield of **3aa**: H_8_‐binap, which has the highest selectivity for **11**, gave the highest yield of **3aa**, and P‐phos, which has the lowest selectivity for **11**, gave the lowest yield of **3aa**. In contrast, the sum of the yields of **12** and **13** correlated with the sum of the yields of **4aa** and **5aa**. P‐phos, which has the highest combined yield of **12** and **13**, gave the highest combined yield of **4aa** and **5aa**, and H_8_‐binap, which has the lowest combined yield of **12** and **13**, gave the lowest combined yield of **4aa** and **5aa**. We then, investigated the reaction of **2a** and **1** **g**, in which one end of the diyne terminus was substituted with an aryl group, using H_8_‐binap and P‐phos. Using H_8_‐binap gave **14** in high yield, whereas using P‐phos gave **15** and **16** in high yield (Figure 3b).

**Figure 4:**
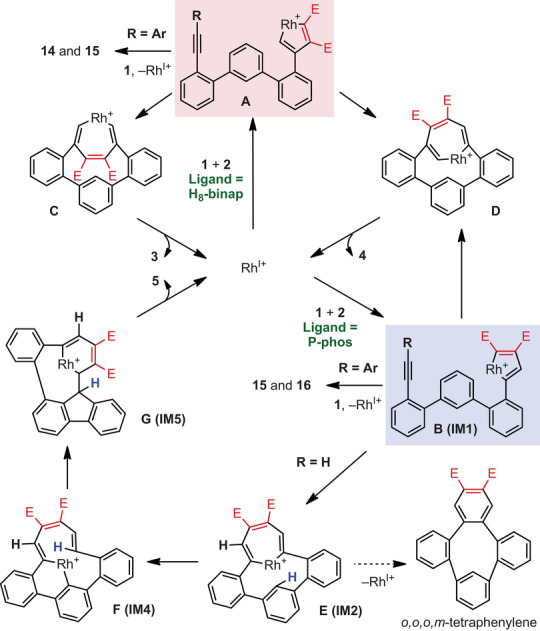
Proposed reaction mechanisms.

From the above results, a plausible mechanism of the [2 + 2 + 1] cycloaddition reaction is as follows (Figure 4). Diyne **1** reacts with **2** and rhodium, producing rhodacycles **A** and **B**. Insertion of an alkyne into **A** produces rhodacycle **C** or **D**, and subsequent reductive elimination affords **3** or **4**, respectively.^[^ ^41^ ^]^ Insertion of an alkyne into rhodacycle **B** also affords rhodacycle **D**, producing **4** by reductive elimination.^[^ ^41^ ^]^ On the other hand, the insertion of an alkyne in the opposite direction produces rhodacycle **E**, which is less distorted than **D**. In this rhodacycle **E**, a metathesis reaction between the Rh^III^─C and C─H bonds proceeds to produce rhodacycle **F**,^[^ ^55^, ^56^, ^57^, ^58^, ^59^, ^60^, ^61^, ^62^ ^]^ but no *o*,*o*,*o*,*m*‐tetraphenylene formation by reductive elimination was observed. Carborhodation produces another rhodacycle **G**, which undergoes reductive elimination to produce **5**. Indeed, when H_8_‐binap was used to react **2a** with **1g** (R = Ar), in which one termini of the diyne is substituted with an aryl group, **14** was generated in high yield, while using P‐phos afforded **15** and **16** in high yields (Figure 3b). These results confirm that using H_8_‐binap produces intermediate **A** predominantly, and using P‐phos produces intermediate **B** predominantly.

**Figure 5:**
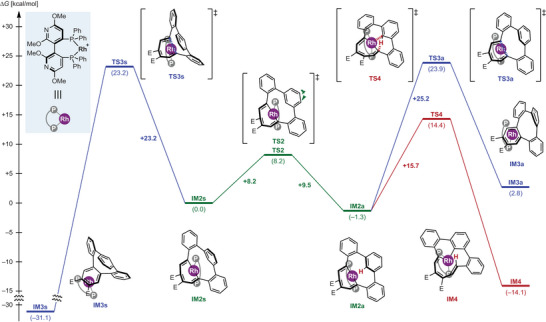
Theoretical mechanistic study: Energy diagrams of proposed reaction pathways. Energy changes are shown in kcal mol^−1^, and represent the relative free energies calculated at the M06/6–311 + G(d,p)&SDD(Rh)+PCM[(CH_2_Cl)_2_]//M06/6–31G(d)&LANL2DZ(Rh).

To verify the validity of this mechanism, we conducted a DFT calculation of the transition state of the Rh^III^─C and C─H metathesis reaction from intermediate **E** (**IM2**) to intermediate **F** (**IM4**) (Figure 5, complete energy diagrams: Figure S33). Intermediate **IM2** has two diastereomers: **IM2a** (anti) is lower in energy than **IM2s** (syn), and they are interconvertible via **TS2** at room temperature with activation energies of 9.5 (anti) and 8.2 (syn) kcal mol^−1^. We confirmed that the metathesis reactions from **IM2a** (anti) to generate **IM4** is an exothermic process, and activation energy is 15.7 kcal mol^−1^ via **TS4** (Figure S34), indicating that the C─H bond activation can proceed at room temperature. The present system is interconvertible, and a comparison of the transition state energies reveals that the transition state energies of **TS3s** and **TS3a** for reductive elimination, yielding *o*,*o*,*o*,*m*‐tetraphenylene (**IM3s** and **IM3a**), are 23.2 and 23.9 kcal mol^−1^, respectively, which are markedly higher and less favorable than the transition state energy for the C─H bond activation (**TS4**: 14.4 kcal mol^−1^).

We also calculated the oxidative cyclization pathways using dimethyl acetylenedicarboxylate (**2a**) and a simplified diyne model (phenylacetylene), revealing that when using P‐phos, the transition state giving rhodacycle **B** is the most stable, while when using H_8_‐BINAP, the transition state giving rhodacycle **A** is the most stable (Figures S35 and S36 and Tables S15 and S16). Furthermore, we also performed a theoretical analysis of the mechanism of the oxidative dimerization reaction of **6ea** to **9**, revealing that this reaction proceeds via one‐electron oxidation due to the high energy level of the HOMO of **6ea** (Table S17 and Figures S37 and S38).^[^ ^63^ ^]^

**Figure 6:**
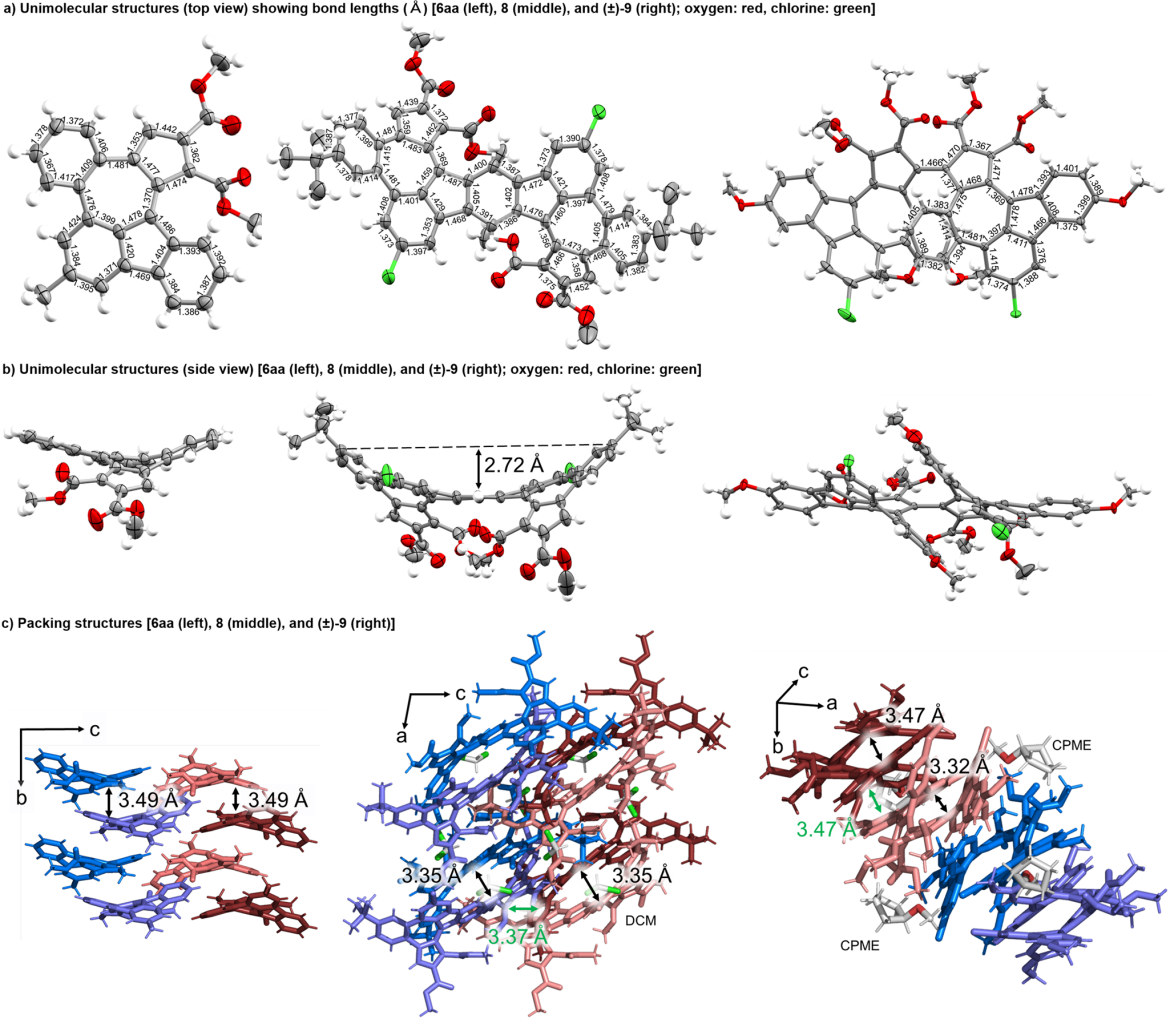
X‐ray crystal structures of **6aa**, **8**, and (±)‐**9**. The shortest C–C distances between two opposing benzene rings are shown. In Figure 6c, the *P*‐isomer and *M*‐isomer are colored in blue and red, respectively. DCM = CH_2_Cl_2_; CPME = cyclopentyl methyl ether.

We successfully obtained single crystals by recrystallization from CH_2_Cl_2_ (DCM)/*n*‐hexane solutions of **6aa** and **8** and a cyclopentyl methyl ether (CPME)/*n*‐hexane solution of (±)‐**9**.^[^ ^64^ ^]^ Thus, we performed their single‐crystal X‐ray diffraction analyses and clarified their structures in the solid state (Figure 6).^[^ ^41^ ^]^ Compound **6aa** (Figure 6, left) has a chiral saddle‐shaped structure. The crystal contains *M*‐ and *P*‐isomers in a 1:1 ratio, and the columnar stacking structure consists of alternating columns of *P*‐ and *M*‐isomers. The distance between the molecules is 3.49 Å, indicating the strong π–π stacking interactions between the identical enantiomers. Compound **8** (Figure 6, middle) also has a chiral saddle‐shaped structure. The crystal contains *M*‐ and *P*‐isomers in a 1:1 ratio, and two molecules of the identical enantiomer form one unit. Units of the opposite enantiomer are stacked alternately. The distance between the molecules is 3.35 Å (black, interactions of identical enantiomers) and 3.37 Å (green, interactions of the opposite enantiomers), indicating the strong π–π stacking interactions. Interestingly, compound **9** (Figure 6, right) exhibits a helically chiral saddle‐shaped structure because of intramolecular π–π stacking between the two terminal benzene rings (black, 3.32 and 3.47 Å). The crystal contains *M*‐ and *P*‐isomers in a 1:1 ratio, and the columnar stacking structure consists of alternating columns of *P*‐ and *M*‐isomers. The distance between the molecules is 3.47 Å (green), indicating the strong π–π stacking interactions between the identical enantiomers. The azulene unit of **6aa**, unlike unsubstituted azulene,^[^ ^65^ ^]^ shows a pronounced bond length alternation, varying between 1.36 and 1.48 Å (Figure 6a, left). The bond lengths of the benzene units are somewhat varied, ranging from 1.37 to 1.42 Å, due to condensation with the azulene unit (Figure 6a, left). This bond length trend is similar in double azulene‐embedded PAH **8** and bis(azulene‐embedded PAH) **9** (Figure 6a, middle and right). Interestingly, in **9**, the bond length of the C─C single bond between the two azulene units is shorter (1.47 Å) than the ordinal C─C single bond (1.54 Å), forming a helicene‐like structure (Figure 6a, right).

The observed π–π stacking interactions of **6aa** in the solid state prompted our investigation into the self‐association process of **6aa** through the π–π stacking interaction in the solution state.^[^ ^66^, ^67^ ^]^ We examined the concentration dependence of the ^1^H NMR spectra of **6aa** in CDCl_3_ at room temperature. As expected, marked upfield shifts of the aromatic protons were observed as the concentration increased (2.0→23.8 mM, Figures S14 and S15). The self‐association observed in this solution state is likely due to the saddle‐shaped and polarized structure of the azulene unit.^[^ ^39^ ^]^ The π‐surface of the negatively curved saddle molecules is solvated, and thus π–π stacking is entropically favored.^[^ ^68^, ^69^ ^]^ The polarized structure also favors stacking due to electrostatic interactions, and these upfield shifts resulted from face‐to‐face aggregations.^[^ ^39^ ^]^

**Figure 7:**
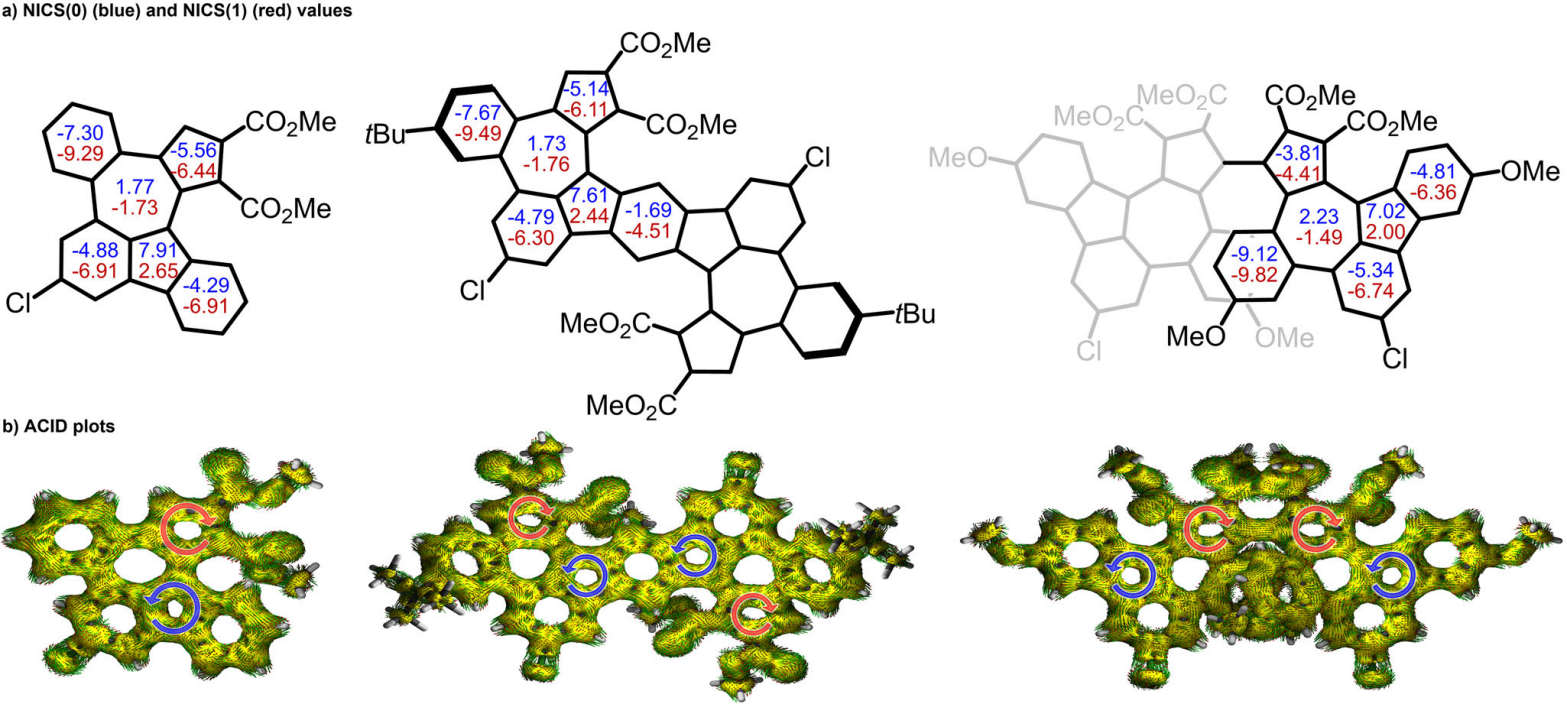
Aromaticity and global conjugation of **6ba** (left), **8** (middle), and **9** (right). Calculated at a) B3LYP/6–311 + G(2d,p)//B3LYP/6–31G(d) and b) B3LYP/6–31 + G(d,p)//B3LYP/6–31G(d) level of theory.

We evaluated the aromaticity of **6ba** by nucleus‐independent chemical shifts (NICS)^[^ ^70^, ^71^, ^72^ ^]^ calculations (Figure 7a). The five‐membered ring site of the fluorene backbone is strongly antiaromatic [NICS(0) = +7.91 ppm and NICS(1) = +2.65 ppm], and the seven‐membered ring site of the azulene backbone is less aromatic [NICS(0) = +1.77 ppm and NICS(1) = −1.73 ppm]. In contrast, the five‐membered ring site of the azulene backbone and the surrounding six‐membered rings show high negative values, consistent with the bond length analysis shown in Figure 6a. To further support the local aromaticity of **6ba** with embedded azulene, anisotropy of the induced current density (ACID)^[^ ^73^, ^74^ ^]^ analysis was performed (Figure 7b). In the ACID plot of **6ba**, a counterclockwise continuous paratropic ring current appears around the five‐membered ring site of the fluorene backbone, and a clockwise continuous diatropic ring current appears around the five‐membered ring site of the azulene backbone. These observations are consistent with the NICS calculations. Similar NICS values and ACID plots to **6ba** are observed for **8** and **9**.

**Figure 8:**
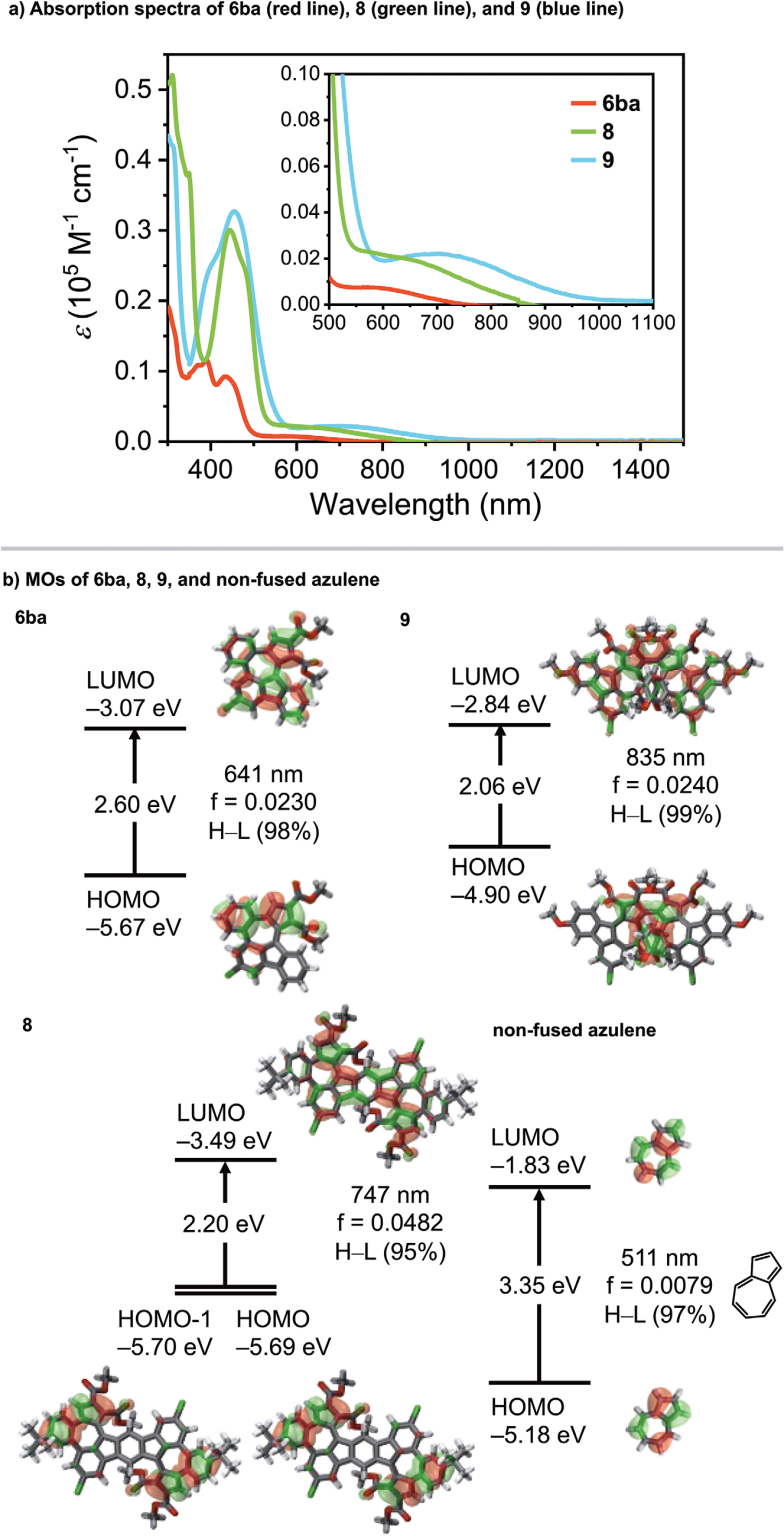
Absorption spectra of **6ba**, **8**, and **9** and MOs of **6ba**, **8**, **9**, and non‐fused azulene based on DFT calculations. Calculated at B3LYP/6–31G(d) level of theory.

Absorption spectra of **6ba**, **8**, and **9** in dichloromethane solution are shown in Figure 8a. For **6ba**, absorption wavelengths were overall markedly red‐shifted compared to unfused azulene.^[^ ^75^ ^]^ For **8**, the longest absorption maximum was at 648 nm, which is red‐shifted compared to **6ba** (573 nm). The further redshifted longest absorption maximum was observed at 698 nm for **9**, with the absorption tail reaching the near‐infrared region. The concentration (1 × 10^−5^ to 1 × 10^−3^ M) and solvent dependence of the absorption spectra were also investigated, but almost no changes were observed (Figures S43 and S44 and Table S18). Notably, these compounds did not exhibit any fluorescence.

We determined the frontier molecular orbitals (MOs) of **6ba**, **8**, and **9** by DFT calculations. As shown in Figure 8b, the HOMO is localized around the five‐membered ring site of the azulene backbone, whereas the LUMO is localized around the seven‐membered ring site. In addition, **6ba**, **8**, and **9** all have markedly lower LUMOs than unsubstituted azulene, and the HOMOs are also lower except for **9**. The HOMO–LUMO energy gaps of **6ba**, **8**, and **9** (2.06–2.60 eV) are all markedly smaller than that of unsubstituted azulene (3.35 eV) due to the increased conjugation. In particular, the HOMO–LUMO energy gaps of **8** and **9** are 2.20 and 2.06 eV, respectively, likely due to the donor–acceptor structure of the diester‐substituted azulene‐embedded PAH skeleton. These small HOMO–LUMO energy gaps lead to the absorption of **8** and **9** extending into the near‐infrared region. Time‐dependent (TD)‐DFT calculations assigned the HOMO–LUMO transitions of **6ba**, **8**, and **9** to 641 nm (*f* = 0.0230), 747 nm (*f* = 0.0481), and 835 nm (*f* = 0.0240), respectively. We investigated whether **8** has a contribution from the *s*‐indacene structure. The LUMO and LUMO + 1 have molecular orbitals with shapes characteristic of *s*‐indacene,^[^ ^76^ ^]^ while the HOMO–3 and HOMO–2 have molecular orbitals derived from terphenylene and HOMO–1 and HOMO do not have orbitals in the central benzene ring (Figure S22). In addition, there was no bond alternation in the central benzene ring (Figure 6a, middle), and the NICS value (Figure 7a, middle) confirmed aromaticity.

**Figure 9:**
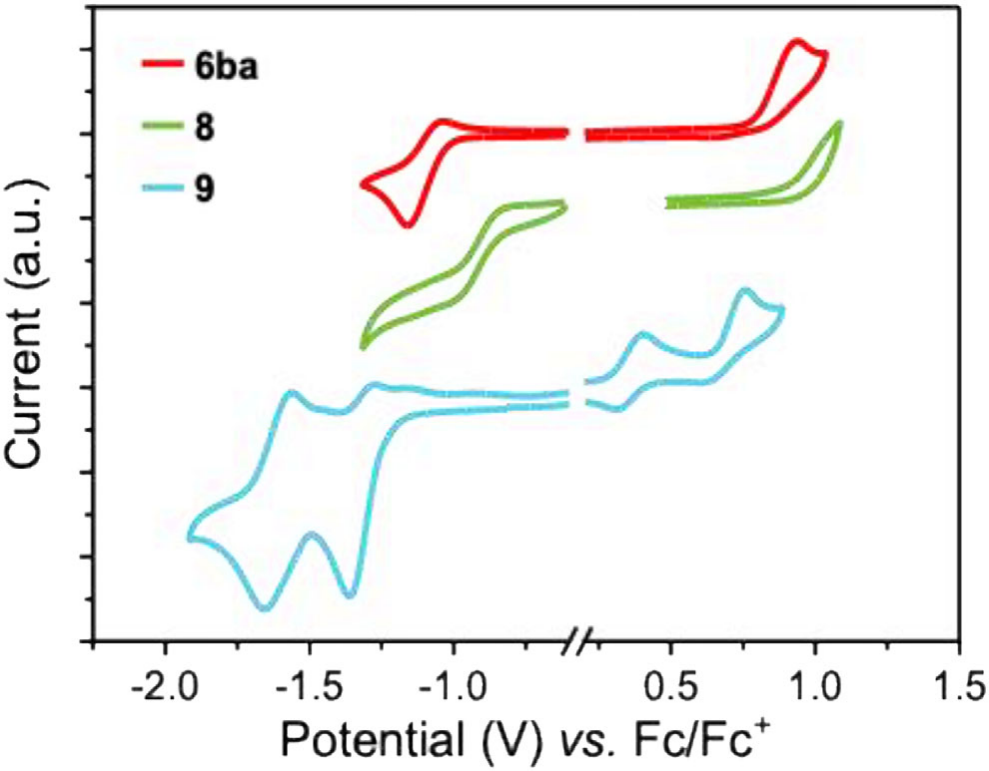
CV curves of **6ba** (red line), **8** (green line), and **9** (blue line).

Next, we investigated the electrochemical behaviors of **6ba**, **8**, and **9** by cyclic voltammetry (CV) in degassed CH_2_Cl_2_ with 0.1 M *n*‐Bu_4_NPF_6_. As shown in Figure 9 and Table 2, compound **6ba** exhibited an irreversible oxidation wave with the onset oxidation potential *E*_ox,onset_ = 0.79 V (versus Fc^+^/Fc) and a quasi‐reversible reduction wave with the onset reduction potential *E*_red,onset_ = −1.03 V. Compound **8** displayed irreversible oxidation and reduction waves with the *E*_ox,onset_ = 0.90 V and *E*_red,onset_ = −0.84 V, respectively. Compound **9** also displayed an irreversible redox wave. Its redox activity was higher than those of **6ba** and **8**, showing two oxidation waves with the *E*_ox,onset_ = 0.29 V and two reduction waves with the *E*_red,onset_ = −1.23 V. Accordingly, HOMO–LUMO levels are estimated to be −5.59/−3.77 eV, −5.70/−3.96 eV, and −5.09/−3.57 eV for **6ba**, **8**, and **9**, respectively, based on the onset potentials of the first oxidation–reduction waves. Electrochemical HOMO–LUMO energy gaps (*E* ^EC^_g_) are calculated to be 1.82, 1.74, and 1.52 eV for **6ba**, **8**, and **9**, respectively, which agreed well with the order of the energy gaps estimated by DFT calculations.

**Table 2:** HOMO and LUMO energy levels of **6ba**, **8**, and **9** based on CV.

Compound	HOMO (eV)	LUMO (eV)	Electrochemical energy gap *E* ^EC^_g_ (eV)
**6ba**	−5.59	−3.77	1.82
**8**	−5.70	−3.96	1.74
**9**	−5.09	−3.57	1.52

## Conclusion

We have achieved the synthesis of electron‐deficient azulene‐embedded PAHs up to 65% yield by the rhodium‐catalyzed intermolecular [2 + 2 + 1] annulation of teraryldiynes with dialkyl acetylenedicarboxylates followed by oxidation at room temperature. The reaction selectivity was controlled by adjusting the dihedral angle of the bisphosphine ligand, enabling the selective synthesis of π‐extended azulenes. Using an electron‐rich methoxy‐substituted diyne and prolonged oxidation time after the arylative [2 + 2 + 1] annulation yielded a helicene‐like bis(azulene‐embedded PAH) in good yield. Small HOMO–LUMO energy gaps (up to *E*_g_^elec^ = 1.52 and *E*_g_^theo^ = 2.06) of the thus obtained electron‐deficient azulene‐embedded PAHs led to long‐wavelength absorption extending into the near‐infrared region. Single‐crystal X‐ray crystallography revealed saddle‐shaped structures and intermolecular π–π stacking (3.35–3.49 Å), and ^1^H NMR spectra revealed concentration‐dependent peak shifts, confirming self‐association in the solid and solution states. We expect that the synthesized electron‐deficient azulene‐embedded PAHs through the current [2 + 2 + 1] annulation–oxidation strategy will be applicable not only in electronic materials but also as ligands and catalysts in organic synthesis.

## Conflict of Interests

The authors declare no conflict of interest.

## Supporting information



Supporting Information

Supporting Information

## Data Availability

The data that support the findings of this study are available in the supplementary material of this article.
