# Supplementary material for: Rhodium‐Catalyzed Intermolecular Arylative [2 + 2 + 1] Annulation–Oxidation to Produce Electron‐Deficient Azulene‐Embedded Polycyclic Aromatic Hydrocarbons
Source: Angew Chem Int Ed Engl. 2025 May 30;64(29):e202505622. doi: 10.1002/anie.202505622 (PMC12258683; doi:10.1002/anie.202505622)
Supplement: Supplementary file 1 — Supporting Information [file ANIE-64-e202505622-s002.pdf]

Table of Contents

1. General

1.1. General Experimental Information **S2**

1.2. General Analytical Information **S2**

2. Synthetic Experiments (Figure 2)

2.1. Synthesis of Diynes and Tetrayne **S3–S9**

2.2. Synthesis of Electron-Deficient Fused Azulenes **S10–S15**

3. Experimental Mechanistic Studies (Figure 3)

3.1. Effect of Ligands on Regioselectivity Using 10 and 2a (Figure 3a) **S16**

3.2. Effect of Ligands on Regioselectivity Using 1g and 2a (Figure 3b) **S16–S17**

4. X-Ray Crystallographic Analyses (Figure 6) **S18–S28**

5. Self-Association of 6aa in Solution-State **S29**

6. DFT and TD-DFT Calculations (Figures 5, 7, and 8b)

6.1. General **S30**

6.2. Structural Analyses (Figures 7 and 8b) **S30–S52**

6.3. Theoretical Mechanistic Studies (Figure 5) **S53–S75**

6.4. Theoretical Mechanistic Studies of Oxidative Cyclization Step **S76–S104**

6.5. Mechanistic Considerations of Oxidative Dimerization **S105–S112**

7. Photophysical Properties (Figure 8a)

7.1. Absorption Spectra of 6ba, 6fa, 7, and 9 (Figure 8a) **S113–S114**

7.2. Absorption Spectra of 6aa **S115**

8. Electronic Properties (Figure 9)

8.1. Cyclic Voltammetry (CV) Curves (Figure 9) **S116**

8.2. HOMO and LUMO Energy Levels Based on CV (Table 2) **S116**

9. ^{1}H and ^{13}C Spectra of New Compounds **S117–S139**

10. References **S140**

1. General

1.1. General Experimental Information

Dry-degassed CH_2Cl_2 (No. 041-32345, Wako Pure Chemical Industries) and $(CH_2Cl)_2$ (No. 284505, Sigma-Aldrich) for the rhodium-catalyzed [2+2+1] cycloaddition were used as received. Solvents for the synthesis of substrates were dried over Molecular Sieves 4Å (Wako) before use. H_8-BINAP, Segphos, tol-Segphos, and xyl-Segphos were obtained from Takasago International Corporation. $[Rh(cod)_2]BF_4$ was obtained from Umicore AG. **S2**,[1] **S7**,[2] **S9**,[3] **S12**,[4] **S19**,[5] and **1a**[4] were prepared according to the literature. All other commercially available reagents were obtained from TCI Chemicals, Wako Pure Chemical Industries, Sigma-Aldrich, and Kanto Chemicals, and used as received unless otherwise noted. Silica gel column chromatography was performed using silica gel [Silica Gel 60 N (spherical, neutral), Kanto Chemicals] and JIS (Japanese Industrial Standards) special grade solvents. Silica gel preparative thin layer chromatography (PTLC) was performed using silica gel (Wakogel® B-5F) and JIS special grade solvents. All reactions were carried out under an atmosphere of argon or nitrogen in oven-dried glassware with magnetic stirring.

1.2. General Analytical Information

All compounds were characterized by ^{1}H, and ^{13}C NMR spectroscopy. Copies of the ^{1}H, and ^{13}C, NMR spectra for all new compounds can be found in section 9 "^{1}H, and ^{13}C NMR Spectra of New Compounds" of the Supplementary Materials. All previously unreported compounds were additionally characterized by high-resolution mass spectrometry (HRMS). ^{1}H, and ^{13}C NMR data were collected on a Bruker AVANCE III HD 400 at ambient temperature. All ^{1}H NMR experiments are reported in δ units, parts per million (ppm), and were measured relative to the signals for residual chloroform (7.26 ppm). All ^{13}C NMR spectra are reported in ppm relative to deuterochloroform (77.16 ppm) and were obtained with ^{1}H decoupling. HRMS analyses were performed on a Bruker micrOTOF Focus II instrument. Melting points were determined on a Mettler MP50 and are uncorrected. The UV/Vis absorption spectra were recorded on a JASCO V-670 spectrometer and a JASCO V-630 spectrophotometer with a resolution of 0.5 nm.

2. Synthetic Experiments (Figure 2)

2.1. Synthesis of Diynes and Tetrayne

5'-Mehtyl-2,2''-diethynyl-1,1':3',1''-terphenyl (1a)[4]

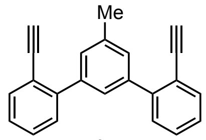

1a

This compound was prepared according to the literature.[4]

5'-Chloro-2,2''-diethynyl-1,1':3',1''-terphenyl (1b)

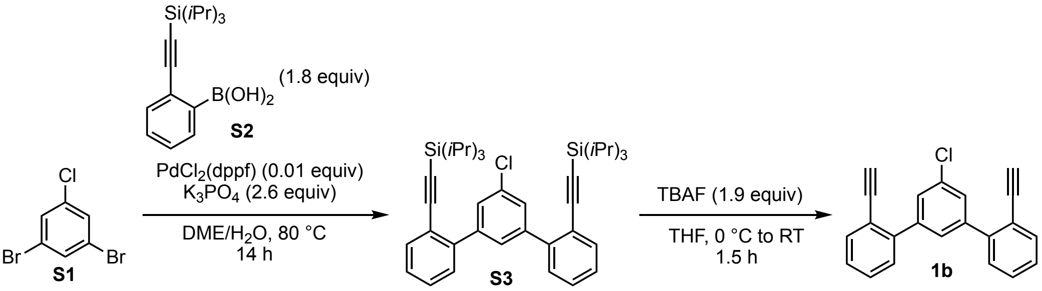

A solution of **S1** (0.754 g, 2.79 mmol), **S2**[1] (1.50 g, 4.95 mmol), $PdCl_2$(dppf) (0.0185 g, 0.025 mmol), and K_3PO_4 (1.57 g, 7.37 mmol) in degassed 1,2-dimethoxyethane (DME)/H_2O (4:1, 38 mL) was stirred at 80 °C for 14 h. The reaction mixture was diluted with water and extracted with ethyl acetate. The organic layer was washed with brine, dried over Na_2SO_4, filtered, and concentrated. The residue was passed through silica gel column chromatography (eluent: *n*-hexane) to give crude **S3**, which was used in the next step without further purification.

To a solution of the crude **S3** in THF (35 mL) was added tetrabutylammonium fluoride (TBAF, 5.2 mL, 5.2 mmol, 1.0 mol/L in THF) at 0 °C. After stirring at room temperature for 1.5 h, the reaction mixture was diluted with water and extracted with CH_2Cl_2. The organic layer was washed with brine, dried over Na_2SO_4, filtered, and concentrated. The residue was purified by silica gel column chromatography (eluent: *n*-hexane/ CH_2Cl_2 = 6:1) to give **1b** (0.551 g, 1.76 mmol, 63% yield).

Pale yellow solid; 87.2–88.1 °C; ^{1}H NMR (400 MHz, $CDCl_3$) δ 7.65–7.57 (m, 5H), 7.44–7.40 (m, 4H), 7.36–7.31 (m, 2H), 3.09 (s, 2H),; ^{13}C NMR ($CDCl_3$, 100 MHz) δ 143.0, 141.6, 134.1, 133.5, 129.6, 129.2, 128.6, 128.4, 127.7, 120.6, 82.9, 81.1; HRMS (ESI) calcd for $C_{22}H_{13}ClNa$ $[M+H]^+$ 335.0603, found 335.0598 (−1.49 ppm).

1,3-Dibromo-5-(*tert*-butoxy)benzene (S5)

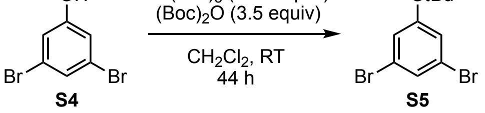

A solution of **S4** (2.50 g, 9.93 mmol), $Sc(OTf)_3$ (0.246 g, 0.500 mmol), and $(Boc)_2O$ (7.64 g, 35.0 mmol) in degassed CH_2Cl_2 (20 mL) was stirred at room temperature for 44 h. The reaction mixture was diluted with water and extracted with ethyl acetate. The organic layer was washed with brine, dried over Na_2SO_4, filtered, and concentrated. The residue was purified by silica gel column chromatography (eluent: *n*-hexane/ EtOAc = 6:1) to give **S5** (1.35 g, 4.39 mmol, 44% yield).

Colorless oil; ^{1}H NMR (400 MHz, $CDCl_3$) δ 7.37 (dd, J = 1.7, 1.7 Hz, 1H), 7.09 (d, J = 1.7 Hz, 2H), 1.36 (s, 9H); ^{13}C NMR ($CDCl_3$, 100 MHz) δ 157.3, 129.0, 125.9, 122.4, 80.4, 28.9; HRMS (APCI) calcd for $C_6H_4Br_2O$ [M–*t*Bu+H]$^+$249.8629, found 249.8706 (+30.8 ppm).

5'-(*tert*-Butoxy)-2,2''-diethynyl-1,1':3',1''-terphenyl (1c)

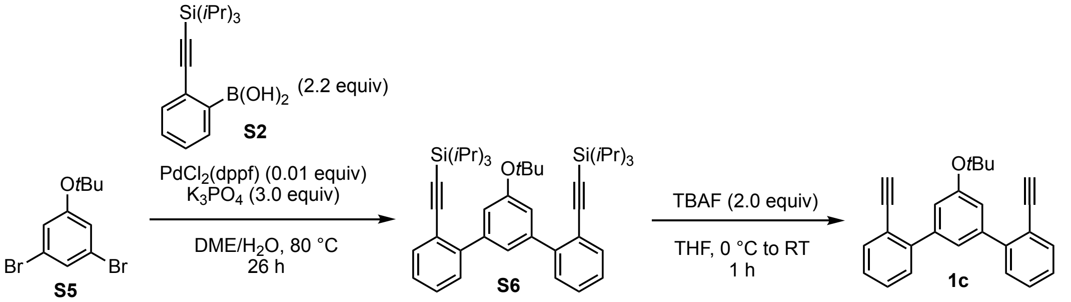

A solution of **S5** (1.25 g, 4.06 mmol), **S2**[1] (2.70 g, 8.94 mmol), $PdCl_2$(dppf) (0.0277 g, 0.038 mmol), and K_3PO_4 (2.59g, 12.20 mmol) in degassed 1,2-dimethoxyethane (DME)/H_2O (4:1, 65 mL) was stirred at 80 °C for 26 h. The reaction mixture was diluted with water and extracted with ethyl acetate. The organic layer was washed with brine, dried over Na_2SO_4, filtered, and concentrated. The residue was passed through silica gel column chromatography (eluent: *n*-hexane/EtOAc = 6:1) to give crude **S6**, which was used in the next step without further purification.

To a solution of the crude **S6** in THF (60 mL) was added tetrabutylammonium fluoride (TBAF, 8.2 mL, 8.2 mmol, 1.0 mol/L in THF) at 0 °C. After stirring at room temperature for 1 h, the reaction mixture was diluted with water and extracted with CH_2Cl_2. The organic layer was washed with brine, dried over Na_2SO_4, filtered, and concentrated. The residue was purified by silica gel column chromatography (eluent: *n*-hexane/ CH_2Cl_2 = 2:1) to give **1c** (0.804 g, 2.29 mmol, 56% yield).

Colorless solid; 138.9–139.6 °C; ^{1}H NMR (400 MHz, $CDCl_3$) δ 7.62 (dd, J = 0.8, 7.7 Hz, 2H), 7.45–7.37 (m, 5H), 7.33–7.27 (m, 4H), 3.08 (s, 2H), 1.42 (s, 9H); ^{13}C NMR ($CDCl_3$, 100 MHz) δ 154.8, 144.1, 140.8, 134.1, 129.8, 129.1, 127.2, 125.2, 124.3, 120.5, 83.4, 80.5, 79.1, 29.2; HRMS (ESI) calcd for $C_{26}H_{22}NaO$ [M+Na]$^+$ 373.1568, found 373.1551 (−4.56 ppm).

5,5''-Di-*tert*-butyl-5'-chloro-2,2''-diethynyl-1,1':3',1''-terphenyl (1d)

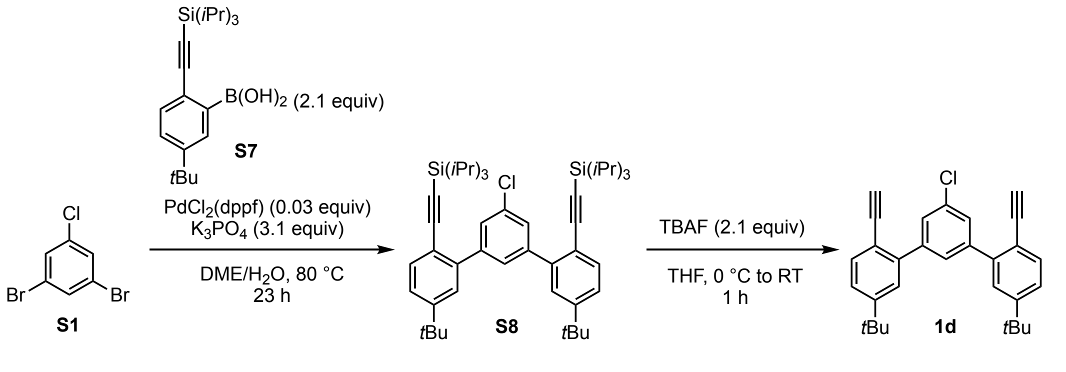

A solution of **S1** 0.0931 g, 0.34 mmol), **S7**[2] (0.252 g, 0.70 mmol), $PdCl_2$(dppf) (0.0254 g, 0.035 mmol), and K_3PO_4 (0.219 g, 1.03 mmol) in degassed 1,2-dimethoxyethane (DME)/H_2O (4:1, 5 mL) was stirred at 80 °C for 23 h. The reaction mixture was diluted with water and extracted with ethyl acetate. The organic layer was washed with brine, dried over Na_2SO_4, filtered, and concentrated. The residue was passed through silica gel column chromatography (eluent: *n*-hexane) to give crude **S8**, which was used in the next step without further purification.

To a solution of the crude **S8** in THF (5 mL) was added tetrabutylammonium fluoride (TBAF, 0.8 mL, 0.8 mmol, 1.0 mol/L in THF) at 0 °C. After stirring at room temperature for 1 h, the reaction

mixture was diluted with water and extracted with EtOAc. The organic layer was washed with brine, dried over Na_2SO_4, filtered, and concentrated. The residue was purified by silica gel column chromatography (eluent: *n*-hexane/ EtOAc = 95:5) to give **1d** (0.0703 g, 0.165 mmol, 17% yield).

Pale yellow solid; Mp 60.7–61.6 °C; ^1H NMR (400 MHz, $CDCl_3$) δ 7.66 (dd, J = 1.4, 1.4 Hz, 1H), 7.63 (d, J = 1.4 Hz, 2H), 7.57 (d, J = 8.2 Hz, 2H), 7.43 (d, J = 2.0 Hz, 2H), 7.36 (dd, J = 2.0, 8.1 Hz, 2H), 3.07 (s, 2H), 1.35 (s, 18H); ^{13}C NMR ($CDCl_3$, 100 MHz) δ 152.7, 142.7, 142.2, 133.9, 133.4, 128.8, 128.4, 126.8, 124.9, 117.7, 83.1, 80.1, 35.1, 31.3; HRMS (ESI) calcd for $C_{30}H_{29}ClNa$ $[M+Na]^+$ 447.1855, found 447.1850 (−7.83 ppm).

(5-Methoxy-2-((triisopropylsilyl)ethynyl)phenyl)boronic acid (S10)

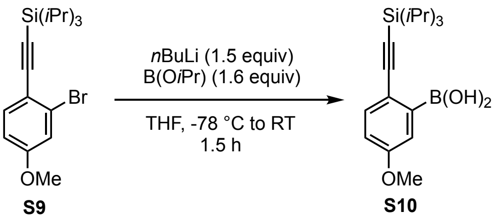

A 1.6 M solution of *n*-BuLi (3.0 mL, 4.53 mmol, 1.51 mol/L in THF) was added dropwise to a solution of **S9**[3] (1.12 g, 3.04 mmol) in THF (10 mL) at –78 °C. After stirring at –78 °C for 1.5 h, $B(Oi\text{-}Pr)_3$ (0.902 g, 4.80 mmol) was added at –78 °C. The reaction mixture was stirred for 2 h while the temperature was raised to room temperature. The reaction was quenched with 1 M aqueous HCl solution. After stirring at room temperature for 1 h, the solution was extracted with ethyl acetate, dried over Na_2SO_4, filtered, and concentrated. The residue was purified by silica gel column chromatography (eluent: *n*-hexane/EtOAc = 20:1) to give **S10** (0.672 g, 2.02 mmol, 66% yield).

Colorless solid; Mp 75.6–76.4 °C; ^1H NMR ($CDCl_3$, 400 MHz) δ 7.54 (d, J = 2.8 Hz, 1H), 7.46 (d, J = 8.5 Hz, 1H), 6.94 (dd, J = 2.8, 8.5 Hz, 1H), 3.84 (s, 3H), 1.17–1.13 (m, 21H); ^{13}C NMR ($CDCl_3$, 100 MHz) δ 159.7, 134.8, 119.9, 119.2, 117.4, 108.7, 94.3, 55.5, 18.7, 11.5. The carbon directly attached to the boron atom was not detected, likely due to quadrupolar relaxation.; HRMS (ESI) calcd for $C_{18}H_{29}BNaO_3Si$ $[M+Na]^+$ 355.1877, found 355.1868 (−2.53 ppm).

5'-Chloro-2,2''-diethynyl-5,5''-dimethoxy-1,1':3',1''-terphenyl (1e)

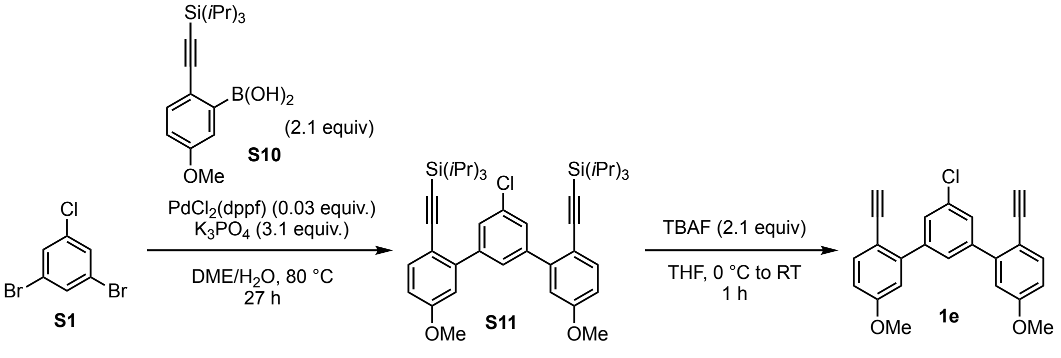

A solution of **S1** (0.626 g, 2.32 mmol), **S10** (1.58 g, 4.76 mmol), $PdCl_2(dppf)$ (0.0552 g, 0.075 mmol), and K_3PO_4 (1.52 g, 7.16 mmol) in degassed 1,2-dimethoxyethane (DME)/H_2O (4:1, 35 mL) was stirred at 80 °C for 27 h. The reaction mixture was diluted with water and extracted with ethyl acetate. The organic layer was washed with brine, dried over Na_2SO_4, filtered, and concentrated. The residue was passed through silica gel column chromatography (eluent: *n*-hexane/EtOAc = 10:1) to give crude **S11**, which was used in the next step without further purification.

To a solution of the crude **S11** in THF (16 mL) was added tetrabutylammonium fluoride (TBAF, 4.9 mL, 4.9 mmol, 1.0 mol/L in THF) at 0 °C. After stirring at room temperature for 1 h, the reaction mixture was diluted with water and extracted with EtOAc. The organic layer was washed with brine,

dried over Na_2SO_4, filtered, and concentrated. The residue was purified by silica gel column chromatography (eluent: *n*-hexane/ DCM = 10:1) to give **1e** (0.767 g, 2.06 mmol, 89% yield). Colorless solid; Mp 121.8–122.6 °C; ^{1}H NMR ($CDCl_3$, 400 MHz) δ 7.67–7.61 (m, 3H), 7.52 (d, J = 8.6 Hz, 2H), 6.92 (d, J = 2.3 Hz, 2H), 6.83 (dd, J = 2.4, 8.5 Hz, 2H), 3.80 (s, 6H), 3.02 (s, 2H); ^{13}C NMR ($CDCl_3$, 100 MHz) δ 160.1, 144.4, 141.5, 135.5, 133.4, 128.4 (2C), 115.0, 113.6, 112.7, 83.0, 79.4, 55.4; HRMS (ESI) calcd for $C_{24}H_{17}ClNaO_2$ $[M+Na]^+$ 395.0815, found 395.0830 (+3.80 ppm).

1,1'-(5-Chloro-1,3-phenylene)bis(2-ethynylnaphthalene) (1f)

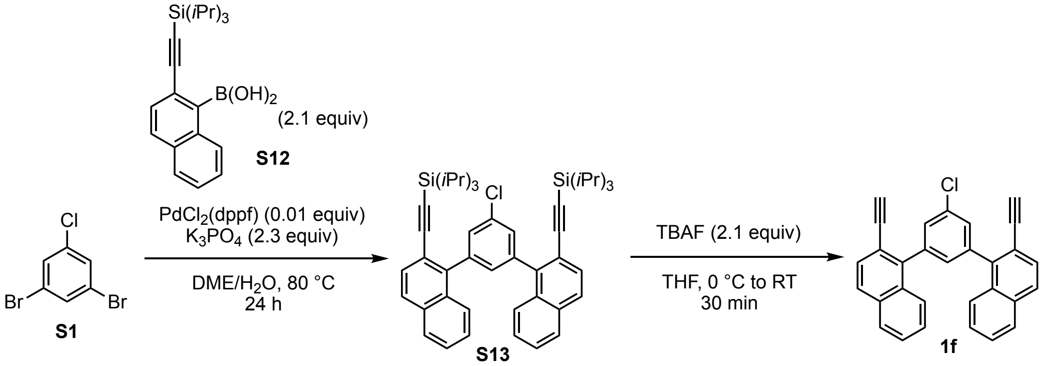

A solution of **S1** (0.407 g, 1.51 mmol), **S12**[4] (1.11 g, 3.16 mmol), $PdCl_2$(dppf) (0.0120 g, 0.016 mmol), and K_3PO_4 (0.749 g, 3.53 mmol) in degassed 1,2-dimethoxyethane (DME)/H_2O (4:1, 30 mL) was stirred at 80 °C for 24 h. The reaction mixture was diluted with water and extracted with ethyl acetate. The organic layer was washed with brine, dried over Na_2SO_4, filtered, and concentrated. The residue was passed through silica gel column chromatography (eluent: *n*-hexane/EtOAc = 10:1) to give crude **S13**, which was used in the next step without further purification.

To a solution of the crude **S13** in THF (16 mL) was added tetrabutylammonium fluoride (TBAF, 3.2 mL, 3.2 mmol, 1.0 mol/L in THF) at 0 °C. After stirring at room temperature for 30 min, the reaction mixture was diluted with water and extracted with EtOAc. The organic layer was washed with brine, dried over Na_2SO_4, filtered, and concentrated. The residue was purified by silica gel column chromatography (eluent: *n*-hexane/ EtOAc = 10:1) followed by gel permeation chromatography (GPC, eluent: CH_3Cl) followed by silica gel PTLC (n-hexane/CH_2Cl_2 = 4:1) to give **1f** (0.0723 g, 0.175 mmol, 12% yield).

Yellow solid; Mp 71.3–72.2 °C; ^{1}H NMR ($CDCl_3$, 400 MHz) δ 7.86–7.68 (m, 7H), 7.60 (d, J = 8.5 Hz, 2H), 7.54–7.34 (m, 8H), 3.12 (major conformer) 3.04 (minor conformer) (total, 2H); ^{13}C NMR ($CDCl_3$, 100 MHz) δ 142.1, 140.3, 140.2, 133.81, 133.76. 133.41, 133.37, 132.05, 131.97, 131.2, 131.1, 129.81, 129.78, 129.02, 128.99, 128.3, 128.16, 128.13, 128.10, 127.0, 126.93, 126.86, 126.80, 126.4, 119.39, 119.14, 83.5, 83.2, 81.7, 81.4; HRMS (ESI) calcd for $C_{30}H_{17}ClNa$ $[M+Na]^+$ 435.0916, found 435.0911 (−1.15 ppm).

((3'-Bromo-5-(*tert*-butyl)-5'-chloro-[1,1'-biphenyl]-2-yl)ethynyl)triisopropylsilane (S14)

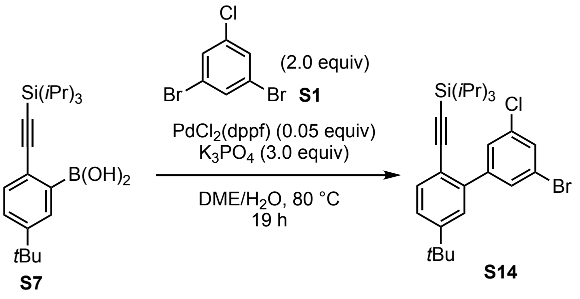

A solution of **S7** (3.56 g, 9.92 mmol), **S1** (5.41 g, 20.0 mmol), $PdCl_2(dppf)$ (0.398 mg, 0.54 mmol), and K_3PO_4 (6.42 g, 30.3 mmol) in degassed DME/H_2O (4:1, 250 mL) was stirred at 80 °C for 19 h. The reaction mixture was diluted with water and extracted with ethyl acetate. The organic layer was washed with brine, dried over Na_2SO_4, filtered, and concentrated. The residue was passed through silica gel column chromatography (eluent: *n*-hexane) to give **S14** (1.71 g, 3.39 mmol, 34% yield).

Yellow oil; ^1H NMR ($CDCl_3$, 400 MHz) δ 7.58 (dd, J = 1.6, 1.6 Hz, 1H), 7.53 (d, J = 8.3 Hz, 1H), 7.49 (d, J = 1.6 Hz, 2H), 7.35 (dd, J = 2.0, 8.2 Hz, 1H), 7.27 (d, J = 1.8 Hz, 1H), 1.33 (s, 9H), 1.04–1.00 (s, 21H); ^{13}C NMR ($CDCl_3$, 100 MHz) δ 152.2, 144.6, 141.2, 134.7, 133.7, 130.8, 130.1, 128.5, 126.2, 125.2, 122.3, 119.3, 105.6, 94.5, 35.0, 31.3, 18.7, 11.4; HRMS (ESI) calcd for $C_{27}H_{36}BrClSi$ $[M+Na]^+$ 525.1356, found 525.1350 (−1.14 ppm).

((5-(*tert*-Butyl)-3'-chloro-5'-(4,4,5,5-tetramethyl-1,3,2-dioxaborolan-2-yl)-[1,1'-biphenyl]-2-yl)ethynyl)triisopropylsilane (S15)

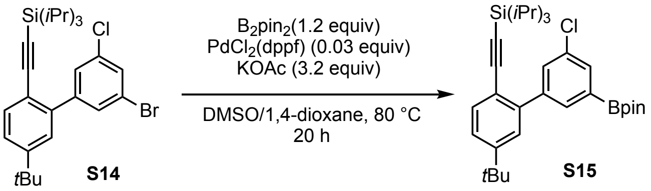

A solution of **S14** (1.51 g, 3.00 mmol), B_2pin_2 (0.935 g, 3.68 mmol), $PdCl_2(dppf)$ (0.0599 mg, 0.082 mmol), and KOAc (0.940 g, 9.57 mmol) in degassed DMSO/1,4-dioxane (1:1, 30 mL) was stirred at 80 °C for 20 h. The reaction mixture was diluted with water and extracted with ethyl acetate. The organic layer was washed with brine, dried over Na_2SO_4, filtered, and concentrated. The residue was passed through silica gel column chromatography (eluent: *n*-hexane/EtOAc = 20:1) to give **S15** (1.32 g, 2.39 mmol, 80% yield).

Colorless oil; ^1H NMR ($CDCl_3$, 400 MHz) δ 7.79–7.77 (m, 1H), 7.74 (dd, J = 0.8, 2.1 Hz, 1H), 7.63 (dd, J = 1.9, 1.9 Hz, 1H), 7.51 (d, J = 8.0 Hz, 1H), 7.33–7.28 (m, 2H), 1.34 (s, 12H), 1.33 (s, 9H), 1.02–0.95 (m, 21H); ^{13}C NMR ($CDCl_3$, 100 MHz) δ 151.9, 142.82, 142.80, 133.7, 133.6, 133.4 (2C), 132.2, 126.4, 124.6, 119.4, 106.1, 93.6, 84.2, 35.0, 31.3, 25.0, 18.7, 11.4 The carbon directly attached to the boron atom was not detected, likely due to quadrupolar relaxation.; HRMS (ESI) calcd for $C_{33}H_{48}BClNaO_2Si$ $[M+Na]^+$ 573.3103, found 573.3123 (+3.49 ppm).

5,5''''-Di-*tert*-butyl-5',5'''-dichloro-2,2'',2'''',5''-tetraethynyl-1,1':3',1'':4'',1''':3''',1''''-quinquephenyl (7)

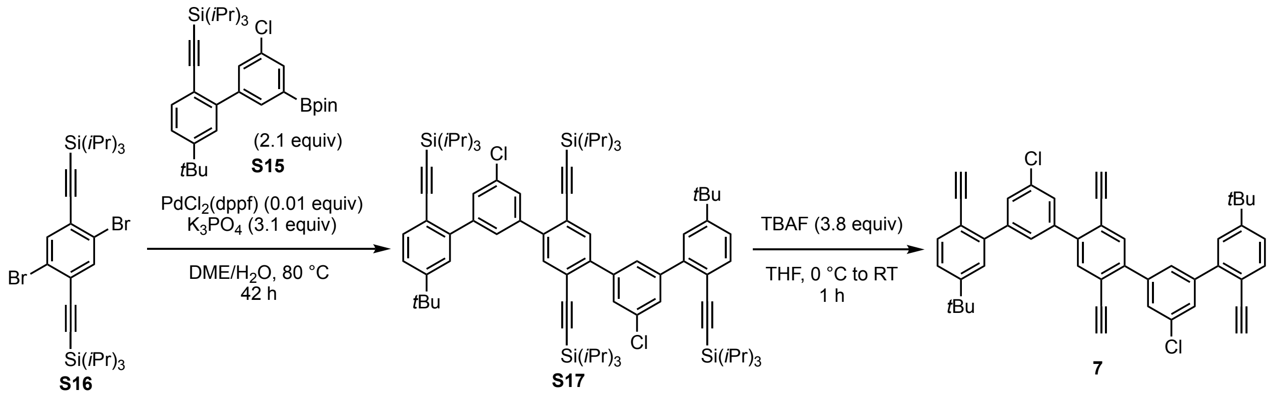

A solution of **S16** (0.601 g, 1.01 mmol), **S15** (1.14 g, 2.07 mmol), $PdCl_2$(dppf) (0.0423 g, 0.06 mmol), and K_3PO_4 (0.655 g, 3.09 mmol) in degassed 1,2-dimethoxyethane (DME)/H_2O (4:1, 20 mL) was stirred at 80 °C for 42 h. The reaction mixture was diluted with water and extracted with ethyl acetate. The organic layer was washed with brine, dried over Na_2SO_4, filtered, and concentrated. The residue was passed through silica gel column chromatography (eluent: *n*-hexane/EtOAc = 20:1) to give crude **S17**, which was used in the next step without further purification.

To a solution of the crude **S17** in THF (27 mL) was added tetrabutylammonium fluoride (TBAF, 3.8 mL, 3.8 mmol, 1.0 mol/L in THF) at 0 °C. After stirring at room temperature for 30 min, the reaction mixture was diluted with water and extracted with EtOAc. The organic layer was washed with brine, dried over Na_2SO_4, filtered, and concentrated. The residue was purified by silica gel column chromatography (eluent: *n*-hexane/CH_2Cl_2 = 3:1) to give **7** (0.202 g, 0.306 mmol, 30% yield).

Colorless solid; Mp 173.9 (decomposition); ^{1}H NMR ($CDCl_3$, 400 MHz) δ 7.71 (s, 2H), 7.69 (dd, J = 1.5, 1.5 Hz, 2H), 7.66 (d, J = 1.5 Hz, 4H), 7.57 (d, J = 8.1 Hz, 2H), 7.42 (d, J = 2.0 Hz, 2H), 7.37 (dd, J = 2.0, 8.1 Hz, 2H), 3.23 (s, 2H), 3.08 (s, 2H), 1.35 (s, 18H).; ^{13}C NMR ($CDCl_3$, 100 MHz) δ152.7, 142.47, 142.45, 140.1, 135.2, 133.9, 133.7, 129.0, 128.5, 128.2, 126.6, 125.0, 121.4, 117.7, 83.0, 82.9, 82.2, 80.3, 35.1, 31.3.; HRMS (ESI) calcd for $C_{46}H_{36}Cl_2Na$ $[M+Na]^+$681.2092, found 681.2086 (−0.88 ppm).

5'-Chloro-2-ethynyl-2''-(p-tolylethynyl)-1,1':3',1''-terphenyl (1g)

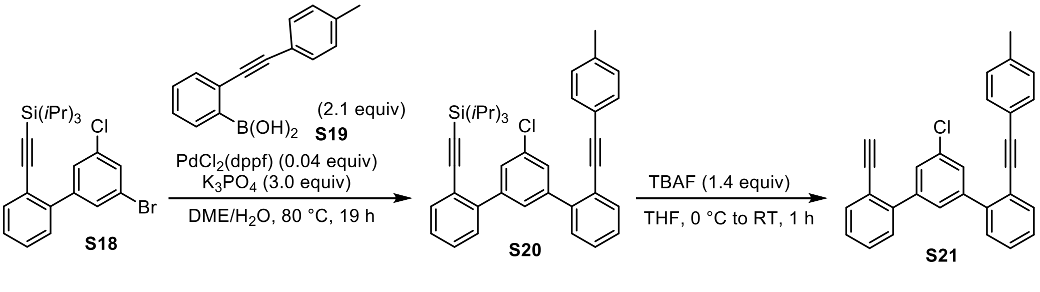

A solution of **S18**[4] (0.225 g, 0.50 mmol), **S19**[5] (0.148 g, 0.63 mmol), $PdCl_2$(dppf) (0.0162 g, 0.022 mmol), and K_3PO_4 (0.149 g, 1.52 mmol) in degassed 1,2-dimethoxyethane (DME)/H_2O (4:1, 10 mL) was stirred at 80 °C for 19 h. The reaction mixture was diluted with water and extracted with ethyl acetate. The organic layer was washed with brine, dried over Na_2SO_4, filtered, and concentrated. The residue was passed through silica gel column chromatography (eluent: *n*-hexane/EtOAc = 10:1) to give crude **S20**, which was used in the next step without further purification.

To a solution of the crude **S20** in THF (6 mL) was added tetrabutylammonium fluoride (TBAF, 0.69 mL, 0.69 mmol, 1.0 mol/L in THF) at 0 °C. After stirring at room temperature for 1 h, the reaction mixture was diluted with water and extracted with EtOAc. The organic layer was washed

with brine, dried over Na_2SO_4, filtered, and concentrated. The residue was purified by silica gel column chromatography (eluent: *n*-hexane/ EtOAc = 95:5), silica gel PTLC (eluent: *n*-hexane/ EtOAc = 10:1) to give **1g** (0.161 g, 0.40 mmol, 80% yield).

Pale yellow oil; ^{1}H NMR ($CDCl_3$, 400 MHz) δ 7.74 (ddd, J = 1.7, 1.7, 8.6 Hz, 2H), 7.67–7.61 (m, 3H), 7.47 (dd, J = 1.3, 7.5 Hz, 1H), 7.42–7.29 (m, 5H), 7.25–7.22 (m, 2H), 7.08 (d, J = 7.8 Hz, 2H), 3.10 (s, 1H), 2.33 (s, 3H); ^{13}C NMR ($CDCl_3$, 100 MHz) δ143.1, 142.2, 142.0, 141.6, 138.6, 134.1, 133.4, 133.0, 131.5, 129.7, 129.4, 129.21, 129.18, 128.8, 128.62, 128.59, 128.4, 127.8, 127.7, 122.0, 120.6, 120.2, 93.4, 88.4, 82.9, 81.0, 21.6; HRMS (ESI) calcd for $C_{29}H_{19}ClNa$ $[M+Na]^+$ 425.1073, found 425.1067 (−1.41 ppm).

2.2. Synthesis of Electron-Deficient Fused Azulenes

Dimethyl 2-methylbenzo[7,8]azuleno[6,5,4-jk]fluorene-9,10-dicarboxylate (6aa)

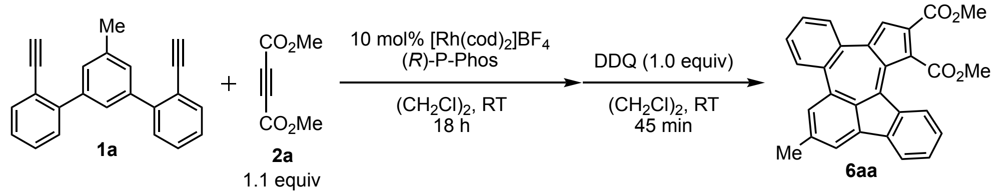

1a **2a** 1.1 equiv **6aa**

(*R*)-P-Phos (6.4 mg, 0.010 mmol) and $[Rh(cod)_2]BF_4$ (4.2 mg, 0.010 mmol) were dissolved in CH_2Cl_2 (1.0 mL), and the mixture was stirred at room temperature for 10 min. H_2 was introduced to the resulting solution in a Schlenk tube. After stirring at room temperature for 30 min, the resulting mixture was concentrated under reduced pressure. The residue was dissolved in $(CH_2Cl)_2$ (9.0 mL) and added to a solution of **1a** (29.1 mg, 0.100 mmol) and **2a** (15.5 mg, 0.109 mmol) in $(CH_2Cl)_2$ (1.0 mL). After stirring at room temperature for 18 h, the resulting mixture was added 2,3-dichloro-5,6-dicyano-1,4-benzoquinone (DDQ) (22.8 mg, 0.100 mmol). After stirring at room temperature for 45 min, the resulting mixture was concentrated and purified by silica gel PTLC (eluent: CH_2Cl_2) to give **6aa** (20.8 mg, 0.0481 mmol, 48% yield).

Black solid; Mp 222.1–222.9 °C; ^1H NMR ($CDCl_3$, 400 MHz) δ 8.10 (dd, J = 1.5, 7.9 Hz, 1H), 7.99 (dd, J = 1.4, 7.9 Hz, 1H), 7.76 (d, J = 7.6 Hz, 1H), 7.67 (s, 1H), 7.58 (d, J = 7.5 Hz, 1H), 7.53 (s, 1H), 7.52–7.44 (m, 3H), 7.39 (ddd, J = 1.0, 7.5, 7.5 Hz, 1H), 7.20 (ddd, J = 1.0, 7.6, 7.6 Hz, 1H), 3.90 (s, 3H), 3.68 (s, 3H), 2.51 (s, 3H); ^{13}C NMR ($CDCl_3$, 100 MHz) δ 168.1, 164.5, 155.9, 142.52, 142.45, 141.0, 139.5, 138.4, 137.1, 136.9, 136.1, 134.9, 134.2, 133.3, 132.1, 130.8, 129.5, 129.0, 128.3, 127.9, 127.5, 127.1, 124.4, 121.3, 120.7, 52.5, 52.3, 22.3; HRMS (ESI) calcd for $C_{29}H_{20}NaO_4$ $[M+Na]^+$ 455.1259 found 455.1247 (−2.64 ppm).

Diethyl 2-methylbenzo[7,8]azuleno[6,5,4-jk]fluorene-9,10-dicarboxylate (6ab)

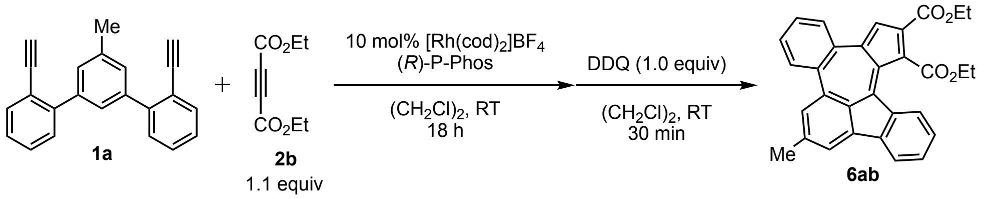

1a **2b** 1.1 equiv **6ab**

(*R*)-P-Phos (6.4 mg, 0.010 mmol) and $[Rh(cod)_2]BF_4$ (4.1 mg, 0.010 mmol) were dissolved in CH_2Cl_2 (1.0 mL), and the mixture was stirred at room temperature for 10 min. H_2 was introduced to the resulting solution in a Schlenk tube. After stirring at room temperature for 30 min, the resulting mixture was concentrated under reduced pressure. The residue was dissolved in $(CH_2Cl)_2$ (9.0 mL) and added to a solution of **1a** (29.2 mg, 0.100 mmol) and **2b** (18.7 mg, 0.110 mmol) in $(CH_2Cl)_2$ (1.0 mL). After stirring at room temperature for 18 h, the resulting mixture was added 2,3- dichloro-5,6-dicyano-1,4-benzoquinone (DDQ) (22.9 mg, 0.101 mmol). After stirring at room temperature for 30 min, the resulting mixture was concentrated, passed through silica gel column chromatography (eluent: CH_2Cl_2), and purified by silica gel PTLC (eluent: CH_2Cl_2) to give **6ab** (20.9 mg, 0.0454 mmol, 45% yield).

Black solid; Mp 76.5–77.4 °C; ^1H NMR ($CDCl_3$, 400 MHz) δ 8.10 (dd, J = 1.5, 7.9 Hz, 1H), 7.97 (d, J = 7.8 Hz, 1H), 7.78 (d, J = 7.6 Hz, 1H), 7.65 (s, 1H) 7.56 (d, J = 7.5 Hz, 1H), 7.53–7.43 (m, 4H), 7.38 (dd, J = 7.4, 7.4 Hz, 1H), 7.18 (ddd, J = 0.8, 7.6, 7.6 Hz, 1H), 4.36 (q, J = 7.1, 7.1, 7.1 Hz, 2H), 4.09 (q, J = 7.2, 7.2, 7.2 Hz, 2H), 2.50 (s, 3H), 1.38 (t, J = 7.1, 7.1 Hz, 3H), 1.06 (t, J = 7.2, 7.2 Hz, 3H); ^{13}C NMR ($CDCl_3$, 100 MHz) δ 167.8, 164.2, 155.7, 142.4, 142.3, 140.9, 139.7, 138.6, 136.9, 136.8, 136.2, 135.4, 134.2, 133.3, 131.9, 130.8, 129.5, 129.0, 128.4, 128.2, 127.9, 127.5, 127.0, 124.6,

121.2, 120.5, 61.7, 61.1, 22.3, 14.4, 13.8; HRMS (ESI) calcd for $C_{31}H_{24}NaO_4$ $[M+Na]^+$ 483.1572 found 483.1558 (−2.90 ppm).

Di-*tert*-butyl 2-methylbenzo[7,8]azuleno[6,5,4-jk]fluorene-9,10-dicarboxylate (6ac)

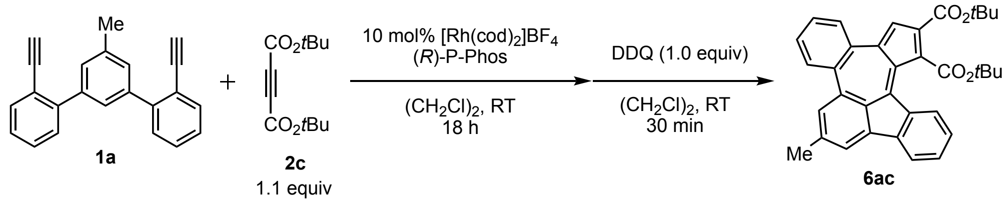

(*R*)-P-Phos (6.4 mg, 0.010 mmol) and $[Rh(cod)_2]BF_4$ (4.1 mg, 0.010 mmol) were dissolved in CH_2Cl_2 (1.0 mL), and the mixture was stirred at room temperature for 10 min. H_2 was introduced to the resulting solution in a Schlenk tube. After stirring at room temperature for 30 min, the resulting mixture was concentrated under reduced pressure. The residue was dissolved in $(CH_2Cl)_2$ (9.0 mL) and added to a solution of **1a** (29.2 mg, 0.100 mmol) and **2c** (24.8 mg, 0.110 mmol) in $(CH_2Cl)_2$ (1.0 mL). After stirring at room temperature for 18 h, the resulting mixture was added 2,3-dichloro-5,6-dicyano-1,4-benzoquinone (DDQ) (22.9 mg, 0.101 mmol). After stirring at room temperature for 30 min, the resulting mixture was concentrated and purified by silica gel PTLC (eluent: CH_2Cl_2), followed by gel permeation chromatography (GPC, eluent: CH_3Cl) to give **6ac** (4.8 mg, 0.0093 mmol, 9% yield).

Black solid; Mp 114.7–115.5 °C; ^1H NMR ($CDCl_3$, 400 MHz) δ 8.15 (dd, J = 1.5, 7.9 Hz, 1H), 8.05 (dd, J = 1.6, 7.9 Hz, 1H), 7.88 (d, J = 7.5 Hz, 1H), 7,74 (s, 1H), 7.59 (d, J = 7.4 Hz, 1H), 7.54–7.44 (m, 3H), 7.42 (s, 1H), 7.38 (ddd, J = 1.0, 7.5, 7.5 Hz, 1H), 7.17 (ddd, J = 1.0, 7.6, 7.6 Hz, 1H), 2.55 (s, 3H), 1.62 (s, 9H), 1.42 (s, 9H); ^{13}C NMR ($CDCl_3$, 100 MHz) δ 165.8, 163.8, 154.9, 142.3, 141.9, 141.1, 139.4, 138.68, 138.65, 137.1, 136.64, 136.62, 134.1, 133.3, 131.6, 130.6, 129.8, 129.6, 128.8, 127.7, 127.39, 127.37, 124.6, 121.2, 120.4, 82.1, 81.5, 28.5, 28.2, 22.3; HRMS (ESI) calcd for $C_{35}H_{32}NaO_4$ $[M+Na]^+$ 539.2198, found 539.2158 (−7.42 ppm).

Dimethyl 2-chlorobenzo[7,8]azuleno[6,5,4-jk]fluorene-9,10-dicarboxylate (6ba)

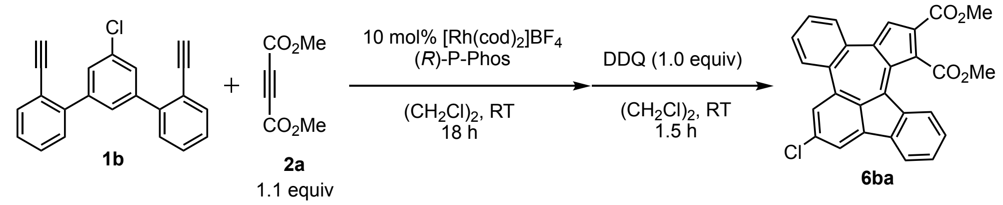

(*R*)-P-Phos (6.4 mg, 0.010 mmol) and $[Rh(cod)_2]BF_4$ (4.1 mg, 0.010 mmol) were dissolved in CH_2Cl_2 (1.0 mL), and the mixture was stirred at room temperature for 10 min. H_2 was introduced to the resulting solution in a Schlenk tube. After stirring at room temperature for 30 min, the resulting mixture was concentrated under reduced pressure. The residue was dissolved in $(CH_2Cl)_2$ (9.0 mL) and added to a solution of **1b** (31.1 mg, 0.099 mmol) and **2a** (15.6 mg, 0.110 mmol) in $(CH_2Cl)_2$ (1.0 mL). After stirring at room temperature for 18 h, the resulting mixture was added 2,3- dichloro-5,6-dicyano-1,4-benzoquinone (DDQ) (22.9 mg, 0.101 mmol). After stirring at room temperature for 90 min, the resulting mixture was concentrated, passed through silica gel column chromatography (eluent: CH_2Cl_2), and purified by silica gel PTLC (eluent: CH_2Cl_2) to give **6ba** (22.7 mg, 0.0501 mmol, 50% yield).

Black solid; Mp 217.0–217.6 °C; ^1H NMR ($CDCl_3$, 400 MHz) δ 8.08 (dd, J = 1.4, 7.9 Hz, 1H), 7.91 (dd, J = 1.1, 7.9 Hz, 1H), 7.83 (d, J = 1.6 Hz, 1H), 7.76 (d, J = 7.6 Hz, 1H), 7.61–7.57 (m, 2H), 7.56–7.45 (m, 3H), 7.43 (ddd, J = 0.8, 7.5, 7.5 Hz, 1H), 7.28–7.22 (m, 1H), 3.90 (s, 3H), 3.68 (s, 3H); ^{13}C NMR ($CDCl_3$, 100 MHz) δ 167.8, 164.3, 154.3, 142.4, 141.3, 140.4, 138.3, 137.9, 137.8, 136.8,

136.4, 135.8, 134.1, 132.3, 132.2, 130.8, 129.6, 129.5, 128.4, 128.3, 127.9, 127.8, 127.2, 125.3, 121.1, 120.3, 52.5, 52.4; HRMS (ESI) calcd for $C_{28}H_{17}ClNaO_4$ $[M+Na]^+$ 475.0713, found 475.0701 (−2.53 ppm).

Dimethyl 2-(*tert*-butoxy)benzo[7,8]azuleno[6,5,4-jk]fluorene-9,10-dicarboxylate (6ca)

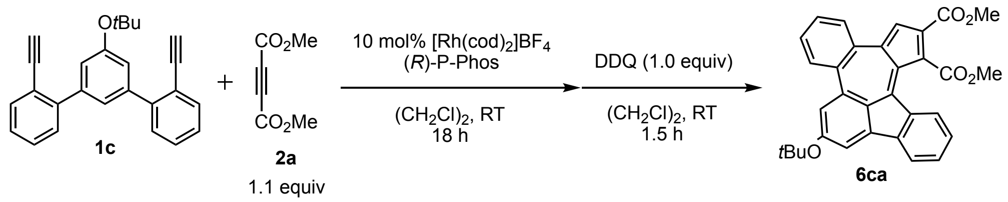

(*R*)-P-Phos (6.4 mg, 0.010 mmol) and $[Rh(cod)_2]BF_4$ (4.1 mg, 0.010 mmol) were dissolved in CH_2Cl_2 (1.0 mL), and the mixture was stirred at room temperature for 10 min. H_2 was introduced to the resulting solution in a Schlenk tube. After stirring at room temperature for 30 min, the resulting mixture was concentrated under reduced pressure. The residue was dissolved in $(CH_2Cl)_2$ (9.0 mL) and added to a solution of **1c** (34.9 mg, 0.100 mmol) and **2a** (15.6 mg, 0.110 mmol) in $(CH_2Cl)_2$ (1.0 mL). After stirring at room temperature for 18 h, the resulting mixture was added 2,3- dichloro-5,6-dicyano-1,4-benzoquinone (DDQ) (22.9 mg, 0.101 mmol). After stirring at room temperature for 90 min, the resulting mixture was concentrated and purified by silica gel PTLC (eluent: CH_2Cl_2) to give **6ca** (20.7 mg, 0.0422 mmol, 42% yield).

Black solid; Mp 189.3–190.1 °C; ^1H NMR ($CDCl_3$, 400 MHz) δ 8.13 (dd, J = 1.5, 7.9 Hz, 1H), 7.98 (dd, J = 1.5, 7.9 Hz, 1H), 7.78 (d, J = 7.6 Hz, 1H), 7.59 (d, J = 7.4 Hz, 1H), 7.56 (s, 1H), 7.55–7.46 (m, 3H), 7.41 (ddd, J = 1.0, 7.5, 7.5 Hz, 1H), 7.32 (d, J = 2.0 Hz, 1H), 7.22 (ddd, J = 1.0, 7.6, 7.6 Hz, 1H), 3.90 (s, 3H), 3.70 (s, 3H), 1.49 (s, 9H); ^{13}C NMR ($CDCl_3$, 100 MHz) δ 168.2, 164.5, 159.5, 142.8, 142.2, 138.9, 138.8, 138.3, 137.0 137.0, 134.6, 134.2, 133.9, 133.1, 132.1, 130.9, 129.4, 129.2, 128.2, 127.8, 127.6, 127.4, 124.4, 121.7, 120.8, 116.0, 80.5, 52.5, 52.3, 29.3; HRMS (ESI) calcd for $C_{32}H_{26}NaO_5$ $[M+Na]^+$ 513.1678, found 513.1677 (−0.19 ppm).

Dimethyl 5,13-di-*tert*-butyl-2-chlorobenzo[7,8]azuleno[6,5,4-jk]fluorene-9,10-dicarboxylate (6da)

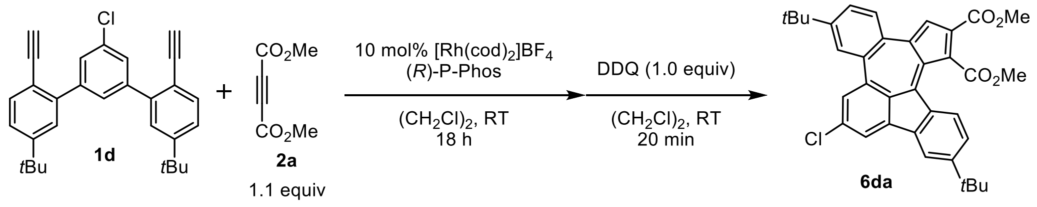

(*R*)-P-Phos (3.2 mg, 0.0050 mmol) and $[Rh(cod)_2]BF_4$ (2.0 mg, 0.0049 mmol) were dissolved in CH_2Cl_2 (1.0 mL), and the mixture was stirred at room temperature for 10 min. H_2 was introduced to the resulting solution in a Schlenk tube. After stirring at room temperature for 30 min, the resulting mixture was concentrated under reduced pressure. The residue was dissolved in $(CH_2Cl)_2$ (4.0 mL) and added to a solution of **1d** (21.3 mg, 0.0501 mmol) and **2a** (7.9 mg, 0.056 mmol) in $(CH_2Cl)_2$ (1.0 mL). After stirring at room temperature for 18 h, the resulting mixture was added 2,3- dichloro-5,6-dicyano-1,4-benzoquinone (DDQ) (11.4 mg, 0.050 mmol). After stirring at room temperature for 15 min, the resulting mixture was concentrated and purified by silica gel PTLC (eluent: CH_2Cl) to give **6da** (18.4 mg, 0.0325 mmol, 65% yield).

Black solid; Mp 126.6–127.6 °C; ^1H NMR ($CDCl_3$, 400 MHz) δ 8.06 (d, J = 8.5 Hz, 1H), 7.92 (d, J = 2.0 Hz, 1H), 7.86 (d, J = 1.3 Hz, 1H), 7.70 (d, J = 8.1 Hz, 1H), 7.67 (d, J = 1.8 Hz, 1H), 7.63 (d, J = 1.4 Hz, 1H), 7.60 (dd, J = 2.1, 8.6 Hz, 1H), 7.51 (s, 1H), 7.29 (dd, J = 1.8, 8.2 Hz, 1H), 3.90 (s, 3H), 3.65 (s, 3H), 1.44 (s, 9H), 1.39 (s, 9H); ^{13}C NMR ($CDCl_3$, 100 MHz) δ 168.0, 164.4, 156.5,

154.5, 150.9, 142.8, 141.4, 139.8, 138.4 137.6, 136.9, 136.8, 135.9, 135.6, 131.8, 130.7, 128.1, 127.9, 127.3, 127.0, 126.0, 125.1, 124.2, 120.0, 118.2, 52.4, 52.3, 35.5, 35.1, 31.4, 31.3; HRMS (ESI) calcd for $C_{36}H_{33}ClNaO_4$ $[M+Na]^+$ 587.1965, found 587.1969 (+0.68 ppm).

Dimethyl 2-chloro-5,13-dimethoxybenzo[7,8]azuleno[6,5,4-jk]fluorene-9,10-dicarboxylate (6ea)

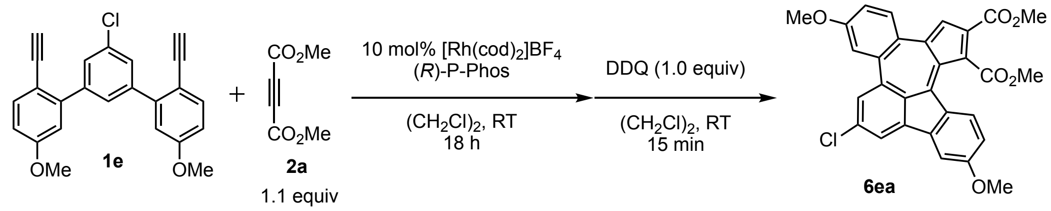

(*R*)-P-Phos (3.2 mg, 0.0050 mmol) and $[Rh(cod)_2]BF_4$ (2.0 mg, 0.0049 mmol) were dissolved in CH_2Cl_2 (1.0 mL), and the mixture was stirred at room temperature for 10 min. H_2 was introduced to the resulting solution in a Schlenk tube. After stirring at room temperature for 30 min, the resulting mixture was concentrated under reduced pressure. The residue was dissolved in $(CH_2Cl)_2$ (4.0 mL) and added to a solution of **1e** (18.6 mg, 0.0499 mmol) and **2a** (7.9 mg, 0.056 mmol) in $(CH_2Cl)_2$ (1.0 mL). After stirring at room temperature for 18 h, the resulting mixture was added 2,3- dichloro-5,6-dicyano-1,4-benzoquinone (DDQ) (11.5 mg, 0.0505 mmol). After stirring at room temperature for 15 min, the resulting mixture was concentrated and purified by silica gel PTLC (eluent: CH_2Cl_2/EtOAc = 95:5) to give **6ea** (7.6 mg, 0.015 mmol, 30% yield).

Brown solid; Mp 270.2–271.2 °C; ^{1}H NMR ($CDCl_3$, 400 MHz) δ 8.03 (d, J = 8.9 Hz, 1H), 7.82 (d, J = 1.5 Hz, 1H), 7.69 (d, J = 8.5 Hz, 1H), 7.58 (d, J = 1.7 Hz, 1H), 7.37 (s, 1H), 7.33 (d, J = 2.6 Hz, 1H), 7.15–7.10 (m, 2H), 6.75 (dd, J = 2.4, 8.5 Hz, 1H), 3.94 (s, 3H), 3.93 (s, 3H), 3.89 (s, 3H), 3.72 (s, 3H); ^{13}C NMR ($CDCl_3$, 100 MHz) δ 168.2, 164.5, 163.8, 158.9, 153.6, 143.6, 142.0, 138.8, 137.4, 137.3, 137.1, 136.8, 135.6, 133.3, 132.6, 130.8, 129.9, 128.1, 127.3, 127.1, 123.0, 120.3, 116.6, 113.4, 113.0, 107.3, 55.9, 55.7, 52.5, 52.3; HRMS (ESI) calcd for $C_{30}H_{21}ClNaO_6$ $[M+Na]^+$ 535.0924, found 535.0927 (−0.93 ppm).

Dimethyl 17-chlorobenzo[c]naphtho[1',2':7,8]azuleno[6,5,4-jk]fluorene-8,9-dicarboxylate (6fa)

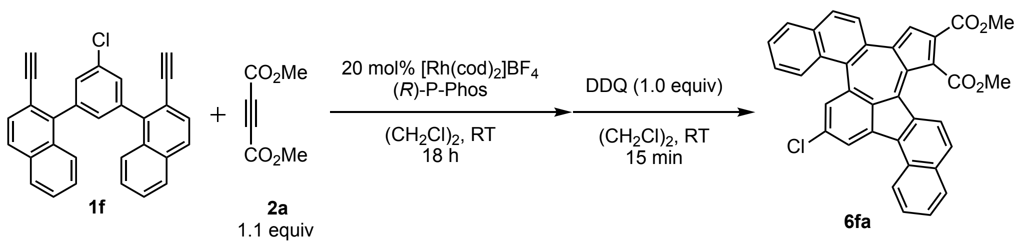

(*R*)-P-Phos (6.4 mg, 0.010 mmol) and $[Rh(cod)_2]BF_4$ (4.1 mg, 0.010 mmol) were dissolved in CH_2Cl_2 (1.0 mL), and the mixture was stirred at room temperature for 10 min. H_2 was introduced to the resulting solution in a Schlenk tube. After stirring at room temperature for 30 min, the resulting mixture was concentrated under reduced pressure. The residue was dissolved in $(CH_2Cl)_2$ (9.0 mL) and added to a solution of **1f** (18.1 mg, 0.0499 mmol) and **2a** (7.9 mg, 0.056 mmol) in $(CH_2Cl)_2$ (1.0 mL). After stirring at room temperature for 18 h, the resulting mixture was added 2,3- dichloro-5,6-dicyano-1,4-benzoquinone (DDQ) (11.5 mg, 0.0505 mmol). After stirring at room temperature for 15 min, the resulting mixture was concentrated and purified by silica gel PTLC (eluent: CH_2Cl_2) to give **6fa** (7.8 mg, 0.0142 mmol, 28% yield). This compound has poor solubility and exhibited low ^{13}C NMR peak intensities.

Black solid; Mp >300 °C; ^{1}H NMR ($CDCl_3$, 400 MHz) δ 8.57 (d, J = 8.4 Hz, 1H), 8.20 (d, J = 1.6

Hz, 1H), 8.15 (d, J = 8.8 Hz, 1H), 8.12–8.07 (m, 1H), 7.98–7.87 (m, 4H), 7.78–7.73 (m, 3H), 7.68 (ddd, J = 1.4, 7.0, 8.4 Hz, 1H), 7.59 (ddd, J = 1.1, 7.0, 8.1 Hz, 1H), 7.55–7.50 (m, 2H), 3.92 (s, 3H), 3.68 (s, 3H); ^{13}C NMR ($CDCl_3$, 100 MHz) 168.0, 164.2, 154.5, 141.4, 140.2, 139.7, 138.2, 137.1, 136.7, 136.3, 135.5, 135.0, 134.0. 133.4, 132.3, 132.05, 132.04 130.2, 129.9, 129.5, 129.4, 129.1, 128.8, 128.28, 128.25, 128.21, 127.9, 127.8, 126.9, 126.5, 125.2, 124.4, 124.0, 123.6, 52.7, 52.4; HRMS (ESI) calcd for $C_{36}H_{21}ClNaO_4$ $[M+Na]^+$ 575.1026 found 575.1021 (−0.87 ppm).

Tetramethyl 5,16-di-tert-butyl-2,13-dichlorodibenzo[3,4:5,6]cyclopenta[7,8]azuleno[2,1-b]benzo[7,8]azuleno[6,5,4-jk]fluorene-9,10,20,21-tetracarboxylate (8)

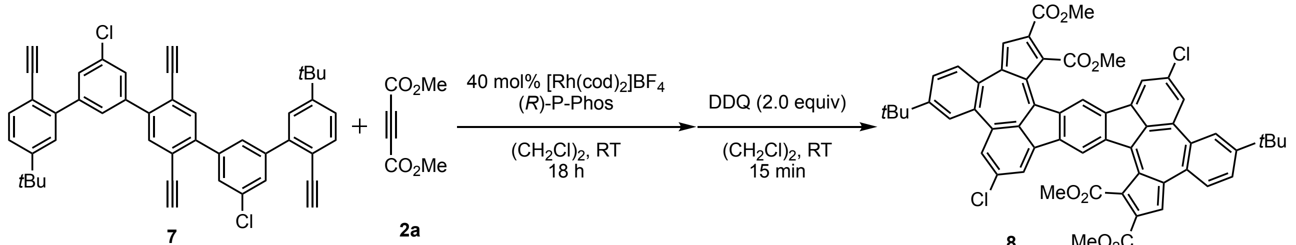

7 2a 2.1 equiv 8

(*R*)-P-Phos (12.9 mg, 0.0200 mmol) and $[Rh(cod)_2]BF_4$ (8.0 mg, 0.0197 mmol) were dissolved in CH_2Cl_2 (1.0 mL), and the mixture was stirred at room temperature for 10 min. H_2 was introduced to the resulting solution in a Schlenk tube. After stirring at room temperature for 30 min, the resulting mixture was concentrated under reduced pressure. The residue was dissolved in $(CH_2Cl)_2$ (9.0 mL) and added to a solution of **7** (33.1 mg, 0.0502 mmol) and **2a** (14.9 mg, 0.105 mmol) in $(CH_2Cl)_2$ (1.0 mL). After stirring at room temperature for 18 h, the resulting mixture was added 2,3- dichloro-5,6-dicyano-1,4-benzoquinone (DDQ) (22.8 mg, 0.100 mmol). After stirring at room temperature for 15 min, the resulting mixture was concentrated and purified by silica gel PTLC (eluent: CH_2Cl_2), followed by gel permeation chromatography (GPC, eluent: CH_3Cl) to give **8** (8.9 mg, 0.0095 mmol, 19% yield).

Black solid; Mp >300 °C; ^{1}H NMR ($CDCl_3$, 400 MHz) δ 8.10 (d, J = 1.0 Hz, 2H), 7.86 (d, J = 1.2 Hz, 2H), 7.62 (d, J = 8.6 Hz, 2H), 7.53 (s, 2H), 7.43–7.33 (m, 4H), 7.22 (s, 2H), 3.92 (s, 6H), 3.50 (s, 6H), 1.53 (s, 18H); ^{13}C NMR ($CDCl_3$, 100 MHz) δ 167.8, 163.9, 151.8, 151.5, 141.2, 140.7, 140.6, 139.5, 137.9, 137.8, 136.2, 136.1, 135.2, 131.4, 130.5, 127.7, 127.6, 127.0, 126.4, 124.1, 120.2, 119.8, 52.7, 52.5, 35.2, 31.4; HRMS (ESI) calcd for $C_{58}H_{44}Cl_2NaO_8$ $[M+Na]^+$ 961.2311 found 961.2305 (−0.62 ppm).

Tetramethyl 2,2'-dichloro-5,5',13,13'-tetramethoxy-[8,8'-bibenzo[7,8]azuleno[6,5,4-jk]fluorene]-9,9',10,10'-tetracarboxylate (9)

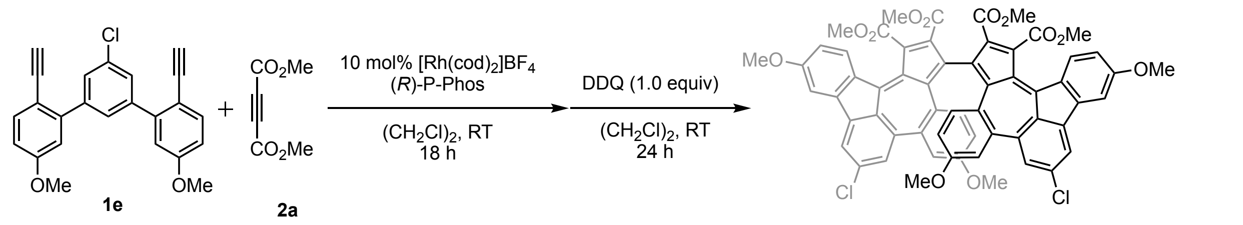

1e 2a 1.1 equiv 9

(*R*)-P-Phos (3.3 mg, 0.0051 mmol) and $[Rh(cod)_2]BF_4$ (2.0 mg, 0.0049 mmol) were dissolved in CH_2Cl_2 (1.0 mL), and the mixture was stirred at room temperature for 10 min. H_2 was introduced to the resulting solution in a Schlenk tube. After stirring at room temperature for 30 min, the resulting mixture was concentrated under reduced pressure. The residue was dissolved in $(CH_2Cl)_2$ (4.0 mL) and added to a solution of **1e** (18.5 mg, 0.0496 mmol) and **2a** (7.9 mg, 0.056 mmol) in $(CH_2Cl)_2$ (1.0 mL). After stirring at room temperature for 18 h, the resulting mixture was added 2,3- dichloro-5,6-

dicyano-1,4-benzoquinone (DDQ) (11.4 mg, 0.0501 mmol). After stirring at room temperature for 24 h, the resulting mixture was concentrated and purified by silica gel PTLC (eluent: CH_2Cl_2/EtOAc = 95:5) to give **9** (13.1 mg, 0.0128 mmol, 52% yield).

Black solid; Mp >300 °C; ^{1}H NMR ($CDCl_3$, 400 MHz) δ 7.72 (d, J = 8.5 Hz, 2H), 7.53 (d, J = 2.6 Hz, 4H), 7.10 (d, J = 2.2 Hz, 2H), 6.95 (d, J = 2.5 Hz, 2H), 6.75 (dd, J = 2.3, 8.5 Hz, 2H), 6.71 (d, J = 8.8 Hz, 2H), 6.04 (dd, J = 2.6, 8.8 Hz, 2H), 3.92 (s, 6H), 3.84 (s, 6H), 3.80 (s, 6H), 3.59 (s, 6H); ^{13}C NMR ($CDCl_3$, 100 MHz) δ 168.8, 164.8, 163.4, 158.9, 150.6, 143.4, 142.4, 141.0, 138.0, 136.7, 136.1, 134.80, 134.76, 134.3, 133.8, 133.3, 130.5, 129.5, 129.0, 126.9, 126.5, 119.6, 113.2, 112.9, 112.6, 107.1, 55.9, 55.1, 52.5, 52.2; HRMS (ESI) calcd for $C_{60}H_{40}Cl_2NaO_{12}$ $[M+Na]^+$ 1045.1795 found 1045.1789 (−0.57 ppm).

3. Experimental Mechanistic Studies (Figure 3)

3.1. Effect of Ligands on Regioselectivity Using 10 and 2a (Figure 3a)

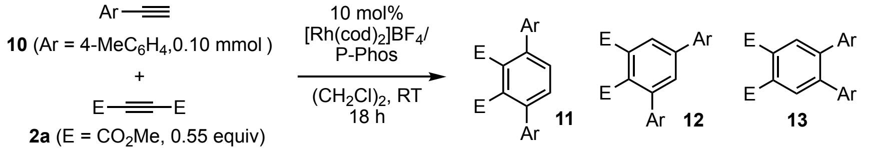

P-Phos (3.2 mg, 0.005 mmol) and [Rh(cod)$_2$]BF$_4$ (1.9 mg, 0.005 mmol) were dissolved in CH_2Cl_2 (1.0 mL), and the mixture was stirred at room temperature for 10 min. H_2 was introduced to the resulting solution in a Schlenk tube. After stirring at room temperature for 30 min, the resulting mixture was concentrated under reduced pressure. The residue was dissolved in $(CH_2Cl)_2$ (4.0 mL) and added to a solution of **10** (11.6 mg, 0.10 mmol) and **2a** (7.9 mg, 0.056 mmol) in $(CH_2Cl)_2$ (1.0 mL). After stirring at room temperature for 18 h, the resulting mixture was concentrated. The yields of **11**, **12**, and **13** were determined by the ^{1}H NMR analysis in $CDCl_3$ using tetrachloroethane as an internal standard, the resulting mixture was concentrated and purified by silica gel PTLC (eluent: *n*-hexane/ EtOAc = 4:1) to give mixture **11, 12, 13** (16.9, 0.0451mmol, 45% yield).

Yellow solid; ^{1}H NMR ($CDCl_3$, 400 MHz) 1H NMR (400 MHz, CDCl3) δ 8.18 (d, J = 1.8 Hz, 1H, **12**), 7.75 (s, 2H, **13**), 7.73 (d, J = 2.0 Hz, 1H, **12**), 7.53 (ddd, J = 1.8, 1.8, 8.1 Hz, 2H, **12**), 7.47 (s, 2H, **11**), 7.33–7.19 (m, 8H, **11**; 4H, **12**; 8H, **13**), 7.05–7.03 (m, 2H, **12**), 3.93 (s, 3H, **12**), 3.92 (s, 6H, **13**), 3.71 (s, 3H, **12**), 3.63 (s, 6H, **11**), 2.40 (s, 3H, **12**), 2.39 (s, 6H, **11**; 3H, **12**), 2.32 (s, 6H, **13**); ^{13}C NMR ($CDCl_3$, 100 MHz) δ169.5, 169.1, 168.1, 166.5, 143.5, 142.2, 141.3, 139.8, 138.4, 137.9, 137.7, 137.3, 137.0, 136.9, 136.6, 136.4, 133.2, 132.6, 132.1, 131.8, 131.4, 130.6, 129.9, 129.6, 129.3, 129.2, 129.0, 128.9, 128.6, 128.3, 127.20, 127.18 52.79, 52.77, 52.51, 52.46, 21.35, 21.30, 21.29 ; HRMS (ESI) calcd for $C_{24}H_{22}NaO_4$ [M+Na]$^+$ 397.1416 found 397.1434 (+4.53 ppm).

3.2. Effect of Ligands on Regioselectivity Using 1g and 2a (Figure 3b)

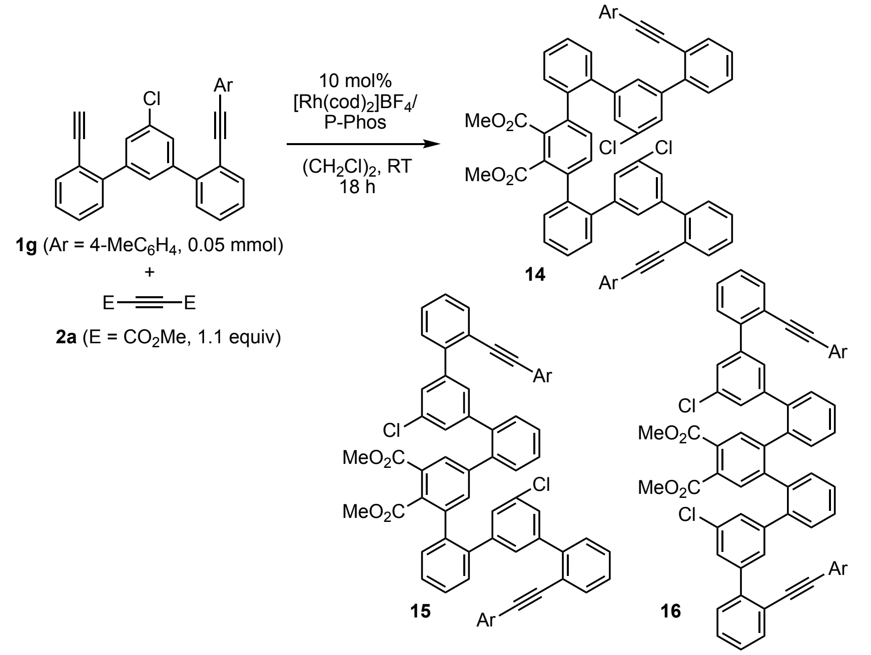

(*R*)-P-Phos (3.3 mg, 0.005 mmol) and [Rh(cod)$_2$]BF$_4$ (2.0 mg, 0.005 mmol) were dissolved in CH_2Cl_2 (1.0 mL), and the mixture was stirred at room temperature for 10 min. H_2 was introduced to the resulting solution in a Schlenk tube. After stirring at room temperature for 30 min, the resulting mixture was concentrated under reduced pressure. The residue was dissolved in $(CH_2Cl)_2$ (4.0 mL)

and added to a solution of **1g** (20.3 mg, 0.050 mmol) and **2a** (8.0 mg, 0.056 mmol) in $(CH_2Cl)_2$ (1.0 mL). After stirring at room temperature for 18 h, the resulting mixture was concentrated and purified by silica gel PTLC (1st PTLC eluent: hexane/ EtOAc = 2:1, 2nd PTLC eluent: hexane/ EtOAc = 2:1) to give **14, 15, 16** (20.7 mg, 0.022 mmol, 43 % yield).

Yellow solid; ^{1}H NMR ($CDCl_3$, 400 MHz) δ 7.77 (dd, J = 1.7, 1.7 Hz, 2H, **16**), 7.74 (s, 2H **16**) 7.70 (dd, J = 1.8, 1.8 Hz, 1H, **15**), 7.66 (dd, J = 1.8, 1.8 Hz, 1H, **15**), 7.63–7.54 (m, 6H, **14**; 2H, **15**), 7.49 (d, J =7.0 Hz, 4H, **14**), 7.45 (dd, J = 1.2, 7.6 Hz, 1H, **15**), 7.43–7.14 (m, 12H, **14**; 14H, **15**; 12H, **16**), 7.13–6.95 (m, 6H, **14**; 7H, **15**; 8H, **16**), 6.95–6.87 (m, 4H, **14**; 1H, **15**), 6.83 (dd, J = 1.0, 7.6 Hz, 1H, **15**), 6.71–6.64 (m, 2H, **16**), 6.57 (dd, J = 1.5, 1.5 Hz, 2H, **16**), 6.49 (dd, J = 1.7, 1.7 Hz, 2H, **16**), 6.26 (dd, J = 0.9, 7.6 Hz, 2H, **16**), 3.97 (s, 6H, **16**), 3.69 (s, 3H, **15**), 3.60 (s, 3H, **15**), 3.53 (s, 6H, **14**), 2.34 (s, 6H, **16**), 2.31 (s, 3H, **15**), 2.29 (s, 3H, **14**), 2.23 (s, 3H, **15**); ^{13}C NMR ($CDCl_3$, 100 MHz) δ 168.8, 167.7, 166.0, 144.2, 142.4, 142.2 142.12, 142.06, 142.0, 141.9, 141.81, 141.77, 141.74, 141.7, 141.5, 141.2, 140.0, 139.8, 139.6, 139.3, 139.1, 138.64, 138.62, 138.56, 138.54, 138.48, 138.4, 137.5, 137.4, 136.9, 133.6, 133.5, 133.4, 133.3, 133.2, 133.1, 133.0, 132.9, 132.8, 132.7, 132.5, 131.72, 131.67, 131.53, 131.46, 131.44, 131.41, 130.5, 130.4, 130.28, 130.25, 130.0, 129.9, 129.6, 129.5, 129.45, 129.38, 129.36, 129.3, 129.21, 129.16, 129.13, 128.8, 128.73, 128.71, 128.6, 128.4, 128.3, 128.1, 128.0, 127.9, 127.8, 127.68, 127.64, 121.77, 121.75, 121.69, 121.5, 120.21, 120.18, 120.17, 120.1, 93.3, 93.2, 88.39. 88.36, 88.30, 88.26, 52.9, 52.54, 52.48, 52.4, 21.7, 21.64, 21.62, 21.5 ; HRMS (ESI) calcd for $C_{64}H_{44}Cl_2NaO_4$ $[M+Na]^+$ 969.2514 found 969.2515 (+0.10 ppm).

4. X-Ray Crystallographic Analysis (Figure 6)

Single crystal X-ray diffraction data for **6ba** were collected using a XtaLAB Mini II diffractometer equipped with a Hybrid Pixel Array detector using graphite monochromatized Mo-Kα(0.71073 Å) radiation. The initial structure was solved by an intrinsic phasing method using SHELXT-2018/2[6] software and refined by a full matrix least-squares method using SHELXL-2018/3[7] software. All hydrogen atoms were located at geometrically calculated positions and included in least-squares calculations using riding models.

Data for **8** and **9** were collected using a Rigaku XtaLAB Synergy diffractometer equipped with a HyPix-6000HE Hybrid Photon Counting (HPC) detector with graphite-monochromatized Mo-Kα (0.71073 Å) radiation. The initial structure was solved by an intrinsic phasing method using SHELXT-2014/5[6] software and refined by a full matrix least-squares method using SHELXL-2019/3[7] software. All hydrogen atoms were located at geometrically calculated positions and included in least-squares calculations using riding models.

Details of the crystal data and the summaries of the intensity data collection parameters for **6aa**, **8**, and **9** are listed in Tables S1–S3. Single crystals suitable for X-ray analyses were grown by slow evaporation of a dichloromethane/*n*-hexane solution of **6aa**, vapor diffusion of a dichloromethane/*n*-hexane solution of **8** and a cyclopentylmethylether/*n*-hexane solution of **9**.

Crystallographic data have been, deposited with the Cambridge Crystallographic Data Centre: Deposition code CCDC 2417135 (**6aa**), CCDC 2417918 (**8**), and CCDC 2417919 [(±)-**9**]. They contain the supplementary crystallographic data for this paper. The data can be obtained free of charge from The Cambridge Crystallographic Data Centre via www.ccdc.cam.ac.uk/structures.

Table S1. Crystallographic data and structure refinement details for **6aa** (CCDC 2417135).

Empirical formula	$C_{29}H_{20}O_4$
Formula weight	432.45
Temperature/K	298.15
Crystal system	monoclinic
Space group	$P2_1/n$
a/Å	12.7501(3)
b/Å	7.7441(2)
c/Å	21.7364(5)
α /°	90
β /°	104.854(2)
γ /°	90
V/Å^3	2074.49(9)
Z	4
ρ_{calc}/g cm^{-3}	1.385
μ/mm^{-1}	0.092
F(000)	904.0
Crystal size/mm^3	0.35 × 0.22 × 0.1
Radiation	Mo Kα (λ = 0.71073)
2θ range for data collection/°	5.606 to 60.982
Index ranges	$-18 \leq h \leq 18$, $-11 \leq k \leq 11$, $-30 \leq l \leq 30$
Reflections collected	39823
Independent reflections	6189 [Rint = 0.0221, Rsigma = 0.0250]
Data/restraints/parameters	6189/0/301
Goodness-of-fit on F^2	1.059
Final R indexes [$I \geq 2\sigma(I)$]	$R_1 = 0.0569$, $wR_2 = 0.1368$
Final R indexes [all data]	$R_1 = 0.0857$, $wR_2 = 0.1504$
Largest diff. peak/hole / e Å^{-3}	0.30/-0.17

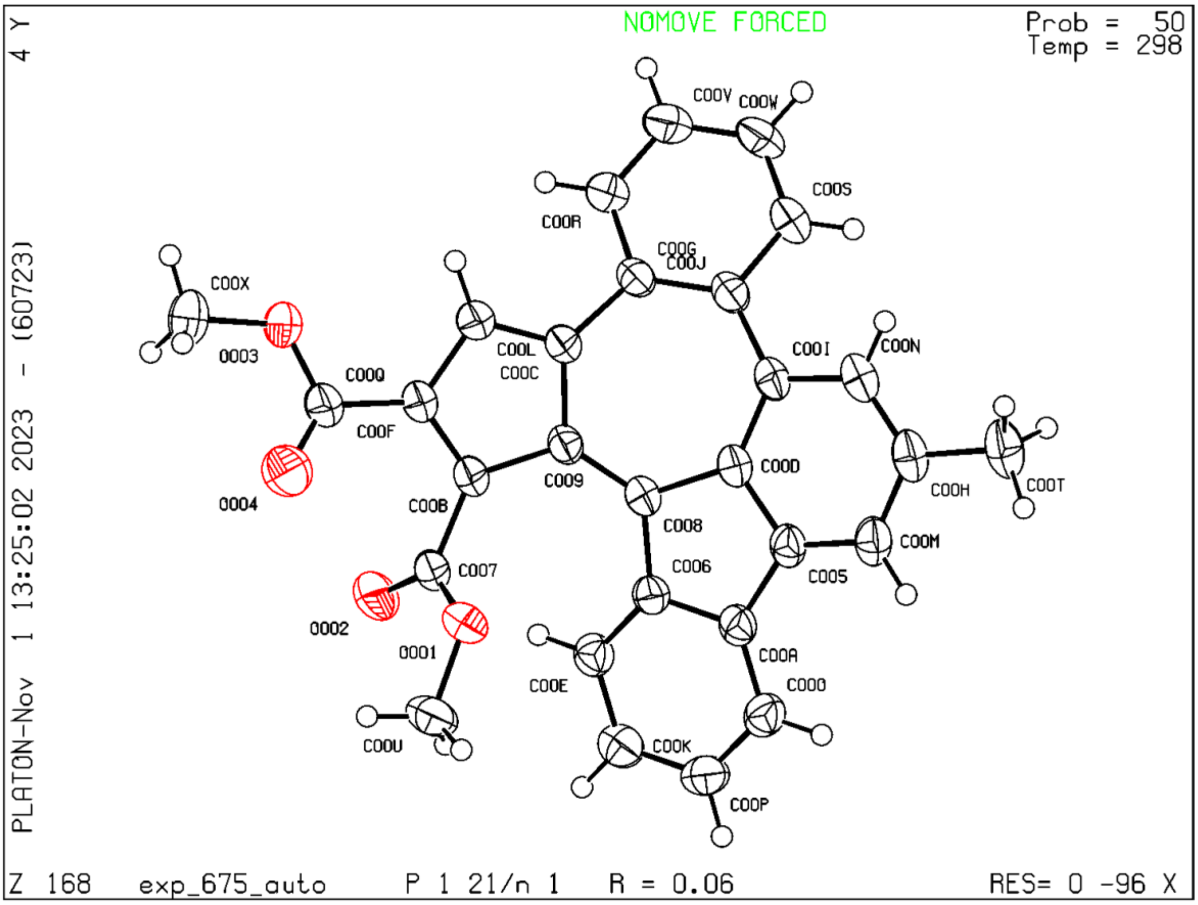

Figure S1. X-ray crystal structures of **6aa** showing thermal ellipsoids at the 50% probability level (oxygen: red).

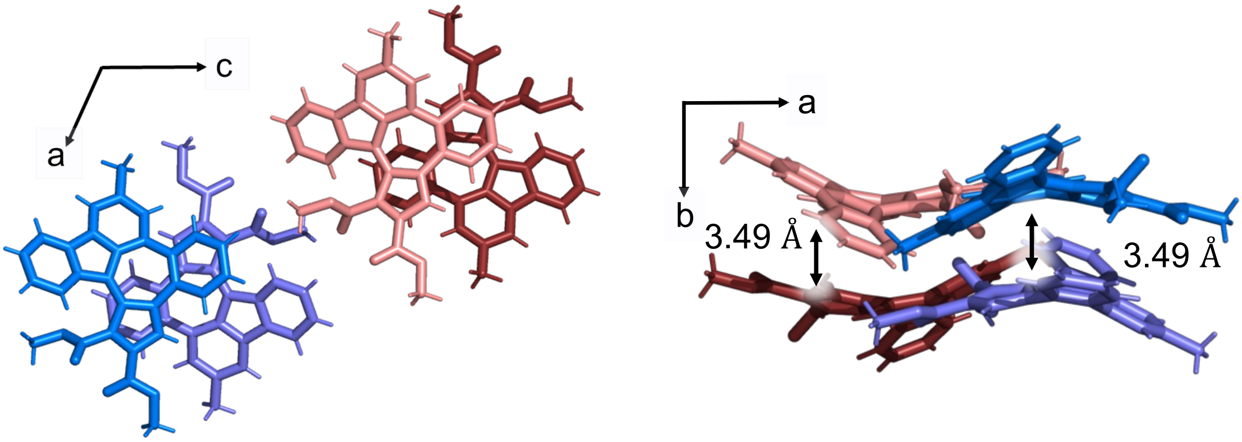

Figure S2. Packing structure of **6aa** in unit cell (left: top view, right: side view). The *P*-isomer and *M*-isomer are colored in blue and red, respectively. The interaction distances are shown.

Table S2. Crystallographic data and structure refinement details for **8** (CCDC 2417918).

Empirical formula	$C_{58}H_{44}Cl_2O_8/CH_2Cl_2$
Formula weight	962.02
Temperature/K	100.15
Crystal system	monoclinic
Space group	$P21/c$
a/Å	20.1823(6)
b/Å	28.3305(7)
c/Å	18.7832(4)
α /°	90
β /°	100.615(2)
γ /°	90
V/Å^3	10556.0(5)
Z	8
ρ_{calc}/g cm^{-3}	1.211
μ/mm^{-1}	0.202
F(000)	4008
Crystal size/mm^3	0.395 × 0.177 × 0.121
Radiation	Mo Kα (λ = 0.71073)
2θ range for data collection/°	3.960 to 52.744
Index ranges	$-25 \leq h \leq 25$, $-35 \leq k \leq 35$, $-23 \leq l \leq 23$
Reflections collected	179058
Independent reflections	21587 [Rint = 0.0627, Rsigma = 0.0314]
Data/restraints/parameters	21587/131/1370
Goodness-of-fit on F^2	1.017
Final R indexes [$I \geq 2\sigma(I)$]	R_1 = 0.0990, wR_2 = 0.2919
Final R indexes [all data]	R_1 = 0.1338, wR_2 = 0.3305
Largest diff. peak/hole / e Å^{-3}	1.409/-0.538

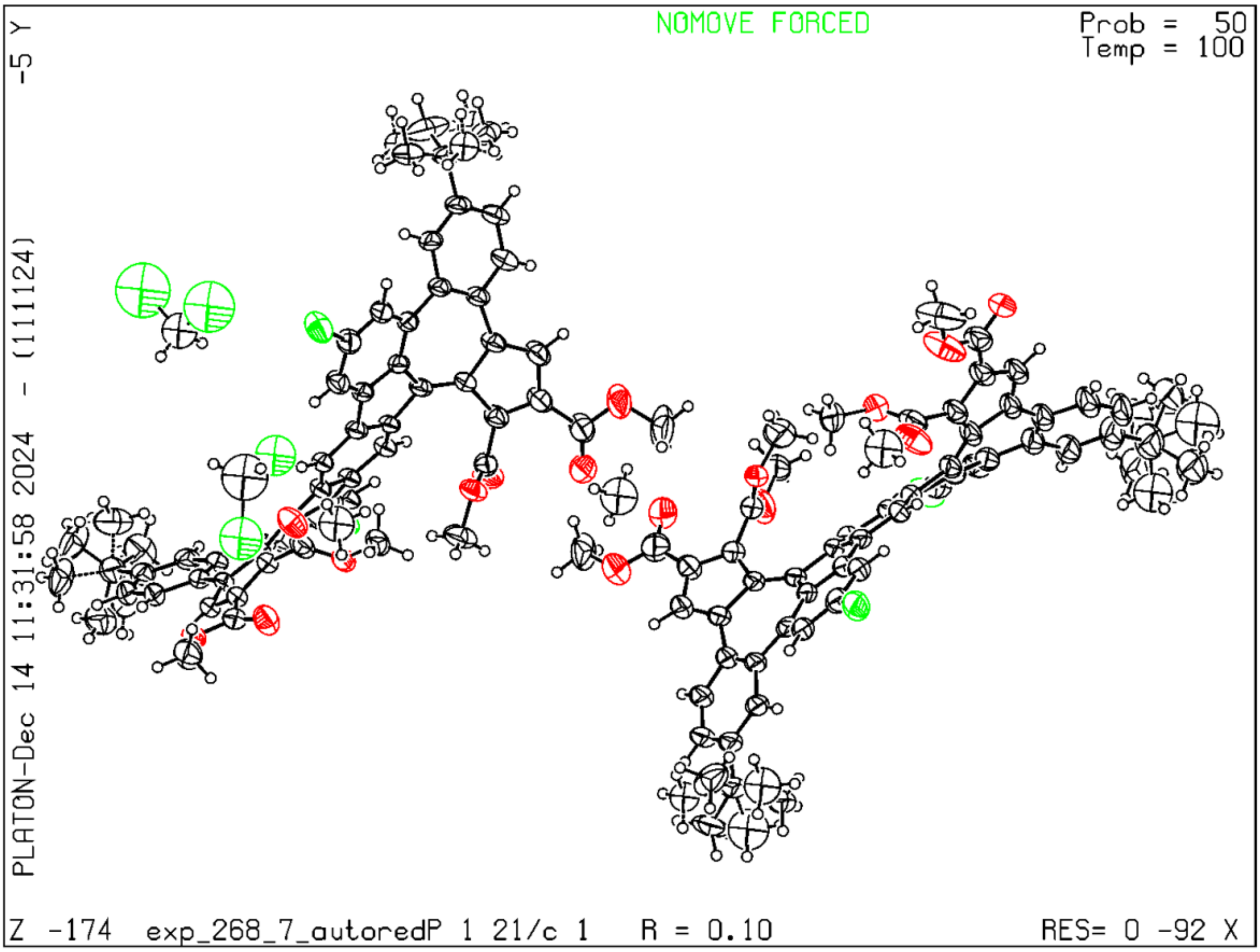

Figure S3. X-ray crystal structures of **8** showing thermal ellipsoids at the 50% probability level (oxygen: red, chlorine: green).

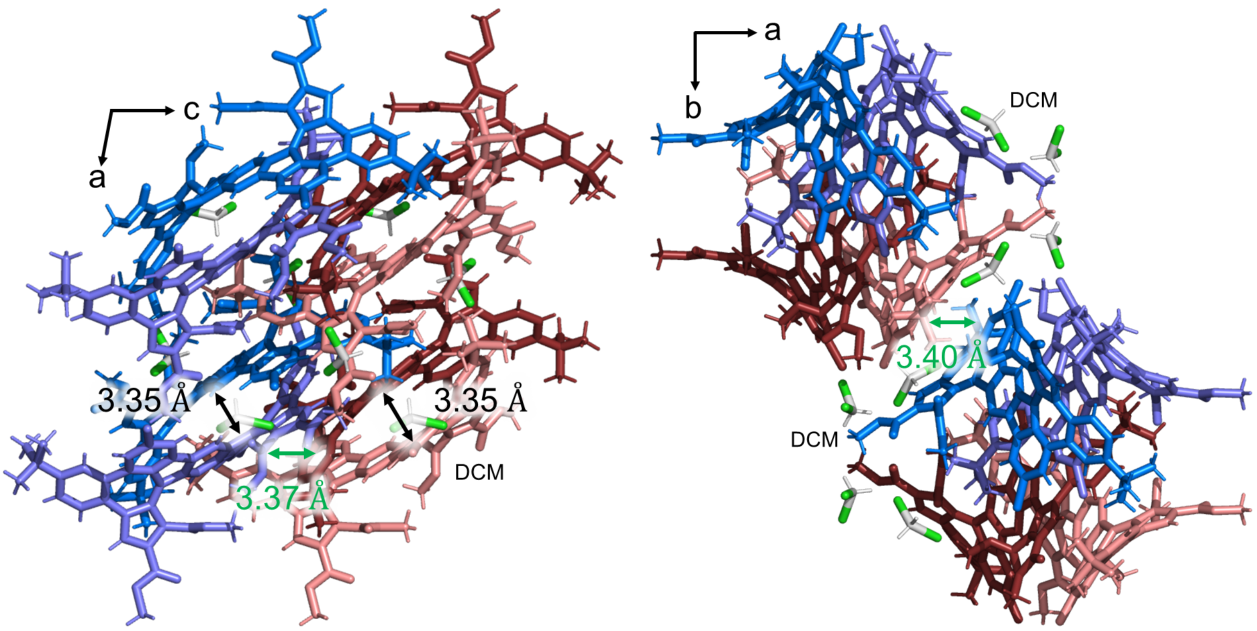

Figure S4. Packing structure of **8** in unit cell (left: top view, right: side view). The *P*-isomer and *M*-isomer are colored in blue and red, respectively. The interaction distances for the same enantiomer (black) and the opposite enantiomer (green) are shown. DCM = CH_2Cl_2.

Table S3. Crystallographic data and structure refinement details for (±)-**9** (CCDC 2417919).

Empirical formula	$C_{60}H_{40}Cl_2O_{12}/C_6H_{12}O$
Formula weight	1123.97
Temperature/K	100(2)
Crystal system	triclinic
Space group	*P*-1
a/Å	15.9299(2)
b/Å	18.3420(2)
c/Å	20.2036(3)
α /°	80.0930(11)
β /°	75.5130(13)
γ /°	84.2100(12)
V/Å^3	5619.93(13)
Z	4
ρ_{calc}/g cm^{-3}	1.328
μ/mm^{-1}	0.183
F(000)	2344
Crystal size/mm^3	0.378 × 0.160 × 0.057
Radiation	Mo Kα (λ = 0.71073)
2θ range for data collection/°	4.008 to 59.262
Index ranges	$-21 \leq h \leq 21$, $-25 \leq k \leq 25$, $-27 \leq l \leq 26$
Reflections collected	145745
Independent reflections	29125 [Rint = 0.0440, Rsigma = 0.0418]
Data/restraints/parameters	29125/284/1657
Goodness-of-fit on F^2	1.176
Final *R* indexes [$I \geq 2\sigma(I)$]	$R_1 = 0.0842$, $wR_2 = 0.1667$
Final *R* indexes [all data]	$R_1 = 0.1060$, $wR_2 = 0.1750$
Largest diff. peak/hole / e Å^{-3}	0.938/-0.778

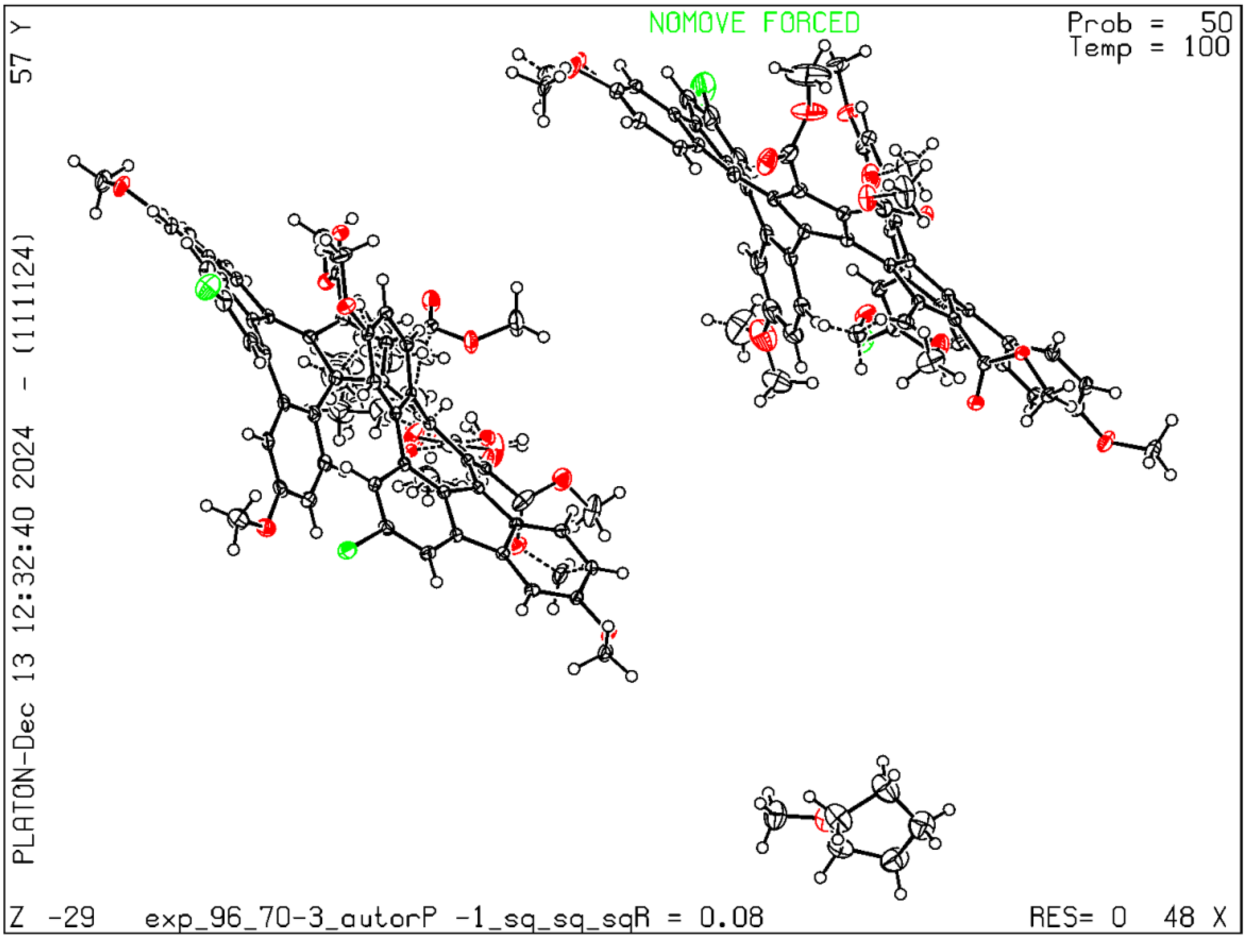

Figure S5. X-ray crystal structures of (±)-**9** showing thermal ellipsoids at the 50% probability level (oxygen: red, chlorine: green).

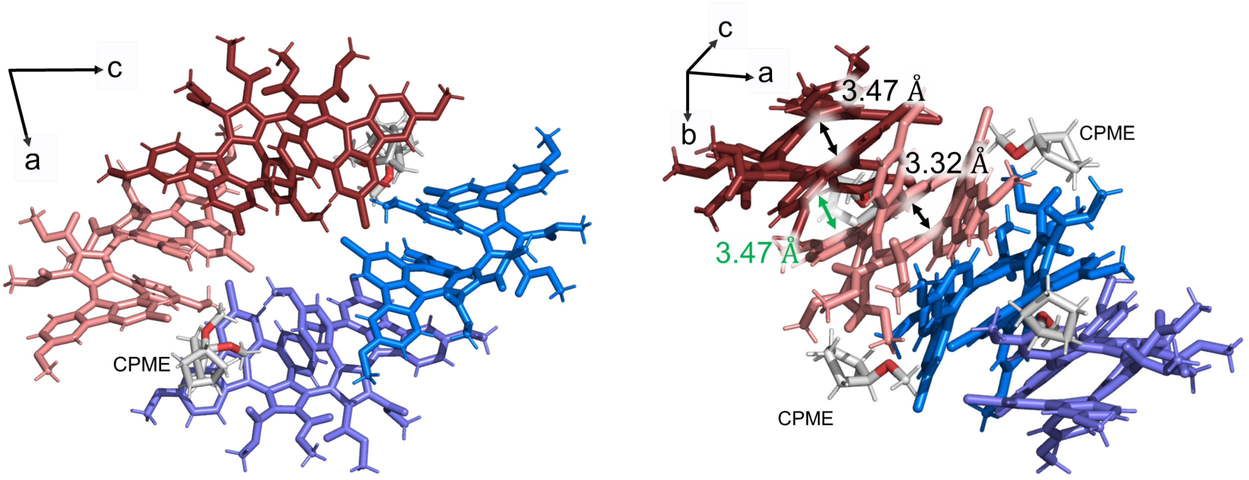

Figure S6. Packing structure of (±)-**9** in unit cell (left: top view, right: side view). The *P*-isomer and *M*-isomer are colored in blue and red, respectively. The intramolecular interaction distances (black) and the intermolecular interaction distances (green) are shown. CPME = cyclopentyl methyl ether.

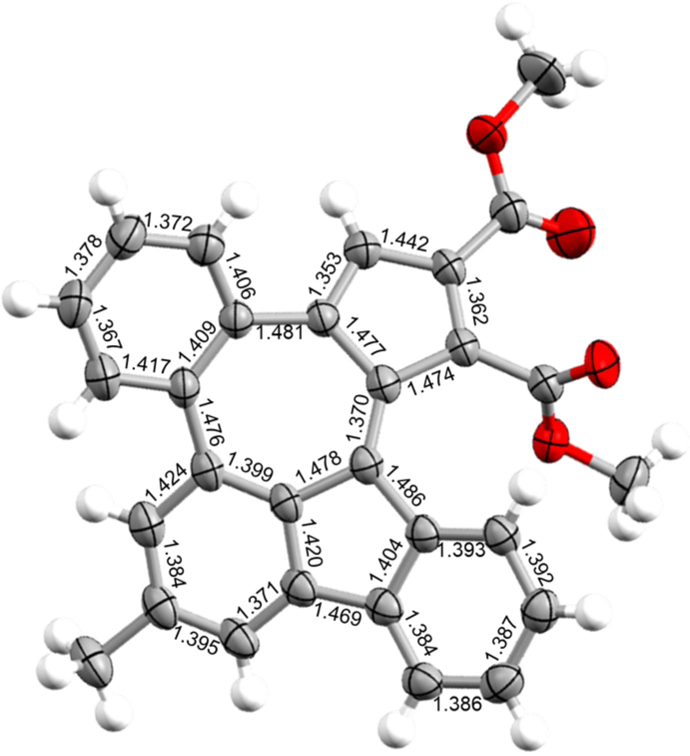

Figure S7. X-ray structure of **6aa** with aromatic carbon-carbon bond length (oxygen: red).

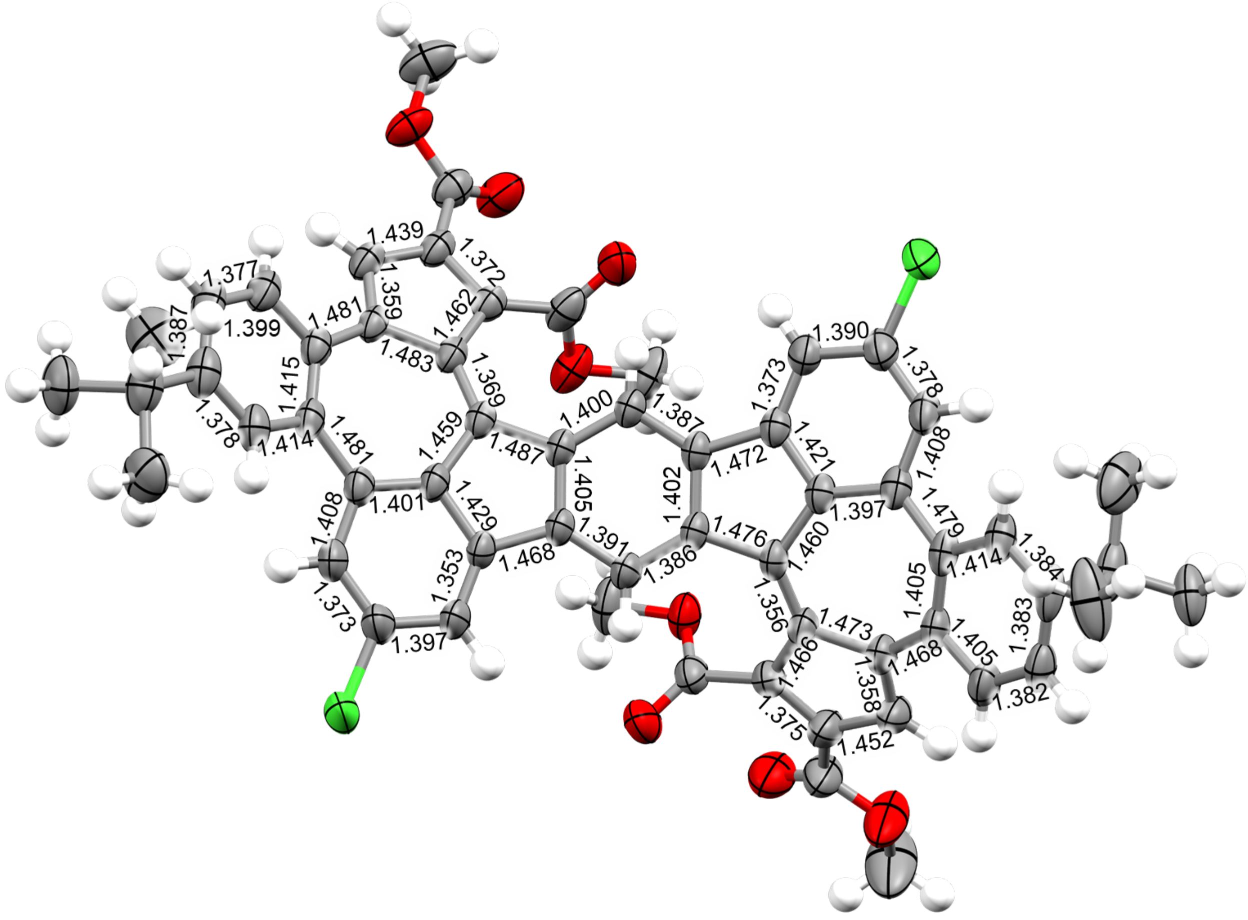

Figure S8. X-ray structure of **8** with aromatic carbon-carbon bond length (oxygen: red, chlorine: green).

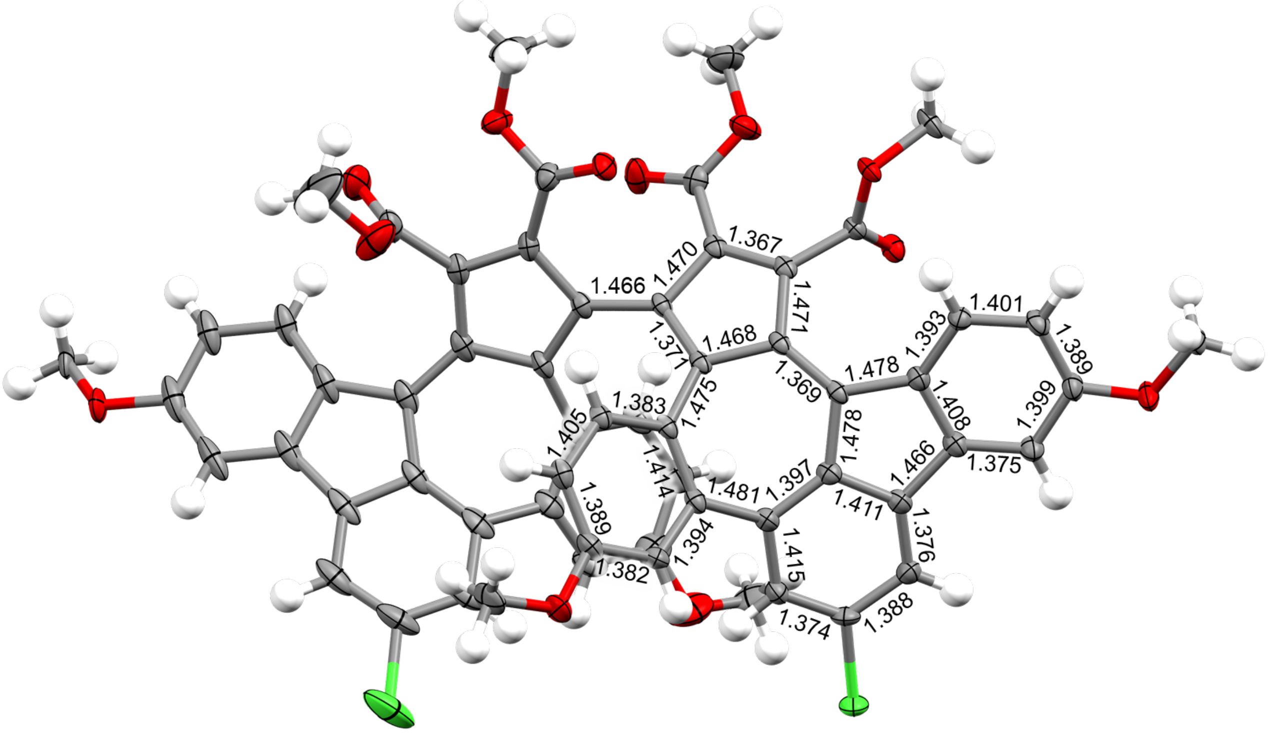

Figure S9. X-ray structure of (±)-**9** (front region) with aromatic carbon-carbon bond length (oxygen: red, chlorine: green).

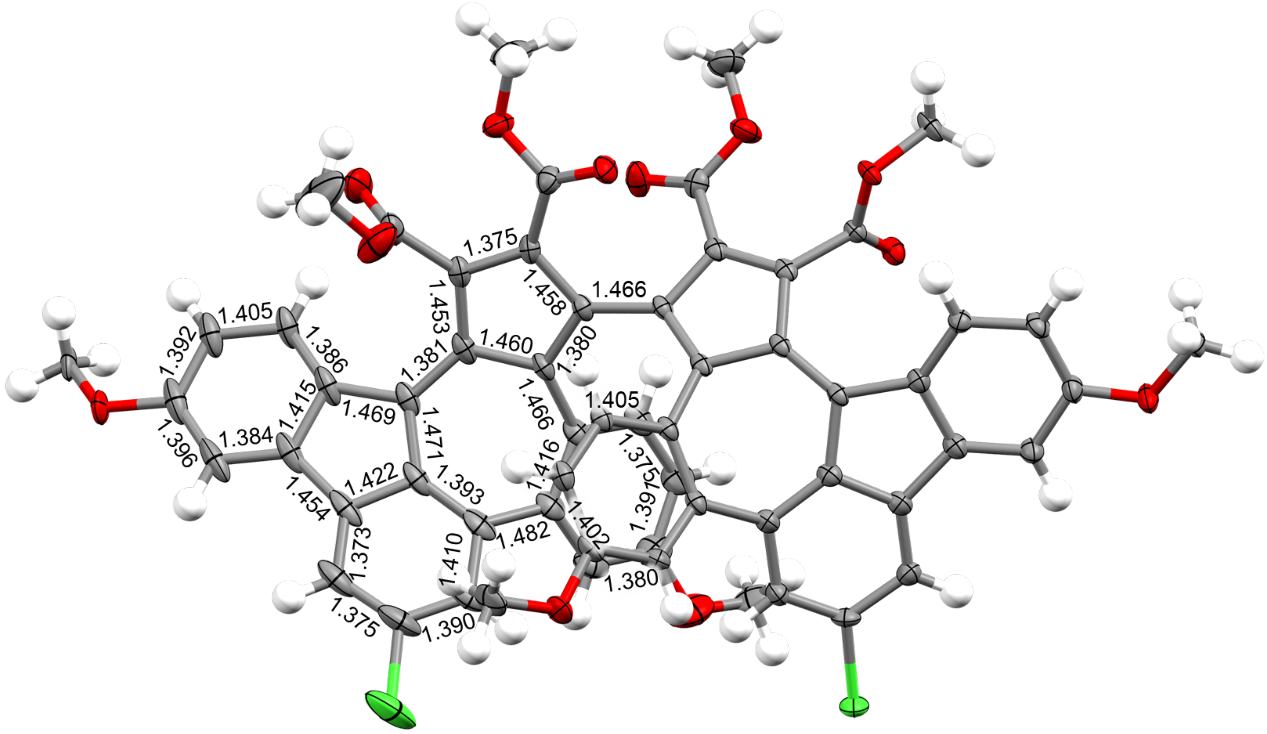

Figure S10. X-ray structure of (±)-**9** (back region) with aromatic carbon-carbon bond length (oxygen: red, chlorine: green).

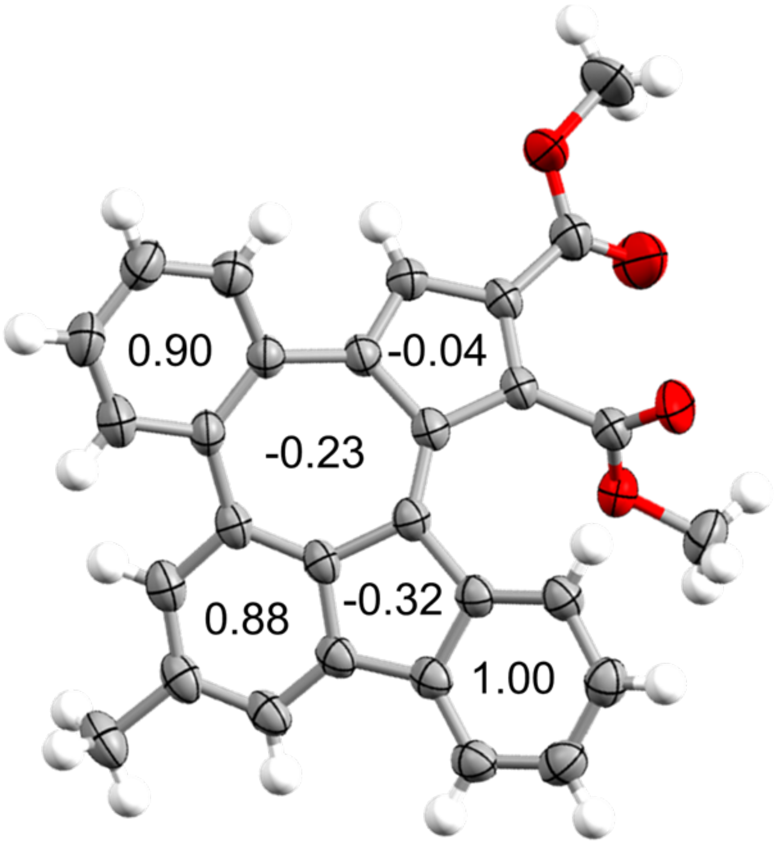

Figure S11.HOMA values of **6aa** calculated from X-ray bond length (oxygen: red).

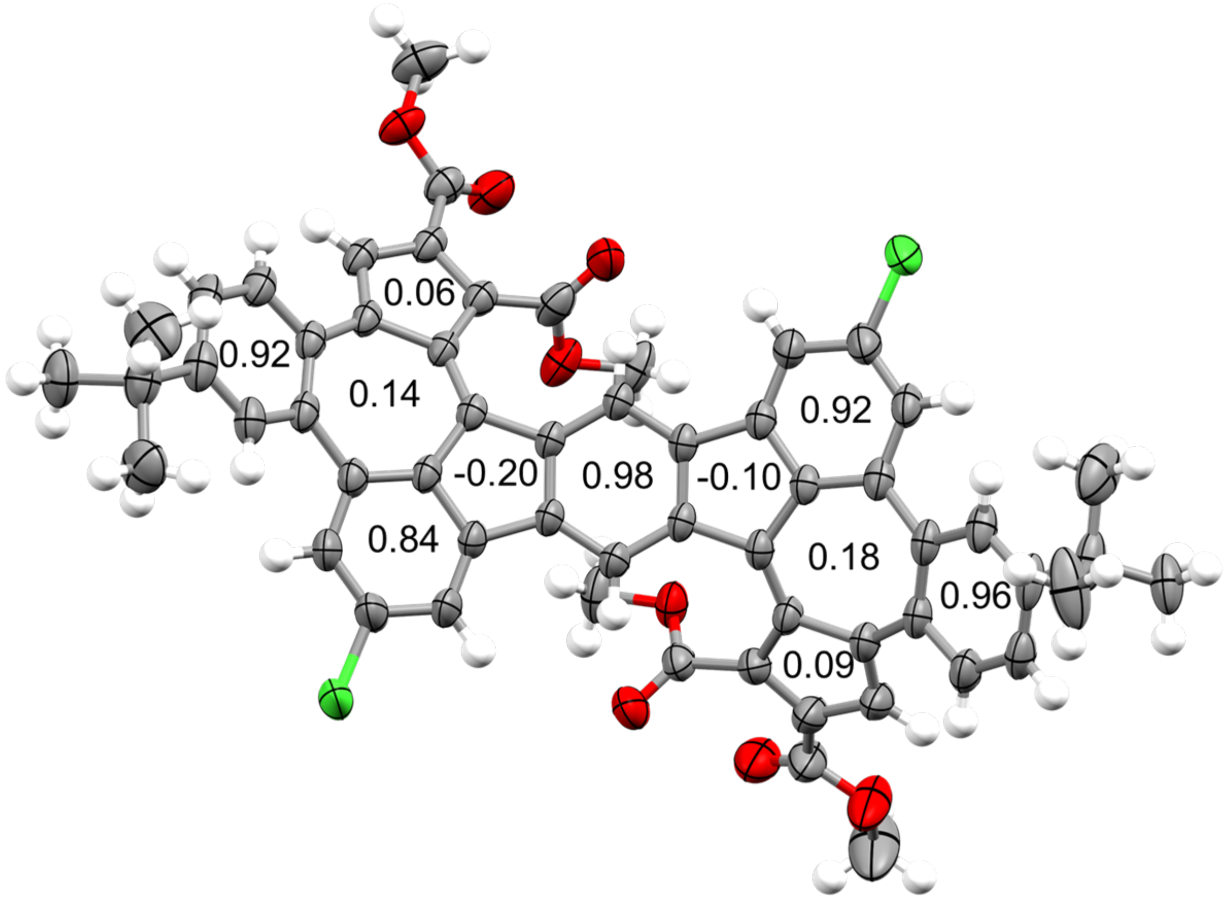

Figure S12. HOMA values of **8** calculated from X-ray bond length (oxygen: red, chlorine: green).

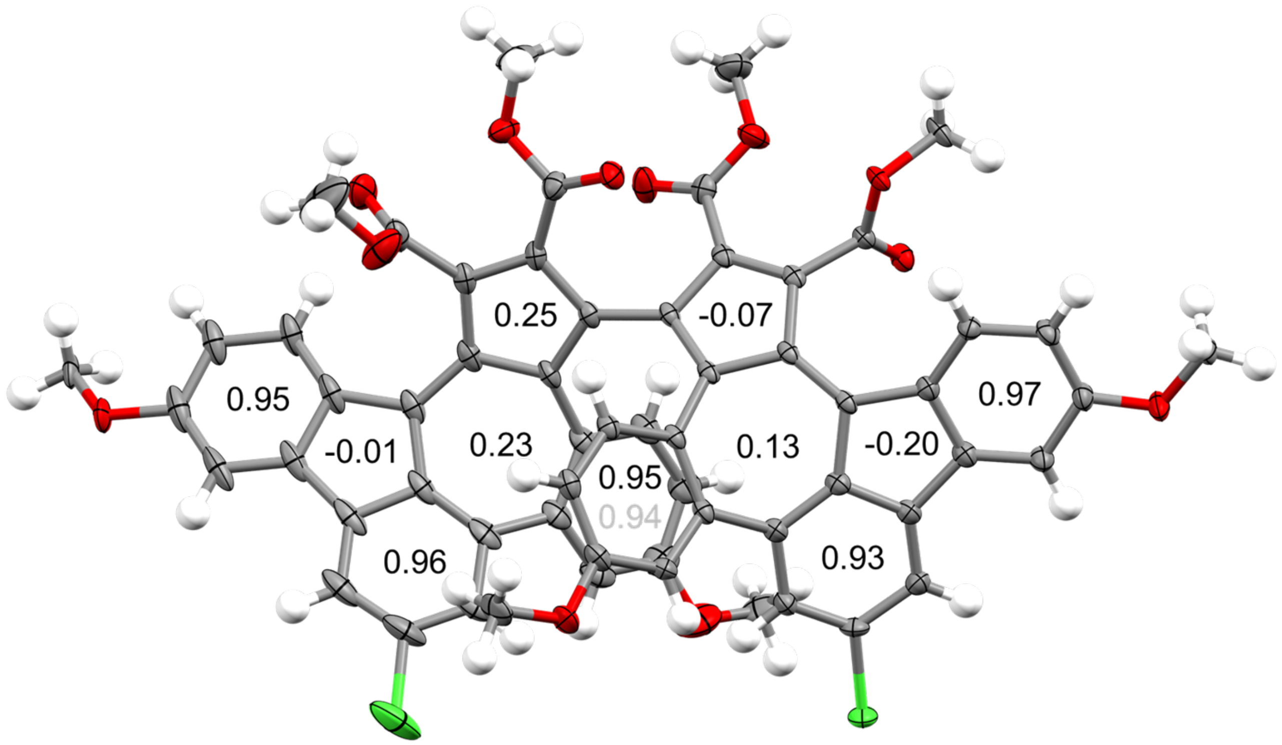

Figure S13. HOMA values of (±)-**9** calculated from X-ray bond length (oxygen: red, chlorine: green). Light text indicates the far side ring.

5. Self-Association of 6aa in Solution-State

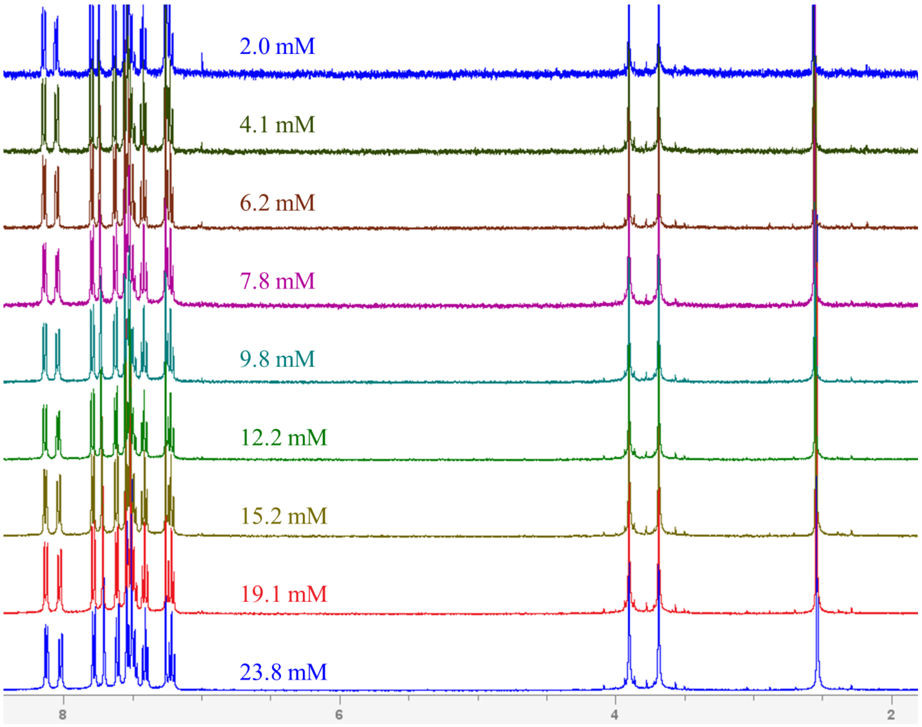

Figure S14. ^{1}H NMR spectra ($CDCl_3$, 400 MHz) of varying concentrations of **6aa.**

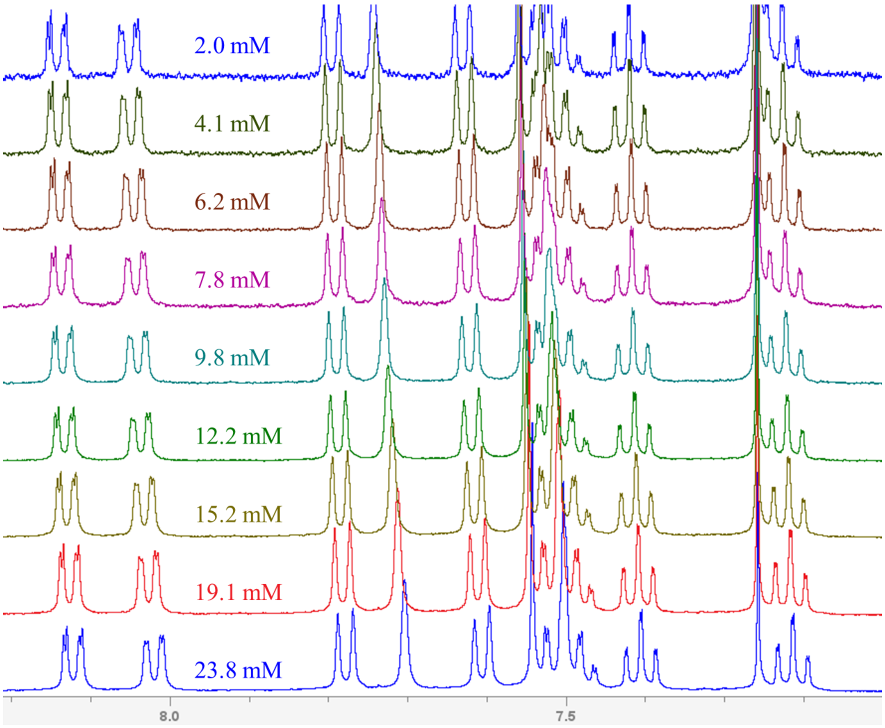

Figure S15. ^{1}H NMR spectra ($CDCl_3$, 400 MHz: aromatic region) of varying concentrations of **6aa**.

6. DFT and TD-DFT Calculations (Figures 5, 7, and 8b)

6.1. General

All calculations were carried out using the Gaussian 16 program.[8]

In structural analyses (section **6.2** and **6.5**), the hybrid density functional method based on B3LYP[9] with 6-31g(d)[10] basis set or UB3LYP with 6-31g(d) basis set were used for geometry optimizations. Compounds **7** and **9** were optimized with C_2 symmetry assumptions. Harmonic vibration frequency calculations at the same level were performed to verify all stationary points as local minima (with no imaginary frequency). Cartesian coordinates of optimized structures are listed in Tables S9–S13. Time-dependent (TD) DFT calculations were carried out at the B3LYP/6-31G(d) level of theory based on the optimized structure (Figures S16–S19). TD-DFT vertical one-electron excitations calculated for **6ba**, **6fa**, **7**, **9**, and **azulene** are summarized in Tables S4–S8 and Figures S20–S24. The nucleus-independent chemical shift (NICS)[11,12] values were calculated using NMR/GIAO keyword at B3LYP/6-311+G(2d,p) level (Figures S25–S28). The anisotropy of the induced current density (ACID)[13] plot based on the optimized structures was performed by using the AICD program provided by Herges at B3LYP/6-31+g(d,p) level (Figures S29–S32). To facilitate comparison with NICS, the molecules are inverted in the X−Y plane. Natural population analysis (NPA) charges and spin densities were analyzed with NBO 7.0 program.[14]

In theoretical mechanistic studies (section **6.3** and **6.4**), the hybrid density functional method based on m06[15] with 6-31g(d) basis set (LANL2DZ basis set for Rh) was used for geometry optimizations. Harmonic vibration frequency calculations at the same level were performed to verify the number of imaginary frequencies for all stationary points (0 for minima and 1 for TSs). The 6-311+G(d,p) basis set (SDD basis set for Rh) was used to calculate the single-point energies because it was envisaged that this strategy would provide greater accuracy with regard to the energetic information. The solvation effect was examined by performing single-point self-consistent reaction field (SCRF) calculations[16] based on the polarizable continuum model (PCM) for gas-phase optimized structures. Geometry optimization and vibrational analysis were performed at the same level. The intrinsic reaction coordinate (IRC) method was used to track minimum energy paths from transition structures to the corresponding local minima.[17] Qualitative noncovalent interactions (NCIs) were visualized with Multiwfn[18,19] and VMD[20] using the optimized electron density at the M06/6-311+G(d,p)&SDD(Rh) level of theory as the SCF energy correction. Natural bond orbital (NBO) and natural population analysis (NPA) charges were analyzed with NBO 7.0 program.

6.2. Structural Analyses (Figures 7 and 8b)

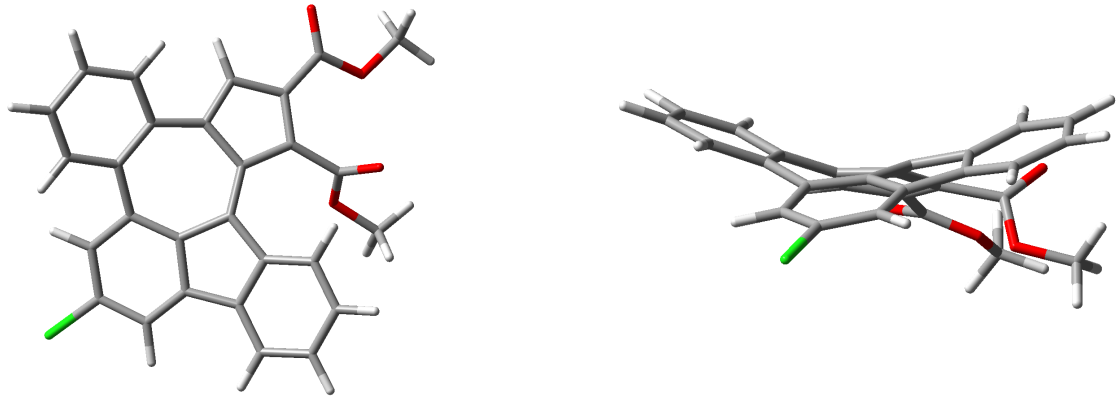

Figure S16. DFT optimized structures of **6ba** (left: top view, right: side view; oxygen: red, chlorine: green).

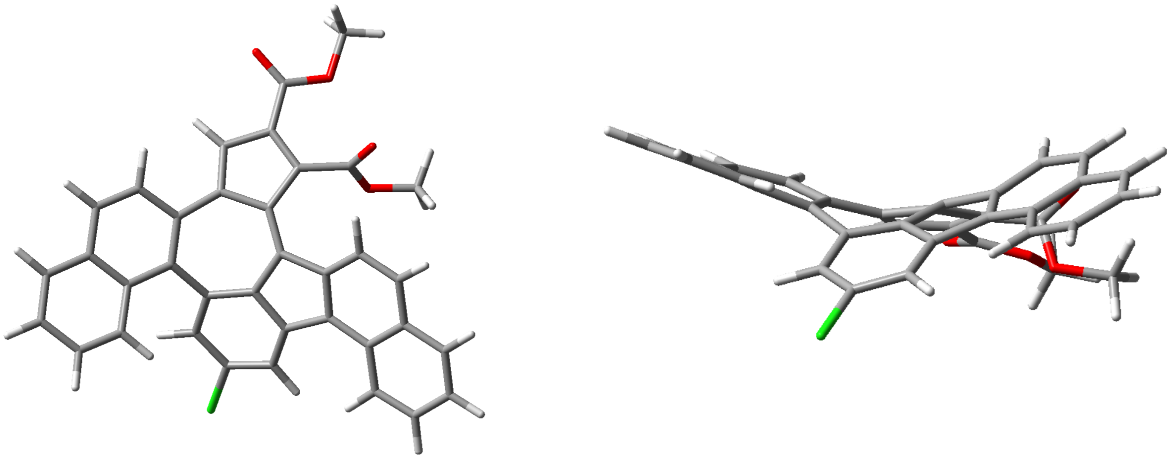

Figure S17. DFT optimized structures of **6fa** (left: top view, right: side view; oxygen: red, chlorine: green).

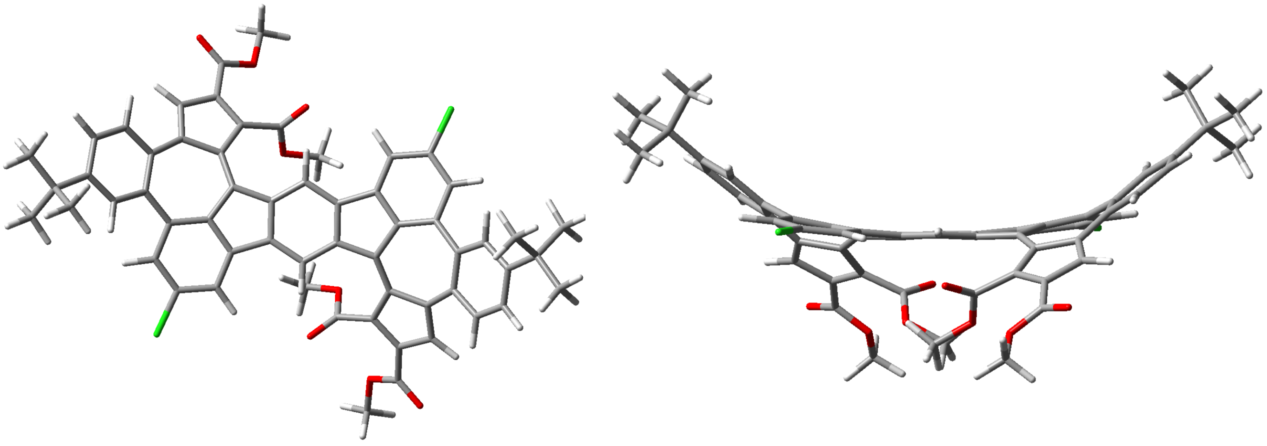

Figure S18. DFT optimized structures of **8** (left: top view, right: side view; oxygen: red, chlorine: green).

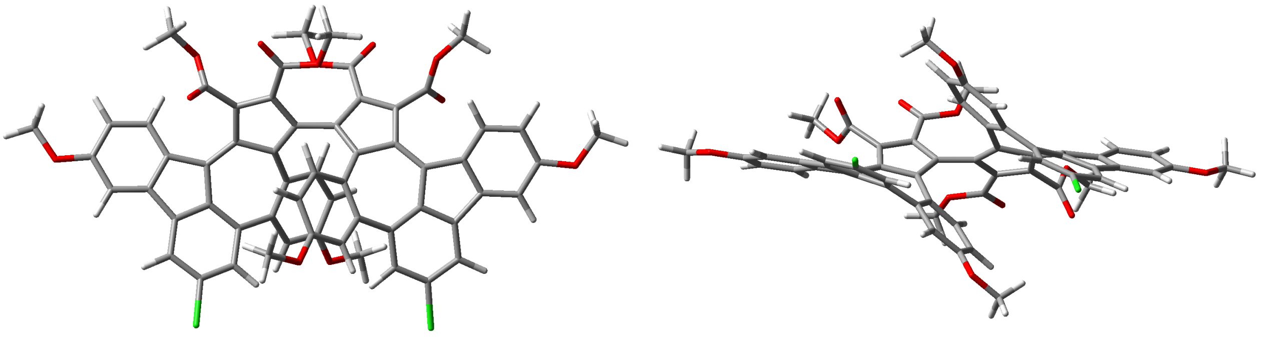

Figure S19. DFT optimized structures of **9** (left: top view, right: side view; oxygen: red, chlorine: green).

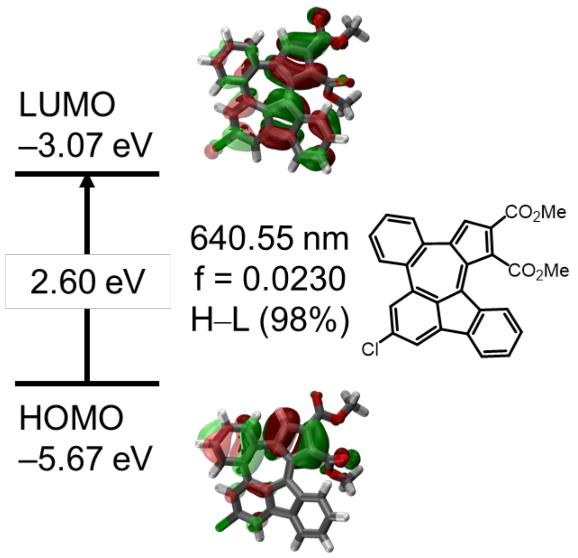

Figure S20. Molecular orbitals of **6ba** calculated by the DFT method at the B3LYP/6-31G(d) level of theory.

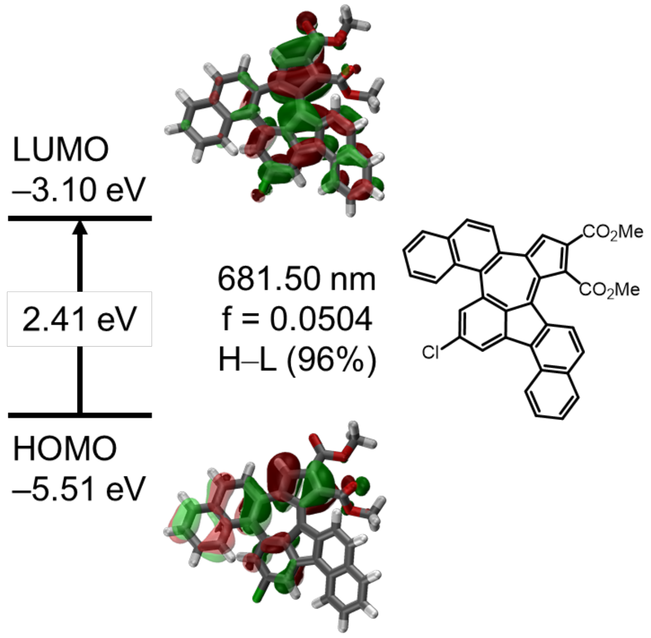

Figure S21. Molecular orbitals of **6fa** calculated by the DFT method at the B3LYP/6-31G(d) level of theory.

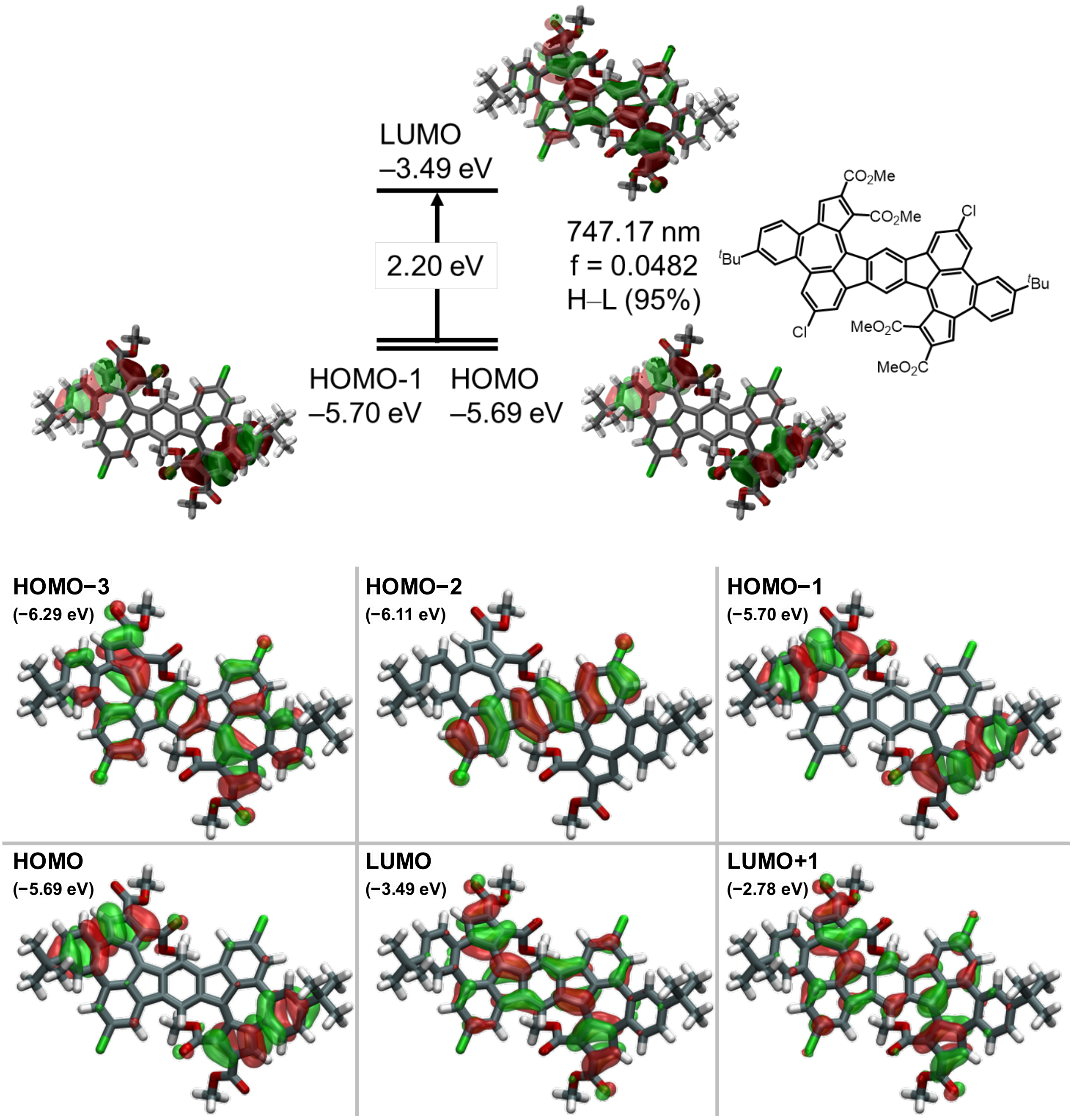

Figure S22. Molecular orbitals of **8** calculated by the DFT method at the B3LYP/6-31G(d) level of theory.

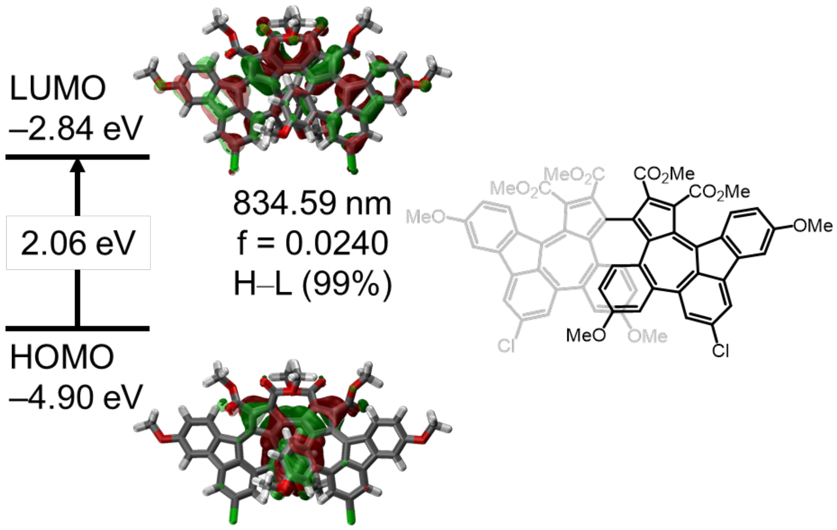

Figure S23. Molecular orbitals of **9** calculated by the DFT method at the B3LYP/6-31G(d) level of theory.

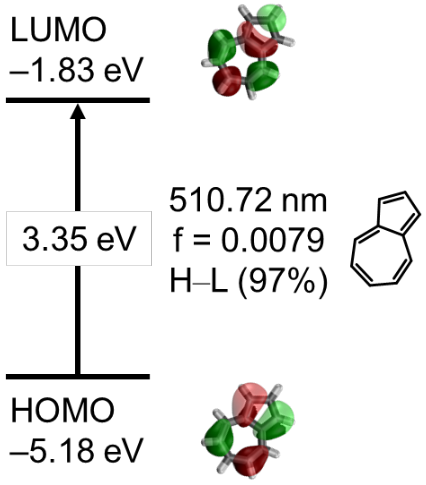

Figure S24. Molecular orbitals of **azulene** calculated by the DFT method at the B3LYP/6-31G(d) level of theory.

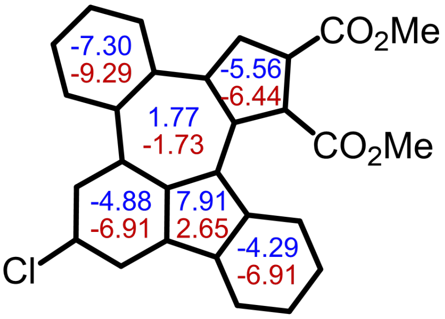

Figure S25. NICS(0) (blue) and NICS(1) (red) values (ppm) of **6ba** [B3LYP/6-311+G(2d,p) level of theory]. Dummy atoms are placed at the center of the benzene rings.

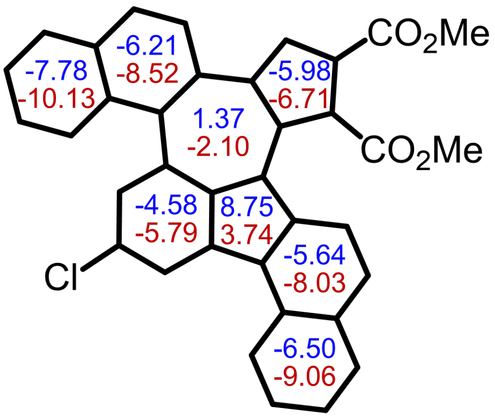

Figure S26. NICS(0) (blue) and NICS(1) (red) values (ppm) of **6fa** [B3LYP/6-311+G(2d,p) level of theory]. Dummy atoms are placed at the center of the benzene rings.

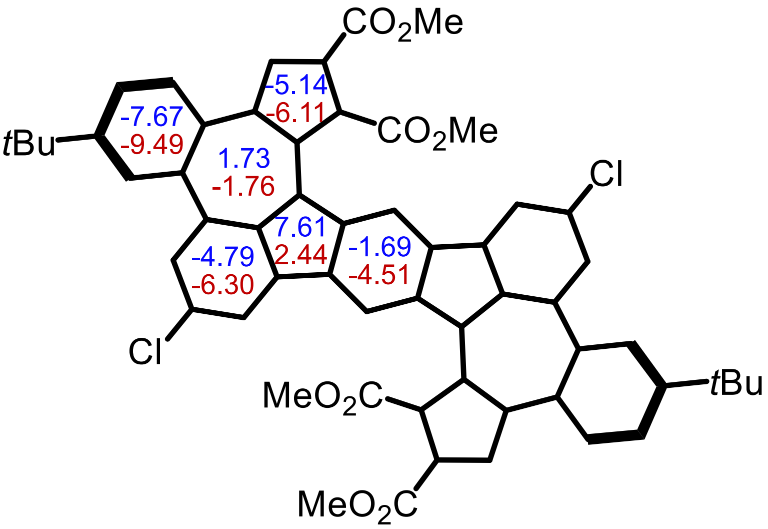

Figure S27. NICS(0) (blue) and NICS(1) (red) values (ppm) of **8** [B3LYP/6-311+G(2d,p) level of theory]. Dummy atoms are placed at the center of the benzene rings.

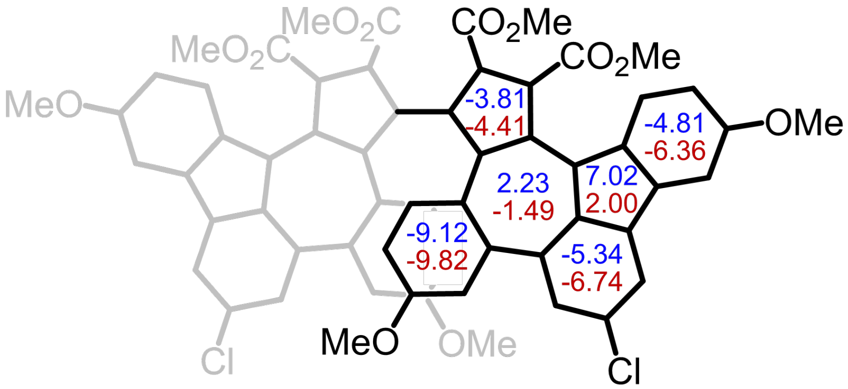

Figure S28. NICS(0) (blue) and NICS(1) (red) values (ppm) of **9** [B3LYP/6-311+G(2d,p) level of theory]. Dummy atoms are placed at the center of the benzene rings.

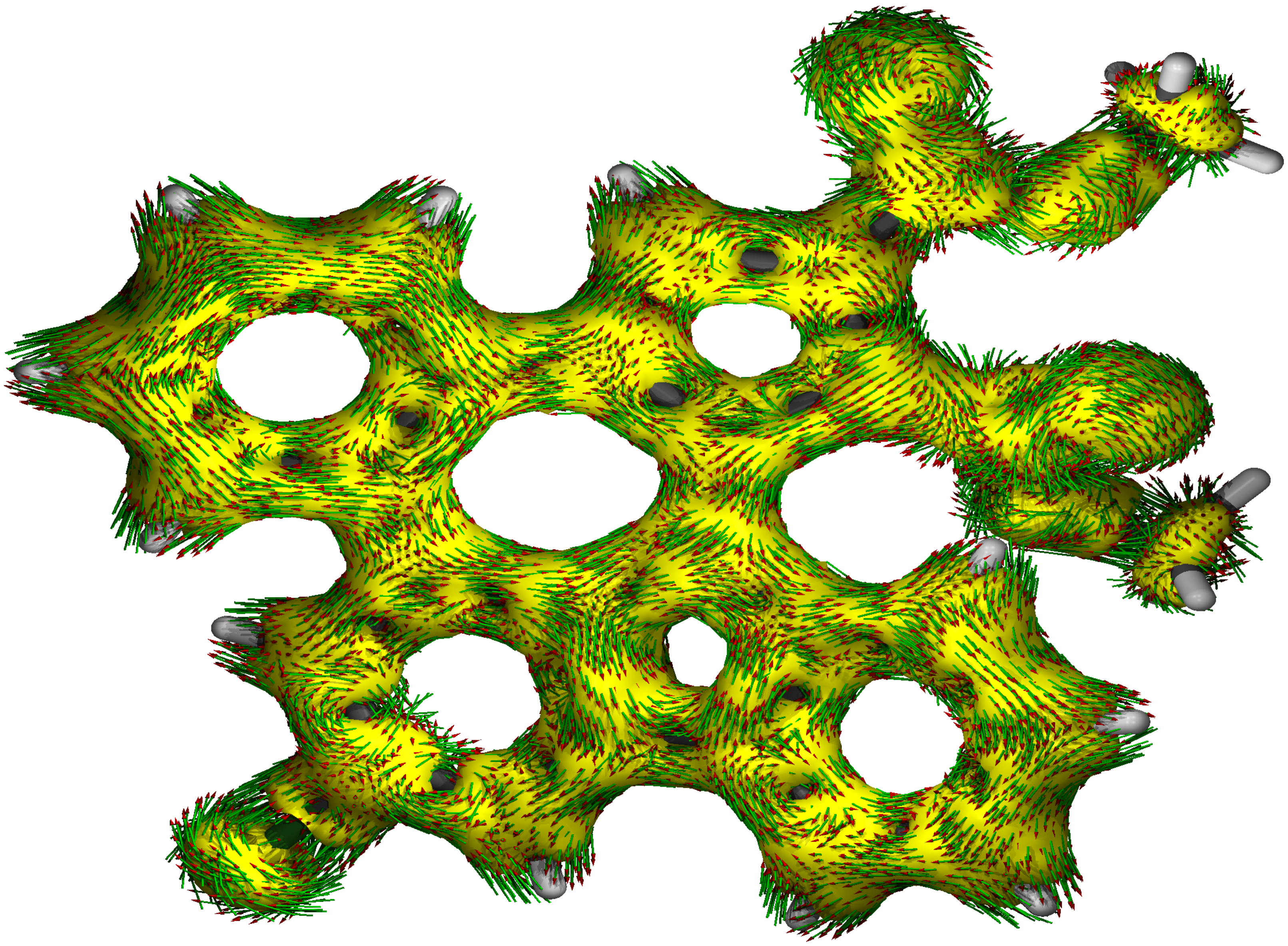

Figure S29. Anisotropy of the induced current density (ACID) plot of **6ba** based on all electrons with isovalue of 0.05 [B3LYP/6-31+g(d,p) level of theory]. The external magnetic field vector is perpendicular to the paper.

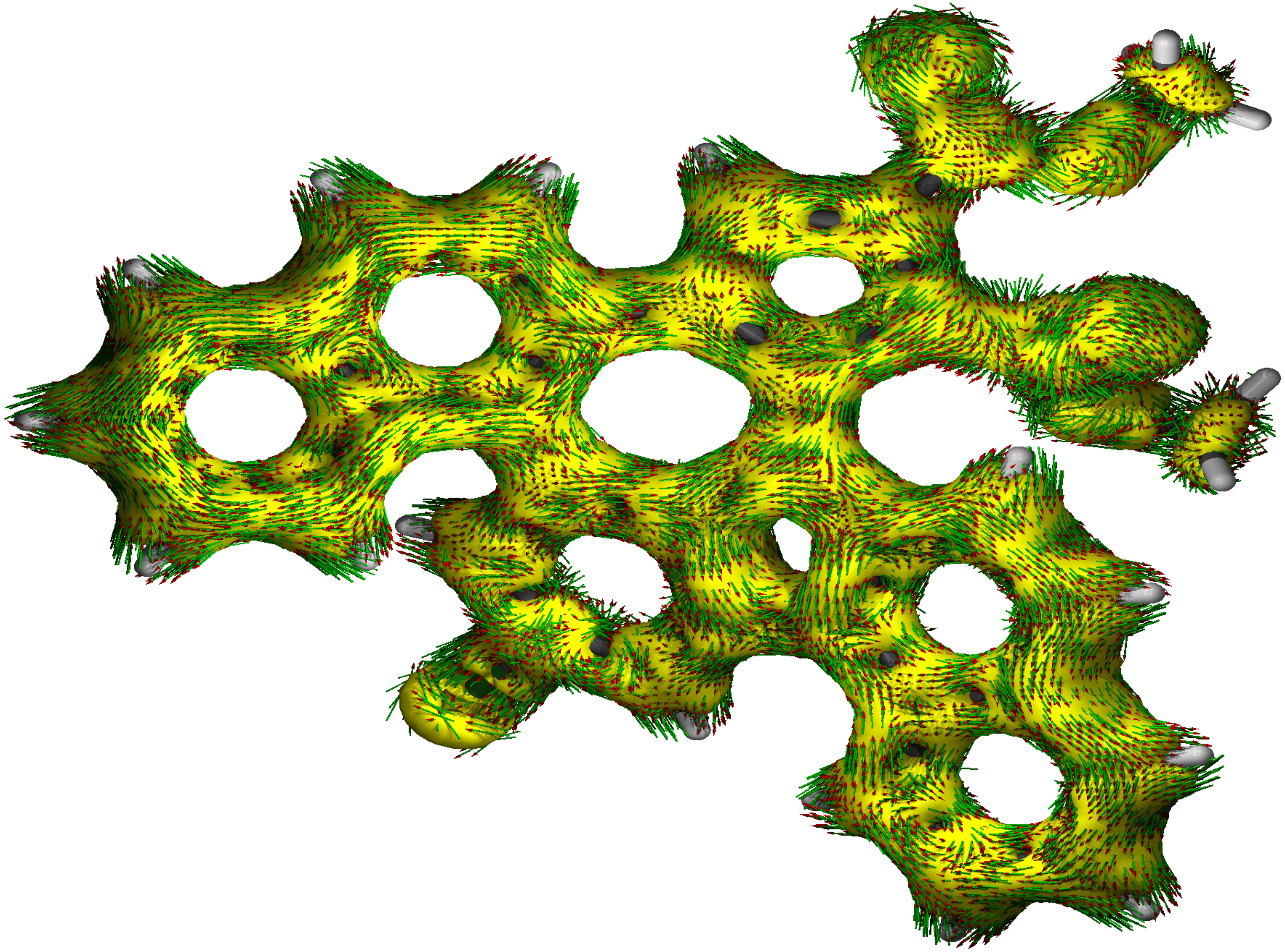

Figure S30. Anisotropy of the induced current density (ACID) plot of **6fa** based on all electrons with isovalue of 0.05 [B3LYP/6-31+g(d,p) level of theory]. The external magnetic field vector is perpendicular to the paper.

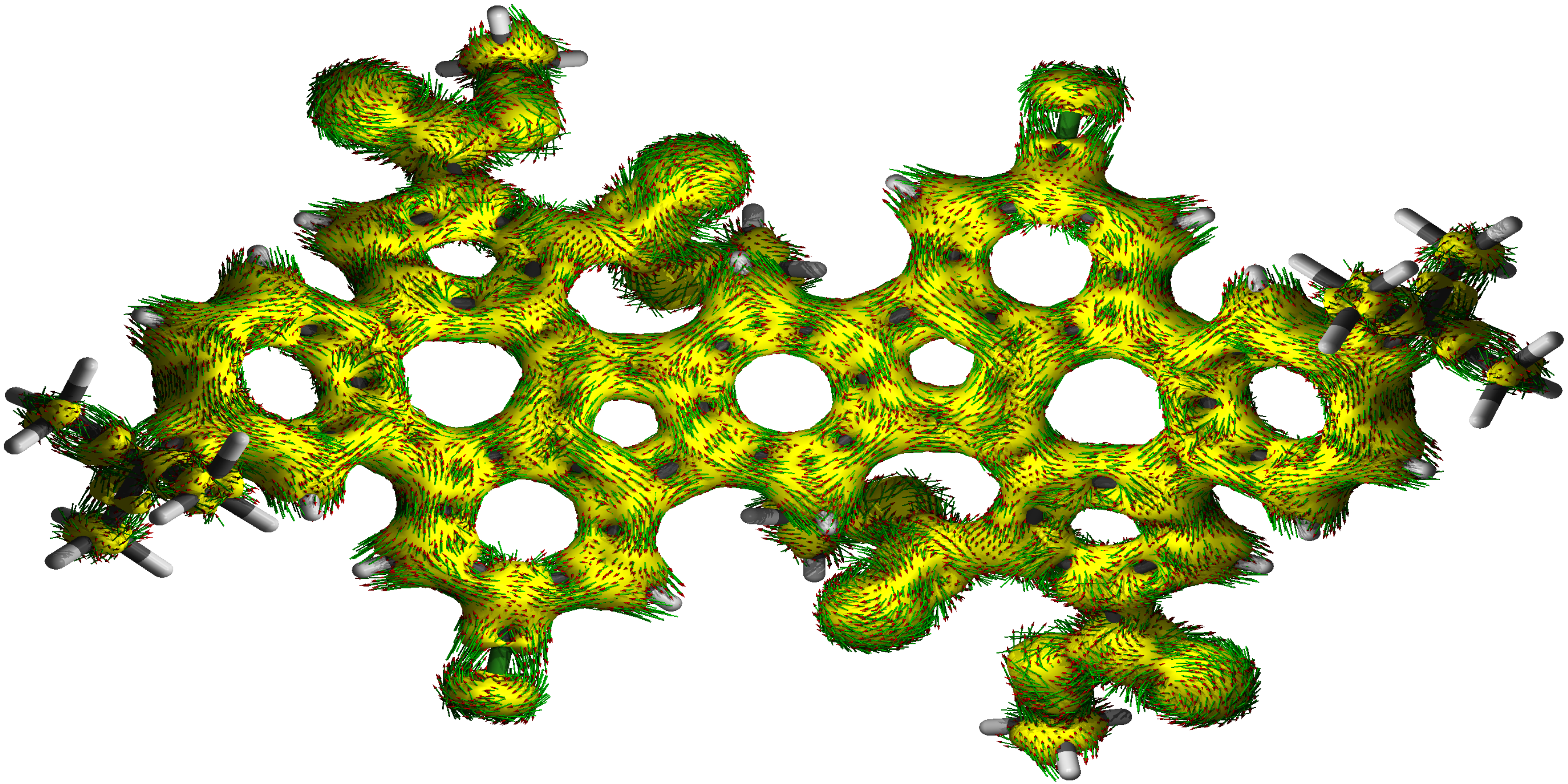

Figure S31. Anisotropy of the induced current density (ACID) plot of **8** based on all electrons with isovalue of 0.05 [B3LYP/6-31+g(d,p) level of theory]. The external magnetic field vector is perpendicular to the paper.

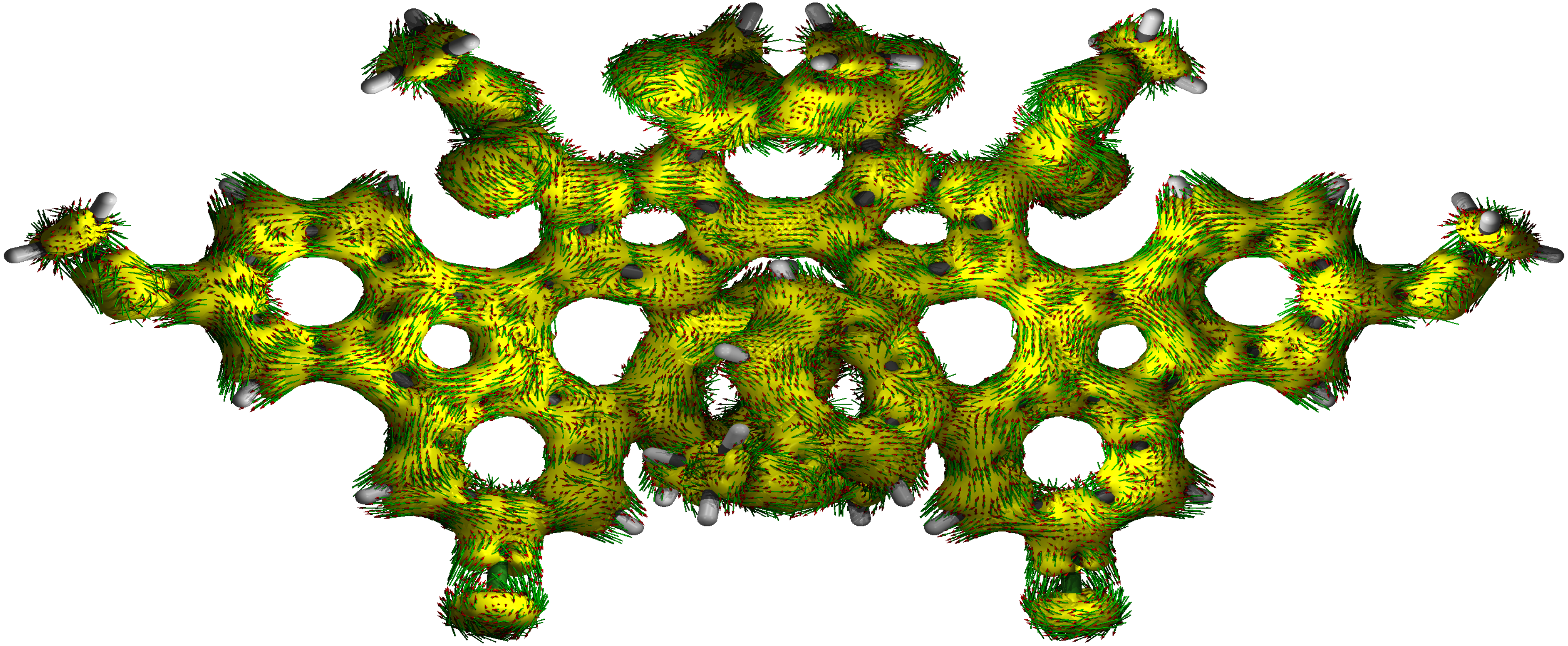

Figure S32. Anisotropy of the induced current density (ACID) plot of **9** based on all electrons with isovalue of 0.05 [B3LYP/6-31+g(d,p) level of theory]. The external magnetic field vector is perpendicular to the paper.

Table S4. TD-DFT vertical one-electron excitations calculated for **6ba**.

exited state	energy (eV)	wavelength (nm)	oscillator strength (*f*) [a]	description [b]
1	1.9356	640.55	0.0230	HOMO → LUMO (0.70270) (98.0%)
3	2.8212	439.48	0.1323	HOMO–3 → LUMO (–0.22162) (9.78%)<br>HOMO–2 → LUMO (-0.36630) (26.7%)<br>HOMO–1 → LUMO (0.52619) (55.1%)<br>HOMO → LUMO+1 (–0.15892) (5.03%)
4	3.2454	382.03	0.1608	HOMO–3 → LUMO (0.61530) (75.5%)<br>HOMO–1 → LUMO (0.17597) (6.18%)<br>HOMO → LUMO+1 (–0.23976) (11.5%)
6	3.5428	349.96	0.1703	HOMO–6 → LUMO (–0.17745) (6.28%)<br>HOMO–3 → LUMO (0.16503) (5.43%)<br>HOMO–1 → LUMO (0.17848) (6.35%)<br>HOMO → LUMO+1 (0.61634) (75.8%)
8	3.7337	332.07	0.0246	HOMO–6 → LUMO (0.49727) (49.6%)<br>HOMO–5 → LUMO (–0.40517) (32.9%)<br>HOMO–2 → LUMO+1 (0.17997) (6.50%)
10	4.0634	305.12	0.1047	HOMO–7 → LUMO (–0.18385) (6.78%)<br>HOMO–1 → LUMO+1 (0.61228) (75.2%)<br>HOMO–2 → LUMO+1 (–0.16288) (5.32%)
12	4.2829	289.49	0.2555	HOMO–1 → LUMO+1 (0.64337) (82.7%)
13	4.3545	284.73	0.1287	HOMO–3 → LUMO+1 (–0.24685) (12.2%)<br>HOMO–2 → LUMO+1 (0.57632) (66.5%)
14	4.4813	276.67	0.0205	HOMO–3 → LUMO+1 (–0.33807) (23.0%)<br>HOMO → LUMO+3 (0.40258) (32.6%)<br>HOMO–2 → LUMO+1 (–0.38452) (29.7%)
15	4.5390	273.15	0.0191	HOMO–10 → LUMO (0.21362) (9.18%)<br>HOMO–9 → LUMO (0.51547) (53.4%)<br>HOMO–8 → LUMO (0.17045) (5.84%)<br>HOMO → LUMO+3 (0.26538) (14.2%)<br>HOMO → LUMO+4 (0.23904) (11.5%)
16	4.5641	271.65	0.0129	HOMO–10 → LUMO (–0.22637) (10.3%)<br>HOMO–9 → LUMO (0.41554) (34.8%)<br>HOMO–8 → LUMO (–0.33394) (22.5%)<br>HOMO → LUMO+3 (–0.33428) (22.5%)
17	4.6471	266.80	0.1327	HOMO–11 → LUMO (0.26969) (14.6%)<br>HOMO–10 → LUMO (0.42381) (36.1%)<br>HOMO–3 → LUMO+1 (0.28131) (15.9%)<br>HOMO–1 → LUMO+2 (–0.15979) (5.13%)<br>HOMO → LUMO+4 (–0.23904) (15.3%)

18	4.6981	263.91	0.0590	HOMO–11 → LUMO (0.49931) (50.1%)<br>HOMO–3 → LUMO+1 (–0.17641) (6.25%)<br>HOMO–2 → LUMO+1 (–0.16375) (5.38%)<br>HOMO–1 → LUMO+2 (0.41070) (33.9%)
19	4.7503	261.01	0.1023	HOMO–10 → LUMO (–0.33046) (21.9%)<br>HOMO–3 → LUMO+1 (0.42036) (35.4%)<br>HOMO–2 → LUMO+1 (0.19966) (5.38%)<br>HOMO → LUMO+3 (0.28474) (16.3%)
20	4.8932	253.38	0.2814	HOMO–11 → LUMO (–0.27092) (14.7%)<br>HOMO–2 → LUMO+2 (–0.22658) (10.3%)<br>HOMO–1 → LUMO+2 (0.38287) (29.4%)<br>HOMO → LUMO+3 (–0.24518) (12.1%)
21	4.9773	249.10	0.2385	HOMO–2 → LUMO+2 (0.57078) (65.5%)<br>HOMO–1 → LUMO+2 (0.19164) (7.38%)<br>HOMO–1 → LUMO+4 (–0.16776) (5.66%)
22	5.0109	247.43	0.0470	HOMO–4 → LUMO+1 (0.46970) (44.3%)<br>HOMO–3 → LUMO+1 (0.19094) (7.33%)<br>HOMO–2 → LUMO+4 (0.17599) (6.23%)<br>HOMO → LUMO+3 (–0.16438) (5.43%)<br>HOMO → LUMO+5 (–0.24248) (11.8%)
24	5.0910	243.54	0.0194	HOMO–13 → LUMO (0.19558) (7.70%)<br>HOMO–12 → LUMO (0.60792) (74.4%)<br>HOMO → LUMO+5 (0.22874) (10.5%)
25	5.1570	240.42	0.0146	HOMO–13 → LUMO (–0.17129) (5.91%)<br>HOMO–6 → LUMO+1 (–0.24680) (12.3%)<br>HOMO–5 → LUMO+1 (0.30916) (19.2%)<br>HOMO–2 → LUMO+4 (0.21869) (9.63%)<br>HOMO → LUMO+6 (0.36785) (27.3%)
26	5.1691	239.86	0.1129	HOMO–13 → LUMO (0.17454) (6.14%)<br>HOMO–6 → LUMO+1 (–0.16372) (5.40%)<br>HOMO–5 → LUMO+1 (0.16326) (5.37%)<br>HOMO–1 → LUMO+3 (–0.17362) (6.07%)<br>HOMO–1→ LUMO+4 (0.54733) (60.3%)
27	5.1978	238.53	0.0157	HOMO–13 → LUMO (0.51695) (53.8%)<br>HOMO–12 → LUMO (–0.20767) (8.69%)<br>HOMO–1 → LUMO+3 (0.29897) (18.0%)<br>HOMO → LUMO+6 (0.15901) (5.09%)
28	5.2540	235.98	0.0703	HOMO–13 → LUMO (–0.22283) (10.0%)<br>HOMO–6 → LUMO+1 (0.18886) (7.19%)<br>HOMO–5 → LUMO+1 (–0.24016) (11.6%)<br>HOMO–4 → LUMO+1 (–0.18757) (7.09%)<br>HOMO–3→ LUMO+2 (0.23352) (11.0%)<br>HOMO–1 → LUMO+3 (0.34264) (23.7%)<br>HOMO–1→ LUMO+4 (0.24735) (12.3%)

[a] Excitation energies with oscillator strength larger than 0.01 are listed. [b] Relative contribution larger than 5% is listed.

Table S5. TD-DFT vertical one-electron excitations calculated for **6fa**.

exited state	energy (eV)	wavelength (nm)	oscillator strength (*f*) [a]	description [b]
1	1.8193	681.50	0.0504	HOMO → LUMO (0.69511) (96.0%)
3	2.4737	501.21	0.0325	HOMO–2 → LUMO (0.68280) (93.3%)
4	2.9365	422.22	0.1925	HOMO–3 → LUMO (0.59254) (70.1%)<br>HOMO–2 → LUMO (–0.34777) (24.1%)
5	3.2191	385.16	0.0266	HOMO–4 → LUMO (0.67052) (70.1%)<br>HOMO–1 → LUMO+1 (0.18028) (24.1%)
6	3.3242	372.97	0.2213	HOMO–5 → LUMO (0.23842) (11.3%)<br>HOMO–3 → LUMO (0.27545) (15.1%)<br>HOMO → LUMO+1 (0.55061) (60.5%)<br>HOMO → LUMO+2 (0.17522) (6.13%)
7	3.3615	368.83	0.0380	HOMO–5 → LUMO (0.64448) (83.2%)<br>HOMO → LUMO+1 (–0.17276) (5.98%)
9	3.5692	347.37	0.0225	HOMO–6 → LUMO (0.58476) (68.7%)<br>HOMO–1 → LUMO+1 (–0.33786) (22.9%)
10	3.7185	333.43	0.2091	HOMO–8 → LUMO (0.20821) (8.69%)<br>HOMO–6 → LUMO (–0.24520) (12.1%)<br>HOMO–2 → LUMO+1 (0.22159) (9.84%)<br>HOMO–1 → LUMO+1 (–0.29277) (17.2%)<br>HOMO→ LUMO+2 (0.46274) (42.9%)
11	3.7349	331.97	0.0450	HOMO–8 → LUMO (0.54911) (60.5%)<br>HOMO → LUMO+2 (–0.36737) (27.1%)
12	3.7931	326.87	0.1182	HOMO–8 → LUMO (0.33893) (23.0%)<br>HOMO–6 → LUMO (0.22904) (10.5%)<br>HOMO–1 → LUMO+1 (0.44362) (39.4%)<br>HOMO–1 → LUMO+2 (–0.18369) (6.76%)<br>HOMO→ LUMO+2 (0.18504) (6.86%)
13	3.8438	322.55	0.1497	HOMO–5 → LUMO (0.60770) (74.1%)<br>HOMO–3 → LUMO (–0.16013) (5.14%)<br>HOMO → LUMO+1 (–0.22671) (10.3%)
14	4.0210	308.34	0.1098	HOMO–8 → LUMO (0.51358) (53.0%)<br>HOMO → LUMO+2 (–0.42381) (36.1%)
15	4.1191	301.00	0.1758	HOMO–3 → LUMO+1 (0.39989) (32.1%)<br>HOMO–2 → LUMO+2 (0.18784) (7.07%)<br>HOMO–1 → LUMO+2 (0.45238) (41.0%)
16	4.1344	299.89	0.0558	HOMO–9 → LUMO (–0.20097) (8.12%)<br>HOMO–2 → LUMO+1 (0.18001) (6.51%)<br>HOMO–2 → LUMO+2 (0.49248) (48.8%)<br>HOMO → LUMO+4 (0.28495) (16.3%)

18	4.3009	288.27	0.0358	HOMO–10 → LUMO (0.41456) (34.6%)<br>HOMO–9 → LUMO (0.50963) (52.3%)
19	4.3356	285.97	0.1666	HOMO–3 → LUMO+2 (–0.36361) (26.5%)<br>HOMO–2 → LUMO+2 (0.22921) (10.5%)<br>HOMO → LUMO+3 (0.40255) (32.5%)<br>HOMO → LUMO+4 (–0.20407) (8.36%)
20	4.3810	283.00	0.0861	HOMO–3 → LUMO+2 (0.26053) (13.6%)<br>HOMO → LUMO+3 (0.53152) (56.8%)<br>HOMO → LUMO+4 (0.26450) (14.1%)
21	4.4448	278.94	0.0546	HOMO–11 → LUMO (0.32017) (20.6%)<br>HOMO–4 → LUMO+1 (–0.24333) (11.9%)<br>HOMO–3 → LUMO+2 (0.34832) (24.4%)<br>HOMO–2 → LUMO+2 (0.22681) (10.3%)<br>HOMO→ LUMO+4 (–0.24951) (12.5%)
22	4.4792	276.80	0.1424	HOMO–11 → LUMO (–0.28079) (15.9%)<br>HOMO–2 → LUMO+2 (0.19410) (7.58%)<br>HOMO → LUMO+4 (–0.22947) (10.6%)<br>HOMO → LUMO+5 (0.46091) (42.8%)
23	4.5244	274.04	0.0368	HOMO–12 → LUMO (–0.36459) (26.8%)<br>HOMO–11 → LUMO (0.44125) (39.2%)<br>HOMO → LUMO+5 (0.30962) (19.3%)
24	4.5400	273.09	0.0235	HOMO–12 → LUMO (–0.26119) (13.7%)<br>HOMO–11 → LUMO (–0.27848) (15.6%)<br>HOMO → LUMO+5 (0.51462) (53.4%)
25	4.5659	271.54	0.0977	HOMO–12 → LUMO (0.39319) (31.1%)<br>HOMO–4 → LUMO+1 (0.29748) (17.8%)<br>HOMO–3 → LUMO+2 (0.18532) (6.92%)<br>HOMO → LUMO+5 (0.17039) (5.85%)<br>HOMO → LUMO+6 (0.26797) (14.5%)
26	4.6240	268.13	0.0927	HOMO–13 → LUMO (–0.28647) (16.5%)<br>HOMO–12 → LUMO (–0.18672) (7.02%)<br>HOMO–4 → LUMO+1 (0.32820) (21.7%)<br>HOMO–3 → LUMO+2 (0.24342) (11.9%)<br>HOMO → LUMO+4 (–0.16397) (5.42%)<br>HOMO → LUMO+6 (–0.28961) (16.9%)
27	4.6721	265.37	0.0208	HOMO–13 → LUMO (0.55825) (62.7%)<br>HOMO–4 → LUMO+1 (0.19410) (10.6%)<br>HOMO–1 → LUMO+3 (–0.22947) (6.71%)<br>HOMO–1 → LUMO+5 (0.46091) (7.04%)
28	4.7118	263.14	0.0763	HOMO–5 → LUMO+1 (0.52637) (55.7%)<br>HOMO–2 → LUMO+4 (–0.28935) (16.8%)<br>HOMO → LUMO+4 (–0.21240) (9.07%)
29	4.7852	259.10	0.1142	HOMO–13 → LUMO (0.23399) (11.0%)<br>HOMO–1 → LUMO+3 (0.49437) (49.2%)<br>HOMO–1 → LUMO+5 (0.23262) (10.9%)

30	4.8473	255.78	0.0109	HOMO–4 → LUMO+1 (–0.23948) (11.5%)<br>HOMO–4 → LUMO+2 (0.22707) (10.4%)<br>HOMO–1 → LUMO+4 (0.57336) (66.1%)

[a] Excitation energies with oscillator strength larger than 0.01 are listed. [b] Relative contribution larger than 5% is listed.

Table S6. TD-DFT vertical one-electron excitations calculated for **8**.

exited state	energy (eV)	wavelength (nm)	oscillator strength (*f*) [a]	description [b]
1	1.6594	747.17	0.0482	HOMO → LUMO (0.68247) (93.6%)
4	2.4527	505.51	0.0594	HOMO–3 → LUMO (–0.29865) (17.8%)<br>HOMO–1 → LUMO+1 (0.61617) (76.0%)
6	2.4991	496.12	0.0240	HOMO–4 → LUMO (0.64553) (83.3%)<br>HOMO–1 → LUMO+1 (–0.24109) (11.6%)
7	2.5035	495.25	0.1769	HOMO–4 → LUMO (0.59902) (71.4%)<br>HOMO–1 → LUMO+1 (0.30568) (18.6%)
9	2.7721	447.25	0.5145	HOMO–5 → LUMO (0.65321) (85.2%)<br>HOMO–3 → LUMO (–0.17121) (5.85%)
12	3.1535	393.17	0.0778	HOMO–7 → LUMO (0.27049) (14.7%)<br>HOMO–4 → LUMO+1 (0.59011) (69.8%)<br>HOMO → LUMO+2 (0.19742) (7.81%)
13	3.2047	386.88	0.0115	HOMO–8 → LUMO (0.66446) (88.6%)
14	3.2054	386.80	0.0217	HOMO–7 → LUMO (0.62676) (78.9%)
15	3.3392	371.29	0.0151	HOMO–5 → LUMO+1 (0.16022) (5.15%)<br>HOMO–3 → LUMO+1 (0.20133) (8.13%)<br>HOMO–3 → LUMO+2 (0.63035) (79.7%)
16	3.3438	370.79	0.1075	HOMO–4 → LUMO+1 (–0.21220) (9.03%)<br>HOMO → LUMO+2 (0.62773) (79.0%)
19	3.4691	357.39	0.1014	HOMO–11 → LUMO (0.61316) (75.5%)<br>HOMO–2 → LUMO+2 (–0.24908) (12.5%)
22	3.6713	337.71	0.0426	HOMO–13 → LUMO (0.44702) (40.2%)<br>HOMO–6 → LUMO+1 (0.48617) (47.5%)
23	3.6968	335.39	0.0432	HOMO–13 → LUMO (0.47805) (45.9%)<br>HOMO–6 → LUMO+1 (–0.39945) (32.0%)<br>HOMO–2 → LUMO+2 (0.16339) (5.35%)<br>HOMO–1 → LUMO+3 (0.18104) (6.58%)
25	3.7745	328.48	0.5175	HOMO–13 → LUMO (–0.17508) (6.15%)<br>HOMO–11 → LUMO (0.19025) (7.26%)<br>HOMO–6 → LUMO+1 (0.19645) (7.74%)<br>HOMO–3 → LUMO+2 (0.21943) (9.65%)<br>HOMO–2 → LUMO+2 (0.47328) (44.9%)<br>HOMO–1 → LUMO+3 (0.28404) (16.2%)

26	3.8904	318.70	0.0746	HOMO–8 → LUMO+1 (0.17549) (6.18%)<br>HOMO–3 → LUMO+2 (0.46425) (43.3%)<br>HOMO–1 → LUMO+3 (–0.43782) (38.5%)
27	3.8968	318.17	0.0402	HOMO–14 → LUMO (–0.21290) (9.08%)<br>HOMO → LUMO+3 (0.56501) (63.9%)
29	3.9723	312.12	0.0232	HOMO–8 → LUMO+1 (0.65143) (85.2%)
30	3.9874	310.94	0.0210	HOMO–15 → LUMO (0.21879) (9.63%)<br>HOMO–7 → LUMO+1 (0.50451) (51.2%)<br>HOMO–4 → LUMO+2 (0.31892) (20.5%)<br>HOMO → LUMO+3 (0.17755) (6.34%)

[a] Excitation energies with oscillator strength larger than 0.01 are listed. [b] Relative contribution larger than 5% is listed.

Table S7. TD-DFT vertical one-electron excitations calculated for **9**.

exited state	energy (eV)	wavelength (nm)	oscillator strength (*f*) [a]	description [b]
1	1.4856	834.59	0.0240	HOMO → LUMO (0.70363) (98.7%)
4	2.2733	545.40	0.0741	HOMO–1 → LUMO+1 (0.69468) (96.7%)
5	2.5070	494.55	0.2281	HOMO–4 → LUMO (0.33443) (22.4%)<br>HOMO–4 → LUMO+1 (0.25866) (13.4%)<br>HOMO–1 → LUMO (0.53020) (56.3%)
7	2.6136	474.38	0.1246	HOMO–5 → LUMO (0.45685) (41.8%)<br>HOMO–3 → LUMO+1 (–0.23152) (10.7%)<br>HOMO–2 → LUMO+1 (–0.43419) (37.8%)
8	2.6359	470.37	0.0129	HOMO–4 → LUMO (0.28468) (16.2%)<br>HOMO–3 → LUMO (0.51651) (53.4%)<br>HOMO–2 → LUMO+1 (0.36454) (26.6%)
9	2.7888	444.58	0.0976	HOMO–5 → LUMO (0.16248) (5.29%)<br>HOMO–4 → LUMO+1 (0.22558) (10.2%)<br>HOMO–3 → LUMO+1 (0.59897) (71.9%)
10	2.7907	444.27	0.0841	HOMO–7 → LUMO (–0.20049) (8.02%)<br>HOMO–4 → LUMO (–0.27775) (15.4%)<br>HOMO–2 → LUMO+1 (0.50779) (51.4%)
11	2.9618	418.61	0.1133	HOMO–6 → LUMO (0.46172) (42.7%)<br>HOMO–5 → LUMO (–0.26991) (14.6%)<br>HOMO–4 → LUMO+1 (0.41200) (34.0%)
13	2.9829	415.65	0.1612	HOMO–6 → LUMO (0.44023) (38.8%)<br>HOMO–5 → LUMO (0.26672) (14.3%)<br>HOMO–4 → LUMO+1 (–0.39770) (31.7%)<br>HOMO–3 → LUMO+1 (0.18384) (6.77%)
16	3.0409	407.72	0.0961	HOMO → LUMO+3 (0.67469) (91.3%)

17	3.1173	397.73	0.1197	HOMO–9 → LUMO (0.43832) (38.5%)<br>HOMO–8 → LUMO+1 (0.48940) (48.0%)
18	3.1380	395.11	0.1301	HOMO–8 → LUMO (0.19478) (7.56%)<br>HOMO–7 → LUMO (–0.30215) (18.2%)<br>HOMO–3 → LUMO (0.19740) (7.77%)<br>HOMO–2 → LUMO+1 (–0.20851) (8.66%)<br>HOMO → LUMO+2 (0.48652) (47.2%)
20	3.2054	386.80	0.0840	HOMO–7 → LUMO+1 (0.63979) (82.1%)
23	3.6365	340.94	0.0647	HOMO–1 → LUMO+2 (0.24113) (11.7%)<br>HOMO → LUMO+4 (0.61845) (77.3%)
24	3.6604	338.72	0.0496	HOMO–1 → LUMO+2 (0.64542) (83.7%)<br>HOMO → LUMO+4 (–0.19762) (7.85%)
25	3.6618	338.59	0.0129	HOMO–1 → LUMO+3 (0.68247) (93.6%)
26	3.6988	335.20	0.0729	HOMO–6 → LUMO (–0.18093) (6.58%)<br>HOMO–5 → LUMO (0.58688) (69.2%)<br>HOMO–4 → LUMO+1 (0.20196) (8.20%)
28	3.7361	331.85	0.0217	HOMO–6 → LUMO (–0.20339) (8.33%)<br>HOMO–5 → LUMO (0.3198) (20.6%)<br>HOMO–4 → LUMO+1 (–0.16588) (5.54%)<br>HOMO–3 → LUMO+1 (0.5195) (54.3%)

[a] Excitation energies with oscillator strength larger than 0.01 are listed. [b] Relative contribution larger than 5% is listed.

Table S8. TD-DFT vertical one-electron excitations calculated for **azulene**.

exited state	energy (eV)	wavelength (nm)	oscillator strength (*f*) [a]	description [b]
1[c]	2.4276	510.72	0.0079	HOMO → LUMO (0.69921) (96.7%)
3	4.7100	263.24	0.0519	HOMO–2 → LUMO (0.28090) (9.08%)<br>HOMO–1 → LUMO+1 (0.63839) (80.5%)
4	5.1662	239.99	1.0370	HOMO–1 → LUMO (0.52214) (49.2%)<br>HOMO → LUMO+1 (0.48267) (42.1%)
6	6.0533	204.82	0.2406	HOMO–2 → LUMO (0.62756) (76.8%)<br>HOMO–1 → LUMO+1 (–0.25754) (12.9%)
11	6.8714	180.43	0.0776	HOMO–3 → LUMO+1 (0.39094) (30.5%)<br>HOMO → LUMO+3 (0.56193) (63.0%)
12	6.8944	179.83	0.1472	HOMO–3 → LUMO (0.37686) (28.0%)<br>HOMO–2 → LUMO+1 (0.54845) (59.4%)
18	7.6683	161.68	0.0142	HOMO–3 → LUMO+1 (0.53998) (57.8%)<br>HOMO → LUMO+3 (–0.39201) (30.4%)<br>HOMO → LUMO+5 (0.16589) (5.45%)

24	8.0810	153.43	0.0250	HOMO–1 → LUMO+3 (0.58586) (68.6%)<br>HOMO → LUMO+8 (0.34207) (23.4%)
25	8.0837	153.38	0.1932	HOMO–8 → LUMO+1 (0.22203) (9.80%)<br>HOMO–3 → LUMO+1 (–0.16833) (5.63%)<br>HOMO → LUMO+5 (0.62843) (78.5%)

[a] Excitation energies with oscillator strength larger than 0.01 are listed. [b] Relative contribution larger than 5% is listed. [c]

Table S9. Cartesian coordinates of optimized **6ba** [B3LYP/6-31g(d) level of theory].

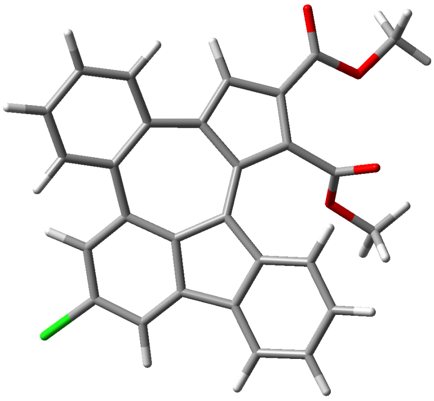

C	3.66856900	0.71511100	0.74727900	C	-4.14371200	1.65015000	0.54087900
C	4.19971200	-0.54396400	0.98272500	O	-4.61368500	2.77280100	0.52094900
C	3.54006300	-1.71323700	0.58538800	O	-4.83106700	0.56285200	0.94588700
C	2.29002400	-1.56580400	0.01236500	C	-6.20227500	0.79753400	1.29941000
C	1.68380900	-0.28823800	-0.15919100	C	-2.83132700	-1.18250700	0.01388900
C	2.40810200	0.88628800	0.12220000	O	-3.70146300	-1.60228000	-0.71998600
Cl	5.75796600	-0.67877400	1.78202300	O	-2.36352800	-1.85849900	1.08475500
C	0.28659500	-0.51069000	-0.55381300	C	-2.96631000	-3.14344700	1.31094600
C	-0.72343200	0.40006500	-0.34294700	H	4.23402300	1.58163900	1.06430100
C	-0.53727100	1.86729300	-0.20740200	H	3.99214100	-2.68604100	0.74454400
C	0.65728200	2.66484600	-0.49806800	H	2.49056500	-4.43872700	-0.45383900
C	2.00355300	2.23886500	-0.31637800	H	0.70364700	-5.71668700	-1.61799200
C	1.38074000	-2.58587500	-0.52432200	H	-1.34182300	-4.56848000	-2.41452600
C	0.18326900	-1.94096900	-0.91188500	H	-1.68019100	-2.15901900	-1.99684800
C	1.57244800	-3.94233300	-0.75637700	H	-0.58175700	4.29815200	-1.15143300
C	0.56771800	-4.65698100	-1.42046400	H	1.25201400	5.86339500	-1.59604500
C	-0.59015200	-4.01151900	-1.86262900	H	3.62312700	5.10230500	-1.29776500
C	-0.78913600	-2.64655600	-1.62070400	H	4.06909500	2.81750400	-0.55876100
C	0.43846500	3.98232300	-0.96593000	H	-2.01859300	3.43898200	0.22375300
C	1.47219700	4.86435100	-1.23033100	H	-6.60616200	-0.18058600	1.56142700
C	2.79369400	4.44194300	-1.06163800	H	-6.26739600	1.48307700	2.14881600
C	3.03980800	3.15093100	-0.62425000	H	-6.74814500	1.22498600	0.45437600
C	-2.14844100	0.13564600	-0.12812000	H	-2.51415100	-3.51835000	2.22960900
C	-2.75113000	1.34704300	0.14386700	H	-4.04846300	-3.04259100	1.42493700
C	-1.77161500	2.39932600	0.06077900	H	-2.75290100	-3.81695000	0.47654300

Table S10. Cartesian coordinates of optimized **6fa** [B3LYP/6-31g(d) level of theory].

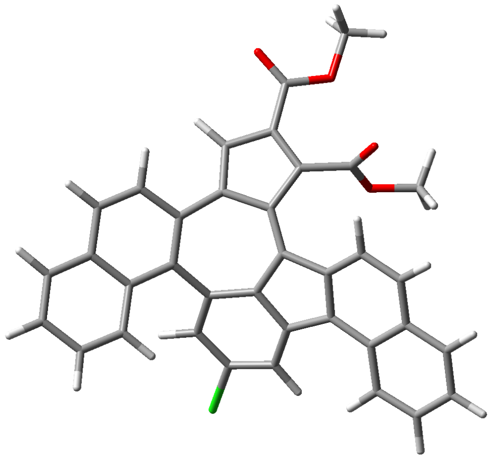

C	-2.75117100	1.27613200	1.28865100	C	-6.06314900	0.60182000	-1.12880200
C	-2.22578200	2.48793300	1.70158600	C	-4.74079400	0.55200200	-0.73750500
C	-0.98600400	2.96128500	1.24557100	C	2.25959900	-4.53216400	0.55562400
C	-0.23955000	2.13096600	0.42390600	O	1.83032400	-5.66927700	0.62104600
C	-0.70928800	0.81688600	0.11597700	O	3.54286200	-4.20582800	0.81398400
C	-2.01764000	0.41670000	0.43190000	C	4.40937500	-5.30882700	1.11867400
Cl	-3.15122500	3.49139900	2.80868900	C	3.19976600	-1.59236400	-0.11406300
C	0.42677500	0.06621400	-0.41633600	O	4.03398500	-1.86321300	-0.95190100
C	0.55628900	-1.29576100	-0.25451000	O	3.44215600	-0.81859800	0.96438200
C	-0.57307100	-2.23943100	-0.02526400	C	4.78574000	-0.31929100	1.07286000
C	-2.00045600	-2.01362500	-0.25828200	H	-3.73323200	0.97766600	1.63249700
C	-2.67150200	-0.78317300	-0.12996400	H	-0.63399500	3.92588100	1.58387400
C	1.07069400	2.31013400	-0.23877700	H	4.25381400	1.95254900	-2.45318700
C	1.44924900	1.06674400	-0.76441400	H	2.83051000	-0.01640800	-2.04974900
C	1.86388000	3.46937800	-0.47275100	H	-2.24167500	-4.11405100	-0.73385900
C	3.05588100	3.30656100	-1.26706800	H	-4.64745400	-4.02090200	-1.13509400
C	3.37578300	2.03647400	-1.81792600	H	-0.52504700	-4.39344900	0.44631900
C	2.58655800	0.93233200	-1.58836100	H	0.63974200	4.94377900	0.55524500
C	-2.76134800	-3.16938100	-0.62937500	H	2.10880400	6.83381900	0.10131600
C	-4.10196900	-3.11725600	-0.87436600	H	4.19929900	6.53055000	-1.22422200
C	-4.78833100	-1.87445900	-0.87470600	H	4.78003000	4.29555900	-2.11409700
C	-4.06022700	-0.68540200	-0.54869800	H	-6.67880200	-2.72285300	-1.47426600
C	1.78761400	-2.07571600	-0.12233500	H	-7.83504100	-0.53396900	-1.66034000
C	1.42037400	-3.36606100	0.20394500	H	-6.54120900	1.56717400	-1.27083400
C	-0.01415600	-3.46609200	0.22994900	H	-4.20496500	1.48359500	-0.60658400
C	1.55038500	4.77623200	-0.00383500	H	5.39814200	-4.87152500	1.25875600
C	2.37354800	5.84737100	-0.26910100	H	4.07843800	-5.81624300	2.02899700
C	3.55716000	5.67717500	-1.02522900	H	4.41983900	-6.02693200	0.29468000
C	3.88204400	4.43431800	-1.51696000	H	4.81347300	0.23735600	2.01000800
C	-6.15213100	-1.79779400	-1.25229100	H	5.49893600	-1.14712000	1.09265000
C	-6.79094500	-0.58320300	-1.36437600	H	5.01741700	0.33712100	0.23002400

Table S11. Cartesian coordinates of optimized **8** [B3LYP/6-31g(d) level of theory].

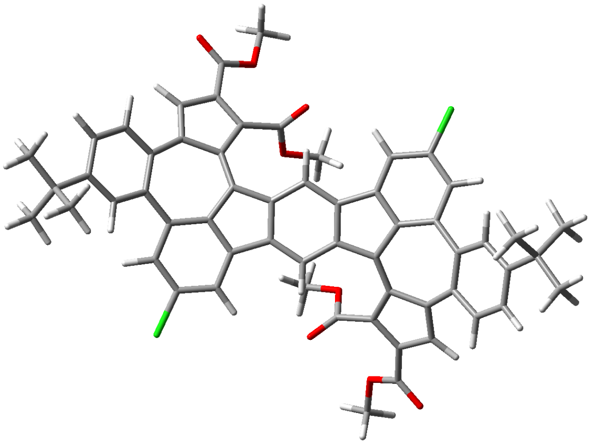

C	-0.09510000	3.01891700	3.63452100
C	0.52849000	4.15858300	4.12322900
C	0.81225100	5.25715000	3.30387600
C	0.51510600	5.13279200	1.95761200
C	-0.03583900	3.93898400	1.41551100
C	-0.43583000	2.89370300	2.26735700
Cl	0.94754600	4.24074800	5.82850900
C	-0.00337000	4.05425400	-0.05312500
C	0.04450300	2.96247500	-0.89621900
C	-0.38931400	1.59605800	-0.54665100
C	-1.34066600	1.25085800	0.51973900
C	-1.32642200	1.79441800	1.83503600
C	0.65345000	6.10958200	0.86989200
C	0.29410800	5.46453000	-0.34114600
C	0.97682100	7.45230800	0.90649700
C	0.91012600	8.19565700	-0.28845000
C	0.49351200	7.58517900	-1.48091100
C	0.18173000	6.22195600	-1.50435500
C	-2.34168300	0.30576500	0.22415100
C	-3.26049900	-0.15200000	1.15825200
C	-3.21160700	0.35702900	2.46148200
C	-2.26447800	1.33149900	2.77247000
C	0.71511300	2.86709600	-2.19000700
C	0.76984500	1.53548600	-2.53486300
C	0.06837900	0.73393600	-1.52609000
C	0.09510000	-3.01891700	3.63452100
C	-0.52849000	-4.15858300	4.12322900
C	-0.81225100	-5.25715000	3.30387600
C	-0.51510600	-5.13279200	1.95761200
C	0.03583900	-3.93898400	1.41551100
C	0.43583000	-2.89370300	2.26735700
Cl	-0.94754600	-4.24074800	5.82850900
C	0.00337000	-4.05425400	-0.05312500
C	-0.04450300	-2.96247500	-0.89621900
C	0.38931400	-1.59605800	-0.54665100
C	1.34066600	-1.25085800	0.51973900
C	1.32642200	-1.79441800	1.83503600
C	-0.65345000	-6.10958200	0.86989200
C	-0.29410800	-5.46453000	-0.34114600
C	-0.97682100	-7.45230800	0.90649700
C	-0.91012600	-8.19565700	-0.28845000
C	-0.49351200	-7.58517900	-1.48091100
C	-0.18173000	-6.22195600	-1.50435500
C	2.34168300	-0.30576500	0.22415100
C	3.26049900	0.15200000	1.15825200
C	3.21160700	-0.35702900	2.46148200
C	2.26447800	-1.33149900	2.77247000
C	-0.71511300	-2.86709600	-2.19000700
C	-0.76984500	-1.53548600	-2.53486300
C	-0.06837900	-0.73393600	-1.52609000
C	-1.52645700	-3.90805800	-2.89117900
O	-0.91012600	-4.34010600	-4.01570900
O	-1.60087300	0.29130500	-3.77173800
C	-1.55746100	-1.05754900	-3.69137400
C	1.52645700	3.90805800	-2.89117900
O	0.91012600	4.34010600	-4.01570900
O	1.60087300	-0.29130500	-3.77173800
C	1.55746100	1.05754900	-3.69137400
O	-2.59782700	-4.32055300	-2.50652000
O	-2.13447400	-1.77795600	-4.48760600
O	2.13447400	1.77795600	-4.48760600
O	2.59782700	4.32055300	-2.50652000
O	1.24397500	9.50786000	-0.17164200
C	1.20515200	10.32747000	-1.33208900
O	-4.05673200	0.00429900	3.47084500
C	-5.04039100	-0.98551500	3.20605800
O	-1.24397500	-9.50786000	-0.17164200
C	-1.20515200	-10.32747000	-1.33208900
O	4.05673200	-0.00429900	3.47084500
C	5.04039100	0.98551500	3.20605800
C	-2.35756000	0.82505800	-4.86807300
C	-1.71819900	-5.17609800	-4.85909700
C	2.35756000	-0.82505800	-4.86807300
C	1.71819900	5.17609800	-4.85909700
H	-0.31986100	2.21082000	4.31920700
H	1.25943500	6.15534600	3.71561000
H	1.25798400	7.96263400	1.82240100
H	0.39681000	8.16313900	-2.39220300
H	-0.16128200	5.77614700	-2.43064500
H	-2.41105200	-0.06896100	-0.78910700
H	-4.00932100	-0.87572600	0.85797800
H	-2.31140400	1.76327700	3.76518300
H	0.31986100	-2.21082000	4.31920700
H	-1.25943500	-6.15534600	3.71561000
H	-1.25798400	-7.96263400	1.82240100
H	-0.39681000	-8.16313900	-2.39220300
H	0.16128200	-5.77614700	-2.43064500
H	2.41105200	0.06896100	-0.78910700
H	4.00932100	0.87572600	0.85797800
H	2.31140400	-1.76327700	3.76518300

H	1.52774100	11.31778200	-1.00683200
H	1.88842200	9.95711200	-2.10668100
H	0.18979100	10.39284300	-1.74352000
H	-5.57855100	-1.12889900	4.14463800
H	-5.74509100	-0.65699800	2.43084100
H	-4.58371300	-1.93545400	2.90028200
H	-0.18979100	-10.39284300	-1.74352000
H	-1.52774100	-11.31778200	-1.00683200
H	-1.88842200	-9.95711200	-2.10668100
H	4.58371300	1.93545400	2.90028200
H	5.57855100	1.12889900	4.14463800
H	5.74509100	0.65699800	2.43084100
H	-3.40932500	0.53801000	-4.78353200
H	-2.24411500	1.90691900	-4.79578500
H	-1.96570800	0.45863700	-5.82053700
H	-2.04198600	-6.07253800	-4.32306100
H	-2.59449600	-4.61769900	-5.19724500
H	-1.08201500	-5.43991200	-5.70502200
H	2.24411500	-1.90691900	-4.79578500
H	1.96570800	-0.45863700	-5.82053700
H	3.40932500	-0.53801000	-4.78353200
H	1.08201500	5.43991200	-5.70502200
H	2.04198600	6.07253800	-4.32306100
H	2.59449600	4.61769900	-5.19724500

Table S12. Cartesian coordinates of optimized **9** [B3LYP/6-31g(d) level of theory].

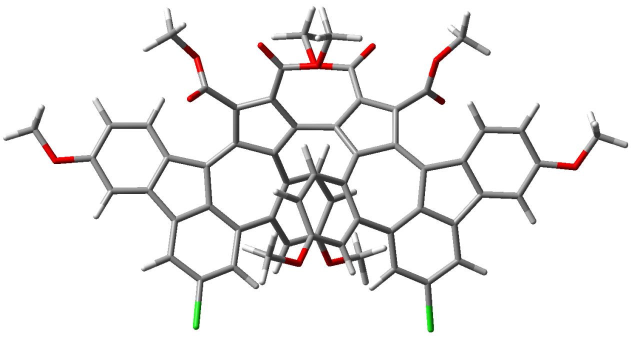

C	1.38997300	5.31601800	0.50109900
C	0.03359800	5.15552900	0.25693500
C	-0.54741900	3.89159100	0.09667000
C	0.30967400	2.80523600	0.11995300
C	1.71883300	2.94888800	0.27171500
C	2.27430900	4.21036000	0.56253200
Cl	-0.98927500	6.57885300	0.16460000
C	2.33196300	1.64581500	-0.01808600
C	3.63326900	1.47654800	-0.43553200
C	4.73907900	2.43954600	-0.18965100
C	4.76090200	3.58525400	0.72249700
C	3.65457200	4.41853600	1.04944700
C	-0.00023200	1.37846200	-0.00589000
C	1.22453200	0.66932000	-0.05303900
C	-1.23071200	0.72682300	0.01214800
C	1.23071200	-0.72682300	0.01214800
C	6.00343900	3.91143700	1.30611400
C	6.18128500	4.99551200	2.14999300
C	5.10081000	5.82092500	2.49686900
C	3.86542400	5.49413700	1.93865700
C	4.18987700	0.40121100	-1.26241800
C	5.50736900	0.72339300	-1.51506800
C	5.84289700	1.94841500	-0.83536100
C	-1.38997300	-5.31601800	0.50109900
C	-0.03359800	-5.15552900	0.25693500
C	0.54741900	-3.89159100	0.09667000
C	-0.30967400	-2.80523600	0.11995300
C	-1.71883300	-2.94888800	0.27171500
C	-2.27430900	-4.21036000	0.56253200
Cl	0.98927500	-6.57885300	0.16460000
C	-2.33196300	-1.64581500	-0.01808600
C	-3.63326900	-1.47654800	-0.43553200
C	-4.73907900	-2.43954600	-0.18965100
C	-4.76090200	-3.58525400	0.72249700
C	-3.65457200	-4.41853600	1.04944700
C	0.00023200	-1.37846200	-0.00589000
C	-1.22453200	-0.66932000	-0.05303900
C	-6.00343900	-3.91143700	1.30611400
C	-6.18128500	-4.99551200	2.14999300
C	-5.10081000	-5.82092500	2.49686900
C	-3.86542400	-5.49413700	1.93865700
C	-4.18987700	-0.40121100	-1.26241800
C	-5.50736900	-0.72339300	-1.51506800
C	-5.84289700	-1.94841500	-0.83536100
C	6.50046100	0.04616800	-2.37732200
O	7.68975800	0.30310600	-2.35562100
O	5.95177300	-0.87084200	-3.20092000
C	6.87974800	-1.58947800	-4.02802600
C	3.47967600	-0.73741100	-1.91530900
O	3.64192100	-1.90949500	-1.64188200
O	2.62859500	-0.30323100	-2.86695700
C	1.91560100	-1.33492500	-3.57141900
C	-6.50046100	-0.04616800	-2.37732200
O	-7.68975800	-0.30310600	-2.35562100
O	-5.95177300	0.87084200	-3.20092000
C	-6.87974800	1.58947800	-4.02802600
C	-3.47967600	0.73741100	-1.91530900
O	-3.64192100	1.90949500	-1.64188200
O	-2.62859500	0.30323100	-2.86695700
C	-1.91560100	1.33492500	-3.57141900
C	5.22380700	7.00966000	3.46673900
C	4.33467500	6.75810800	4.70797400
C	4.76090200	8.30505200	2.75824200
C	6.67300600	7.21793400	3.94650300
C	-5.22380700	-7.00966000	3.46673900
C	-4.33467500	-6.75810800	4.70797400

C	-4.76090200	-8.30505200	2.75824200	H	-7.39939200	0.90527100	-4.70432300
C	-6.67300600	-7.21793400	3.94650300	H	-7.61741100	2.11051400	-3.41240100
H	1.77524400	6.31901000	0.63011000	H	-1.32403200	0.81522900	-4.32568100
H	-1.61305600	3.78096500	-0.07463000	H	-2.61678700	2.02886800	-4.04114800
H	-2.15159900	1.28832800	0.10327200	H	-1.26480000	1.88715300	-2.88861100
H	2.15159900	-1.28832800	0.10327200	H	4.64421700	5.84833100	5.23470100
H	6.85146600	3.26562500	1.10777500	H	4.41198900	7.59923200	5.40755500
H	7.16836800	5.17279600	2.56191700	H	3.27912700	6.64568300	4.43883900
H	3.00227600	6.07660700	2.23774800	H	3.71722900	8.24283500	2.43233000
H	6.82927200	2.38813900	-0.87959000	H	4.84490900	9.16086400	3.43879100
H	-1.77524400	-6.31901000	0.63011000	H	5.37599300	8.51151200	1.87504200
H	1.61305600	-3.78096500	-0.07463000	H	7.04992200	6.34995300	4.49924000
H	-6.85146600	-3.26562500	1.10777500	H	6.71713700	8.08076600	4.62023100
H	-7.16836800	-5.17279600	2.56191700	H	7.35495500	7.41569200	3.11158200
H	-3.00227600	-6.07660700	2.23774800	H	-4.64421700	-5.84833100	5.23470100
H	-6.82927200	-2.38813900	-0.87959000	H	-4.41198900	-7.59923200	5.40755500
H	6.27640200	-2.30286700	-4.58969900	H	-3.27912700	-6.64568300	4.43883900
H	7.39939200	-0.90527100	-4.70432300	H	-3.71722900	-8.24283500	2.43233000
H	7.61741100	-2.11051400	-3.41240100	H	-4.84490900	-9.16086400	3.43879100
H	1.32403200	-0.81522900	-4.32568100	H	-5.37599300	-8.51151200	1.87504200
H	2.61678700	-2.02886800	-4.04114800	H	-7.04992200	-6.34995300	4.49924000
H	1.26480000	-1.88715300	-2.88861100	H	-6.71713700	-8.08076600	4.62023100
H	-6.27640200	2.30286700	-4.58969900	H	-7.35495500	-7.41569200	3.11158200

Table S13. Cartesian coordinates of optimized **azulene** [B3LYP/6-31g(d) level of theory].

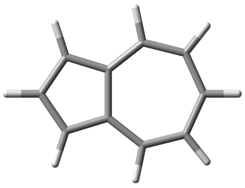

C	0.55196500	1.59468500	0.00000000	C	-1.90204500	1.14996800	0.00000100
C	1.91098600	1.26636600	0.00000000	H	0.32393000	2.66087500	0.00000000
C	2.50380700	0.00000000	0.00000000	H	2.59920300	2.10903800	-0.00000100
C	1.91098600	-1.26636600	0.00000000	H	3.59310800	-0.00000100	0.00000000
C	0.55196500	-1.59468600	0.00000000	H	2.59920300	-2.10903800	0.00000100
C	-0.55432400	-0.74989100	0.00000000	H	0.32393000	-2.66087600	0.00000000
C	-0.55432400	0.74989000	0.00000100	H	-2.24823500	-2.17701300	-0.00000100
C	-1.90204600	-1.14996800	0.00000000	H	-3.79372400	0.00000100	0.00000000
C	-2.70850200	0.00000000	0.00000000	H	-2.24823100	2.17701400	0.00000100

6.3. Theoretical Mechanistic Studies (Figure 5)

Table S14. Summary of the Gibbs-free energies and imaginary frequencies.

	G (hartree) M06/6-311+G(d,p)&SDD +PCM[$(CH_2Cl)_2$]	*G* (hartree) M06/6-31G(d)&LANL2DZ	Imaginary frequency (cm^{-1}) M06/6-31G(d)&LANL2DZ
**Diyne**	-845.960060	-845.755346	None
**DMAD**	-532.854077	-532.702529	None
**(*R*)-P-phos / Rh^+**	-2670.633230	-2668.988187	None
**IM1**	-4049.500938	-4047.496370	None
**TS1a**	-4049.477950	-4047.471368	-324.5146
**TS1s**	-4049.486414	-4047.482899	-287.4176
**IM2a**	-4049.530232	-4047.527064	None
**IM2s**	-4049.528089	-4047.525846	None
**TS2**	-4049.515064	-4047.515945	-90.6998
**TS3a**	-4049.490035	-4047.485937	-140.2567
**TS3s**	-4049.491124	-4047.490755	-236.1874
**IM3a**	-4049.523654	-4047.519482	None
**IM3s**	-4049.577723	-4047.580216	None
**TS4**	-4049.505179	-4047.500691	-1048.8956
**IM4**	-4049.550588	-4047.551028	None
**TS5**	-4049.525586	-4047.522784	-201.9404
**IM5**	-4049.589509	-4047.589826	None
**TS6**	-4049.553222	-4047.551741	-369.8448
**IM6**	-4049.616461	-4047.616477	None

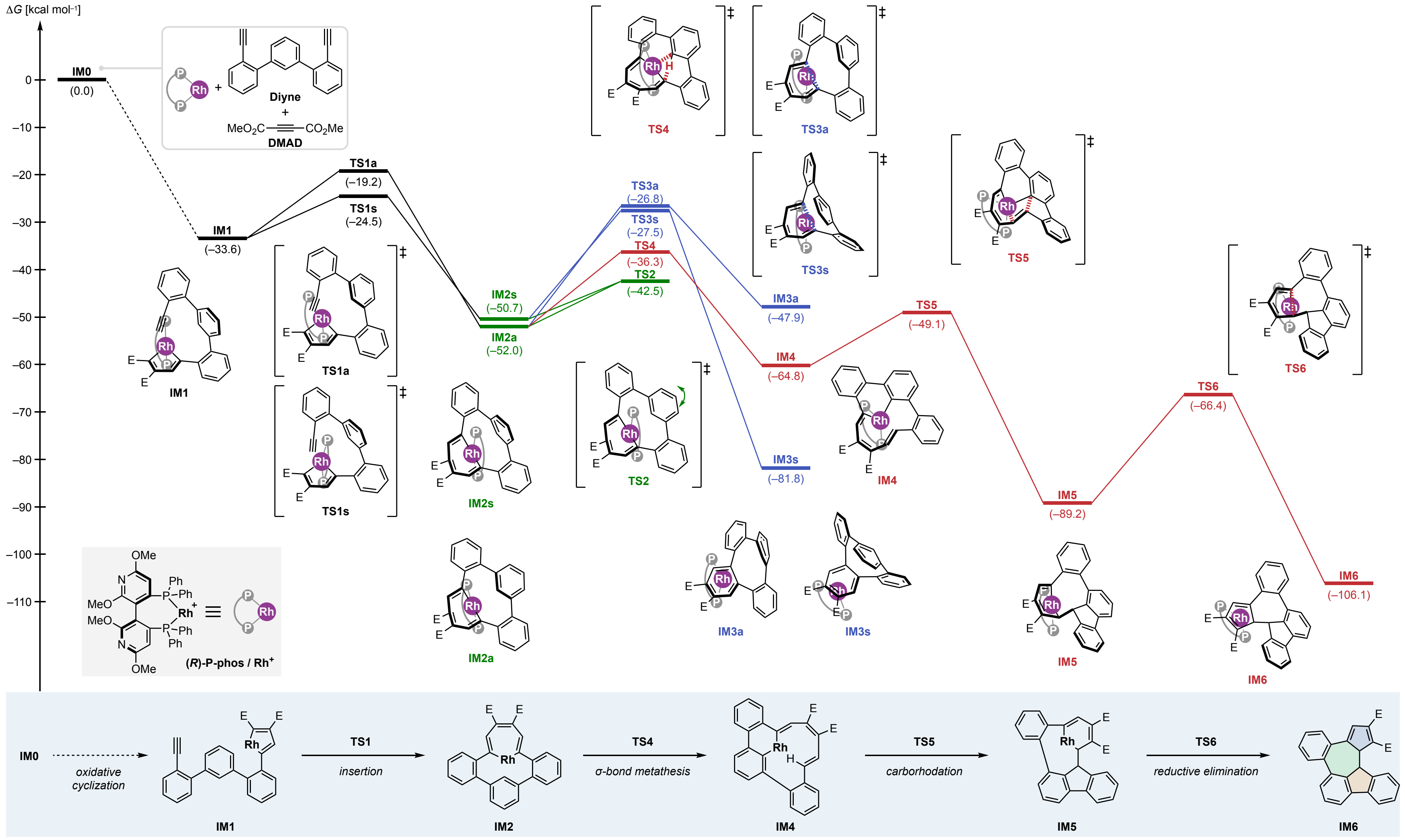

Figure S33. Energy diagram of proposed reaction pathways. Energy changes are shown in kcal mol^{-1}, and represent the relative free energies calculated at the M06/6-311+G(d,p)&SDD(Rh)+PCM[$(CH_2Cl)_2$]//M06/6-31G(d)&LANL2DZ(Rh) levels of theory.

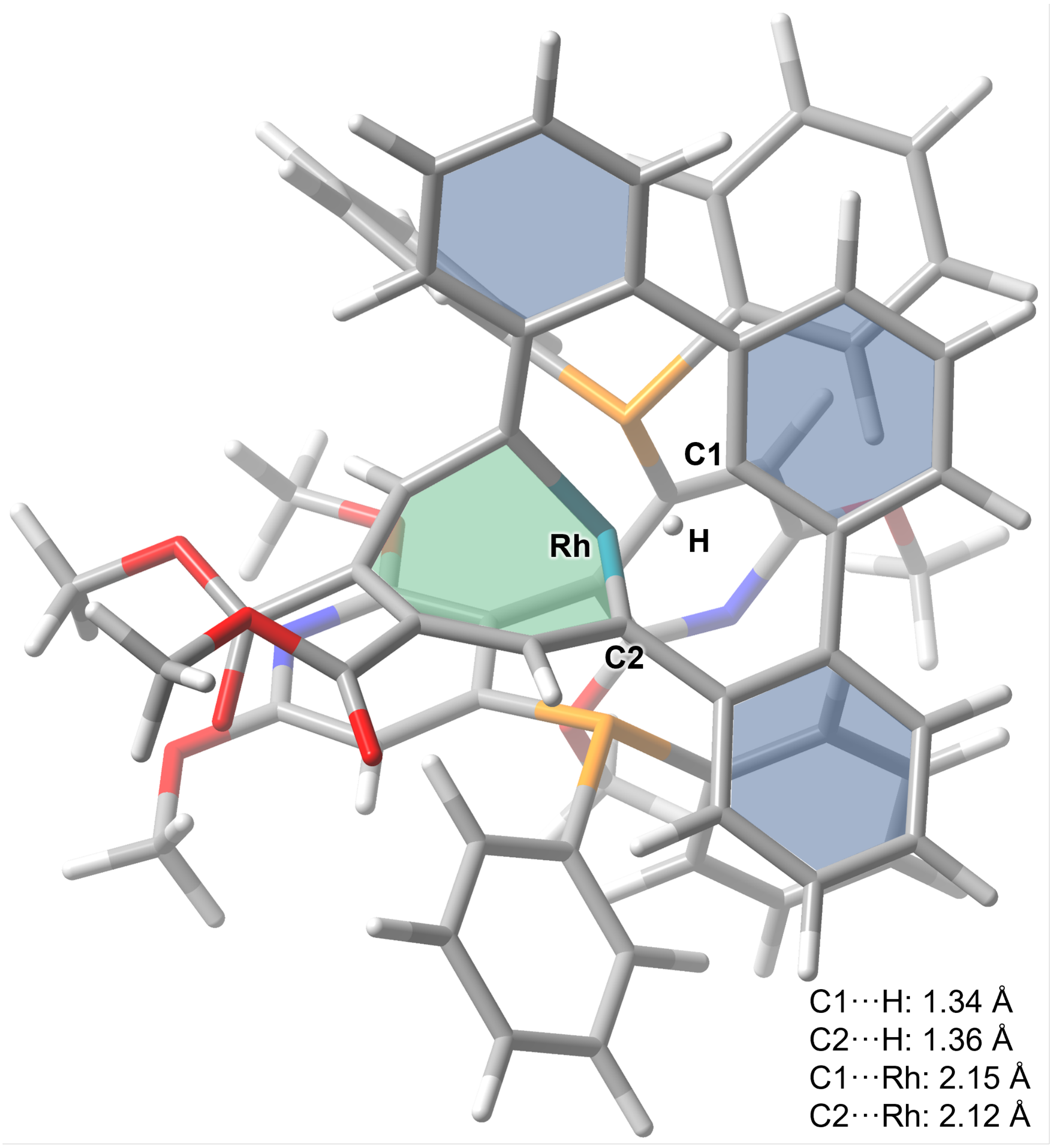

Figure S34. Structure and selected bond lengths for **TS4**.

Cartesian Coordinates of the Computed Structures

Diyne

C	-4.05732500	-1.44360400	-0.67771200
C	-4.99981300	-1.02121600	0.24645900
C	-4.70780300	0.05259300	1.08138200
C	-3.47680000	0.68717500	0.98582600
C	-2.50735100	0.27718400	0.06626200
C	-2.81174200	-0.80833000	-0.78426100
C	-1.21682400	0.99894500	0.01550300
C	0.00000800	0.31446500	-0.00005800
C	1.21684000	0.99894200	-0.01561700
C	1.20298400	2.39776200	-0.02018500
C	0.00001800	3.09080700	-0.00005700
C	-1.20294800	2.39776700	0.02007400
C	2.50736200	0.27716600	-0.06632500
C	3.47680900	0.68703600	-0.98593800
C	4.70781800	0.05244400	-1.08139900
C	4.99982700	-1.02124500	-0.24632600
C	4.05732700	-1.44352200	0.67788900
C	2.81174200	-0.80824200	0.78433900
C	1.91040400	-1.26821600	1.79260000
C	-1.91044800	-1.26841400	-1.79251300
C	-1.18926400	-1.69092700	-2.66578000
C	1.18919000	-1.69072800	2.66583900
H	-4.27216200	-2.27390800	-1.34806900
H	-5.96053200	-1.52800600	0.31380800
H	-5.43684200	0.39267800	1.81474000
H	-3.23960400	1.51249100	1.65646800
H	0.00001400	-0.77371000	-0.00004200
H	2.14756400	2.94110000	-0.01643500
H	0.00002000	4.17949000	-0.00005800
H	-2.14752200	2.94111700	0.01632200
H	3.23961600	1.51227400	-1.65668000
H	5.43686300	0.39243300	-1.81479500
H	5.96055100	-1.52803700	-0.31359300
H	4.27216700	-2.27374000	1.34835200
H	-0.53573300	-2.05073200	-3.43109200
H	0.53553300	-2.05048700	3.43106300

DMAD

C	-0.60076700	0.20261400	0.06376500
C	0.60076000	0.20257300	-0.06380800
C	-2.03244600	0.25058900	0.27858500
O	-2.56706200	0.96922700	1.08585000
O	-2.66072200	-0.60376000	-0.53385300
C	-4.08168600	-0.62288900	-0.39245000
C	2.03243900	0.25056200	-0.27861000
O	2.56709400	0.96921900	-1.08583100
O	2.66070900	-0.60378200	0.53386300
C	4.08167800	-0.62287000	0.39247100
H	-4.44019700	-1.35794500	-1.11449400
H	-4.50078400	0.36565400	-0.60698100
H	-4.36118600	-0.91310800	0.62568100
H	4.44019500	-1.35802600	1.11441100
H	4.50074900	0.36565300	0.60713800
H	4.36119700	-0.91294100	-0.62569800

(*R*)-P-phos / Rh^+

N	2.11506600	2.43102000	2.08008400
C	1.01624000	1.69913300	2.19652700
C	0.71682800	0.57817400	1.39653200
C	1.68292000	0.23905300	0.43633100
C	2.82799000	1.01164100	0.27906200
C	2.98709100	2.10686500	1.13468700
C	-0.58291600	-0.11510900	1.54643800
C	-0.84621200	-0.91278600	2.67575300
N	-1.97345600	-1.58267500	2.87263000
C	-2.92463000	-1.49647500	1.95277000
C	-2.80836200	-0.72594500	0.78985600
C	-1.61635400	-0.04047800	0.59855900
P	-1.29213100	0.80562400	-0.98735400
P	1.28611900	-1.05462300	-0.78552200
Rh	-0.00907700	-0.29693400	-2.41502000
O	0.10266000	2.04875500	3.10545700
C	0.38409000	3.18629700	3.90838300
O	0.13155000	-1.01373700	3.57938500
C	-0.10345600	-1.84888500	4.70403600
O	-4.05765600	-2.18105200	2.11313600
C	-4.15862100	-3.01767800	3.25755400
O	4.08006600	2.85220100	0.96534100
C	4.22933100	3.99447700	1.79724200
C	2.81959200	-1.59515500	-1.60142800
C	2.71406700	-1.92340000	-2.95795800
C	3.81845300	-2.38752700	-3.66245000
C	5.03809700	-2.53149100	-3.00848300
C	5.14965900	-2.22039800	-1.65547800
C	4.04589200	-1.75683100	-0.94913100
C	0.69238300	-2.50424500	0.14682800
C	-0.41198000	-3.21979900	-0.31990600
C	-0.84733200	-4.35269100	0.35986900
C	-0.17370800	-4.77835000	1.50037200
C	0.93737200	-4.07446500	1.96196000
C	1.37129500	-2.93772700	1.29109000
C	-2.85370100	0.94561500	-1.90779600
C	-3.51135700	2.16836700	-2.08138500
C	-4.67104700	2.22824800	-2.84581800
C	-5.17641300	1.07836000	-3.44607600
C	-4.52157500	-0.14051400	-3.28813800
C	-3.36052800	-0.20807800	-2.52844500
C	-0.76870800	2.48770200	-0.54729100
C	-1.41222600	3.17911100	0.48645800
C	-1.02248700	4.47708500	0.79379500
C	0.01358400	5.08217500	0.08393500
C	0.65557200	4.39638800	-0.94288700

C	0.26222500	3.10112100	-1.26327000
H	3.57868100	0.81421800	-0.48160800
H	-3.63251400	-0.69683700	0.08210500
H	-0.48724700	3.31212100	4.55482900
H	1.28419200	3.02623900	4.51273400
H	0.53245900	4.07698000	3.28497300
H	0.80997800	-1.80132700	5.30096600
H	-0.95766600	-1.48970900	5.28894500
H	-0.30123200	-2.88076500	4.38709800
H	-5.13415200	-3.50204400	3.18257600
H	-3.35842900	-3.76812200	3.26104500
H	-4.09636400	-2.43081900	4.18102700
H	5.16360500	4.46530700	1.48517200
H	3.38968000	4.68755600	1.66249100
H	4.28295900	3.70751000	2.85359900
H	1.74922700	-1.84217300	-3.47385200
H	3.72694100	-2.63940900	-4.71674200
H	5.90655500	-2.89338700	-3.55498800
H	6.10358600	-2.34080200	-1.14613200
H	4.14499600	-1.51308300	0.10810200
H	-0.93555800	-2.87140000	-1.21247400
H	-1.71384300	-4.90285100	-0.00195300
H	-0.51064000	-5.66729800	2.03067800
H	1.46824600	-4.41213200	2.85065100
H	2.22266700	-2.37498600	1.67519800
H	-3.11822000	3.07282100	-1.61967900
H	-5.18263600	3.17986900	-2.97429000
H	-6.08365100	1.13284100	-4.04441800
H	-4.91424100	-1.03876100	-3.76001600
H	-2.84278600	-1.16171400	-2.40244900
H	-2.20802400	2.69838200	1.05717700
H	-1.52699600	5.01765500	1.59299400
H	0.31834800	6.09747300	0.33165600
H	1.46533100	4.86901000	-1.49529200
H	0.76478400	2.54818800	-2.05922200

IM1

N	-2.00783400	-4.47990300	1.85403300
C	-2.49606900	-3.54527900	1.05295000
C	-1.71842700	-2.60995600	0.34200200
C	-0.33262500	-2.73264300	0.48514000
C	0.20088000	-3.70589500	1.32244800
C	-0.69128100	-4.54005000	2.00055200
C	-2.43828000	-1.58172300	-0.44498500
C	-3.15423300	-1.99744900	-1.59508600
N	-4.02653300	-1.26202600	-2.25415100
C	-4.28135200	-0.03716100	-1.80140700
C	-3.60133400	0.52648900	-0.72191100
C	-2.65299900	-0.25313300	-0.05255600
P	-1.52991800	0.63799100	1.10896200
P	0.74673000	-1.61039900	-0.49733600
Rh	0.36469000	1.07954000	-0.27220500
O	-3.82714100	-3.43387800	0.93754600
C	-4.62517600	-4.34596900	1.67533100
O	-2.89421000	-3.23493800	-2.02962800
C	-3.53769600	-3.64965300	-3.22477300
O	-5.21727400	0.60689100	-2.50920500
C	-5.57147000	1.92437100	-2.11988900
O	-0.16494900	-5.43706500	2.84041800
C	-1.06957200	-6.23991900	3.58141300
C	2.42142500	-2.18464800	-0.01213200
C	2.91293300	-1.79725800	1.24104700
C	4.15634300	-2.23480600	1.68051700
C	4.92933400	-3.06009700	0.86826600
C	4.45173700	-3.44530500	-0.38013600
C	3.20401900	-3.01293300	-0.81982700
C	0.46891600	-2.31393500	-2.17142600
C	0.43916500	-1.48014200	-3.29097600
C	0.23518100	-2.01570600	-4.56137000
C	0.05822600	-3.38623800	-4.72190800
C	0.08112800	-4.22315600	-3.60877900
C	0.27822700	-3.69134600	-2.34048700
C	-2.49388300	2.01511800	1.85912300
C	-2.85044400	3.15473500	1.12281600
C	-3.47356000	4.23425300	1.73737900
C	-3.76495100	4.19244300	3.09676100
C	-3.43099200	3.06310800	3.83690400
C	-2.79446400	1.98654000	3.22918000
C	-1.21117000	-0.45053900	2.53996700
C	-2.22149100	-1.27113400	3.05461800
C	-1.97506300	-2.05883700	4.17250700
C	-0.72982300	-2.01920600	4.79891700
C	0.26684400	-1.18366400	4.30876500
C	0.02661400	-0.40248700	3.18164800
C	0.87683400	2.76198900	0.79300000
C	-0.66756700	2.52996400	-1.24913200
C	-0.41159400	3.80650500	-0.87572400
C	0.43455400	3.93055400	0.27767800
C	1.41646300	2.64220400	2.15903600
C	2.73179600	2.29995500	2.55547700
C	3.01421700	2.19209400	3.92400800
C	2.05784700	2.43939900	4.90026000
C	0.78888600	2.85898600	4.51704300
C	0.48690900	2.96328500	3.16884700
C	3.87936200	2.17253900	1.62820100
C	5.02158500	2.95400500	1.85139500
C	6.13593200	2.83100600	1.03151500
C	6.13105000	1.92548800	-0.02207400
C	5.00139500	1.13864700	-0.26661700
C	3.89133200	1.27088900	0.57246100
C	4.98395400	0.19587900	-1.39540300
C	6.10981600	-0.56936600	-1.69959800
C	6.11900600	-1.44236900	-2.78090000
C	4.99125100	-1.59109000	-3.58925000
C	3.85573200	-0.85501500	-3.30789300
C	3.83763500	0.04739000	-2.22401400
C	2.73318300	0.90444700	-2.07321600
C	1.79187500	1.69978700	-1.90127400
C	-1.56416400	2.23328000	-2.37626600
O	-2.42501200	3.00452000	-2.75965700
O	-1.33152200	1.04294600	-2.94293700
C	-2.12366900	0.74093200	-4.09640700
C	-1.07505200	4.99960800	-1.48160100
O	-1.89833700	5.65817300	-0.89041400
O	-0.62334700	5.25119300	-2.70883100
C	-1.33079600	6.27644600	-3.40940800
H	1.26787600	-3.83886900	1.47656300
H	-3.80850100	1.54231700	-0.40845700
H	-5.66016100	-4.08415200	1.44524300
H	-4.42013300	-5.38076000	1.37821200
H	-4.43946800	-4.24906600	2.75293800

H	-3.19688200	-4.67280600	-3.40141200
H	-4.62765000	-3.61990900	-3.11843300
H	-3.24989600	-3.00485800	-4.06542000
H	-6.36331000	2.23401500	-2.80494000
H	-5.95624700	1.94161300	-1.09066500
H	-4.71523000	2.60661600	-2.21490300
H	-0.44924500	-6.87847700	4.21389300
H	-1.72769000	-5.61609100	4.20074400
H	-1.69172600	-6.85252000	2.91859700
H	2.31047600	-1.15798600	1.89057800
H	4.52236800	-1.92697500	2.65843400
H	5.90371100	-3.40411200	1.21035400
H	5.05227400	-4.08959200	-1.02065600
H	2.83947600	-3.32759000	-1.79698200
H	0.56418300	-0.40493700	-3.16842900
H	0.21239700	-1.35503400	-5.42770100
H	-0.10109500	-3.80351800	-5.71469500
H	-0.06058400	-5.29597300	-3.72754400
H	0.28102700	-4.35298800	-1.47415500
H	-2.62549600	3.23446200	0.06362900
H	-3.70351300	5.11367500	1.13975900
H	-4.24704300	5.04029500	3.57974100
H	-3.65655700	3.01831500	4.90089900
H	-2.51182200	1.13343500	3.84142200
H	-3.20049200	-1.30240300	2.57271200

H	-2.75929600	-2.71073700	4.55536300
H	-0.54183000	-2.63942400	5.67352700
H	1.23799600	-1.13528900	4.79910900
H	0.80711100	0.26079800	2.80826000
H	0.63740700	4.89484300	0.74890600
H	4.02658800	1.91639800	4.21895900
H	2.31456900	2.33439300	5.95269200
H	0.03307900	3.10856200	5.26011700
H	-0.49840700	3.30820300	2.86328000
H	5.02137100	3.67527000	2.66800100
H	7.01027700	3.45429600	1.20747400
H	6.99461200	1.85119800	-0.68203800
H	3.02881700	0.62384900	0.42831000
H	6.98841400	-0.48867000	-1.06134600
H	7.01639100	-2.02153800	-2.99228600
H	5.00508900	-2.27548900	-4.43429900
H	2.96446300	-0.94852500	-3.92670200
H	1.45266300	2.64613600	-2.30222500
H	-1.77157400	1.33053900	-4.95083200
H	-1.98795400	-0.32795400	-4.28005900
H	-3.18011300	0.96202700	-3.91801800
H	-1.30903300	7.21547900	-2.84772000
H	-0.82381300	6.38882100	-4.36890800
H	-2.37053000	5.96289900	-3.55603400

TS1a

N	-3.01040600	3.23968900	2.71153400
C	-1.88547700	3.35067900	2.02489200
C	-1.46017000	2.45860600	1.01874500
C	-2.30389300	1.37421700	0.75005300
C	-3.50063800	1.25502600	1.45317600
C	-3.79985600	2.21395500	2.42265200
C	-0.21755200	2.79927400	0.29523800
C	-0.25918700	3.94309800	-0.54465700
N	0.77214500	4.47009600	-1.16696400
C	1.96172700	3.89648900	-0.99756500
C	2.14323800	2.75636600	-0.21626300
C	1.03729600	2.20052300	0.43791500
P	1.27523600	0.54856000	1.23020900
P	-1.84306600	0.14855500	-0.56989300
Rh	0.67984100	-1.05218600	-0.47730900
O	-1.05391600	4.36106900	2.31936200
C	-1.46575100	5.27328900	3.32498900
O	-1.46454200	4.50167500	-0.71714600
C	-1.54207900	5.61729600	-1.59260700
O	2.94208200	4.49816200	-1.67466900
C	4.24797500	3.94645000	-1.60982900
O	-4.95049800	2.06296700	3.08651900
C	-5.24955500	3.01252500	4.09694700
C	-3.23021100	-1.05591600	-0.40241300
C	-3.37476300	-1.73958500	0.80938500
C	-4.50342500	-2.51326700	1.05308700
C	-5.47198300	-2.66958600	0.06630000
C	-5.30805500	-2.03965300	-1.16289600
C	-4.20456300	-1.22387400	-1.39201000
C	-2.35695400	1.04570800	-2.08774300
C	-2.02846400	0.49285400	-3.32795200
C	-2.55682500	1.01555500	-4.50148800
C	-3.39771500	2.12502400	-4.44969900
C	-3.70457100	2.70209700	-3.22148200
C	-3.19855100	2.16033400	-2.04188600

C	2.94789700	0.56275300	2.01183800
C	4.12752000	0.72468000	1.26859000
C	5.36996700	0.72039600	1.88959000
C	5.46750200	0.53471400	3.26588600
C	4.31060200	0.33990500	4.01007300
C	3.06342600	0.34746800	3.39167500
C	0.17315500	0.41082700	2.67234400
C	-0.01001500	1.47488100	3.56188000
C	-0.80166100	1.30502100	4.69065300
C	-1.39821000	0.07135300	4.95165200
C	-1.20085300	-0.99436400	4.08151400
C	-0.41656300	-0.82380600	2.94307800
C	2.39309000	-2.02856500	0.10434900
C	2.03426900	-0.21378500	-1.81862900
C	3.31086900	-0.66395200	-1.61417100
C	3.47291000	-1.70641700	-0.67361900
C	2.60978700	-2.85544600	1.30887000
C	1.73457300	-3.81176100	1.88293400
C	2.19003500	-4.58280000	2.96127000
C	3.44060800	-4.40502400	3.53190000
C	4.27357300	-3.41455100	3.03006900
C	3.85924300	-2.66594000	1.94205000
C	0.34794000	-4.05378500	1.44050900
C	-0.62202500	-4.36700100	2.40550600
C	-1.91389100	-4.69318900	2.02626800
C	-2.28371000	-4.62941900	0.68996400
C	-1.36554800	-4.24176700	-0.28980300
C	-0.03546900	-4.01615400	0.09678000
C	-1.82971200	-4.04754200	-1.67626300
C	-2.87207200	-4.83795100	-2.17239500
C	-3.38716800	-4.66296200	-3.44836400
C	-2.85001700	-3.68892600	-4.28455700
C	-1.80026300	-2.90850300	-3.83104500
C	-1.28199200	-3.06298700	-2.53202700
C	-0.11705300	-2.27644400	-2.21283200

C	1.05220200	-1.93486000	-2.54051100
C	1.83605300	0.94284400	-2.71785900
O	2.76412600	1.42097700	-3.34592000
O	0.59413700	1.40575000	-2.78667800
C	0.41103000	2.51637400	-3.67687300
C	4.51467400	-0.10673000	-2.30501700
O	5.26538300	0.69562400	-1.79402500
O	4.69374600	-0.68161800	-3.48861400
C	5.74771700	-0.12323700	-4.27884300
H	-4.21822500	0.46051800	1.28169100
H	3.12943200	2.32830000	-0.12368200
H	-0.66521600	6.01266500	3.39725400
H	-2.40940000	5.76032100	3.05386800
H	-1.60052300	4.76371400	4.28767200
H	-2.59050000	5.92537100	-1.58187200
H	-0.89873600	6.43456400	-1.24889100
H	-1.23794700	5.33857300	-2.60950200
H	4.87773400	4.60455600	-2.21174700
H	4.62294800	3.93436400	-0.57603300
H	4.27138900	2.93055800	-2.02541900
H	-6.21320900	2.70918200	4.51155400
H	-4.48118700	3.00618800	4.88049800
H	-5.31840100	4.02489200	3.68196900
H	-2.62322600	-1.62978400	1.59506700
H	-4.61818700	-3.00700400	2.01756100
H	-6.35447600	-3.27773500	0.25691500
H	-6.05520000	-2.16181600	-1.94502500
H	-4.12052800	-0.70309500	-2.34431700
H	-1.33960200	-0.34688400	-3.36928900
H	-2.30033900	0.56480600	-5.45957100
H	-3.80992600	2.54164800	-5.36688500
H	-4.35809000	3.57157300	-3.17475100
H	-3.46690000	2.61127200	-1.08839300
H	4.12062400	0.82801900	0.18844600
H	6.26053100	0.85727700	1.27813200
H	6.44062900	0.53525100	3.75344800
H	4.36988100	0.17725600	5.08456000
H	2.18059600	0.17842200	4.00311500
H	0.45998300	2.43969400	3.36483700
H	-0.95590500	2.14035000	5.37197100
H	-2.01545900	-0.05605400	5.83893300
H	-1.66103500	-1.96232200	4.27812400
H	-0.26373600	-1.66655300	2.26456600
H	4.44757200	-2.17813300	-0.53536600
H	1.54445300	-5.37052700	3.34438100
H	3.75300900	-5.03016200	4.36583800
H	5.24529300	-3.21676600	3.47897600
H	4.51218600	-1.87530300	1.58169600
H	-0.35283700	-4.35422400	3.46080800
H	-2.65108000	-4.96594200	2.78045900
H	-3.31858200	-4.81530400	0.41302100
H	0.73525800	-3.89773300	-0.66008900
H	-3.27061200	-5.63386700	-1.54615200
H	-4.19642300	-5.30139300	-3.79669500
H	-3.23154200	-3.55208600	-5.29400100
H	-1.34670600	-2.17131700	-4.49173300
H	1.95633500	-2.27448300	-3.02188500
H	0.56032000	2.19138900	-4.71252000
H	-0.61908700	2.84717400	-3.52521400
H	1.11189600	3.32299400	-3.43932800
H	6.70375400	-0.19036500	-3.75053400
H	5.77308900	-0.70764000	-5.19948000
H	5.51967900	0.92693500	-4.49336500

TS1s

N	-5.07832900	-0.65528800	1.41379400
C	-4.44448100	0.25124600	0.68690100
C	-3.17662200	0.06784100	0.10209700
C	-2.57591700	-1.17815400	0.31666900
C	-3.22722800	-2.14543500	1.07244300
C	-4.47465400	-1.81910800	1.60990500
C	-2.60403400	1.18076600	-0.68734200
C	-3.24174400	1.51227100	-1.91337700
N	-2.93221300	2.53956100	-2.67664100
C	-1.96399800	3.35401900	-2.26419300
C	-1.22324500	3.12504600	-1.10636400
C	-1.54556200	2.01643600	-0.31196900
P	-0.37581700	1.58215200	1.05415400
P	-0.94273600	-1.51079100	-0.47966100
Rh	1.17838300	0.14488700	-0.13159400
O	-5.01290000	1.45394900	0.52557900
C	-6.29716100	1.65114300	1.09546500
O	-4.21482600	0.68812100	-2.30797900
C	-4.88766600	1.00697000	-3.51759500
O	-1.75700800	4.39034000	-3.08858300
C	-0.84826900	5.40420100	-2.68840100
O	-5.06683700	-2.74497400	2.37059500
C	-6.30131900	-2.39412900	2.97575500
C	-0.54871400	-3.22077600	0.04579700
C	-0.34763700	-3.46239600	1.41449800
C	0.03741300	-4.71684900	1.86416700
C	0.23733300	-5.75270600	0.95356500
C	0.05355900	-5.52022500	-0.40283000
C	-0.32981900	-4.26069700	-0.85921700
C	-1.52063400	-1.58499400	-2.21776200
C	-1.17090900	-0.56106800	-3.09892700
C	-1.67882100	-0.54704800	-4.39507100
C	-2.52247400	-1.56660700	-4.82375000
C	-2.89322500	-2.57915900	-3.94152700
C	-2.41410400	-2.57681700	-2.63702600
C	0.23971700	3.14040200	1.82298400
C	0.98583500	4.09792700	1.11902500
C	1.47426500	5.23177000	1.75896800
C	1.24553600	5.42927900	3.11443200
C	0.52926700	4.47831200	3.83217500
C	0.03476100	3.34510200	3.19789700
C	-1.38708700	0.87332500	2.40199600
C	-2.57548000	1.49948200	2.79938200
C	-3.29865800	0.99803200	3.87362700
C	-2.83787900	-0.11871600	4.57243200
C	-1.65186200	-0.73305500	4.19185800
C	-0.93279900	-0.23891800	3.10609100
C	2.52427300	0.47187900	1.39001600
C	2.27429600	1.72436000	-0.86655900
C	3.17871300	2.27072300	-0.00169300
C	3.33443600	1.55054000	1.21632500
C	2.49836000	-0.31090700	2.63765200
C	2.89554800	-1.65960600	2.81428000
C	2.81575100	-2.21842600	4.09498700
C	2.39060200	-1.48576400	5.19573300
C	2.06142200	-0.14397300	5.03765000

C	2.11383900	0.42647500	3.77533700
C	3.48460500	-2.49408500	1.74182700
C	4.73925000	-3.09331300	1.91633500
C	5.28902100	-3.87201400	0.90454300
C	4.61342100	-4.04658900	-0.30047100
C	3.36259000	-3.45588300	-0.49486900
C	2.80857500	-2.72058600	0.55324200
C	2.65485000	-3.53690200	-1.78782800
C	2.58932900	-4.73586200	-2.49916200
C	1.93302300	-4.81895200	-3.72195600
C	1.33646000	-3.68713200	-4.27367600
C	1.40885600	-2.47686400	-3.59890000
C	2.05534600	-2.38681500	-2.36034800
C	2.25458100	-1.10232900	-1.73856500
C	3.01471500	-0.19719200	-1.31203300
C	1.85675100	2.42549700	-2.09295700
O	1.97014700	3.63140300	-2.22495600
O	1.31479800	1.63061100	-3.01947900
C	0.88296100	2.27201500	-4.22149900
C	4.03389700	3.45194100	-0.31556500
O	4.13391100	4.42686800	0.38918600
O	4.71595500	3.25141500	-1.44718200
C	5.47267600	4.37541500	-1.89958600
H	-2.81119300	-3.12819200	1.27136300
H	-0.43191500	3.80555500	-0.82523800
H	-6.57738300	2.67593300	0.84215100
H	-7.02538100	0.94513800	0.67997300
H	-6.26988400	1.52264300	2.18487800
H	-5.61907800	0.20852100	-3.66280600
H	-5.38771100	1.97926200	-3.44218800
H	-4.18583100	1.03786000	-4.35932400
H	-0.91802900	6.18323800	-3.45053200
H	-1.13438400	5.82429800	-1.71388700
H	0.18196400	5.02428900	-2.64179500
H	-6.60308500	-3.26642100	3.55936200
H	-6.17988900	-1.52158200	3.63089200
H	-7.06118700	-2.16190500	2.22066300
H	-0.49840800	-2.66249200	2.14038800
H	0.18816600	-4.88395600	2.92922600
H	0.53980500	-6.73746900	1.30417800
H	0.21597000	-6.31981900	-1.12391400
H	-0.44546500	-4.09600400	-1.92975900
H	-0.49270400	0.22993300	-2.77365200
H	-1.42034100	0.26911200	-5.06942300
H	-2.90847200	-1.56346200	-5.84176100
H	-3.57167200	-3.36591200	-4.26584800
H	-2.74046000	-3.35028900	-1.94168000
H	1.22692300	3.97638200	0.06635000
H	2.06668400	5.94235400	1.18822400
H	1.63554800	6.31448500	3.61273000
H	0.34938600	4.61409400	4.89700200
H	-0.51368600	2.61776100	3.79146200
H	-2.94108400	2.37330200	2.25774700
H	-4.22745200	1.48224800	4.17127700
H	-3.40630600	-0.50352000	5.41727300
H	-1.27933100	-1.60156400	4.73352200
H	-0.00048400	-0.71956400	2.81459900
H	4.07260800	1.83978600	1.96746800
H	3.11208100	-3.26003200	4.21839700
H	2.34004300	-1.95636800	6.17580300
H	1.75972800	0.45868600	5.89214300
H	1.84993100	1.47642700	3.64568900
H	5.29094000	-2.92614700	2.84117900
H	6.26799800	-4.32623500	1.04471500
H	5.07483800	-4.61478900	-1.10785700
H	1.80849600	-2.31319600	0.44531800
H	3.04139100	-5.62632900	-2.06210600
H	1.88898800	-5.77105000	-4.24774500
H	0.82796700	-3.74137800	-5.23417800
H	0.98309400	-1.57513900	-4.03440400
H	4.03011200	0.12079500	-1.13529000
H	1.65924700	2.94404700	-4.59930400
H	0.69941800	1.46467000	-4.93498200
H	-0.03851500	2.84265200	-4.04611900
H	6.17308900	4.70678300	-1.12667700
H	6.00856000	4.03852100	-2.78816800
H	4.79232100	5.19775000	-2.14875700

IM2a

N	3.24958300	-3.41909300	1.92111300
C	2.12214800	-3.51721600	1.23567000
C	1.61364200	-2.53067800	0.36457500
C	2.37753200	-1.36167500	0.23486500
C	3.58629200	-1.26521900	0.91944100
C	3.96693500	-2.31788600	1.75302500
C	0.38109000	-2.87649600	-0.37914500
C	0.47313200	-3.87490400	-1.38030400
N	-0.54721500	-4.38592000	-2.03946300
C	-1.76886700	-3.98176300	-1.70547100
C	-2.00895000	-3.04174400	-0.70515700
C	-0.91635400	-2.46727600	-0.05364500
P	-1.20893500	-1.04287300	1.08332600
P	1.75797400	0.05016600	-0.80244900
Rh	-0.58403200	1.09948000	-0.03118400
O	1.37785200	-4.62168000	1.38798000
C	1.88922100	-5.64101300	2.23093100
O	1.70569300	-4.31182400	-1.67400600
C	1.82268100	-5.25023700	-2.73312400
O	-2.73717200	-4.54320700	-2.44196600
C	-4.07546100	-4.14478900	-2.20437500
O	5.11844800	-2.17634200	2.41825000
C	5.49622400	-3.22677600	3.29346300
C	3.09350400	1.29988200	-0.57055400
C	3.37182800	1.75585300	0.72231100
C	4.46773200	2.57778100	0.95928900
C	5.24751000	3.02964300	-0.10014200
C	4.93575800	2.63759300	-1.39709000
C	3.88312200	1.75891700	-1.62947900
C	2.10400100	-0.53159900	-2.50894200
C	1.69643900	0.25988400	-3.58852300
C	2.05582300	-0.07870400	-4.88710000
C	2.81579200	-1.22184600	-5.12476500
C	3.22436600	-2.01380200	-4.05730800
C	2.88201000	-1.66641400	-2.75252000
C	-2.95267600	-1.23320900	1.63870400
C	-4.00171700	-1.00867700	0.73213300
C	-5.32622300	-1.08819000	1.14652900
C	-5.62723200	-1.38889400	2.47364300
C	-4.59575600	-1.60145300	3.38107000
C	-3.26739100	-1.51564900	2.97088100
C	-0.20110700	-1.35379700	2.57079000

C	-0.17288000	-2.60076300	3.20601800
C	0.63729400	-2.79345500	4.31854400
C	1.42936800	-1.75187800	4.79971000
C	1.39854900	-0.50774200	4.17837200
C	0.57800700	-0.30917000	3.07247300
C	-2.17793600	1.90817300	0.82905800
C	-1.85735300	1.11445200	-1.73068100
C	-3.16955700	1.62243900	-1.28972000
C	-3.29342400	2.09448900	-0.02344700
C	-2.39778300	2.19397900	2.24876900
C	-1.47823100	2.83336900	3.12002900
C	-1.83873700	3.01560800	4.45773000
C	-3.05015900	2.56186800	4.96151400
C	-3.95485600	1.93062600	4.11615500
C	-3.63779700	1.77418500	2.77778000
C	-0.19309300	3.37146400	2.64995900
C	0.90206500	3.46136200	3.51979900
C	2.08216900	4.05285500	3.09345600
C	2.22454500	4.45171500	1.77076600
C	1.18437100	4.28281000	0.85068000
C	-0.04678600	3.83495200	1.33737200
C	1.42040800	4.50939300	-0.58576000
C	2.30996500	5.52379200	-0.96271000
C	2.66617100	5.74171600	-2.28384500
C	2.13204500	4.92703400	-3.27637100
C	1.23963800	3.92731900	-2.92741400
C	0.84575000	3.69395800	-1.59540700
C	-0.14965100	2.63039900	-1.39378600
C	-1.07458200	2.26678700	-2.29489100
C	-2.00944800	-0.16684100	-2.49420800
O	-3.02410800	-0.83641100	-2.41866600
O	-0.95822700	-0.46038200	-3.23963400
C	-0.96521500	-1.66963000	-4.00550400
C	-4.35997400	1.60463100	-2.19046000
O	-5.48693000	1.45198500	-1.78549400
O	-4.01298400	1.79157400	-3.46505900
C	-5.09275300	1.72279800	-4.40161800
H	4.24951800	-0.41190300	0.84145800
H	-3.02076100	-2.75376000	-0.45807300
H	1.15021300	-6.44498100	2.20226100
H	2.85788200	-6.00384200	1.86816000
H	2.01704100	-5.27562700	3.25788700
H	2.88982700	-5.47352000	-2.81011200
H	1.25492900	-6.16212500	-2.51866400
H	1.45351800	-4.82352400	-3.67479600
H	-4.68397600	-4.69539400	-2.92493300
H	-4.39575000	-4.40990700	-1.18662500
H	-4.19857400	-3.06402700	-2.36206100
H	6.44278500	-2.91399300	3.73938700
H	4.73967700	-3.37716900	4.07404400
H	5.62858900	-4.16866100	2.74814000
H	2.75963800	1.42870900	1.56716700
H	4.70368600	2.87737400	1.97942400
H	6.09845900	3.68243300	0.08604300
H	5.53140400	2.99313900	-2.23583600
H	3.68982800	1.42457900	-2.64665300
H	1.08908200	1.14427000	-3.40562800
H	1.73862100	0.55030700	-5.71741600
H	3.09523400	-1.48976700	-6.14212800
H	3.82923500	-2.90185500	-4.23396900
H	3.22267400	-2.28918600	-1.92831300
H	-3.79801400	-0.77248100	-0.31185900
H	-6.11915800	-0.89947500	0.42451200
H	-6.66419700	-1.45313100	2.79830600
H	-4.82006700	-1.82911000	4.42178500
H	-2.47671600	-1.66101300	3.70433400
H	-0.76792700	-3.42627200	2.81341700
H	0.65425200	-3.76350300	4.81308200
H	2.07032300	-1.91286500	5.66476800
H	2.01666200	0.30975300	4.54712500
H	0.55427000	0.67150200	2.58700400
H	-4.21536400	2.57550700	0.30725400
H	-1.16816200	3.56831200	5.11242100
H	-3.29365400	2.72303500	6.00985800
H	-4.91047800	1.56803300	4.48941400
H	-4.34801100	1.26761300	2.12654100
H	0.82282300	3.07379300	4.53461700
H	2.91601500	4.16399800	3.78522400
H	3.18000100	4.84153600	1.42808900
H	-0.91724400	3.91264200	0.69219400
H	2.71211300	6.17875000	-0.19127400
H	3.35550500	6.54478400	-2.53665600
H	2.40375100	5.07318100	-4.32001400
H	0.82761400	3.29366300	-3.71144100
H	-1.35525800	2.74225700	-3.24192600
H	-0.94870500	-1.39504100	-5.06453200
H	-0.05255100	-2.21971400	-3.75153500
H	-1.85080600	-2.26959500	-3.78003900
H	-5.85702900	2.46694800	-4.15731900
H	-4.65393400	1.92657500	-5.37911800
H	-5.54125000	0.72409100	-4.37788400

IM2s

N	-5.00459800	-0.84976500	1.50690500
C	-4.42438400	0.14260100	0.84983000
C	-3.19373600	0.04496300	0.17381300
C	-2.57726000	-1.21098900	0.18985000
C	-3.16782800	-2.26402000	0.87918800
C	-4.37592000	-2.01661300	1.53629400
C	-2.65036300	1.25641100	-0.47561900
C	-3.31960400	1.76960100	-1.61544700
N	-3.03207600	2.90798600	-2.21214500
C	-2.06299700	3.65740000	-1.69357500
C	-1.29541800	3.25978300	-0.59973700
C	-1.58973300	2.03257000	0.00617000
P	-0.37730100	1.41489500	1.25680700
P	-1.01251500	-1.42248400	-0.77282500
Rh	1.20932000	-0.03590500	-0.02497900
O	-5.01278600	1.34755000	0.86079400
C	-6.24497700	1.46520600	1.55336400
O	-4.30383000	1.01308200	-2.10861300
C	-4.98523200	1.50017500	-3.25507100
O	-1.88361800	4.81278600	-2.35077300
C	-0.93548200	5.73682000	-1.84540100
O	-4.90755500	-3.03017000	2.22770500
C	-6.12044800	-2.77888100	2.91852200
C	-0.69328700	-3.21804500	-0.59539800
C	-0.31915000	-3.70285500	0.66543800
C	-0.06326900	-5.05467900	0.85658100
C	-0.17160500	-5.94004400	-0.21270900
C	-0.53886000	-5.46690500	-1.46731200

C	-0.80109700	-4.11387400	-1.66059700	H	-6.55197800	2.50649900	1.43342800
C	-1.67784700	-1.19685400	-2.46282900	H	-7.00216500	0.79520600	1.13032200
C	-1.02449000	-0.34848600	-3.35518400	H	-6.12344300	1.22537700	2.61783300
C	-1.54389900	-0.13724300	-4.62947800	H	-5.72639300	0.73545100	-3.50041800
C	-2.71717400	-0.77588400	-5.01762700	H	-5.47527300	2.45718700	-3.04303400
C	-3.38005800	-1.61695200	-4.12602100	H	-4.29086500	1.63816400	-4.09265200
C	-2.87038400	-1.81990400	-2.84991500	H	-1.00165200	6.61517200	-2.49132900
C	0.28393700	2.90157500	2.11652500	H	-1.18026100	6.02826700	-0.81423200
C	1.25423300	3.69531600	1.48848700	H	0.08081000	5.32186700	-1.88071700
C	1.86399000	4.74306100	2.16931500	H	-6.37306200	-3.71402100	3.42285300
C	1.51131700	5.01775100	3.48759900	H	-5.99478700	-1.97304900	3.65358400
C	0.54241200	4.24296200	4.11738400	H	-6.92000600	-2.49482400	2.22430700
C	-0.06324500	3.18766100	3.44236700	H	-0.24761900	-3.02067300	1.51699200
C	-1.29966400	0.55097500	2.57602300	H	0.22152400	-5.41701600	1.84261400
C	-2.53027200	1.02285500	3.04601600	H	0.02942800	-6.99950200	-0.06526900
C	-3.18639600	0.35321400	4.07195100	H	-0.62059200	-6.15312700	-2.30866000
C	-2.61546500	-0.78011200	4.64958900	H	-1.07817300	-3.75667100	-2.65113600
C	-1.38185000	-1.23950800	4.20346500	H	-0.10603200	0.15296700	-3.05270700
C	-0.72847200	-0.57659400	3.16863200	H	-1.02741300	0.52511300	-5.32297400
C	2.65622400	0.40153000	1.19249700	H	-3.12143800	-0.61497300	-6.01571500
C	2.33684000	1.32755000	-1.30675600	H	-4.30351900	-2.11059700	-4.42371300
C	3.39326500	1.89168300	-0.45910300	H	-3.40401500	-2.46196100	-2.14839300
C	3.60510600	1.35303400	0.77608400	H	1.55019000	3.50473200	0.45906700
C	2.68298400	-0.09750600	2.57766600	H	2.62878500	5.32996100	1.66389600
C	2.93698500	-1.43200800	2.97914400	H	1.99357400	5.83252600	4.02438200
C	2.87901100	-1.74367100	4.34093700	H	0.25882800	4.45235400	5.14733500
C	2.58214400	-0.78257900	5.29865100	H	-0.79055200	2.57308300	3.96843200
C	2.40064700	0.54185300	4.91082700	H	-2.98637300	1.90519300	2.59326300
C	2.47491000	0.87978400	3.56885700	H	-4.15240400	0.71551800	4.42122500
C	3.42527000	-2.43815900	2.01884500	H	-3.13444400	-1.30110300	5.45219100
C	4.62270600	-3.12046100	2.26602700	H	-0.92205400	-2.11646800	4.65635300
C	5.15706600	-3.95854500	1.29571700	H	0.24295500	-0.93811200	2.82707400
C	4.53368500	-4.09021400	0.05893700	H	4.44237600	1.65781100	1.40691500
C	3.33116700	-3.42778500	-0.20526700	H	3.07934800	-2.77122700	4.64330900
C	2.77086800	-2.65904100	0.81342200	H	2.52415900	-1.06195000	6.34878300
C	2.70929400	-3.48562200	-1.54123100	H	2.21335800	1.31814100	5.65045300
C	2.71008800	-4.69079800	-2.24736900	H	2.35891800	1.91948100	3.26840900
C	2.22496200	-4.77680300	-3.54608000	H	5.15431500	-2.95304000	3.20218800
C	1.73332900	-3.63725800	-4.17303400	H	6.09511200	-4.47654100	1.48559200
C	1.72678200	-2.42862500	-3.49014400	H	5.00243800	-4.68341900	-0.72522700
C	2.20454900	-2.32455000	-2.17356200	H	1.77728100	-2.23429000	0.66863300
C	2.22515100	-0.98191800	-1.58533100	H	3.08810700	-5.58449700	-1.75125800
C	2.75921900	0.11792900	-2.10461100	H	2.23267600	-5.73294200	-4.06639200
C	1.64495500	2.39946000	-2.09154300	H	1.36164300	-3.68436200	-5.19491400
O	1.59356200	3.55975300	-1.72750300	H	1.34960400	-1.53065000	-3.97882600
O	1.14161900	1.96443800	-3.24328900	H	3.39824400	0.22187700	-2.98945100
C	0.50763000	2.94364500	-4.07308100	H	1.11446900	3.85322100	-4.11954600
C	4.21008900	3.05836500	-0.91055200	H	0.42770900	2.48710300	-5.06158700
O	4.61037500	3.92032200	-0.16705800	H	-0.48863400	3.18680100	-3.68677900
O	4.44222600	2.99527100	-2.22397500	H	6.09990500	4.25583300	-2.26069700
C	5.14046400	4.11894100	-2.76865900	H	5.29122300	3.89439600	-3.82532100
H	-2.74028200	-3.26053900	0.92826000	H	4.53436900	5.02329400	-2.64542800
H	-0.49180100	3.88654800	-0.23325700				

TS2

N	-3.69415200	-3.28385300	1.56336400	C	-3.73673700	-0.17061700	-1.60110200
C	-3.76248600	-2.15696200	0.87163400	N	-4.14914000	0.95790300	-2.14680400
C	-2.68624000	-1.58149600	0.17072500	C	-3.79049400	2.10112700	-1.56729100
C	-1.48884900	-2.30434900	0.17920800	C	-2.94032400	2.15927100	-0.46311500
C	-1.39581800	-3.49031200	0.89850400	C	-2.47028600	0.95799000	0.07383800
C	-2.52907300	-3.91633200	1.59698300	P	-1.07728200	1.05402800	1.28139500
C	-2.89914300	-0.26223600	-0.46412600	P	-0.09096800	-1.66459400	-0.83914900

Rh	0.98754700	0.61958700	-0.07673100
O	-4.91813600	-1.47748200	0.86534300
C	-6.00960900	-2.04162600	1.57455000
O	-4.10825500	-1.32996300	-2.15167900
C	-4.85183400	-1.26586800	-3.35896700
O	-4.29196600	3.18425500	-2.17614800
C	-4.01619300	4.45727900	-1.61813700
O	-2.40788700	-5.02620300	2.33207400
C	-3.53715500	-5.42589300	3.09183100
C	1.20223800	-2.93302200	-0.57855100
C	1.79445600	-3.01969500	0.68809800
C	2.76202800	-3.98096200	0.94956100
C	3.16684100	-4.85072900	-0.06017700
C	2.59937800	-4.75572100	-1.32628600
C	1.61747300	-3.80401500	-1.58696400
C	-0.76143300	-1.99733000	-2.51003300
C	-0.58620600	-1.06917600	-3.53447200
C	-1.10634200	-1.31757000	-4.80279700
C	-1.80117600	-2.49635400	-5.05149400
C	-1.98225300	-3.42625200	-4.02949300
C	-1.47198400	-3.17736200	-2.76214900
C	-1.22154800	2.67834500	2.13087300
C	-0.86565800	3.85447100	1.45443400
C	-0.83651900	5.07551300	2.11802000
C	-1.17171400	5.14339200	3.46788200
C	-1.53949000	3.98551400	4.14573500
C	-1.55591300	2.75899300	3.48788600
C	-1.39355400	-0.14068500	2.62430600
C	-2.68356000	-0.39317900	3.10232200
C	-2.87265000	-1.28453400	4.15224400
C	-1.77926400	-1.91572300	4.74355000
C	-0.49279000	-1.64828500	4.28889000
C	-0.30162300	-0.76393700	3.23169600
C	2.05332900	1.78802600	1.08282000
C	1.16333500	2.32549600	-1.36968400
C	1.76934300	3.41019400	-0.58028600
C	2.29494600	3.10516900	0.63823500
C	2.43730100	1.41465000	2.45320500
C	3.25881200	0.34098500	2.91586200
C	3.27146900	0.10516100	4.29756800
C	2.58412200	0.89310400	5.21026200
C	1.87284700	1.99924800	4.76519100
C	1.82010000	2.25514300	3.40551800
C	4.13758700	-0.49539000	2.06018000
C	5.17933900	-1.27254200	2.58419300
C	5.93489000	-2.07235800	1.73527900
C	5.66537400	-2.14593500	0.37440000
C	4.64068200	-1.37220000	-0.19095600
C	3.95189900	-0.53873400	0.68382700
C	4.23628100	-1.46515700	-1.61924300
C	4.91279100	-2.38240300	-2.43570800
C	4.59604800	-2.59741600	-3.76800300
C	3.56279500	-1.87620500	-4.34942300
C	2.88362800	-0.94781900	-3.57807400
C	3.18541900	-0.71259800	-2.22288200
C	2.38230800	0.37379200	-1.63613100
C	2.19493900	1.57691400	-2.17556900
C	-0.06490900	2.77798600	-2.10012400
O	-0.68862100	3.77273200	-1.78121300
O	-0.36098700	1.99654000	-3.13182400

C	-1.52298400	2.29562300	-3.91573100
C	1.80686900	4.81728600	-1.08265300
O	1.67510700	5.77728400	-0.36317300
O	2.01798900	4.85294100	-2.39811300
C	1.99305800	6.16074700	-2.98001700
H	-0.49088500	-4.08887800	0.94462400
H	-2.63838200	3.11168200	-0.04573900
H	-6.83890100	-1.34321000	1.44308700
H	-6.27540500	-3.02586600	1.17214200
H	-5.77183600	-2.15398100	2.64028000
H	-5.06313400	-2.30355300	-3.62837900
H	-5.78281100	-0.70495900	-3.22247100
H	-4.26084900	-0.78538600	-4.15061500
H	-4.54758500	5.17867300	-2.24268600
H	-4.39161800	4.52677500	-0.58759000
H	-2.93865300	4.67272400	-1.63575000
H	-3.22609300	-6.32014300	3.63611700
H	-3.83353400	-4.63597900	3.79483000
H	-4.39091000	-5.65514500	2.44353200
H	1.47728000	-2.34587900	1.48869200
H	3.21232700	-4.04029200	1.93900700
H	3.92982000	-5.60063900	0.14152200
H	2.92131800	-5.42524900	-2.12212100
H	1.18524900	-3.73445300	-2.58437300
H	-0.05018800	-0.14399000	-3.33390500
H	-0.96348400	-0.58789100	-5.59901700
H	-2.20547300	-2.69266900	-6.04314700
H	-2.52782400	-4.34878300	-4.21995200
H	-1.62790200	-3.90351400	-1.96406100
H	-0.60677100	3.83366800	0.39783800
H	-0.53227000	5.96711700	1.57253500
H	-1.14501100	6.09757700	3.99078600
H	-1.80802500	4.03075400	5.19983700
H	-1.81287300	1.86142600	4.04707100
H	-3.54299300	0.09683900	2.64110200
H	-3.88028300	-1.48920600	4.51162800
H	-1.93364700	-2.61355900	5.56464500
H	0.36710800	-2.12861600	4.75299300
H	0.71144800	-0.55002400	2.88477000
H	2.86296100	3.83085100	1.22366800
H	3.84557500	-0.73518400	4.67905500
H	2.62924900	0.65218000	6.27050700
H	1.35846800	2.65877400	5.46090500
H	1.24975300	3.11235000	3.05052800
H	5.44039700	-1.23751700	3.63921500
H	6.75418000	-2.65921200	2.14663400
H	6.27305700	-2.81578300	-0.22795900
H	3.20208300	0.10429100	0.26095900
H	5.72459200	-2.96966000	-2.01631700
H	5.15873500	-3.32879200	-4.34498100
H	3.29183800	-2.02307700	-5.39295900
H	2.07850900	-0.36916900	-4.02753700
H	2.68043300	2.02814800	-3.04866300
H	-1.97294500	3.23944300	-3.59822700
H	-1.19995500	2.35612700	-4.95890700
H	-2.24061200	1.47637700	-3.78785300
H	2.73828600	6.80448300	-2.50300500
H	2.22205000	6.02166300	-4.03715500
H	0.99868500	6.60161600	-2.85205100

TS3a

N	5.16614700	-0.35964700	-1.24061900	C	-2.69682800	-3.71604000	-0.17797000
C	4.32299100	0.59806500	-0.90370200	C	-3.24971300	-2.60575400	-0.80111400
C	3.07687000	0.40345700	-0.25432500	C	-2.84187500	-3.77122700	1.28072600
C	2.72330900	-0.92850100	-0.01986700	C	-2.94779000	-4.99438800	1.94573100
C	3.61886900	-1.94895500	-0.34388500	C	-3.15905200	-5.06417100	3.31565100
C	4.83222200	-1.60855100	-0.93422300	C	-3.30030100	-3.88497100	4.04153400
C	2.38120300	1.63949300	0.18047500	C	-3.18552100	-2.65835100	3.40372300
C	3.07770200	2.43010500	1.12899800	C	-2.90358000	-2.56343800	2.02482600
N	2.78831200	3.66950200	1.46389200	C	-2.64731600	-1.19631800	1.55130200
C	1.77412800	4.25419500	0.83893400	C	-1.84086300	-0.36513100	2.25404800
C	1.00392400	3.60032600	-0.12360300	C	-0.50504900	1.81271300	2.46489900
C	1.27587900	2.26727200	-0.42340600	O	-0.39810800	3.00972500	2.30538700
P	-0.02066000	1.34289800	-1.36829400	O	0.27764000	1.06731600	3.24016700
P	1.06812000	-1.41584300	0.66273900	C	1.33272900	1.72585700	3.94847200
Rh	-1.11451600	-0.21717700	0.10502400	C	-3.17627200	3.01852800	2.22539500
O	4.63973100	1.86313400	-1.22548700	O	-3.65020000	3.99462900	1.68787500
C	5.89867300	2.08521800	-1.84317800	O	-2.95560500	2.89823600	3.53651100
O	4.11089200	1.83235100	1.75424300	C	-3.13460300	4.09798700	4.29146700
C	4.91365900	2.64852200	2.59750300	H	3.40707300	-2.99948200	-0.16611900
O	1.54010600	5.50614800	1.24063500	H	0.17784900	4.11692500	-0.58919300
C	0.36339300	6.14750600	0.78718100	H	5.96515400	3.16498600	-1.99295400
O	5.67844400	-2.60407800	-1.21406600	H	6.71698100	1.73814500	-1.20186500
C	6.91898600	-2.26204900	-1.81157700	H	5.96414300	1.56326200	-2.80548700
C	0.96474200	-3.07386100	-0.13332400	H	5.71801500	2.00014800	2.95266600
C	1.01094900	-3.07666400	-1.53175400	H	5.32420900	3.49848600	2.04072100
C	1.21830300	-4.25730900	-2.23099000	H	4.33491600	3.03864400	3.44343000
C	1.35339500	-5.45980800	-1.54276900	H	0.33606900	7.11581800	1.29136500
C	1.25793600	-5.47280000	-0.15429600	H	0.38341800	6.31031900	-0.30010500
C	1.06961300	-4.28624500	0.55201000	H	-0.53074300	5.56595100	1.05751700
C	1.43418400	-1.75705700	2.42037300	H	7.45166600	-3.20583000	-1.94554700
C	0.49735200	-2.45600000	3.19349800	H	6.76841500	-1.77222500	-2.78114900
C	0.71284100	-2.65130000	4.55197900	H	7.49718700	-1.59115600	-1.16511900
C	1.86632000	-2.15223500	5.15380600	H	0.95735300	-2.13683900	-2.08247700
C	2.80405700	-1.46682000	4.38905000	H	1.28284400	-4.22934700	-3.31874300
C	2.59172300	-1.26557400	3.02725500	H	1.53547900	-6.38568700	-2.08571200
C	-1.08970700	2.63706600	-2.11185300	H	1.36405000	-6.40896500	0.39135400
C	-1.87090600	3.45401300	-1.28085900	H	1.06058400	-4.31073700	1.64018400
C	-2.67491000	4.45229600	-1.81598200	H	-0.41062900	-2.85678200	2.73635500
C	-2.72873500	4.63405200	-3.19643300	H	-0.02484600	-3.19798800	5.13748200
C	-1.97441600	3.81575500	-4.03072300	H	2.03627100	-2.30566600	6.21785600
C	-1.15619600	2.82403300	-3.49519800	H	3.71114600	-1.08197500	4.85272700
C	0.76467200	0.54768300	-2.80735600	H	3.32874700	-0.71222200	2.44351200
C	2.07564600	0.82213100	-3.20106900	H	-1.84314500	3.33084700	-0.19996500
C	2.64190200	0.12144800	-4.26210200	H	-3.26350000	5.07430100	-1.14300700
C	1.89118500	-0.81785900	-4.96344600	H	-3.35735000	5.41492300	-3.62100400
C	0.56436200	-1.05335100	-4.61007000	H	-2.01092000	3.95365900	-5.10986200
C	0.00722600	-0.38391700	-3.52647600	H	-0.56329300	2.20355200	-4.16493800
C	-3.03630300	0.28435200	-0.32576400	H	2.65851500	1.58115800	-2.68077900
C	-1.59755100	1.02025900	1.80818200	H	3.67419800	0.31933400	-4.54627100
C	-2.78611300	1.79648100	1.47999500	H	2.33706800	-1.35806000	-5.79646900
C	-3.59074500	1.29388500	0.51306900	H	-0.03400800	-1.77029900	-5.17104900
C	-3.61527200	0.13138100	-1.68063600	H	-1.02739900	-0.58302200	-3.23721700
C	-3.47467600	-0.97126400	-2.57979500	H	-4.59426800	1.69415800	0.36316400
C	-3.87916300	-0.83713000	-3.90786100	H	-3.80221700	-1.70395800	-4.56282400
C	-4.42474600	0.34240100	-4.39787300	H	-4.73006500	0.41143600	-5.43998800
C	-4.60121800	1.41391600	-3.53375700	H	-5.03455900	2.34832200	-3.88448700
C	-4.21451400	1.29988200	-2.20735200	H	-4.32324700	2.17905700	-1.57977400
C	-2.98434700	-2.27585300	-2.11871400	H	-2.08003400	-3.00085000	-3.95421800
C	-2.30022200	-3.21029200	-2.90685800	H	-1.38570300	-5.15201100	-2.95904300
C	-1.89074400	-4.41279700	-2.34044600	H	-1.61433100	-5.55482300	-0.53576800
C	-2.04528500	-4.65620200	-0.97597200	H	-3.91602200	-1.97165900	-0.23228300

H	-2.89783500	-5.91138300	1.35927400
H	-3.24766200	-6.03058800	3.80725300
H	-3.50370700	-3.91669000	5.11048900
H	-3.29883100	-1.74183800	3.98123500
H	-1.25269400	-0.69802800	3.11443700
H	1.34665400	1.29657900	4.95340900
H	2.27894300	1.50615400	3.44072800
H	1.16283400	2.80562700	3.97614500
H	-4.14921500	4.48702600	4.16359700
H	-2.95342500	3.82764800	5.33270800
H	-2.41167100	4.85155400	3.95702300

TS3s

N	-4.90008700	1.05141400	-2.04313000
C	-4.51770500	0.20757900	-1.09663800
C	-3.37054800	0.37081000	-0.29045300
C	-2.61685400	1.52856500	-0.52604400
C	-3.00628500	2.42572600	-1.51545200
C	-4.15529900	2.12746500	-2.25155400
C	-3.07672000	-0.68067400	0.71263600
C	-3.93401800	-0.81952000	1.82837400
N	-3.86115500	-1.78568300	2.72415000
C	-2.96102600	-2.74726000	2.54079600
C	-2.08300800	-2.76355300	1.45503800
C	-2.12162500	-1.69792200	0.55943900
P	-0.82740100	-1.55412200	-0.74919400
P	-1.04450200	1.79846400	0.39065100
Rh	0.78062200	0.10670500	-0.14181500
O	-5.23930200	-0.90120600	-0.89922800
C	-6.40309800	-1.07206800	-1.69276100
O	-4.87341100	0.12459400	1.98072800
C	-5.65920700	0.06889800	3.16254800
O	-2.96316900	-3.67168400	3.50610800
C	-1.99808200	-4.70644900	3.44789100
O	-4.49195200	2.97939900	-3.22489600
C	-5.63974500	2.66291800	-3.99756100
C	-0.33137400	3.24561100	-0.47578900
C	0.53468600	2.99078100	-1.54556800
C	1.12543500	4.03252600	-2.24996300
C	0.85105800	5.34841100	-1.88647100
C	-0.01464500	5.61493600	-0.82906400
C	-0.60737400	4.56958800	-0.12640200
C	-1.59060400	2.46289200	2.00845600
C	-0.62248200	2.87445300	2.93245400
C	-0.99861400	3.41119600	4.15684500
C	-2.34912300	3.51951700	4.48537300
C	-3.31586900	3.10157000	3.57805500
C	-2.94222100	2.58616000	2.33877800
C	-0.07926000	-3.21189400	-0.99794700
C	0.64042400	-3.82657300	0.03727000
C	1.34254300	-5.00537100	-0.18716600
C	1.34610000	-5.58533700	-1.45110500
C	0.64329300	-4.98185900	-2.48867100
C	-0.06047200	-3.80354400	-2.26802000
C	-1.83684500	-1.31614500	-2.26475900
C	-2.85047100	-2.24562300	-2.53329200
C	-3.62050600	-2.12870400	-3.68303300
C	-3.40129600	-1.07351000	-4.56669400
C	-2.40588500	-0.13889800	-4.30121000
C	-1.62407600	-0.26271400	-3.15571800
C	2.47046300	-0.73163500	-0.81153500
C	1.60088300	-0.65103300	1.66686800
C	2.57182400	-1.70940800	1.32283200
C	3.10517100	-1.65655900	0.08541400
C	2.81827700	-0.86555600	-2.24707800
C	4.16047200	-0.94700800	-2.71790400
C	4.41821800	-1.40410900	-4.00458500
C	3.37613700	-1.73400900	-4.86855700
C	2.06084100	-1.56852100	-4.45276700
C	1.79286100	-1.13396400	-3.15978300
C	5.19145000	-0.34372100	-1.85933900
C	6.36870300	-0.93424300	-1.39322000
C	7.11349400	-0.28360500	-0.41069500
C	6.66670100	0.91205100	0.15690800
C	5.49755900	1.50805500	-0.31845400
C	4.83866800	0.90766000	-1.37809400
C	4.78918600	2.65624900	0.26950300
C	5.42607400	3.87445100	0.49603100
C	4.71520500	4.98191900	0.94362900
C	3.34720600	4.87404700	1.17590400
C	2.70930000	3.65420800	1.00087700
C	3.40427400	2.51728400	0.56159100
C	2.77818400	1.18685500	0.61868600
C	2.21816700	0.69886100	1.73729900
C	0.59581500	-1.04135600	2.70822100
O	0.32390900	-2.19716400	2.96371000
O	0.03649100	0.01053100	3.29457000
C	-1.00747700	-0.25189600	4.23913300
C	3.08007200	-2.65345000	2.34955300
O	3.40325100	-3.79573200	2.12404000
O	3.16397300	-2.05552200	3.54379100
C	3.57004800	-2.90376100	4.61902000
H	-2.45245300	3.33061000	-1.74819000
H	-1.36797900	-3.56630600	1.33462200
H	-6.83679500	-2.02355900	-1.37680600
H	-7.11597300	-0.25647600	-1.52530800
H	-6.14898500	-1.10232000	-2.76012600
H	-6.35538800	0.90836700	3.09131900
H	-6.20629800	-0.87731600	3.23149700
H	-5.02571700	0.17230300	4.05336400
H	-2.15522400	-5.31220800	4.34285100
H	-2.14264500	-5.33636400	2.55873300
H	-0.97962200	-4.29503900	3.45127400
H	-5.72362900	3.45841100	-4.74099700
H	-5.52331700	1.68985600	-4.49152100
H	-6.54026300	2.63229400	-3.37308500
H	0.76934900	1.95606300	-1.83650200
H	1.80371200	3.81933300	-3.07404300
H	1.31495600	6.16918400	-2.42987100
H	-0.23341700	6.64434300	-0.55113600
H	-1.28649400	4.78251900	0.69963300
H	0.43623800	2.76703400	2.69869800
H	-0.23551400	3.73932600	4.86046400
H	-2.64465300	3.93301900	5.44784100
H	-4.37295300	3.18920700	3.82422700
H	-3.71266500	2.27023000	1.63846700
H	0.67111900	-3.38541200	1.03195700
H	1.91001900	-5.44448900	0.63070900
H	1.90188200	-6.50395000	-1.62941300
H	0.64362100	-5.42635400	-3.48225300
H	-0.59142300	-3.34427700	-3.09981800

H	-3.03646800	-3.06460000	-1.83741500	H	3.95520000	1.39728000	-1.78449200
H	-4.39830200	-2.86255100	-3.88825000	H	6.49125100	3.95517900	0.28007500
H	-4.00973900	-0.98038800	-5.46476500	H	5.22524600	5.93138600	1.09446900
H	-2.24167600	0.69761800	-4.97845600	H	2.77419300	5.73800800	1.50869800
H	-0.85897600	0.48695200	-2.94794900	H	1.64289400	3.57697900	1.20239100
H	3.92849900	-2.30434300	-0.22018600	H	2.18288800	1.23162800	2.69557300
H	5.45229100	-1.44943200	-4.34591900	H	-0.74380300	-1.09846400	4.87979900
H	3.59486700	-2.08404900	-5.87543600	H	-1.11919700	0.66683500	4.81926600
H	1.23196300	-1.79098600	-5.12376600	H	-1.94151900	-0.47156900	3.70691100
H	0.76109100	-1.05771400	-2.83298200	H	4.54539000	-3.35252700	4.40698100
H	6.67407300	-1.91472900	-1.75915600	H	3.62358400	-2.26471100	5.50158600
H	8.02725000	-0.74334300	-0.03882800	H	2.83136300	-3.69995700	4.76317500
H	7.20722500	1.35800000	0.99173000				

IM3a

N	-5.26939500	1.37524800	0.03418200	C	3.04013700	-0.43012900	-0.81037500
C	-4.55537300	0.27158100	0.16364000	C	1.72491000	-1.60536500	1.06834000
C	-3.17250200	0.21864700	0.47014500	C	2.66484600	-2.50181100	0.33585700
C	-2.53024100	1.45907800	0.58423500	C	3.39343000	-1.87314800	-0.58161000
C	-3.27990000	2.63069500	0.47511900	C	2.97290700	-0.07104000	-2.27583300
C	-4.64540600	2.53182800	0.21933200	C	2.86450100	1.20232600	-2.93339200
C	-2.62754100	-1.14432400	0.69007600	C	2.61131400	1.27452800	-4.30613500
C	-3.18361600	-1.87291600	1.77105400	C	2.46945500	0.14214400	-5.09154300
N	-3.05069400	-3.16668000	1.98024400	C	2.59844200	-1.10546200	-4.48884000
C	-2.35729500	-3.87047000	1.09281200	C	2.84807000	-1.19803400	-3.13156900
C	-1.75380500	-3.28695400	-0.02154200	C	3.04926100	2.44375300	-2.18546900
C	-1.85633100	-1.90935900	-0.20309600	C	2.29427700	3.61399700	-2.30736500
P	-0.69313500	-1.16777900	-1.42114100	C	2.41861400	4.60965000	-1.34027900
P	-0.68896400	1.61092800	0.78948800	C	3.15264600	4.38357900	-0.17526000
Rh	1.05320700	0.01346900	-0.22023800	C	3.89973800	3.21185000	-0.06961600
O	-5.16740000	-0.90473000	-0.05213400	C	3.96221700	2.36109300	-1.15760900
C	-6.55928400	-0.87707200	-0.32960300	C	4.44176100	2.58085600	1.13170200
O	-3.88512800	-1.15695600	2.66968900	C	5.22602500	3.25034800	2.06797000
C	-4.58314900	-1.88361800	3.67241600	C	5.79953200	2.57308300	3.13697500
O	-2.26109500	-5.16909400	1.38617900	C	5.59069500	1.20531800	3.26772600
C	-1.41595900	-5.97783800	0.58763800	C	4.77085900	0.53768000	2.36486100
O	-5.33641900	3.67319200	0.14303800	C	4.15545100	1.20063600	1.28816100
C	-6.72800700	3.58006700	-0.11662300	C	3.16542900	0.31349300	0.54390200
C	-0.49848300	3.35066500	0.22366600	C	2.32188200	-0.34911800	1.46326800
C	-0.77263700	3.59447000	-1.12569800	C	0.73463400	-2.27101200	1.95621000
C	-0.90989700	4.89239500	-1.59868900	O	0.42892000	-3.43836300	1.82375600
C	-0.74149700	5.96934600	-0.73124400	O	0.27580400	-1.45578800	2.90522600
C	-0.42590200	5.73666300	0.60437800	C	-0.61663000	-1.99455700	3.88456700
C	-0.31447500	4.43361700	1.08516600	C	2.90345700	-3.90952600	0.75018700
C	-0.44466800	1.69626100	2.59591100	O	2.84937400	-4.85392800	-0.00442400
C	0.81552600	2.07587800	3.07795400	O	3.19733400	-3.98728600	2.04996600
C	1.07255300	2.08333100	4.44330400	C	3.23923200	-5.31372600	2.58013400
C	0.07331400	1.71024100	5.34074000	H	-2.84999600	3.62226400	0.57397300
C	-1.18213500	1.34458700	4.86723200	H	-1.17330400	-3.89462300	-0.70089900
C	-1.44497600	1.33484100	3.49914100	H	-6.85418900	-1.92155400	-0.45190500
C	-0.19360200	-2.53065800	-2.53923700	H	-7.11544500	-0.41866100	0.49622500
C	0.66483000	-3.52835800	-2.06183900	H	-6.76879100	-0.31348600	-1.24680800
C	1.09015700	-4.55985000	-2.88883400	H	-5.13451100	-1.13497200	4.24606800
C	0.66431700	-4.60150800	-4.21493600	H	-5.27361500	-2.60504100	3.22085800
C	-0.19118600	-3.61611700	-4.69955800	H	-3.89125100	-2.43054300	4.32406100
C	-0.61853400	-2.58238200	-3.87049200	H	-1.44311400	-6.97267000	1.03726500
C	-1.59186800	-0.03660900	-2.52665100	H	-1.78277000	-6.04237800	-0.44682700
C	-2.98260700	-0.02655500	-2.65394700	H	-0.38364600	-5.59771000	0.59233000
C	-3.58972100	0.87644400	-3.52160600	H	-7.09675100	4.60791700	-0.12025000
C	-2.81544300	1.73826800	-4.29533800	H	-6.91729700	3.10764200	-1.08819700
C	-1.42619200	1.69693100	-4.20697700	H	-7.23697300	2.99881600	0.66138700
C	-0.81755500	0.82214000	-3.31365400	H	-0.94576400	2.75667900	-1.80207300

H	-1.15740100	5.06006100	-2.64684500	H	2.56608900	2.26380000	-4.76129400
H	-0.86002800	6.98892200	-1.09400000	H	2.28891700	0.23031800	-6.16087900
H	-0.29640200	6.57396200	1.28796200	H	2.50477400	-2.01903200	-5.07268100
H	-0.12422500	4.26858300	2.14461200	H	2.90904000	-2.19935600	-2.71446500
H	1.60083200	2.37748400	2.37873500	H	1.56733300	3.72410300	-3.11381400
H	2.05461500	2.38317200	4.80606700	H	1.83284900	5.52148600	-1.43417900
H	0.27310700	1.71520800	6.41063900	H	3.08845700	5.08107100	0.66028000
H	-1.96852200	1.06385200	5.56652100	H	4.63858900	1.51202300	-1.12173700
H	-2.42960500	1.03294100	3.13925900	H	5.41422600	4.31382700	1.92247700
H	1.00539800	-3.51581700	-1.02927400	H	6.42606000	3.10597000	3.84939100
H	1.76142900	-5.31466600	-2.48270700	H	6.05540100	0.65197400	4.08120800
H	0.99539300	-5.40534800	-4.87007900	H	4.60619000	-0.53277100	2.49388600
H	-0.53411100	-3.65063800	-5.73212900	H	2.11568600	0.08550200	2.44045700
H	-1.28539400	-1.81900900	-4.26808300	H	-0.16232500	-1.82523100	4.86550600
H	-3.59230600	-0.72442900	-2.08018400	H	-1.55707900	-1.43734400	3.81553500
H	-4.67524500	0.89840000	-3.60210200	H	-0.78896800	-3.05976900	3.70777300
H	-3.29774800	2.43517100	-4.97819300	H	3.98681100	-5.91686900	2.05613400
H	-0.81292200	2.35553000	-4.82205900	H	3.49912600	-5.20796600	3.63440200
H	0.27184000	0.80826300	-3.22724100	H	2.25264000	-5.77966800	2.47127500
H	4.25356300	-2.32385200	-1.07737500				

IM3s

N	-5.53619000	0.74658400	-0.61757000	C	-0.59204600	-3.29467300	-3.20894500
C	-4.76361500	-0.04098500	0.11316100	C	-2.26632800	-1.00983300	-2.32953700
C	-3.37720400	0.14641400	0.32372200	C	-3.52179600	-1.62315700	-2.28359600
C	-2.82019600	1.27210200	-0.30306000	C	-4.46890600	-1.34174600	-3.26144000
C	-3.61677400	2.09896100	-1.08845100	C	-4.16499400	-0.46491100	-4.30111000
C	-4.96820800	1.77997200	-1.22190900	C	-2.90662400	0.12385100	-4.36783800
C	-2.67606900	-0.93392100	1.06015400	C	-1.96094700	-0.14959600	-3.38439200
C	-2.96612200	-1.16524000	2.42269600	C	2.98992700	-0.33603000	-0.60063300
N	-2.59568000	-2.22959100	3.10601500	C	1.66511900	0.13905200	1.92657200
C	-1.92220100	-3.17819600	2.46801500	C	1.92910600	-1.17928400	1.53326900
C	-1.50107000	-3.05314200	1.14284900	C	2.44735000	-1.35910500	0.23378500
C	-1.86207100	-1.89735500	0.44758700	C	3.52852600	-0.95081200	-1.87615900
P	-0.97856700	-1.45318700	-1.11043100	C	4.82655900	-1.52582700	-1.89662500
P	-1.03390100	1.69509500	-0.12550900	C	5.18339800	-2.39679000	-2.91792200
Rh	0.54355900	0.03242700	-0.14983000	C	4.28243400	-2.68903800	-3.94033300
O	-5.31230300	-1.13322700	0.65993000	C	3.02696400	-2.09472900	-3.95042100
C	-6.69126800	-1.36277800	0.41764500	C	2.64591300	-1.24483900	-2.91341900
O	-3.66741400	-0.21062400	3.05114800	C	5.71442000	-0.96373600	-0.85278100
C	-3.78953600	-0.32518000	4.46156000	C	6.10967600	-1.54547100	0.35156100
O	-1.67051600	-4.25685800	3.22997700	C	6.40323800	-0.71961100	1.44139400
C	-1.06532700	-5.38507900	2.62312300	C	6.15784300	0.65525200	1.38884500
O	-5.70272200	2.56417800	-2.01579900	C	5.77278500	1.23025000	0.17667300
C	-7.05802900	2.19597800	-2.21868100	C	5.72298000	0.42232300	-0.94861300
C	-0.69895900	2.93205900	-1.44176300	C	5.00605100	2.47566600	-0.04989900
C	0.06112500	2.55886700	-2.55398700	C	5.54800000	3.75621200	-0.04817900
C	0.35655200	3.47714200	-3.55653100	C	4.79192600	4.85113300	-0.45828000
C	-0.09264200	4.78898200	-3.44733500	C	3.48271200	4.66125400	-0.88220100
C	-0.84063700	5.17759800	-2.33806500	C	2.91570500	3.38959800	-0.85213800
C	-1.14596200	4.25631200	-1.34279600	C	3.65430000	2.27961400	-0.44246900
C	-1.02930100	2.71290200	1.40543400	C	2.94470500	0.99287000	-0.08224000
C	-0.04719300	3.69516400	1.60044700	C	2.14851200	1.17631200	1.09157800
C	-0.02960600	4.45359300	2.76518900	C	0.88951100	0.45957500	3.17256200
C	-0.99352100	4.24474700	3.74928900	O	-0.22974300	0.06661000	3.39175300
C	-1.96470400	3.26649700	3.56586100	O	1.60734000	1.23689800	3.98068800
C	-1.98260600	2.50030700	2.40423500	C	0.98507300	1.54896600	5.23024100
C	-0.27781500	-2.97376100	-1.88132200	C	1.67473100	-2.37894000	2.37068300
C	0.65023700	-3.77719200	-1.20392300	O	1.55745500	-3.49969200	1.91830000
C	1.23644200	-4.87020900	-1.83056400	O	1.64862900	-2.08359700	3.66432100
C	0.91963500	-5.17645600	-3.15026000	C	1.29991500	-3.15550100	4.54854300
C	0.00347000	-4.38622100	-3.83423800	H	-3.23023900	2.95488100	-1.63198900

H	-0.90492000	-3.82553000	0.67367200
H	-6.92945400	-2.29328700	0.93730900
H	-7.30317600	-0.54192400	0.80860700
H	-6.88910300	-1.46478000	-0.65717200
H	-4.37982700	0.53979400	4.77539900
H	-4.29694900	-1.25438400	4.74170200
H	-2.79818700	-0.30550900	4.93216100
H	-1.04780600	-6.16285300	3.39032600
H	-1.65811700	-5.73701700	1.76726700
H	-0.03939900	-5.15962800	2.30134200
H	-7.46722200	2.94131600	-2.90388000
H	-7.12868000	1.19391400	-2.66159600
H	-7.61577300	2.20218300	-1.27507100
H	0.47312600	1.54853800	-2.59619500
H	0.95821400	3.17234100	-4.41066800
H	0.14578700	5.51363000	-4.22362100
H	-1.18959100	6.20439600	-2.24701000
H	-1.73012400	4.57211300	-0.47853900
H	0.69459200	3.89853100	0.82629400
H	0.73064200	5.22147900	2.89586600
H	-0.98773400	4.84931800	4.65481500
H	-2.72414600	3.09639400	4.32772400
H	-2.74955100	1.73756200	2.28892700
H	0.91817500	-3.57133200	-0.17030700
H	1.94678000	-5.48328500	-1.27866700
H	1.38261100	-6.02989800	-3.64206300
H	-0.26061800	-4.61995400	-4.86414100
H	-1.31049700	-2.70007700	-3.76862600
H	-3.76477000	-2.31234500	-1.47321400
H	-5.44992500	-1.81226900	-3.21323500
H	-4.90976100	-0.24684700	-5.06432800
H	-2.65985100	0.80176000	-5.18262900
H	-0.97452300	0.30836400	-3.44234800
H	2.56431900	-2.38295800	-0.11490300
H	6.18589500	-2.82259400	-2.92579300
H	4.57284100	-3.36665000	-4.74097100
H	2.32160000	-2.31468600	-4.75004700
H	1.63591600	-0.83110200	-2.88594200
H	6.10721500	-2.62869700	0.47078100
H	6.71093800	-1.16994900	2.38367500
H	6.19986800	1.25469900	2.29761300
H	5.41272600	0.87295800	-1.89011300
H	6.58792000	3.88380100	0.25000900
H	5.23396000	5.84549800	-0.46473200
H	2.88440600	5.50276200	-1.22882300
H	1.87725000	3.26196600	-1.14965400
H	2.01222000	2.19758300	1.44454100
H	0.83413000	0.63119200	5.80940200
H	1.67373600	2.21933600	5.74655900
H	0.01813900	2.03503300	5.06281600
H	1.95732600	-4.01426300	4.38004700
H	1.43306000	-2.76085300	5.55659100
H	0.25533800	-3.44609900	4.38486900

TS4

N	-5.04385000	-1.57743900	0.29985800
C	-3.86925600	-1.98173000	0.75889700
C	-2.74250900	-1.15164600	0.93631800
C	-2.91794800	0.19919100	0.60658700
C	-4.15265200	0.64395500	0.14360000
C	-5.17651400	-0.29444000	-0.00282900
C	-1.51751100	-1.77809700	1.48880600
C	-1.50013600	-2.16378300	2.85089600
N	-0.54817800	-2.87276200	3.42522700
C	0.44382800	-3.31536700	2.65820100
C	0.55976800	-3.00163300	1.30553200
C	-0.43526400	-2.21386400	0.71803900
P	-0.25112600	-1.59502000	-1.01958000
P	-1.49836300	1.35927700	0.80352300
Rh	0.58492800	0.80691800	-0.49792400
O	-3.71378900	-3.27438500	1.06783500
C	-4.83587500	-4.12524900	0.89589700
O	-2.54878200	-1.77819700	3.58858700
C	-2.53710000	-2.13157900	4.96348700
O	1.32066100	-4.09684300	3.31225200
C	2.28249100	-4.80586200	2.54676000
O	-6.34385500	0.15192100	-0.47708200
C	-7.38239000	-0.79989300	-0.64898000
C	-2.17806600	2.98804700	0.30142100
C	-2.68437400	3.14803000	-0.99358100
C	-3.23261900	4.36067400	-1.39121300
C	-3.23704400	5.44610500	-0.51876100
C	-2.69689600	5.30828400	0.75517500
C	-2.18136000	4.08329100	1.17030400
C	-1.40960500	1.51818700	2.63339500
C	-0.26713000	2.04655800	3.24061400
C	-0.22542000	2.27098500	4.61222800
C	-1.33328600	1.96784400	5.39724400
C	-2.48187100	1.45661600	4.80144400
C	-2.52442600	1.23384600	3.42935500
C	0.99389500	-2.71990300	-1.76291900
C	2.30338800	-2.68664600	-1.26800400
C	3.29814100	-3.49245400	-1.80693300
C	2.99823000	-4.33383900	-2.87619400
C	1.70640900	-4.35938900	-3.39267200
C	0.70855800	-3.55712100	-2.84446100
C	-1.80878100	-2.05790300	-1.85547800
C	-2.34449500	-3.34703200	-1.74252400
C	-3.49331300	-3.68600600	-2.44663900
C	-4.12188200	-2.74078400	-3.25650000
C	-3.59771300	-1.45751200	-3.36775200
C	-2.44027300	-1.11843500	-2.67206900
C	2.17165900	0.57646900	-1.88746700
C	3.41158800	-0.06759700	0.97628500
C	4.07991700	0.02827000	-0.28692600
C	3.51323800	0.30688100	-1.51571500
C	1.68303200	0.25723000	-3.22592200
C	0.57626700	0.97396000	-3.74987000
C	0.00200300	0.56389900	-4.95867200
C	0.53410400	-0.49604600	-5.67412600
C	1.67895700	-1.14164500	-5.20528500
C	2.24769600	-0.76840200	-3.99859500
C	0.21339900	2.22050500	-3.07799000
C	-0.49553700	3.24782400	-3.71745800
C	-0.57566200	4.50608200	-3.14443700
C	0.08097500	4.79420800	-1.95028400
C	0.79010500	3.80827000	-1.26465400
C	0.78623400	2.49935500	-1.81390400
C	1.61493400	4.13338100	-0.09765000
C	1.98593700	5.45592300	0.16838100
C	2.83764100	5.77729700	1.21540800

C	3.38421700	4.78397900	2.03304800
C	3.05374000	3.46923900	1.78863000
C	2.15749500	3.11909600	0.74781500
C	1.77915800	1.75928700	0.62224200
C	2.30390000	0.65317100	1.35908400
C	3.82969000	-1.18261200	1.88165600
O	4.27125700	-2.24015100	1.48032700
O	3.60810700	-0.89498600	3.16463600
C	3.87509900	-1.93740900	4.10810500
C	5.50751200	-0.41193800	-0.35675100
O	6.03193300	-0.89860500	-1.33150400
O	6.15010400	-0.17115200	0.79607500
C	7.46144500	-0.72614300	0.87738600
H	-4.36027700	1.68030400	-0.09984600
H	1.38485500	-3.38832400	0.72087200
H	-4.49959300	-5.11950100	1.19896900
H	-5.67513900	-3.80206300	1.52253600
H	-5.16307100	-4.13427200	-0.15178300
H	-3.47549800	-1.74828300	5.37256900
H	-2.48037300	-3.21822600	5.09037500
H	-1.68296900	-1.67347600	5.47721900
H	2.81860800	-5.44040100	3.25670100
H	1.79206200	-5.43756300	1.79336100
H	2.99418300	-4.12712800	2.05467900
H	-8.23002400	-0.24209200	-1.05269600
H	-7.07875300	-1.58926900	-1.34780900
H	-7.65531600	-1.26381900	0.30613400
H	-2.66704200	2.31046800	-1.69302400
H	-3.64940000	4.46059700	-2.39244800
H	-3.66510700	6.39679100	-0.83153800
H	-2.69867200	6.15105000	1.44418200
H	-1.79831300	3.98138600	2.18477600
H	0.58988000	2.32781300	2.63254200
H	0.67291700	2.68659900	5.06525500

H	-1.30504300	2.13959100	6.47159300
H	-3.35819000	1.23258500	5.40755400
H	-3.43169600	0.83392900	2.98080700
H	2.56273900	-2.02726800	-0.44364500
H	4.30408300	-3.44034300	-1.39254700
H	3.77219600	-4.96462700	-3.30966100
H	1.46807500	-5.00870700	-4.23357800
H	-0.29162000	-3.58095900	-3.27384800
H	-1.85771900	-4.08394100	-1.10224700
H	-3.89912900	-4.69362400	-2.36743100
H	-5.02178700	-3.01045500	-3.80671400
H	-4.08776700	-0.71941600	-4.00025400
H	-2.01636300	-0.11607200	-2.76233900
H	4.21319800	0.18419100	-2.35030800
H	-0.87494700	1.08181800	-5.34581700
H	0.06909600	-0.81014800	-6.60619500
H	2.11137200	-1.96794000	-5.76674600
H	3.10018000	-1.32381500	-3.60994100
H	-0.94210300	3.07370600	-4.69477000
H	-1.12794400	5.29564900	-3.65084600
H	0.00870100	5.80060200	-1.54345100
H	1.99746300	1.92653800	-1.92152700
H	1.63861800	6.25656100	-0.47897900
H	3.09936100	6.82040700	1.38362200
H	4.06558300	5.04439300	2.83922900
H	3.46894100	2.66904600	2.40048100
H	1.81231000	0.38891800	2.30031600
H	3.04290000	-2.65077500	4.11927700
H	4.80550800	-2.45295000	3.85200200
H	3.95908400	-1.44659300	5.07927000
H	8.08860900	-0.35733700	0.05990500
H	7.86210100	-0.41238000	1.84292700
H	7.40449200	-1.81960700	0.82155300

IM4

N	-3.65823100	-3.57989900	0.49269700
C	-2.48718500	-3.41402100	1.08826100
C	-1.82328300	-2.18075700	1.22581500
C	-2.47051600	-1.05462900	0.68682500
C	-3.70609800	-1.21549400	0.07040200
C	-4.24421800	-2.50278700	-0.00818100
C	-0.50881600	-2.13047600	1.89122200
C	-0.40401800	-2.33480900	3.29254100
N	0.70280000	-2.19097900	3.98634400
C	1.80072500	-1.77058800	3.35782600
C	1.84584000	-1.55429800	1.97908500
C	0.67634100	-1.80264800	1.24304100
P	0.73159100	-1.48065100	-0.55455600
P	-1.66589800	0.62917500	0.71971500
Rh	0.24016300	1.06705700	-0.91766500
O	-1.84532600	-4.49193400	1.55245100
C	-2.50776300	-5.74207200	1.44647700
O	-1.52930100	-2.67080200	3.92750000
C	-1.44083800	-2.87559300	5.33117600
O	2.83648200	-1.58446700	4.17878900
C	4.04925200	-1.09990800	3.62976200
O	-5.41113400	-2.62739100	-0.64731200
C	-5.94520900	-3.93628800	-0.77456500
C	-3.12081100	1.72412600	0.49998800
C	-3.68207900	1.82125900	-0.78033900
C	-4.83306400	2.56935400	-0.99405900

C	-5.43098100	3.24506500	0.06630100
C	-4.86640600	3.17510000	1.33524500
C	-3.71685200	2.42258500	1.55292600
C	-1.16746700	0.75686500	2.48082400
C	0.13948500	1.09317000	2.84113300
C	0.50384300	1.19694600	4.17936500
C	-0.44182900	0.97288800	5.17365300
C	-1.74395900	0.61604900	4.82696800
C	-2.10321800	0.48916300	3.49052300
C	2.19676100	-2.41981000	-1.18352100
C	3.09210200	-3.15794800	-0.40519100
C	4.07691000	-3.93327000	-1.01616600
C	4.19151100	-3.97138400	-2.40069700
C	3.30091400	-3.24069300	-3.18352800
C	2.30460500	-2.48714400	-2.57973300
C	-0.54588200	-2.45639900	-1.44050300
C	-0.55522000	-3.85425400	-1.35183400
C	-1.43842700	-4.59143100	-2.12741800
C	-2.30155600	-3.94680500	-3.01466400
C	-2.28113600	-2.56201700	-3.12235100
C	-1.40396000	-1.81828800	-2.33552200
C	1.78870900	1.65543100	-2.44360600
C	3.20195100	2.13814900	0.39347700
C	3.40999100	1.12110600	-0.49011000
C	2.56243800	0.83976100	-1.62707200
C	1.30537200	1.02029900	-3.69277200

C	-0.04142600	1.15119300	-4.07248300
C	-0.51535900	0.39724300	-5.15042400
C	0.33547000	-0.41930500	-5.88444400
C	1.68830800	-0.47902800	-5.56181100
C	2.16247700	0.22608700	-4.46230500
C	-0.87782100	2.17145700	-3.40797100
C	-1.73138600	2.90680100	-4.24067800
C	-2.39132000	4.03150600	-3.77408100
C	-2.19516000	4.42827600	-2.46703500
C	-1.40802900	3.67847500	-1.57560600
C	-0.76923500	2.51850200	-2.04483300
C	-1.29614700	4.14005400	-0.18622500
C	-2.18755400	5.07223000	0.35919000
C	-2.06677000	5.51089500	1.66729600
C	-1.03880900	5.02668900	2.47342800
C	-0.14205800	4.11169300	1.95114000
C	-0.25058400	3.66074100	0.62151400
C	0.75918800	2.74438100	0.10043800
C	2.05179800	2.98481600	0.45409100
C	4.12911100	2.35605100	1.55504500
O	3.74002700	2.24161300	2.69687700
O	5.34394500	2.73820300	1.18894100
C	6.29752300	2.81760800	2.25180500
C	4.56065700	0.20627800	-0.27315000
O	5.06648600	-0.02675200	0.80667900
O	4.99173200	-0.33098200	-1.41998900
C	6.15020300	-1.15866200	-1.30510800
H	-4.27827900	-0.39704500	-0.35150600
H	2.74940600	-1.18824600	1.49826900
H	-1.82887200	-6.47335200	1.89066000
H	-3.45948100	-5.73006600	1.99008900
H	-2.70784900	-5.99415400	0.39750500
H	-2.45069800	-3.14121700	5.65206400
H	-0.73919500	-3.68400000	5.56483000
H	-1.10219400	-1.96265800	5.83654500
H	4.75200100	-1.04171500	4.46374700
H	4.44637000	-1.78492700	2.86655000
H	3.91881500	-0.10313500	3.18611300
H	-6.87444600	-3.82253300	-1.33684900
H	-5.24972000	-4.59074800	-1.31522900
H	-6.14919400	-4.37700600	0.20821100
H	-3.21671900	1.30844500	-1.62388500
H	-5.25559700	2.63103300	-1.99540300
H	-6.33415200	3.82967100	-0.09904000
H	-5.31804500	3.71442500	2.16612300
H	-3.27558400	2.40245900	2.54692100
H	0.89599900	1.26354700	2.07746100
H	1.53500300	1.44609100	4.42867000
H	-0.16225100	1.05869700	6.22214800
H	-2.48260600	0.41867700	5.60246000
H	-3.11234300	0.16666800	3.23242600
H	3.01144100	-3.16704000	0.67905000
H	4.75207800	-4.52013000	-0.39518000
H	4.96237500	-4.58028400	-2.86961000
H	3.36354600	-3.27433400	-4.27028700
H	1.58472700	-1.96171000	-3.20896700
H	0.12527800	-4.36215400	-0.66726800
H	-1.44500000	-5.67768300	-2.05276500
H	-2.98314700	-4.53097500	-3.63051000
H	-2.94679400	-2.05361300	-3.81771200
H	-1.38814000	-0.72842900	-2.42623400
H	2.68787300	-0.17290900	-2.00294500
H	-1.57147400	0.45049400	-5.41419500
H	-0.05500900	-0.99453300	-6.72157600
H	2.37068000	-1.08285200	-6.15750600
H	3.21604300	0.16054700	-4.18559800
H	-1.80942400	2.63027000	-5.29089300
H	-3.00953700	4.62536500	-4.44389200
H	-2.64575800	5.35779500	-2.12884700
H	-3.02757800	5.43169700	-0.22954200
H	-2.78910800	6.22151800	2.06511500
H	-0.93773600	5.35925800	3.50438300
H	0.65644400	3.72031600	2.57918700
H	2.19995700	3.89082800	1.04781000
H	7.22226100	3.17547400	1.79747400
H	5.95363500	3.50777800	3.02820400
H	6.43938900	1.82060300	2.68525600
H	6.33909600	-1.54736500	-2.30722700
H	7.00083700	-0.56576300	-0.95305400
H	5.96585900	-1.97818200	-0.60309500
H	1.81458800	2.74300700	-2.35849900

TS5

N	-5.14173500	-0.80840800	-0.06478400
C	-4.17093700	-1.40981400	0.60777400
C	-2.94522400	-0.80773600	0.93894700
C	-2.76477300	0.52659300	0.54051300
C	-3.78450600	1.17536200	-0.14630800
C	-4.94531900	0.44913200	-0.43193300
C	-1.91510600	-1.52001100	1.71312200
C	-2.09041200	-1.72103900	3.10647200
N	-1.13471600	-2.08659300	3.92954800
C	0.09300500	-2.24830500	3.43634200
C	0.37482800	-2.20187600	2.07000000
C	-0.67256900	-1.86495800	1.19573900
P	-0.29904700	-1.72662200	-0.60546700
P	-1.13032000	1.35765300	0.84693600
Rh	0.74841300	0.63350200	-0.73298500
O	-4.32777600	-2.68609700	0.97048000
C	-5.57096100	-3.29910700	0.66797400
O	-3.31194600	-1.48101000	3.59111100
C	-3.50916700	-1.70950500	4.98009800
O	1.00718700	-2.47202000	4.38841600
C	2.37116200	-2.38787000	4.03700300
O	-5.89311900	1.08392500	-1.12996300
C	-7.06072900	0.34629300	-1.45716200
C	-1.50644200	3.13920900	0.62822700
C	-1.85842600	3.62034100	-0.63847500
C	-2.16892900	4.96117800	-0.82531400
C	-2.10859600	5.84705800	0.24662600
C	-1.72421900	5.38612200	1.50040700
C	-1.42096300	4.04156800	1.69312200
C	-0.86524000	1.20604700	2.64362800
C	0.41990300	1.00957400	3.15015300
C	0.63839200	0.99336500	4.52552300
C	-0.42879700	1.17620800	5.39646400
C	-1.71431000	1.38469200	4.89637000
C	-1.93749100	1.39797600	3.52603900
C	0.58875000	-3.31580600	-0.92193800
C	0.52912500	-4.41353200	-0.05332600
C	1.15351100	-5.61157800	-0.38489000

C	1.84293300	-5.73653400	-1.58757400
C	1.88807500	-4.65994400	-2.46688900
C	1.25897500	-3.46334700	-2.13924400
C	-1.83222100	-1.99402800	-1.57089600
C	-2.50725800	-3.21957400	-1.56064400
C	-3.62347300	-3.40395600	-2.36664200
C	-4.07449200	-2.37054300	-3.18683800
C	-3.40146500	-1.15310800	-3.20911600
C	-2.27929900	-0.96798600	-2.40755300
C	2.63950200	0.38486300	-1.94031300
C	3.43509100	0.99230200	1.14790700
C	3.28023000	-0.23679000	0.54141200
C	2.59757800	-0.42245300	-0.71133600
C	2.82546600	-0.52946400	-3.10314500
C	1.80524400	-0.58707600	-4.05218900
C	1.89108600	-1.49401900	-5.11208800
C	2.99985300	-2.32299300	-5.21651400
C	4.02318500	-2.25942900	-4.26918000
C	3.92817000	-1.37580200	-3.20019000
C	0.80284500	0.44970300	-3.86965000
C	-0.14054800	0.76917300	-4.84642000
C	-0.82783800	1.97063800	-4.77679000
C	-0.45905400	2.90075900	-3.81795700
C	0.50621000	2.63012000	-2.82560200
C	0.94838600	1.29120700	-2.74242600
C	0.95976200	3.76467700	-1.99265800
C	0.94152100	5.04797100	-2.55908400
C	1.25941500	6.17643800	-1.81997100
C	1.60839900	6.04974600	-0.47718600
C	1.71500200	4.78721500	0.07635100
C	1.44109000	3.62820200	-0.67326500
C	1.73957500	2.30836800	-0.14342000
C	2.83189700	2.19121400	0.68771200
C	4.23210100	1.12166600	2.41463200
O	3.71575900	1.01319000	3.50275600
O	5.49146400	1.45505700	2.17918000
C	6.32986600	1.50116400	3.33955400
C	3.95203500	-1.42007800	1.13763700
O	4.70372400	-1.37963500	2.08901100
O	3.65751900	-2.55552300	0.48284400
C	4.31789800	-3.73733900	0.93117600
H	-3.73638100	2.21278100	-0.45739400
H	1.36908400	-2.42697700	1.69012400
H	-5.48843600	-4.32644200	1.02995100
H	-6.39533400	-2.78461000	1.17549900
H	-5.76145500	-3.28683100	-0.41246100
H	-4.54982700	-1.44146100	5.17614600
H	-3.33325700	-2.76193900	5.23027300
H	-2.82889200	-1.09192600	5.57831100
H	2.93571700	-2.53574800	4.96010800
H	2.65198300	-3.17424800	3.32038800
H	2.61591200	-1.39959100	3.61984000
H	-7.68815800	1.02795500	-2.03538600
H	-6.80879500	-0.53960100	-2.05379500
H	-7.58866400	0.02075300	-0.55325100
H	-1.89109200	2.93886400	-1.49046800
H	-2.45293100	5.31741200	-1.81466400
H	-2.35259000	6.89763500	0.10065300
H	-1.66319400	6.07449900	2.34127000
H	-1.12574200	3.69931500	2.68294300
H	1.26324500	0.86951300	2.47626800
H	1.65070800	0.84312900	4.89569500
H	-0.26161700	1.16239600	6.47181200
H	-2.54864800	1.53916100	5.57917400
H	-2.94457800	1.55832600	3.13887800
H	-0.01143000	-4.34368400	0.88796100
H	1.09058500	-6.45520400	0.30039300
H	2.33021900	-6.67553300	-1.84429900
H	2.40765500	-4.74519200	-3.42000900
H	1.28221300	-2.64079900	-2.85263200
H	-2.16020300	-4.02931900	-0.91855600
H	-4.14296600	-4.36107400	-2.35893300
H	-4.94796100	-2.52173200	-3.81925700
H	-3.74596200	-0.34333100	-3.85133100
H	-1.74685800	-0.01438900	-2.42559000
H	2.45201900	-1.47702100	-0.93932500
H	1.10115400	-1.55173600	-5.85906800
H	3.07282400	-3.02195600	-6.04763800
H	4.89353000	-2.90577700	-4.36285600
H	4.71058300	-1.33990100	-2.44105000
H	-0.30392900	0.08749200	-5.67939600
H	-1.58822300	2.21879300	-5.51396600
H	-0.93173500	3.88140700	-3.82313100
H	0.68705600	5.16459400	-3.61019700
H	1.22440700	7.15696400	-2.29045100
H	1.81464800	6.93038600	0.12753500
H	1.99262900	4.68057700	1.12381400
H	3.34230600	3.10533500	1.00816500
H	7.32019100	1.78278700	2.97994200
H	5.95326900	2.23673800	4.05704500
H	6.35275200	0.51126400	3.80760900
H	4.00512100	-4.52477300	0.24178400
H	5.40399900	-3.60112200	0.90717800
H	4.01590700	-3.98643900	1.95500500
H	3.24591500	1.29586200	-1.90713300

IM5

N	-4.91183700	0.47684400	-2.05090800
C	-4.35000800	-0.54043800	-1.41434300
C	-3.34152000	-0.41793700	-0.44146400
C	-2.92124200	0.88650000	-0.13870000
C	-3.51194600	1.96483800	-0.78786200
C	-4.49865500	1.69658300	-1.74017600
C	-2.82720100	-1.62181400	0.24311200
C	-3.64432700	-2.25795700	1.21248400
N	-3.26144300	-3.25414600	1.98011300
C	-2.00924600	-3.69102600	1.86508700
C	-1.11508000	-3.18808300	0.92135600
C	-1.55238600	-2.15784200	0.07561300
P	-0.32643800	-1.35098400	-1.02685700
P	-1.50293300	1.15113900	1.02157700
Rh	0.75966600	0.40624100	0.38297800
O	-4.72883800	-1.78457200	-1.72643400
C	-5.74782200	-1.92884800	-2.70233200
O	-4.88269900	-1.77310200	1.35146600
C	-5.72496900	-2.42371600	2.29217100
O	-1.70896200	-4.65611400	2.74363300
C	-0.40713600	-5.20224100	2.71819500
O	-5.03383400	2.75081700	-2.36553100
C	-6.02376600	2.48979000	-3.34790100
C	-1.59096800	2.93665200	1.40909200

C	-1.25826200	3.85127800	0.40106000
C	-1.33345900	5.21799800	0.63401000
C	-1.70541000	5.69034100	1.89078400
C	-1.99319600	4.79024300	2.90986000
C	-1.93883500	3.41826400	2.67279300
C	-2.00499100	0.28669900	2.54784100
C	-1.09985800	-0.55268700	3.20318600
C	-1.48257700	-1.22400500	4.36072200
C	-2.76142600	-1.04618500	4.87707200
C	-3.66494700	-0.20119500	4.23333500
C	-3.29655500	0.45374200	3.06498300
C	0.66077600	-2.68484000	-1.82161800
C	0.40432100	-4.05456700	-1.72069700
C	1.21261600	-4.97115100	-2.38972700
C	2.28519000	-4.53472900	-3.15973700
C	2.52377400	-3.16983500	-3.29929700
C	1.70447100	-2.25399600	-2.65271700
C	-1.20677200	-0.73843600	-2.51065200
C	-1.96593700	-1.60809500	-3.30150100
C	-2.56334700	-1.14316700	-4.46517800
C	-2.39832100	0.18631200	-4.85404300
C	-1.62585300	1.04719800	-4.08269500
C	-1.02594500	0.58436200	-2.91407900
C	3.66496100	0.91280600	-0.64988300
C	2.96636200	0.08129100	2.33881800
C	2.52952000	-0.66945300	1.14383900
C	2.76995400	-0.21984700	-0.16917600
C	4.65351000	0.19938400	-1.54471500
C	4.30290400	0.40446600	-2.88610100
C	4.98474100	-0.26245700	-3.89966600
C	6.00600900	-1.14216500	-3.54619800
C	6.32610000	-1.36871900	-2.20621800
C	5.63728000	-0.70849300	-1.18866200
C	3.20145400	1.36724100	-2.93805800
C	2.53456900	1.88946200	-4.04063500
C	1.58741600	2.88469300	-3.82786400
C	1.37440800	3.38780800	-2.54935700
C	2.03873100	2.87758600	-1.41555900
C	2.90260000	1.79179500	-1.62764100
C	1.93979900	3.68882200	-0.16811400
C	2.13092000	5.06779200	-0.32595400
C	2.06481200	5.95297600	0.74230900
C	1.82862000	5.47771200	2.02910200
C	1.71032800	4.11497400	2.22853100
C	1.74517800	3.20643700	1.15056700
C	1.63549800	1.79106700	1.43894500
C	2.48200900	1.32825100	2.51623200
C	4.05254600	-0.52989100	3.15142900
O	4.68916300	-1.48546300	2.76397300
O	4.24305000	0.09769400	4.30852800
C	5.28891800	-0.43650700	5.12646400
C	2.30689800	-2.12387600	1.42856700
O	1.75328800	-2.52176900	2.43249000
O	2.83601900	-2.90608500	0.49126600
C	2.82996400	-4.30492400	0.76401300
H	-3.25597800	2.99939800	-0.58674600
H	-0.09443300	-3.54992300	0.87712800
H	-5.90434100	-3.00480000	-2.80785700
H	-6.67500600	-1.44177500	-2.37866200
H	-5.43770100	-1.49134300	-3.65963700
H	-6.66588000	-1.86876500	2.27666700
H	-5.89341200	-3.46851900	2.00680300
H	-5.27996300	-2.40641400	3.29393500
H	-0.37526600	-5.94533500	3.51820600
H	-0.21037300	-5.70439500	1.75691500
H	0.35758900	-4.43190900	2.89304900
H	-6.31740700	3.46666300	-3.73810600
H	-5.62133800	1.86354000	-4.15382500
H	-6.89066900	1.98143600	-2.90974100
H	-0.95371500	3.48653300	-0.58245200
H	-1.08864800	5.91641900	-0.16511600
H	-1.76350400	6.76161900	2.07511300
H	-2.27564500	5.15324700	3.89640700
H	-2.18074900	2.72449000	3.47614400
H	-0.10169400	-0.70955100	2.79112700
H	-0.77751800	-1.89600100	4.84761800
H	-3.06142600	-1.57241600	5.78136000
H	-4.66721400	-0.06347300	4.63632100
H	-4.01576500	1.08913200	2.54598100
H	-0.43491600	-4.41660300	-1.12857100
H	0.99660200	-6.03524800	-2.30730600
H	2.92174100	-5.25602000	-3.66882700
H	3.34554400	-2.81085000	-3.91743500
H	1.87162100	-1.18625900	-2.81655500
H	-2.09824700	-2.64716400	-2.99740400
H	-3.15313500	-1.82231900	-5.07916100
H	-2.86565200	0.54588400	-5.76926000
H	-1.48710500	2.08392100	-4.38649900
H	-0.40621000	1.25554200	-2.31307600
H	2.66177300	-0.99669000	-0.92405200
H	4.73483600	-0.10002400	-4.94802500
H	6.55949800	-1.66356200	-4.32518500
H	7.12034700	-2.06878700	-1.95443600
H	5.85654500	-0.90837900	-0.13824700
H	2.76280600	1.54444300	-5.04807100
H	1.04373200	3.30957500	-4.66985800
H	0.68495300	4.22261200	-2.41633800
H	2.35851200	5.45105600	-1.31913800
H	2.21047900	7.01741400	0.56810900
H	1.76101300	6.16244200	2.87170600
H	1.53065100	3.73302100	3.23295800
H	2.88272800	1.98774100	3.28864200
H	5.30656700	0.17960100	6.02618200
H	5.07636800	-1.48073600	5.37604100
H	6.24739700	-0.38205500	4.60030000
H	3.43261000	-4.76038300	-0.02369000
H	3.26251800	-4.49519500	1.75231300
H	1.80910300	-4.70430800	0.72343400
H	4.11729500	1.46270200	0.18815500

TS6

N	-5.17469500	0.77499600	-1.66929100
C	-4.56502400	-0.32066400	-1.24004200
C	-3.45373300	-0.33308600	-0.37753700
C	-2.97335500	0.91884900	0.03926200
C	-3.60995300	2.07661200	-0.39395800
C	-4.70204100	1.94139600	-1.25538600
C	-2.86626000	-1.62374000	0.03795400
C	-3.55205600	-2.44282000	0.96725200
N	-3.08252700	-3.56892900	1.46073600
C	-1.86816200	-3.96677200	1.08841900

C	-1.08701300	-3.26143200	0.17095600
C	-1.62117800	-2.08961900	-0.37941600
P	-0.55582000	-0.99914000	-1.39069300
P	-1.42107400	1.02404100	1.04291400
Rh	0.58965800	0.43709900	0.04849700
O	-4.99743800	-1.51006300	-1.67282300
C	-6.07618300	-1.51408100	-2.59408500
O	-4.74917700	-2.00531700	1.37018000
C	-5.43921700	-2.79892500	2.32480600
O	-1.48575200	-5.10589900	1.67791700
C	-0.22280400	-5.64511000	1.34414800
O	-5.27776200	3.07112700	-1.67975600
C	-6.36415800	2.94622000	-2.58418200
C	-1.43306200	2.75252700	1.63901000
C	-1.14909500	3.76854500	0.71581700
C	-1.23820400	5.10309500	1.08757600
C	-1.56943300	5.44013200	2.39839700
C	-1.79570600	4.43811300	3.33465000
C	-1.73281900	3.09763200	2.95830400
C	-1.78094500	-0.03806800	2.48420900
C	-0.85397100	-1.00409700	2.88216200
C	-1.13050500	-1.84712700	3.95281700
C	-2.32975100	-1.71546900	4.64507200
C	-3.26113600	-0.75414600	4.25334200
C	-2.99921700	0.07236100	3.16721700
C	0.55614800	-2.06742500	-2.38689100
C	0.30098200	-3.40662800	-2.70201400
C	1.16507100	-4.10449400	-3.54154100
C	2.28844100	-3.47771800	-4.07343300
C	2.53462600	-2.13813300	-3.78635500
C	1.66635600	-1.43556100	-2.95957200
C	-1.57760400	-0.27818500	-2.72666400
C	-2.36790300	-1.09374600	-3.54347400
C	-3.06619800	-0.53941500	-4.60823000
C	-2.97717200	0.82799100	-4.86704700
C	-2.18253100	1.63990900	-4.06501800
C	-1.47849600	1.08695200	-2.99818100
C	4.01899100	1.02393100	-0.12436500
C	2.60133600	-0.24433100	2.53668900
C	2.65229200	-0.81281800	1.23833500
C	2.85363500	0.08084000	0.15921000
C	5.06489900	0.12193700	-0.74404800
C	5.06316600	0.27833000	-2.13860600
C	5.89902800	-0.50205300	-2.93140700
C	6.72569200	-1.43437800	-2.30993800
C	6.71570500	-1.59224900	-0.92240300
C	5.87353100	-0.81829600	-0.12634400
C	4.09876000	1.32193900	-2.48765000
C	3.67031400	1.78126700	-3.73036000
C	2.70255600	2.78080500	-3.76813100
C	2.22128400	3.35067700	-2.59174000
C	2.65399900	2.91226100	-1.33044800
C	3.56784100	1.85858400	-1.30379700
C	2.29350000	3.67430000	-0.11313400
C	2.40960400	5.06578200	-0.18492900
C	2.19862800	5.88088400	0.92010900
C	1.88283800	5.30422800	2.14404400
C	1.79565800	3.92578400	2.24880900
C	1.97733300	3.08379900	1.13418800
C	1.75688000	1.64981100	1.31436300
C	2.21160900	1.07530000	2.53398100
C	3.01852900	-0.99997400	3.74643800

O	3.82037900	-1.90312800	3.71956900
O	2.43013600	-0.52643400	4.84708200
C	2.81601400	-1.17289800	6.06295700
C	2.43091200	-2.26876700	1.03017300
O	1.77640300	-2.96930800	1.77225700
O	3.06199400	-2.69286500	-0.06656100
C	2.98323500	-4.08928100	-0.34449200
H	-3.30349000	3.07472300	-0.09947100
H	-0.07909300	-3.58310600	-0.06826900
H	-6.26914600	-2.56609800	-2.81628200
H	-6.96878500	-1.04895600	-2.16046000
H	-5.80661700	-0.97291500	-3.51078500
H	-6.38823800	-2.28669100	2.49996100
H	-5.61484700	-3.81039300	1.94224900
H	-4.86450200	-2.87196900	3.25710500
H	-0.13576400	-6.57718500	1.90712300
H	-0.16181500	-5.87010800	0.26709500
H	0.58985700	-4.96144500	1.62550800
H	-6.67527200	3.96740800	-2.81464200
H	-6.05373500	2.42697500	-3.49949900
H	-7.19372400	2.39116900	-2.13080400
H	-0.86874400	3.50790600	-0.30768800
H	-1.03444400	5.88289600	0.35574700
H	-1.63910800	6.48645000	2.69081800
H	-2.04036900	4.69588300	4.36355000
H	-1.93276000	2.32237300	3.69622600
H	0.06848000	-1.13027700	2.31694000
H	-0.41122200	-2.61955800	4.22289500
H	-2.55110000	-2.37397200	5.48318000
H	-4.20574300	-0.65980000	4.78703000
H	-3.74876000	0.79420400	2.83947200
H	-0.57580500	-3.91002800	-2.29566200
H	0.95511300	-5.14503400	-3.78361200
H	2.96345000	-4.03010500	-4.72447200
H	3.40297400	-1.63359900	-4.20843100
H	1.84420800	-0.37082100	-2.78052500
H	-2.44930800	-2.16168000	-3.33771000
H	-3.67907400	-1.17695000	-5.24381100
H	-3.52414400	1.25849400	-5.70412900
H	-2.10993300	2.70699000	-4.26799200
H	-0.84752800	1.71407400	-2.36386600
H	2.55999000	-0.34429400	-0.81774900
H	5.92182700	-0.37968000	-4.01448000
H	7.39314800	-2.04677100	-2.91345400
H	7.37269300	-2.32580800	-0.45939100
H	5.84445000	-0.95857500	0.95562800
H	4.07399200	1.36546400	-4.65304500
H	2.33540500	3.14304000	-4.72642300
H	1.49494800	4.16206900	-2.64570500
H	2.71271600	5.51280000	-1.13064700
H	2.29659600	6.96048600	0.82534700
H	1.70665500	5.92314800	3.02164400
H	1.50314400	3.49748000	3.20524300
H	2.31231700	1.67103100	3.44284200
H	2.24884800	-0.68176000	6.85471500
H	2.57356100	-2.23941700	6.01681300
H	3.89236400	-1.06043100	6.22942600
H	3.72996800	-4.27843700	-1.11795700
H	3.19901400	-4.67047600	0.55813000
H	1.98576100	-4.34520100	-0.72423400
H	4.37365700	1.58045900	0.75270700

IM6

N	-4.92319300	1.49461300	-2.02121100	C	3.58443300	2.73461200	-0.87242500
C	-4.37362800	0.29255300	-1.91835100	C	4.34859800	1.60229900	-0.60374900
C	-3.43130800	-0.07201000	-0.93989000	C	2.89446700	3.48545000	0.20066300
C	-3.06781500	0.93046900	-0.02501000	C	2.94033700	4.88326700	0.11322000
C	-3.64596200	2.19090800	-0.10988800	C	2.41442200	5.70823100	1.09529800
C	-4.56420400	2.41693000	-1.14009200	C	1.83613600	5.13997500	2.22438100
C	-2.88502200	-1.44414500	-0.89490600	C	1.77932100	3.76202800	2.33716500
C	-3.69417400	-2.52152400	-0.45040500	C	2.27788600	2.90314500	1.34079900
N	-3.25885700	-3.74547600	-0.23943100	C	2.10441700	1.46026400	1.55469600
C	-1.96055400	-3.99464500	-0.40670000	C	1.39023700	0.87679600	2.59544900
C	-1.06026500	-3.03796200	-0.87336400	C	1.17974200	-1.36556500	3.82922600
C	-1.55435300	-1.75802800	-1.15175000	O	1.58212600	-2.45205400	4.15286600
P	-0.35245600	-0.44157100	-1.55242700	O	0.22015800	-0.67913500	4.47830700
P	-1.72117600	0.60087700	1.18755600	C	-0.36394600	-1.33769400	5.60024300
Rh	0.40950900	0.42406500	0.43369500	C	2.55184800	-2.25051800	1.05417600
O	-4.69633800	-0.64394900	-2.81491500	O	1.93640100	-3.22105500	1.43766000
C	-5.66235300	-0.30106300	-3.79673700	O	3.26678300	-2.22214300	-0.07966400
O	-4.97453800	-2.23421400	-0.20289000	C	3.35684500	-3.44171700	-0.80663300
C	-5.79370500	-3.29174700	0.27783900	H	-3.42575500	2.99870000	0.58029500
O	-1.61022100	-5.24242600	-0.07697600	H	-0.00192100	-3.26098100	-0.97392400
C	-0.24360700	-5.59609600	-0.17653900	H	-5.76769000	-1.18644300	-4.42752600
O	-5.08475900	3.64407900	-1.22402300	H	-6.62306800	-0.05259000	-3.33101000
C	-5.97782600	3.89978000	-2.29799800	H	-5.32947100	0.55756800	-4.39259800
C	-1.80466100	1.94025200	2.42634000	H	-6.78811600	-2.85747400	0.40297800
C	-1.47770500	3.24607100	2.03680900	H	-5.82495800	-4.12028200	-0.43818100
C	-1.49978500	4.28160900	2.96346700	H	-5.41558100	-3.67140200	1.23534600
C	-1.78855800	4.01672000	4.29948300	H	-0.17332900	-6.62877700	0.17129500
C	-2.06148100	2.71321500	4.70356400	H	0.10247100	-5.54236100	-1.22050400
C	-2.08026400	1.67919200	3.77282200	H	0.38823200	-4.94985100	0.45123700
C	-2.25869800	-0.92216200	2.04507000	H	-6.26674900	4.94804900	-2.19779000
C	-1.38122900	-1.99613900	2.21310000	H	-5.48451300	3.73074300	-3.26325000
C	-1.78843400	-3.14066100	2.89181900	H	-6.86252600	3.25552500	-2.23753300
C	-3.07901000	-3.21680500	3.40784000	H	-1.19354500	3.45368200	1.00349000
C	-3.96150200	-2.14934600	3.24337300	H	-1.26980300	5.29548900	2.64108600
C	-3.55992200	-1.00882700	2.55717800	H	-1.79414400	4.82562700	5.02756500
C	0.91062200	-1.25096600	-2.61433100	H	-2.27410800	2.49904500	5.74954800
C	0.61026400	-2.37325900	-3.39916900	H	-2.29831100	0.66346300	4.09856800
C	1.56069200	-2.90332500	-4.26455100	H	-0.37814300	-1.95577200	1.78636900
C	2.81958900	-2.31832300	-4.36445000	H	-1.08741700	-3.96593800	3.01256400
C	3.11850800	-1.19143500	-3.60681400	H	-3.40248600	-4.11193300	3.93596200
C	2.16880800	-0.65574900	-2.74290600	H	-4.97209600	-2.20915100	3.64430100
C	-1.12430000	0.72962900	-2.72338000	H	-4.26094300	-0.18467100	2.41497900
C	-1.74486200	0.26822300	-3.88932300	H	-0.36935800	-2.84370100	-3.33488500
C	-2.26939200	1.17551600	-4.80019000	H	1.31195600	-3.77536700	-4.86656000
C	-2.17510000	2.54503700	-4.55600400	H	3.56340800	-2.73568200	-5.04061700
C	-1.54587700	3.00789300	-3.40537300	H	4.09485000	-0.71564400	-3.68256700
C	-1.01485000	2.10177600	-2.49089300	H	2.41644200	0.25026600	-2.18520100
C	4.35830300	0.77807800	0.65813100	H	-1.83299500	-0.80171200	-4.07951000
C	1.71446800	-0.53370600	2.71437300	H	-2.75194400	0.81367500	-5.70668500
C	2.51908300	-0.90257000	1.66986100	H	-2.58598700	3.25334100	-5.27342900
C	2.87330500	0.34406400	0.87683000	H	-1.46593200	4.07707700	-3.21733900
C	5.34605500	-0.31028200	0.33570200	H	-0.51160200	2.45058500	-1.58533100
C	5.76964000	-0.19504700	-0.99686300	H	2.49408200	0.18758900	-0.17571300
C	6.66057700	-1.11748900	-1.53693900	H	6.99657400	-1.03444900	-2.57070200
C	7.13678800	-2.14066300	-0.72097200	H	7.84092900	-2.86684500	-1.12309600
C	6.74022600	-2.23229100	0.61447100	H	7.14026700	-3.02683200	1.24150600
C	5.84345700	-1.31127700	1.15368900	H	5.52976400	-1.39009700	2.19540800
C	5.13333800	0.98312100	-1.58737700	H	5.81237600	1.05314300	-3.64292100
C	5.18319400	1.50501800	-2.87589200	H	4.39040000	3.02073000	-4.18154100
C	4.39291500	2.61329900	-3.17245100	H	2.99650000	4.07603600	-2.46008600
C	3.60757700	3.21477200	-2.19402000	H	3.45147400	5.33339000	-0.73534800

H	2.48772900	6.78902700	0.99309400
H	1.44916600	5.76475900	3.02730200
H	1.34008000	3.33716500	3.23682100
H	0.77607800	1.39617300	3.32371300
H	-0.87070500	-0.56016200	6.17683600
H	-1.08742100	-2.08611900	5.25207200
H	0.40548500	-1.82900300	6.20334500
H	4.14557200	-3.28320600	-1.54641400
H	3.60997400	-4.27048000	-0.13737400
H	2.40528600	-3.65541000	-1.31388400
H	4.66508000	1.34928000	1.54975100

6.4. Theoretical Mechanistic Studies of Oxidative Cyclization Step

We calculated the oxidative cyclization pathway using dimethyl acetylenedicarboxylate (**2a**, DMAD) and a simplified diyne model (phenylacetylene). Four stereoisomers are assumed for the rhodacycle **IM2** formed from phenylacetylene and DMAD (Figure S35). **IM2a**, **IM2b**, **IM2c**, and **IM2d** (**IM2a'**, **IM2b'**, **IM2c'**, and **IM2d'**) can be interconverted by coordination-site exchange. Thus, in the case of using diynes, **IM2a** / **IM2b** (**IM2a'** / **IM2b'**) corresponds to the formation of rhodacycle **A**, and **IM2c** / **IM2d** (**IM2c'** / **IM2d'**) corresponds to the formation of rhodacycle **B**.

We calculated the transition states of the oxidative cyclization to give these rhodacycles for P-phos and H_8-BINAP, summarizing the energy diagrams in Figure S35 and the transition state energies and activation energies in Table S15.

In a previous report by Pla-Quintana/Sola et al., the oxidative cyclization follows the Curtin-Hammet pathway, and the product is obtained via the most stable transition state.[21] When using P-phos, **TS1d** ($G^‡$ = 5.1 kcal/mol) is the most stable. In contrast, when using H_8-BINAP, **TS1b** ($G^‡$ = −7.2 kcal/mol) is the most stable. Furthermore, comparing the activation energies reveals that the activation barriers for **TS1c** ($\Delta G^‡$ = 16.1 kcal mol^{-1}) and **TS1d** ($\Delta G^‡$ = 16.2 kcal mol^{-1}) are lower when using P-phos. In contrast, the activation barrier for **TS1b** ($\Delta G^‡$ = 13.1 kcal mol^{-1}) is lower when using H_8-BINAP. These results are consistent with the regioselectivity observed in Figure 3.

Next, we analyzed the transition state structures to clarify the origin of the change in regioselectivity. Both the dihedral angle and the coordinate angle of the ligand in the transition state (Table S16) are larger for H_8-BINAP than for P-phos, suggesting that the steric crowding around the complex varies with the ligand, presumably influencing the selectivity of the resulting intermediate. Furthermore, from the NCI and NBO analyses of the transition state using P-phos (Figure S36), noncovalent interactions were observed between one of the phenyl groups on the ligand and phenylacetylene in **TS1b**.

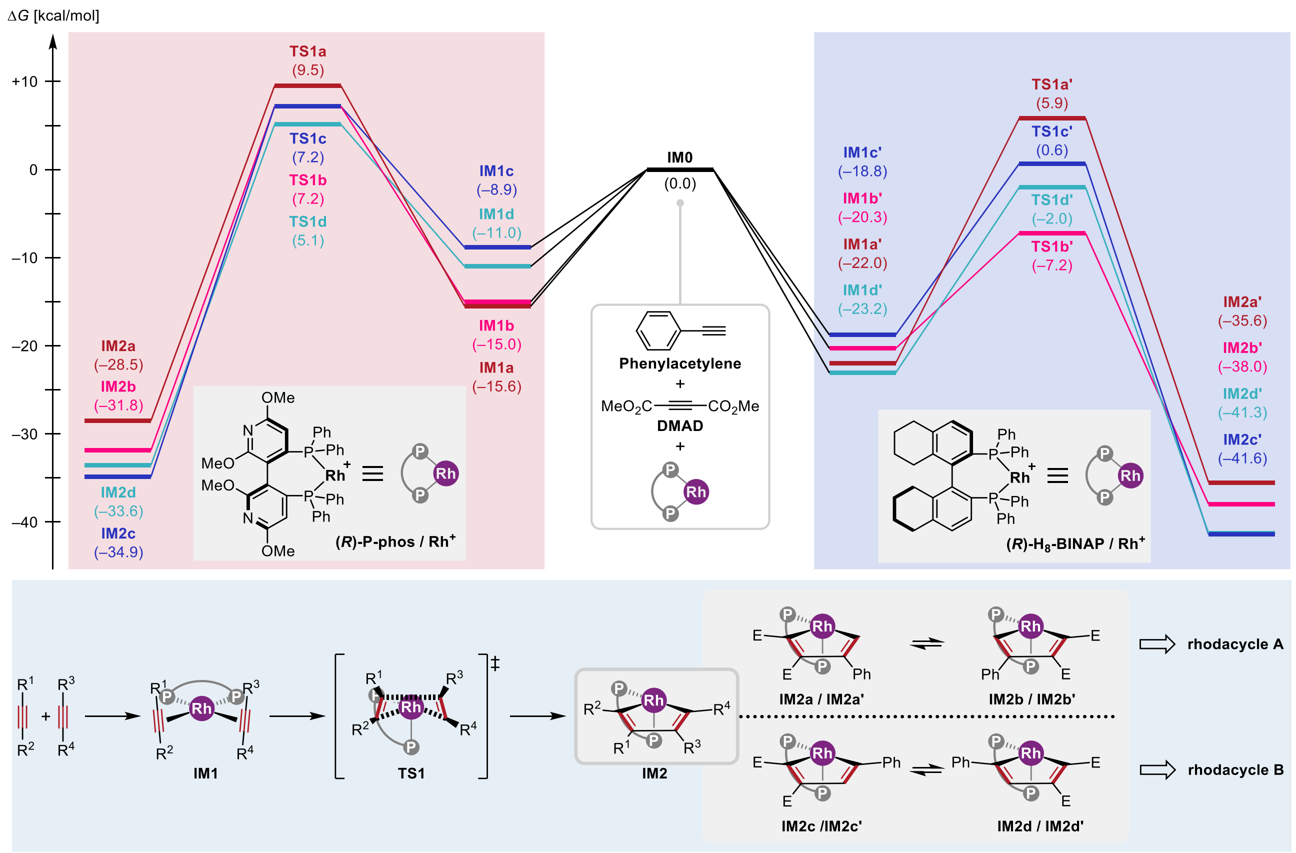

Figure S35. Energy diagram of oxidative cyclization step. Energy changes are shown in kcal mol^{-1}, and represent the relative free energies calculated at the M06/6-311+G(d,p)&SDD(Rh)+PCM[(CH2Cl)2]//M06/6-31G(d)&LANL2DZ(Rh) levels of theory.

Table S15. Transition state energies and activation energies of oxidative cyclization steps.

Ligand	Type	Energy [kcal mol^{-1}]			
		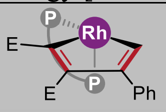 path a / a'	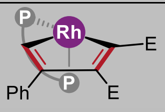 path b / b'	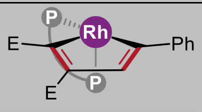 path c / c'	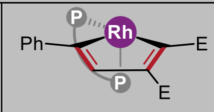 path d / d'
P-phos	$G^{\ddagger}$	9.5	7.2	7.2	**5.1**
	$\Delta G^{\ddagger}$	25.0	22.1	16.1	16.2
H_8-BINAP	$G^{\ddagger}$	5.9	**−7.2**	0.6	−2.0
	$\Delta G^{\ddagger}$	27.9	13.1	19.4	21.2

Table S16. Structural parameters.

	Dihedral angle [°]	Bite angle [°]
**(*R*)-P-phos / Rh$^+$**	68.6	92.0
**TS1a**	71.4	93.7
**TS1b**	72.5	92.1
**TS1c**	73.2	92.0
**TS1d**	67.0	89.2
**(*R*)-H_8-BINAP / Rh$^+$**	71.2	91.1
**TS1a'**	79.2	94.6
**TS1b'**	77.3	91.3
**TS1c'**	81.5	92.4
**TS1d'**	75.6	90.8

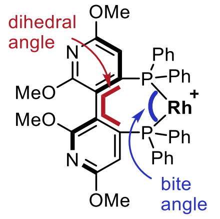

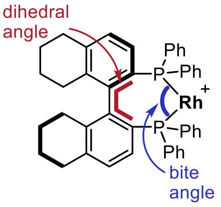

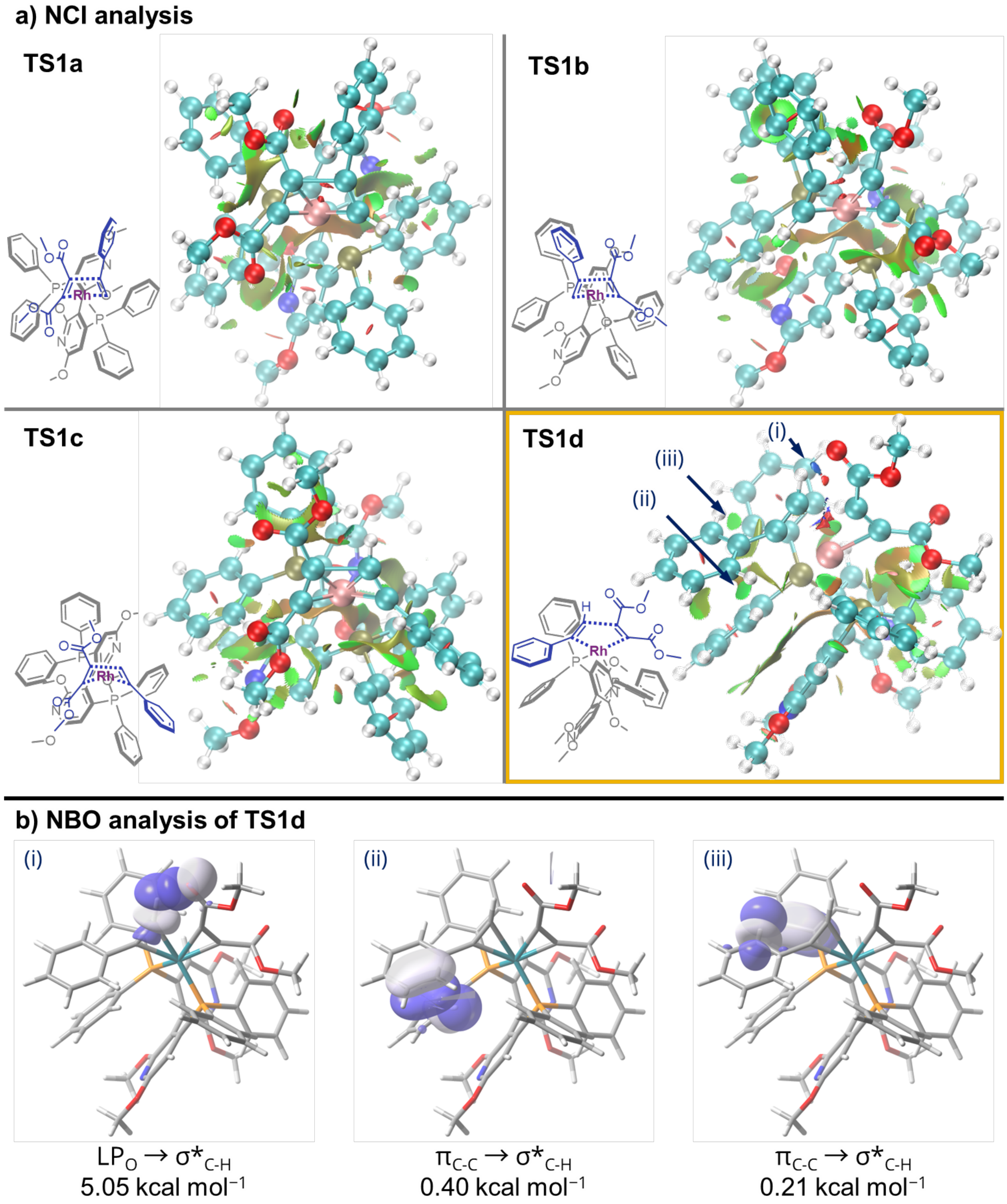

Figure S36. Theoretical mechanistic studies of the transition states. NCI and NBO analyses were conducted at the M06/6-311+G(d,p)&SDD(Rh)//M06/6-31G(d)&LANL2DZ(Rh) levels of theory.

Table S17. Summary of the Gibbs-free energies and imaginary frequencies.

	G (hartree) M06/6-311+G(d,p)&SDD +PCM[$(CH_2Cl)_2$]	*G* (hartree) M06/6-31G(d)&LANL2DZ	Imaginary frequency (cm^{-1}) M06/6-31G(d)&LANL2DZ
**Phenylacetylene**	-308.147899	-308.066931	None
**DMAD**	-532.854077	-532.702529	None
**(*R*)-P-phos / Rh^+**	-2670.633230	-2668.988187	None
**IM1a**	-3511.660015	-3509.783991	None
**IM1b**	-3511.659070	-3509.782584	None
**IM1c**	-3511.649328	-3509.773288	None
**IM1d**	-3511.652802	-3509.778167	None
**TS1a**	-3511.620126	-3509.746138	-478.3568
**TS1b**	-3511.623807	-3509.744242	-375.9268
**TS1c**	-3511.623654	-3509.744522	-403.5519
**TS1d**	-3511.627045	-3509.751586	-185.8685
**IM2a**	-3511.680696	-3509.807993	None
**IM2b**	-3511.685888	-3509.807630	None
**IM2c**	-3511.690872	-3509.810890	None
**IM2d**	-3511.688685	-3509.818215	None
**(*R*)-H_8-BINAP / Rh^+**	-2492.377015	-2490.791173	None
**IM1a’**	-3333.414105	-3331.597363	None
**IM1b’**	-3333.411292	-3331.593610	None
**IM1c’**	-3333.408998	-3331.593172	None
**IM1d’**	-3333.415899	-3331.595585	None
**TS1a’**	-3333.369595	-3331.550732	-477.0877
**TS1b’**	-3333.390429	-3331.570525	-301.6970
**TS1c’**	-3333.378012	-3331.561239	-360.2558
**TS1d’**	-3333.382167	-3331.564456	-157.5794
**IM2a’**	-3333.435760	-3331.617790	None
**IM2b’**	-3333.439585	-3331.623332	None
**IM2c’**	-3333.445221	-3331.632022	None
**IM2d’**	-3333.444870	-3331.631738	None

Cartesian Coordinates of the Computed Structures

Structures of **(*R*)-P-phos / Rh^{+}** and **DMAD** were shown in section 6.3.

Phenylacetylene

C	1.20873800	-0.11965700	0.00000000	C	-0.00003500	3.22503400	0.00000000
C	1.20492300	-1.50746700	0.00000000	H	2.14608800	0.43350800	0.00000000
C	0.00002200	-2.20448000	0.00000000	H	2.14881500	-2.04965100	0.00000000
C	-1.20489900	-1.50747900	0.00000000	H	0.00001700	-3.29307200	0.00000000
C	-1.20874000	-0.11968100	0.00000000	H	-2.14876900	-2.04970300	0.00000000
C	0.00000000	0.58926100	0.00000000	H	-2.14608500	0.43349300	0.00000000
C	-0.00002700	2.01638200	0.00000000	H	0.00004100	4.29394700	0.00000000

IM1a

C	-1.61199900	0.30883900	3.06172700	C	1.62521300	5.13544600	1.55455300
C	-2.42273200	0.97072200	2.41094100	C	0.24141600	0.77771000	-1.66234600
C	-3.45048100	1.68900000	1.72619400	C	-0.77422600	1.69977600	-1.93224200
C	-4.78764600	1.46743700	2.09438200	C	1.56546100	1.20526200	-1.50982500
C	-3.15673600	2.57359000	0.67599000	C	-0.39723900	3.03579000	-2.07675500
C	-5.80780000	2.13058300	1.42589800	C	1.80919100	2.56539800	-1.79401500
C	-4.18338400	3.22105000	0.00741700	P	-0.27853600	-0.91244900	-1.18166700
C	-5.50752500	3.00429600	0.38239800	Rh	-1.01642700	-0.34526400	0.93538000
C	-2.34299000	-1.95106900	1.13766600	H	-1.01213400	-0.06319600	3.87361200
C	-2.96804100	-1.11622600	0.43688900	H	-5.00895000	0.77264000	2.90292600
C	-4.03603400	-0.64113000	-0.44611700	H	-2.12522300	2.72241000	0.35686700
O	-3.90764200	0.23265400	-1.27125100	H	-6.84237000	1.96323000	1.71917200
O	-5.15632800	-1.32973600	-0.22602100	H	-3.94294300	3.88125100	-0.82367000
C	-6.25092700	-0.97252800	-1.07344000	H	-6.31134600	3.51575000	-0.14390900
C	-2.04315600	-3.19834700	1.83702100	H	-7.08631500	-1.59855500	-0.75701000
O	-1.12552600	-3.34569900	2.61291600	H	-6.49283800	0.08929000	-0.95423300
O	-2.90838000	-4.14717800	1.48482700	H	-5.99529500	-1.16724500	-2.12108100
C	-2.71547700	-5.41490900	2.12299900	H	-1.72527800	-5.81495600	1.88227800
P	1.21508700	0.52666300	1.50969000	H	-2.80672600	-5.30971200	3.20865100
C	1.72125700	-0.02031100	3.17783900	H	-3.50003800	-6.06331400	1.73156200
C	2.52265600	0.76947400	4.00702800	H	2.82966300	1.76497500	3.68835200
C	1.32801700	-1.29443300	3.60448300	H	0.67789200	-1.90944500	2.97900500
C	2.92880500	0.28620400	5.24688300	H	3.54993000	0.90683300	5.88986500
C	1.73992700	-1.77271400	4.84368900	H	1.42672800	-2.76331400	5.16797200
C	2.54096700	-0.98337200	5.66461000	H	2.85985300	-1.35647000	6.63605100
C	2.61549100	-0.00454300	0.43266400	H	3.65540900	-1.13997100	1.97958400
C	2.62366600	0.35576100	-0.92541900	H	1.27477400	-1.65197000	-3.54959300
C	3.61459000	-0.82278800	0.94089600	H	0.95908200	-2.89353600	0.56696200
C	3.68024300	-0.14742000	-1.69903000	H	2.90642400	-3.46564500	-3.96832400
C	4.59787300	-1.27043500	0.04954300	H	2.59416300	-4.71294000	0.13672600
C	1.03222200	-2.14847200	-1.45686800	H	3.56992500	-4.99514900	-2.12781700
C	1.56968600	-2.31949000	-2.73854900	H	-1.26831600	0.12496000	-3.83541100
C	1.39525300	-3.01653600	-0.42660600	H	-1.96005900	-3.23931700	-1.22394600
C	2.48825000	-3.33307800	-2.97166700	H	-2.97902300	-0.73561600	-5.38061200
C	2.31020300	-4.03924100	-0.66944000	H	-3.68722400	-4.07358300	-2.76553000
C	2.85809000	-4.19374100	-1.93746500	H	-4.22569500	-2.81412500	-4.84113800
C	-1.52323500	-1.47107400	-2.39882300	H	3.52407800	2.34862000	1.12940600
C	-1.79997900	-0.79127900	-3.58701600	H	-0.68484800	2.68563900	1.96393700
C	-2.19412700	-2.66974300	-2.12366500	H	3.72584100	4.80650800	1.19507100
C	-2.76833800	-1.27590000	-4.45980600	H	-0.49696700	5.14721900	1.94637600
C	-3.16591500	-3.14623700	-2.99492600	H	1.71675200	6.22019000	1.56401100
C	-3.46335600	-2.44206000	-4.15909800	H	-1.82800400	1.42556200	-1.97147700
C	1.39569000	2.34920100	1.52198700	N	4.63939200	-0.93583600	-1.23254600
C	2.64037500	2.95740900	1.32052100	N	0.86282800	3.45310200	-2.06133500
C	0.27700300	3.15087000	1.76174600	O	5.52856000	-2.09066000	0.54307200
C	2.75178300	4.34324300	1.34595000	O	3.67537100	0.16946100	-2.99827500
C	0.38705600	4.53721200	1.76707300	O	3.08182500	2.96849700	-1.75203600

O	-1.38207400	3.93237300	-2.22706400
C	-1.00619500	5.29854600	-2.33179800
H	-0.37429300	5.46737400	-3.21116900
H	-1.94135800	5.85479400	-2.42858100
H	-0.46006100	5.62438900	-1.43756600
C	3.34204700	4.33319900	-2.04549500
H	2.81329200	4.99153000	-1.34472700
H	4.42291800	4.45444000	-1.94185400
H	3.03052500	4.57959200	-3.06710400
C	4.71886000	-0.35988100	-3.80167400
H	4.52931100	0.01038800	-4.81165600
H	5.69921600	-0.01740600	-3.45106200
H	4.70482900	-1.45731700	-3.78799500
C	6.50981800	-2.58088400	-0.35821000
H	7.10222500	-1.76078000	-0.78037300
H	7.14775600	-3.24217500	0.23181500
H	6.04225700	-3.13578300	-1.18111600

IM1b

N	-4.33517500	1.70969100	-1.45097700
C	-4.02850400	0.63952200	-0.73291000
C	-2.83860100	0.48623900	0.00598000
C	-1.94639400	1.56838100	-0.02299600
C	-2.26467100	2.70748100	-0.75481700
C	-3.46947900	2.71214800	-1.46444000
C	-2.60712300	-0.77890300	0.73289700
C	-3.36523000	-1.08148600	1.87847700
N	-3.26691800	-2.21059000	2.56635200
C	-2.39757500	-3.11808700	2.14456100
C	-1.58348800	-2.95130600	1.01878700
C	-1.69709100	-1.75960300	0.31427100
P	-0.57878000	-1.37464300	-1.09415000
P	-0.29303800	1.39359900	0.77878500
O	-4.87922900	-0.39174900	-0.72057800
C	-6.07762100	-0.25776800	-1.46861500
O	-4.22674100	-0.14599700	2.29212400
C	-4.98122700	-0.42734200	3.46006000
O	-2.26752000	-4.26529600	2.81639900
C	-3.06111100	-4.43229800	3.98114200
O	-3.73141000	3.79497600	-2.20254400
C	-4.92525700	3.78257900	-2.97110700
C	-0.66685800	0.85258300	2.47551800
C	0.03645800	-0.21112000	3.04431400
C	-0.25325400	-0.61706900	4.34385700
C	-1.23816700	0.03720800	5.07636500
C	-1.94271900	1.09896700	4.51072900
C	-1.66582500	1.50364100	3.21115800
C	-1.69721800	-0.94067900	-2.46786700
C	-2.79528100	-1.74874200	-2.78705400
C	-3.64277000	-1.38894300	-3.82828800
C	-3.41517600	-0.21328200	-4.54209800
C	-2.33449400	0.60193400	-4.22009100
C	-1.47440100	0.23585200	-3.18980400
Rh	1.12840400	0.18577400	-0.59707200
C	1.98192500	-0.51364700	-2.59614700
C	2.84561300	1.43952400	0.05856100
C	2.79294500	0.36970100	0.70839900
C	3.08868000	-0.79791200	-2.13410300
C	3.47013600	2.64788600	-0.46816200
O	3.64123900	2.85244100	-1.64871200
O	3.85347500	3.45412900	0.51927500
C	4.53356300	4.64037600	0.10332000
H	-1.63024500	3.58611800	-0.80187100
H	-0.90551400	-3.75065900	0.73748500
H	-6.62060000	-1.19447100	-1.32298800
H	-6.67531600	0.58683300	-1.10710800
H	-5.85854900	-0.10500900	-2.53343900
H	-5.61035600	0.45109200	3.62237300
H	-5.60329200	-1.31917000	3.32260500
H	-4.32047100	-0.58742900	4.32195200
H	-2.79222300	-5.41129500	4.38320200
H	-2.84654800	-3.64705300	4.71669700
H	-4.12975200	-4.40300400	3.73842700
H	-4.93436600	4.72817900	-3.51710600
H	-4.92935600	2.93696900	-3.67092800
H	-5.80890600	3.70854200	-2.32671700
H	0.78837000	-0.74565100	2.46322600
H	0.28993300	-1.45393700	4.77887100
H	-1.46156900	-0.28032000	6.09357800
H	-2.71697000	1.60896500	5.08191700
H	-2.23490300	2.31857300	2.76171300
H	-2.99165300	-2.65380900	-2.21042700
H	-4.49066700	-2.02450800	-4.07867000
H	-4.08641000	0.06822800	-5.35174600
H	-2.16166400	1.52706000	-4.76740500
H	-0.64091100	0.88841000	-2.91910200
H	4.73117600	5.20176400	1.01785900
H	5.46946800	4.38411000	-0.40407600
H	3.90312900	5.22277400	-0.57740700
C	0.20450800	-2.96540900	-1.52259300
C	-0.03874700	-3.62766300	-2.72750300
C	1.13321600	-3.49652700	-0.61696400
C	0.61782900	-4.82218200	-3.01265000
H	-0.74123300	-3.21494800	-3.45025500
C	1.76494800	-4.70215100	-0.89742800
H	1.36869200	-2.96319000	0.30750900
C	1.51084100	-5.36552400	-2.09650500
H	0.42343300	-5.33105000	-3.95472800
H	2.46493600	-5.12222900	-0.17617200
H	2.01312000	-6.30548500	-2.31690500
C	0.33376400	3.10691500	0.90777000
C	0.48682000	3.76301700	2.13025100
C	0.69599600	3.77023900	-0.27408100
C	0.96139200	5.07214800	2.16724600
H	0.23570600	3.25611200	3.06027800
C	1.14787200	5.08221700	-0.23485800
H	0.62950600	3.25318100	-1.23393800
C	1.27730200	5.73713900	0.98864700
H	1.07625200	5.57415000	3.12593400
H	1.40639800	5.58982900	-1.16315000
H	1.63391900	6.76538600	1.02094400
C	3.32881600	-0.71699000	1.52688700
O	2.73436400	-1.73414600	1.81312900
O	4.57382700	-0.43155700	1.89732600
C	5.20981500	-1.42208800	2.70967000
H	6.21576600	-1.04408000	2.89865000
H	4.66196200	-1.55246300	3.64867700
H	5.25131800	-2.37794800	2.17611400
C	4.35558600	-1.09215600	-1.56719600
C	5.35037800	-0.09992200	-1.53896200
C	4.62420600	-2.36993400	-1.04952500
C	6.59344400	-0.38720600	-0.99456200
H	5.12784000	0.88288800	-1.95269700

C	5.87448700	-2.64684700	-0.51667100
H	3.84392700	-3.12898400	-1.08240500
C	6.85646700	-1.65726100	-0.48626900
H	7.36561400	0.37886400	-0.97459600
H	6.09023300	-3.64167000	-0.13051100
H	7.83712500	-1.88140300	-0.06965500
H	1.28100900	-0.45242000	-3.41185900

IM1c

P	1.24935600	-0.87015600	0.15286500
C	2.81875600	-1.05389000	1.08559300
C	3.70928800	-2.11015800	0.87880700
C	3.10361800	-0.11255200	2.08291800
C	4.83912100	-2.24089800	1.68108300
C	4.22408900	-0.25483300	2.89293600
C	5.09059400	-1.32637800	2.69823700
C	-0.01104700	-1.60791400	1.27840000
C	-1.26734800	-1.97226500	0.77030100
C	0.22216800	-1.64687500	2.64773300
C	-2.21930700	-2.40775400	1.71240800
C	-0.83546700	-2.03637300	3.47271900
C	-2.79911800	0.75240000	0.98333200
C	-4.09889900	0.33937300	0.65903400
C	-5.05849800	0.22735100	1.65661400
C	-3.43248800	0.89716100	3.30765900
C	-4.72608000	0.50459800	2.98206100
C	1.35766600	-2.01674900	-1.26275900
C	1.40586300	-3.40485200	-1.08752600
C	1.39230000	-1.48604200	-2.55510600
C	1.51652500	-4.24244700	-2.19171000
C	1.48755300	-2.32699600	-3.65969000
C	1.55551600	-3.70512500	-3.47685100
C	-1.82781000	-0.64713700	-1.31172500
C	-2.18045100	-0.61654200	-2.65557700
C	-1.63291700	-1.87270900	-0.65810300
C	-2.32557100	-1.84413500	-3.31189400
C	-1.82846800	-3.02786000	-1.43643900
P	-1.57043900	0.90470900	-0.35519800
C	-2.16576000	2.24566800	-1.44202600
C	-1.43721900	2.53804400	-2.60672300
C	-3.26957400	3.03383100	-1.11156300
C	-1.83600500	3.57236600	-3.44265500
C	-3.65958500	4.07609700	-1.94811500
C	-2.95331400	4.34023400	-3.11505600
Rh	0.67108400	1.40104800	-0.17873400
C	3.25905800	1.59969700	-1.27565200
C	2.34947800	2.42590400	-1.37098000
C	0.43716100	2.84368400	1.35628400
C	0.19913700	3.50896600	0.31292600
H	3.53394800	-2.83196300	0.08321900
H	2.42724500	0.72695300	2.23554900
H	5.52550200	-3.06798000	1.50765500
H	4.42558800	0.47801500	3.67284700
H	5.96867400	-1.44167000	3.33116300
H	1.17398500	-1.37222100	3.09393000
H	-4.35990800	0.10459600	-0.37361200
H	-6.06898600	-0.08622900	1.40018200
H	-3.16642400	1.10791300	4.34251900
H	-5.48111600	0.41287200	3.76111300
H	1.33534700	-3.83209900	-0.08642700
H	1.33112000	-0.40366500	-2.69299700
H	1.56254100	-5.32096200	-2.04839400
H	1.50136400	-1.90571400	-4.66353700
H	1.63487800	-4.36499900	-4.33911000
H	-2.35972000	0.30106600	-3.20732000
H	-0.55691600	1.94396800	-2.86341600
H	-3.82521100	2.84438100	-0.19518200
H	-1.27553700	3.77930800	-4.35285700
H	-4.51788100	4.68740100	-1.67835500
H	-3.26766700	5.15067100	-3.77034500
C	-2.47141300	1.02789300	2.30973600
H	-1.45587600	1.30965500	2.56844600
C	-0.21078100	4.72169700	-0.38398900
O	-1.22407700	5.32158500	-0.12807900
O	0.66297900	5.06166800	-1.34327800
C	0.72068100	2.77919800	2.79321000
O	1.07136000	3.73806000	3.43221000
O	0.55571300	1.54376300	3.28394100
C	0.33370600	6.26059000	-2.05330400
H	0.27463600	7.10639200	-1.36156100
H	1.13837400	6.40939000	-2.77462000
H	-0.63005000	6.14234400	-2.56106500
C	0.80812600	1.38496100	4.68350900
H	0.47640900	0.37209600	4.92863400
H	1.87923500	1.50178900	4.88588000
H	0.25438900	2.13245800	5.26036100
C	4.37535000	0.72429700	-1.20394400
C	4.57477100	-0.27045700	-2.17531300
C	5.34008400	0.92072000	-0.20232700
C	5.71647700	-1.05687700	-2.13648600
H	3.83327500	-0.41040100	-2.95958200
C	6.47816500	0.13027500	-0.17350500
H	5.18332300	1.70136700	0.53994200
C	6.66765900	-0.85729000	-1.13815900
H	5.87078000	-1.82266900	-2.89378000
H	7.22417000	0.28601100	0.60332700
H	7.56584700	-1.47180200	-1.11516200
H	1.94068000	3.37198600	-1.69273300
N	-2.01645000	-2.43332200	3.02269100
N	-2.16708900	-3.01869100	-2.71871900
O	-0.62112700	-1.98555100	4.79582000
O	-3.40956400	-2.78428900	1.23617200
O	-1.63209000	-4.20232800	-0.82969600
O	-2.63749700	-1.80033300	-4.60964500
C	-1.69787000	-2.36748400	5.64094300
H	-1.98181700	-3.41153600	5.46684200
H	-1.33131100	-2.24162200	6.66183500
H	-2.57544600	-1.73189200	5.46775100
C	-4.38629300	-3.20581700	2.17581000
H	-5.27140700	-3.45605800	1.58611800
H	-4.04197100	-4.08426500	2.73355200
H	-4.61810000	-2.40177300	2.88609100
C	-1.83706300	-5.37622000	-1.60046100
H	-1.16264100	-5.39966200	-2.46604700
H	-1.62327400	-6.21029200	-0.92811100
H	-2.87099300	-5.43517100	-1.95937300
C	-2.73863300	-3.03889200	-5.29692300
H	-2.97557600	-2.78320400	-6.33169600
H	-1.79097400	-3.58994700	-5.24732800
H	-3.53146500	-3.66368900	-4.86982100

IM1d

P	0.11457900	-0.64546000	-1.32564400	H	0.36983300	-2.20484300	2.85928300
C	-1.04354100	-0.93716800	-2.70493100	H	-0.11350200	1.84465300	4.27677900
C	-0.75280000	-1.77486300	-3.78318900	H	-0.15898400	-3.05092500	5.11497900
C	-2.30360100	-0.33073500	-2.62938000	H	-0.65844900	0.98069300	6.52553300
C	-1.70410000	-1.99181000	-4.77480400	H	-0.69219400	-1.46330000	6.95196300
C	-3.24664600	-0.54138900	-3.62628000	C	-0.65317000	2.91559500	0.95636700
C	-2.94886200	-1.37633000	-4.70019700	H	-1.38245100	2.33183600	0.39407000
C	0.75385700	1.06381300	-1.57411900	C	-0.68180200	-4.06319500	-0.41874600
C	1.87550800	1.48567300	-0.84353800	O	-0.06411500	-4.83375500	0.27435300
C	0.07307900	1.95827700	-2.39095100	O	-0.74400800	-4.09182500	-1.74987900
C	2.23627700	2.83926000	-0.98936000	C	-3.90198700	-2.51565300	1.12079200
C	0.54632000	3.27395600	-2.44406500	O	-4.14220400	-2.83786400	2.25850100
C	0.33314200	2.26178800	1.69536800	O	-4.81151900	-2.09345600	0.23591400
C	1.30212100	3.00992500	2.37494800	C	-6.14462600	-2.00435900	0.75179800
C	1.26524800	4.39832800	2.32658300	H	-6.17598800	-1.30015900	1.59133000
C	-0.67688700	4.30504600	0.89634000	H	-6.75952800	-1.64621800	-0.07589300
C	0.27909200	5.04624100	1.58423700	H	-6.49009400	-2.98499200	1.09226600
C	1.59466100	-1.67406800	-1.61983200	C	0.03278800	-5.11222000	-2.38301800
C	2.35611700	-1.47858500	-2.77913100	H	-0.19624200	-6.09250000	-1.95464300
C	1.99730400	-2.62902700	-0.68540800	H	-0.24370500	-5.07844000	-3.43878700
C	3.48227600	-2.25981100	-3.01105600	H	1.10195500	-4.89877700	-2.25606800
C	3.13194500	-3.40144200	-0.91364400	H	-2.53234800	-0.69595200	3.12864400
C	3.87073000	-3.22154700	-2.07972500	C	-3.96904900	1.56083000	0.62631100
C	2.16320900	0.10333600	1.26683400	C	-4.69229400	1.09390900	-0.48490100
C	2.95575100	-0.70860300	2.06778600	C	-3.97894100	2.93136600	0.93608900
C	2.65616200	0.61044800	0.05647600	C	-5.39281900	1.99145300	-1.27801700
C	4.24558900	-1.00084800	1.60908800	H	-4.68984700	0.02682700	-0.70707900
C	3.97232800	0.23922800	-0.27735700	C	-4.69098600	3.81707100	0.14050600
P	0.41364300	0.44104200	1.70248400	H	-3.42818000	3.28325200	1.80700200
C	0.19803300	-0.11636000	3.42376400	C	-5.39320600	3.35025700	-0.96850700
C	0.17064600	-1.49864200	3.66854000	H	-5.95517500	1.63020900	-2.13720800
C	-0.10624400	0.77101100	4.45870100	H	-4.70387800	4.87647400	0.38911000
C	-0.13931000	-1.97827500	4.93452600	H	-5.94969000	4.04802100	-1.59107700
C	-0.42085900	0.28370700	5.72429800	N	1.59995100	3.70521800	-1.76593500
C	-0.43890400	-1.08644600	5.96309700	N	4.74285300	-0.53776700	0.47110800
Rh	-1.13792200	-0.88248100	0.59239800	O	-0.12732000	4.12789800	-3.22065700
C	-1.47198600	-2.95095900	0.11952800	O	4.99086600	-1.80233400	2.37363100
C	-2.55698100	-2.49264300	0.56021600	O	4.44742000	0.68822800	-1.44268500
C	-3.27129800	0.65632800	1.47497700	O	3.27652000	3.26452400	-0.26639000
C	-2.68336200	-0.10980600	2.23716200	C	0.33390000	5.46980200	-3.26348600
H	0.21304900	-2.27282200	-3.84676100	H	1.35665600	5.52294500	-3.65432900
H	-2.54565800	0.31461000	-1.78154500	H	-0.35235500	5.99655200	-3.92983400
H	-1.46933400	-2.64814200	-5.61083000	H	0.31806400	5.92261600	-2.26387400
H	-4.21867200	-0.05470400	-3.56131000	C	3.62316700	4.63723200	-0.36158400
H	-3.68976900	-1.54971300	-5.47818700	H	2.79647100	5.27219600	-0.01530500
H	-0.79399000	1.68245200	-2.98409600	H	4.49374400	4.76509800	0.28592100
H	2.09268600	2.50470800	2.93179800	H	3.87350800	4.91154100	-1.39252700
H	2.01720800	4.97773400	2.85962400	C	5.77163800	0.31683200	-1.79262000
H	-1.44092400	4.80461700	0.30110800	H	5.95141000	0.75971200	-2.77487700
H	0.26075300	6.13405400	1.54022500	H	6.49361200	0.70401900	-1.06440000
H	2.07599600	-0.70496200	-3.49553100	H	5.87135500	-0.77462100	-1.84044000
H	1.43210200	-2.76127600	0.23594600	C	6.28767700	-2.13536800	1.90061000
H	4.06613800	-2.10891000	-3.91754200	H	6.91162900	-1.24000900	1.79793100
H	3.43411200	-4.14174400	-0.17448700	H	6.71096400	-2.80712500	2.65006300
H	4.75655100	-3.82769700	-2.26252700	H	6.23225300	-2.63787500	0.92660300
H	2.62349100	-1.11806800	3.01735500				

TS1a

C	-1.83293700	1.07598200	2.37411100	C	-4.09192500	2.66367100	0.33905600
C	-2.94692400	0.98652000	1.73304100	C	-6.57234500	1.76474700	1.25480600
C	-4.16391400	1.61979900	1.27027200	C	-5.25801500	3.24979900	-0.13011500
C	-5.41043400	1.16352500	1.71858400	C	-6.49582400	2.80153400	0.32655700

C	-2.12073100	-1.60908900	1.28686200
C	-3.00428100	-0.77102400	0.82992300
C	-4.07203000	-0.82051900	-0.19415900
O	-4.06053100	-0.13148800	-1.18473000
O	-5.00729600	-1.71188800	0.12385200
C	-6.08842400	-1.79027300	-0.81127900
C	-1.79677500	-2.91560200	1.85587900
O	-0.75400800	-3.17971200	2.41310200
O	-2.79975600	-3.76993400	1.65956700
C	-2.58851300	-5.08791300	2.18085800
P	1.21664700	0.72888700	1.53329100
C	1.55175700	0.43753000	3.29995700
C	2.20648100	1.37235700	4.10645300
C	1.14481400	-0.78640200	3.84482700
C	2.45782800	1.08124500	5.44359000
C	1.41123900	-1.07471900	5.17813100
C	2.06580200	-0.14174300	5.97817500
C	2.65125600	0.01741600	0.63050500
C	2.74396500	0.18464500	-0.76215000
C	3.58196800	-0.75778500	1.30753100
C	3.82714200	-0.45018600	-1.39068600
C	4.59411600	-1.35686200	0.54779000
C	1.11771500	-2.27778100	-1.32340200
C	1.62811700	-2.59458500	-2.58810900
C	1.53221300	-2.99757800	-0.20117000
C	2.56567100	-3.60954500	-2.72033800
C	2.47770500	-4.01104800	-0.34012800
C	2.99528400	-4.31298500	-1.59514500
C	-1.44068200	-1.62243900	-2.33055600
C	-1.66764900	-1.10630000	-3.60808900
C	-2.18298700	-2.72852000	-1.89800200
C	-2.64812000	-1.66487400	-4.42203800
C	-3.15553400	-3.28841200	-2.71656800
C	-3.39815900	-2.74816600	-3.97692900
C	1.40889900	2.52766300	1.25195500
C	2.68128700	3.11405100	1.25564100
C	0.28746000	3.33128000	1.02765300
C	2.81993800	4.48298400	1.05976800
C	0.43089300	4.69852700	0.81477000
C	1.69618100	5.27607600	0.83555600
C	0.44483200	0.60705100	-1.76638900
C	-0.49037500	1.53731000	-2.22924500
C	1.77159900	0.99656300	-1.52735600
C	-0.02731400	2.82659700	-2.50742400
C	2.11269400	2.29854200	-1.94559900
P	-0.17715000	-1.00490900	-1.16626000
Rh	-0.96902800	-0.03142700	0.86168300
H	-1.45179300	1.65129600	3.21026000
H	-5.45261100	0.33887900	2.42935600
H	-3.11866300	2.98323400	-0.03484500
H	-7.54099400	1.42446200	1.61540100
H	-5.20472000	4.05373400	-0.86103900
H	-7.40863000	3.26344800	-0.04478800
H	-6.77110700	-2.54178300	-0.41236800
H	-6.58479500	-0.81629800	-0.89134600
H	-5.71470800	-2.08550300	-1.79833500
H	-1.70321900	-5.54082200	1.72291000
H	-2.45126200	-5.04677800	3.26590500
H	-3.48711200	-5.65124200	1.92736100
H	2.51524200	2.33350600	3.69817200
H	0.61324300	-1.51427100	3.22857100
H	2.96235800	1.81516500	6.06901700
H	1.09759200	-2.02996700	5.59455500
H	2.26603400	-0.36668300	7.02410500
H	3.54734400	-0.92546300	2.38105300
H	1.29402300	-2.04142300	-3.46691100
H	1.11915100	-2.76503400	0.78190600
H	2.96033600	-3.85774800	-3.70440200
H	2.80941800	-4.56297700	0.53774800
H	3.72956500	-5.10982300	-1.70247500
H	-1.08049400	-0.26620000	-3.97524900
H	-1.99689000	-3.16768700	-0.91651100
H	-2.82067500	-1.25249500	-5.41425800
H	-3.72267700	-4.15063100	-2.36897500
H	-4.16154300	-3.18293600	-4.61957300
H	3.56718800	2.49408100	1.39943300
H	-0.70745900	2.88556000	1.01844200
H	3.81205300	4.93162500	1.07264300
H	-0.45071300	5.31280500	0.63779700
H	1.80956300	6.34743100	0.67838400
H	-1.54674000	1.30374400	-2.34979100
N	4.72258500	-1.20006800	-0.76183700
N	1.24717000	3.18309500	-2.42082600
O	5.46101400	-2.13253800	1.20372000
O	3.92139200	-0.30167500	-2.71580400
O	3.39398500	2.65208600	-1.81807200
O	-0.93731100	3.73370700	-2.87881800
C	-0.46702000	5.04227000	-3.16920600
H	0.23198300	5.03020800	-4.01357400
H	-1.35498200	5.62468100	-3.42401100
H	0.04161100	5.47885500	-2.30039200
C	3.75897900	3.95290900	-2.25448100
H	3.19971500	4.72170900	-1.70660900
H	4.82853400	4.04118600	-2.04998400
H	3.56827200	4.07303300	-3.32721200
C	5.00424500	-0.94842100	-3.36656300
H	4.88788300	-0.72067200	-4.42853800
H	5.96554700	-0.56872000	-3.00134300
H	4.96898200	-2.03259200	-3.20069900
C	6.48171400	-2.75960100	0.44196300
H	7.12415900	-2.01593100	-0.04384200
H	7.06070100	-3.35021000	1.15497100
H	6.05163700	-3.40921200	-0.33054600

TS1b

N	4.37933100	-0.95651100	1.98762500
C	3.85689600	-1.24709800	0.80437400
C	2.81837000	-0.51998100	0.19124700
C	2.35914900	0.60420200	0.89409500
C	2.89145800	0.92199600	2.13882200
C	3.89521300	0.08864900	2.64325600
C	2.28035500	-0.95978800	-1.11519100
C	3.05462800	-0.80315000	-2.28084200
N	2.65557300	-1.13886700	-3.49945200
C	1.44989000	-1.67269300	-3.63479300
C	0.58912500	-1.92281000	-2.56089500
C	1.02082500	-1.55671200	-1.29155200
P	-0.12312700	-1.68837200	0.14247000
P	0.93847200	1.57042900	0.25034300
O	4.31029600	-2.31867100	0.14668900
C	5.35733500	-3.06077600	0.75273500
O	4.26389900	-0.25245400	-2.13021300
C	5.05849900	-0.09582700	-3.29568400

O	1.00703600	-1.99376800	-4.85238400
C	1.84684900	-1.67928700	-5.95361800
O	4.36917600	0.37644900	3.85846000
C	5.36163900	-0.48881800	4.39049600
C	1.42249000	2.01665700	-1.45188800
C	0.46962800	1.94387500	-2.47276700
C	0.81093000	2.27838300	-3.77809000
C	2.10516200	2.69999100	-4.07087300
C	3.06130200	2.77087200	-3.06105800
C	2.72861500	2.41547700	-1.75854100
C	0.86553200	-2.46817500	1.46034200
C	1.56539400	-3.65106500	1.18637000
C	2.30232200	-4.27231300	2.18694700
C	2.36763500	-3.70798200	3.45970300
C	1.69624300	-2.52025700	3.73101600
C	0.94301800	-1.90528400	2.73586700
Rh	-1.16569300	0.43111200	0.32883500
C	-2.01134800	-0.15263700	2.06192900
C	-2.29811700	2.23683600	0.15648500
C	-3.14595300	1.28619400	-0.00542300
C	-3.13659500	-0.20368300	1.45370900
C	-2.06207800	3.66992500	0.06595100
O	-2.60309900	4.48703500	0.76985400
O	-1.13695000	3.93981100	-0.86418700
C	-0.75202400	5.30971900	-0.97471400
H	2.56363100	1.77615900	2.72555400
H	-0.37030300	-2.39447900	-2.75154500
H	5.56519900	-3.88726800	0.06929700
H	6.25311600	-2.44262700	0.88255400
H	5.04904500	-3.44368000	1.73402600
H	5.99062000	0.36189100	-2.95588000
H	5.26215200	-1.06460000	-3.76597800
H	4.55835800	0.55294500	-4.02617800
H	1.29501300	-1.99001900	-6.84306700
H	2.05344000	-0.60203800	-5.98918600
H	2.79908200	-2.21840500	-5.89038100
H	5.58727200	-0.10174500	5.38626300
H	4.98739900	-1.51844100	4.45612200
H	6.26401800	-0.48351100	3.76821200
H	-0.55142300	1.63045100	-2.24569300
H	0.06370200	2.21282700	-4.56656800
H	2.37081200	2.97194400	-5.09101800
H	4.07636000	3.09263000	-3.28911100
H	3.49848700	2.41741100	-0.98645500
H	1.54177600	-4.07944200	0.18344500
H	2.83601500	-5.19609700	1.96964000
H	2.95042300	-4.19406700	4.24029300
H	1.76392800	-2.06397700	4.71719300
H	0.44922300	-0.95668300	2.94190800
H	0.08154100	5.32055500	-1.68245100
H	-1.58517700	5.91334900	-1.34957800
H	-0.43587300	5.69483500	0.00168900
C	-1.40015200	-2.90577700	-0.33193700
C	-1.49529700	-4.18326000	0.22964100
C	-2.36241500	-2.49606200	-1.26419800
C	-2.51503300	-5.04470400	-0.16776000
H	-0.78039700	-4.51204900	0.98133300
C	-3.37550700	-3.35967000	-1.66019500
H	-2.35066900	-1.48197500	-1.66522700
C	-3.44674300	-4.64051400	-1.11929800
H	-2.57766500	-6.03864700	0.27129500
H	-4.12021600	-3.01269400	-2.37346100
H	-4.24018400	-5.31943000	-1.42672300
C	0.95738400	3.09311300	1.26069700
C	1.85509600	4.13443100	1.00444200
C	0.09982500	3.18415400	2.36035100
C	1.89011600	5.24738300	1.83655600
H	2.52160000	4.08834000	0.14473100
C	0.13464600	4.30029700	3.18992400
H	-0.61572900	2.38295400	2.55597800
C	1.03066000	5.33180300	2.92907300
H	2.58935900	6.05481400	1.62754400
H	-0.54866800	4.36774800	4.03361900
H	1.05577500	6.20693100	3.57528600
C	-4.27060100	0.92223300	-0.89818900
O	-4.32784000	-0.09230200	-1.55098600
O	-5.16553100	1.90697000	-0.92451600
C	-6.29286900	1.67405700	-1.77490600
H	-6.91394500	2.56672500	-1.69168900
H	-5.96898700	1.52211700	-2.80929700
H	-6.83781800	0.78667900	-1.43283100
C	-4.45625500	-0.79727700	1.43321900
C	-5.60096100	0.00253800	1.56043400
C	-4.58624800	-2.18644000	1.31337200
C	-6.85847100	-0.58652200	1.55518400
H	-5.49528100	1.07995700	1.67967000
C	-5.84771500	-2.76383900	1.29637900
H	-3.69158000	-2.80182800	1.22849400
C	-6.98373200	-1.96682200	1.41236100
H	-7.74514000	0.03396900	1.67047100
H	-5.94216800	-3.84365000	1.19494300
H	-7.97152800	-2.42359900	1.40162700
H	-1.52754100	-0.23344600	3.02702300

TS1c

P	-0.71246300	-1.51267900	0.11335200
C	-2.04994400	-2.56413200	-0.56288900
C	-2.14517200	-3.93152700	-0.28780700
C	-3.01855400	-1.96465800	-1.37367400
C	-3.18504500	-4.68294200	-0.82391700
C	-4.05559500	-2.71783200	-1.91048000
C	-4.14032000	-4.07907900	-1.63547900
C	0.68446200	-1.71354300	-1.05997700
C	1.99638600	-1.38487200	-0.68331700
C	0.39602900	-2.05731100	-2.37583100
C	2.97233500	-1.50024100	-1.69445800
C	1.45351100	-2.06388400	-3.28769400
C	2.07236300	1.63872500	-1.41196500
C	3.44384100	1.91033800	-1.31728200
C	4.20608700	2.04664200	-2.47051500
C	2.25668900	1.60513700	-3.82055600
C	3.61335900	1.89468000	-3.72333100
C	-0.16834400	-2.30432300	1.66658700
C	0.48001500	-3.54530600	1.68340100
C	-0.42212500	-1.65017200	2.87676800
C	0.83026700	-4.13285500	2.89334200
C	-0.05393900	-2.23170300	4.08602500
C	0.56515400	-3.47769600	4.09394200
C	2.09903600	0.36561600	1.15817200
C	2.51036100	0.74085400	2.43099300
C	2.39025100	-0.91732400	0.66579400
C	3.21730800	-0.20178400	3.18526400
C	3.13556900	-1.74868200	1.52137300

P	1.08317700	1.48654900	0.11387100
C	1.15650600	3.13006700	0.91357200
C	0.49138700	3.30366100	2.13778700
C	1.75880100	4.23392400	0.30652100
C	0.46616600	4.54596500	2.75724200
C	1.72082900	5.48131100	0.92419400
C	1.08631700	5.63778200	2.15091700
Rh	-1.26015700	0.82619700	0.35845200
C	-3.24935700	0.57161100	1.08610300
C	-2.96916100	1.81639600	1.22597800
C	-1.81654800	1.91425700	-1.26244800
C	-1.98644500	2.73755500	-0.27732200
H	-1.42167800	-4.41770400	0.36315100
H	-2.96322400	-0.89572300	-1.57227200
H	-3.25025800	-5.74625400	-0.60100900
H	-4.80860700	-2.23559700	-2.53137200
H	-4.95618400	-4.66933300	-2.04843400
H	-0.60781400	-2.30887700	-2.70967100
H	3.91453700	2.01270200	-0.33851200
H	5.26995200	2.26434400	-2.39178100
H	1.79246900	1.47211900	-4.79689600
H	4.21473000	2.00087600	-4.62468400
H	0.73894800	-4.04017900	0.74709400
H	-0.91034600	-0.67107100	2.87457800
H	1.32099600	-5.10502100	2.89808100
H	-0.25309500	-1.71167600	5.02096100
H	0.84493100	-3.93999500	5.03888400
H	2.32332100	1.72417600	2.85261300
H	-0.00754100	2.45541500	2.61318700
H	2.25033800	4.12967700	-0.65863000
H	-0.04030600	4.66386700	3.71368600
H	2.19054600	6.33412200	0.43866100
H	1.06921100	6.61226600	2.63631700
C	1.48640400	1.48271600	-2.66838400
H	0.43617700	1.21666600	-2.74664800
C	-1.93457600	4.20896100	-0.10357800
O	-1.37011900	4.91721800	-0.89730500
O	-2.55171300	4.63399000	1.00063400
C	-2.04607300	1.67685600	-2.68558600
O	-2.52327000	2.51501200	-3.40963000
O	-1.63253300	0.46159100	-3.06313300
C	-2.50733200	6.05099000	1.20243700
H	-2.99581500	6.56552300	0.36925500
H	-3.04094300	6.23413500	2.13613200
H	-1.46615400	6.38439800	1.27431800
C	-1.70572400	0.18369100	-4.46523400
H	-1.08639500	-0.70277800	-4.62657200
H	-2.74352300	-0.00883200	-4.75809200
H	-1.32496200	1.03545900	-5.03900500
C	-4.26579300	-0.43779200	1.22171700
C	-4.02144800	-1.69154500	1.79733400
C	-5.54884300	-0.14776200	0.72844800
C	-5.04015600	-2.62928800	1.88365700
H	-3.03171300	-1.92513100	2.18939200
C	-6.55683500	-1.09822600	0.80139300
H	-5.73718600	0.82833800	0.28348100
C	-6.30535800	-2.34127500	1.37720400
H	-4.84036800	-3.59796800	2.33796900
H	-7.54610700	-0.86516100	0.41235200
H	-7.09777800	-3.08479900	1.43616600
H	-3.10635900	2.73583200	1.78126400
N	2.71348600	-1.82607400	-2.95349900
N	3.53177100	-1.41010000	2.74059800
O	1.15004500	-2.32007800	-4.56745000
O	4.23327700	-1.22955200	-1.34589700
O	3.43510100	-2.96979500	1.06480300
O	3.58143000	0.15980900	4.41806000
C	2.22332600	-2.33415700	-5.49860400
H	2.94918800	-3.11628100	-5.24831000
H	1.76878000	-2.53896300	-6.47012300
H	2.74220900	-1.36743100	-5.51484600
C	5.22668800	-1.33124100	-2.35451700
H	6.16649900	-1.05830800	-1.86875100
H	5.28565800	-2.35327100	-2.74615400
H	5.01335100	-0.64492700	-3.18392700
C	4.20342100	-3.81117800	1.91068600
H	3.68038300	-3.99869400	2.85732000
H	4.33493100	-4.74367900	1.35676000
H	5.17840800	-3.36200300	2.13179500
C	4.26477000	-0.80501600	5.20455400
H	4.44332900	-0.32587600	6.16933900
H	3.65304200	-1.70666900	5.33358500
H	5.21648000	-1.08933600	4.74092900

TS1d

P	-0.50551200	0.98443100	1.12194300
C	-1.44704200	0.94244600	2.68744800
C	-1.80186700	2.08678200	3.40497400
C	-1.82623000	-0.31427000	3.17379100
C	-2.51787400	1.97287300	4.59143600
C	-2.53530800	-0.42557000	4.36398300
C	-2.88345600	0.71985100	5.07350000
C	1.24168700	0.72224400	1.62656000
C	2.25932800	0.94521700	0.68824100
C	1.54166600	0.24925100	2.89794400
C	3.57003900	0.66713100	1.12352000
C	2.88980000	0.01944200	3.19528800
C	2.13400300	-1.81402600	-0.58984700
C	3.31023600	-1.81424400	-1.35067500
C	4.45081400	-2.44233000	-0.86487500
C	3.27197100	-3.03350700	1.15582000
C	4.43134000	-3.05503500	0.38661300
C	-0.47656100	2.70323600	0.50105700
C	0.11644800	3.71148600	1.27199600
C	-0.99616000	3.01891200	-0.75578600
C	0.13636400	5.02225400	0.80878300
C	-0.96497300	4.32880600	-1.22222800
C	-0.40723800	5.33323100	-0.43605900
C	1.33940600	0.69709600	-1.67008100
C	1.13933700	1.23426200	-2.93509200
C	2.01267500	1.43536900	-0.68416200
C	1.62782100	2.52398700	-3.17448000
C	2.44725700	2.72047400	-1.06403700
P	0.65585000	-0.95658600	-1.23192900
C	0.22386400	-1.74103400	-2.82492500
C	-0.91023000	-1.26705500	-3.50082000
C	0.93238300	-2.82129400	-3.35709900
C	-1.31333400	-1.85009500	-4.69431900
C	0.51793700	-3.40969200	-4.54975800
C	-0.59995300	-2.92556300	-5.21935700
Rh	-1.40253700	-0.72578400	-0.16714800
C	-3.13584900	0.44791500	-0.32253100
C	-3.62918800	-0.73027500	-0.37937800

C	-1.51646100	-2.94425000	-0.17092700
C	-2.59839500	-2.62751700	-0.73246800
H	-1.53812400	3.07447600	3.03109000
H	-1.56200100	-1.21112600	2.60821200
H	-2.79234300	2.87059200	5.14246700
H	-2.82157100	-1.40795500	4.73448600
H	-3.44370900	0.63579600	6.00263800
H	0.78765800	0.06440300	3.65812700
H	3.33875800	-1.31074800	-2.31797000
H	5.36178100	-2.44344100	-1.46098600
H	3.25369900	-3.49650300	2.14148500
H	5.32734800	-3.54483700	0.76465800
H	0.58401100	3.46947800	2.22726000
H	-1.40654700	2.23418000	-1.38997200
H	0.59227500	5.80173200	1.41700700
H	-1.37603300	4.55760400	-2.20403800
H	-0.38656900	6.36026600	-0.79685300
H	0.63593200	0.70271800	-3.73716500
H	-1.48653100	-0.43832300	-3.08386100
H	1.80315900	-3.21767800	-2.83690300
H	-2.19016000	-1.46798600	-5.21304100
H	1.07588600	-4.25142100	-4.95558100
H	-0.91942400	-3.38769700	-6.15129000
C	2.12683400	-2.41409400	0.66854800
H	1.22786300	-2.37591000	1.27995900
C	-3.66761900	1.81781500	-0.30513500
O	-4.06325200	2.37127500	-1.30125700
O	-3.65365700	2.33252100	0.92160000
C	-5.00546600	-1.27798600	-0.41192300
O	-5.35378700	-2.34450500	-0.86313200
O	-5.83318600	-0.37104500	0.10164600
C	-7.22207200	-0.72226200	0.05587300
H	-7.39859800	-1.64233900	0.62145400
H	-7.75050300	0.11771600	0.50788800
H	-7.54103900	-0.86852100	-0.98079400
C	-4.09255900	3.68953600	1.02206100
H	-5.08620600	3.80733700	0.57977000
H	-4.11666600	3.91129400	2.09082200
H	-3.38284900	4.34848200	0.50561500
H	-3.53162200	-2.92510300	-1.19654400
C	-0.62803200	-4.00219100	0.25474900
C	-0.44266400	-4.24789500	1.62162000
C	-0.00794200	-4.83212000	-0.68725000
C	0.35570500	-5.30392300	2.03780400
H	-0.93798000	-3.60680600	2.35104600
C	0.80215200	-5.87784900	-0.26242800
H	-0.17710400	-4.65192400	-1.74818400
C	0.98667000	-6.11541600	1.09667600
H	0.48391500	-5.49825000	3.10099700
H	1.28086500	-6.52098700	-0.99829000
H	1.61454300	-6.94161500	1.42437000
N	3.87756300	0.22974800	2.33703300
N	2.26884300	3.24706300	-2.26753100
O	3.16934500	-0.44445000	4.41647200
O	1.41336600	3.03413200	-4.38942100
O	3.05237400	3.45301900	-0.12465800
O	4.55041900	0.84138000	0.23392100
C	4.53325800	-0.69568700	4.72130700
H	5.12707200	0.22340200	4.65610800
H	4.54105200	-1.07865600	5.74383800
H	4.95918200	-1.43670500	4.03319200
C	5.87316100	0.54247100	0.65180800
H	5.95832400	-0.51010600	0.95032600
H	6.50716200	0.74342600	-0.21481400
H	6.17189300	1.17479400	1.49565800
C	3.48747900	4.75365000	-0.48906900
H	3.94686000	5.17174600	0.40965600
H	4.21924400	4.71031200	-1.30376700
H	2.64000200	5.37400100	-0.80747900
C	1.87298400	4.35554900	-4.63270500
H	2.96214900	4.41945100	-4.52693500
H	1.57870800	4.58552100	-5.65866600
H	1.41065300	5.06560200	-3.93534600

IM2a

C	-2.34715400	0.70936000	-1.24781000
C	-3.47888500	0.09592600	-0.84278300
C	-4.81253100	0.69073000	-1.05083000
C	-5.70489500	0.81751300	0.02019600
C	-5.16739200	1.22067300	-2.29408600
C	-6.91927600	1.46763500	-0.14982700
C	-6.38743100	1.86446700	-2.46519600
C	-7.26531300	1.99069300	-1.39356400
C	-1.94055600	-1.48011100	0.06046600
C	-3.23604500	-1.16074200	-0.10804700
C	-4.31734800	-2.10080400	0.28899000
O	-5.38790500	-2.18941300	-0.25850000
O	-3.93622700	-2.87184000	1.32473800
C	-4.87061300	-3.88438600	1.69654700
C	-1.36208100	-2.68817400	0.69533600
O	-0.84503800	-2.71464100	1.79286500
O	-1.41578500	-3.73434500	-0.13257000
C	-0.84437200	-4.94462800	0.36721300
P	-0.52202600	1.19090300	0.79009300
C	-1.88867100	1.19472100	1.97959700
C	-2.79366200	2.26266300	2.00419500
C	-2.11492500	0.07084000	2.78485800
C	-3.89077400	2.22252600	2.85622500
C	-3.21589500	0.04043400	3.63247700
C	-4.09900000	1.11604200	3.67491200
C	1.05641700	0.87698300	1.67425500
C	2.25420300	0.90788100	0.93561200
C	1.07652100	0.67985700	3.04848600
C	3.43801300	0.79481800	1.69048500
C	2.32786400	0.51800100	3.65639000
C	2.78034200	-2.15366500	0.33047800
C	4.14134700	-2.00773800	0.03408300
C	2.38721000	-2.76631700	1.52513700
C	5.09771700	-2.49415100	0.91699700
C	3.35293600	-3.23760400	2.41002600
C	4.70415800	-3.10755400	2.10499100
C	1.50930200	-2.68891900	-2.27377700
C	2.65148300	-3.32499800	-2.76923400
C	0.28787200	-2.85695200	-2.93734300
C	2.57164500	-4.10057700	-3.91968000
C	0.21162400	-3.62189800	-4.09589800
C	1.35631900	-4.24330900	-4.58572500
C	-0.31527300	2.90410800	0.19958500
C	-0.10074400	3.90364800	1.15716300
C	-0.28937800	3.23272300	-1.15660000
C	0.08785800	5.22088500	0.75799600
C	-0.09094200	4.55135600	-1.55255300
C	0.08327800	5.54682700	-0.59631200

C	2.09446200	0.04245600	-1.45985700	H	3.46278300	-4.59547600	-4.30057300
C	2.15456600	0.29551400	-2.82971900	H	-0.74172500	-3.74375600	-4.60575300
C	2.33874700	1.07921500	-0.53373000	H	1.30009100	-4.85039800	-5.48716300
C	2.53898300	1.58161200	-3.23174500	H	-0.08908800	3.65310400	2.21862600
C	2.75875700	2.30791200	-1.08314800	H	-0.41318500	2.45751500	-1.91259400
P	1.50158400	-1.58111200	-0.82997500	H	0.23913100	5.99500200	1.50752000
Rh	-0.61597700	-0.31745200	-0.86914100	H	-0.07402500	4.79827900	-2.61271300
H	-2.37370700	1.67143800	-1.76668100	H	0.22502900	6.58060000	-0.90653400
H	-5.43064500	0.42077000	0.99887600	H	1.96888100	-0.47048400	-3.57920100
H	-4.48486300	1.10325000	-3.13575000	N	3.47689800	0.59394600	2.99975600
H	-7.60371000	1.56304500	0.69141400	N	2.85231900	2.55324800	-2.38421600
H	-6.65704800	2.26187400	-3.44206400	O	2.33403200	0.28699700	4.96904000
H	-8.22259500	2.49098700	-1.52767200	O	4.58883700	0.88303200	1.01717300
H	-4.41522400	-4.41771500	2.53313900	O	3.05804200	3.27736800	-0.21885500
H	-5.82176500	-3.43468600	1.99929300	O	2.59586500	1.81151700	-4.54329500
H	-5.05417800	-4.56401700	0.85740800	C	2.99620500	3.10750200	-4.96766100
H	0.22494500	-4.80756300	0.57028800	H	4.01255400	3.33511700	-4.62663700
H	-1.35064100	-5.24981500	1.28937000	H	2.95897500	3.08381200	-6.05834500
H	-0.98868900	-5.68785000	-0.41824100	H	2.31428500	3.87419300	-4.57987200
H	-2.65307500	3.12159100	1.35024000	C	3.52353600	4.51201500	-0.74583300
H	-1.45422300	-0.79557400	2.73291200	H	2.77559400	4.96035800	-1.41061300
H	-4.59086800	3.05535500	2.86935300	H	3.68970100	5.15463700	0.12170200
H	-3.38570700	-0.83524900	4.25544100	H	4.45848600	4.37185500	-1.30052100
H	-4.95967900	1.08750600	4.34068700	C	5.79167500	0.78981000	1.76679000
H	0.18065300	0.65483300	3.66132400	H	6.59938300	0.86499800	1.03483700
H	4.45447500	-1.49205400	-0.87461600	H	5.86336300	1.60612700	2.49462600
H	1.32880900	-2.85605800	1.77293100	H	5.84676300	-0.16726800	2.30026600
H	6.15543800	-2.38759900	0.68041300	C	3.59523500	0.08492100	5.59182800
H	3.04498600	-3.70883600	3.34159100	H	4.22799000	0.97478700	5.49650500
H	5.45690200	-3.48489900	2.79512000	H	3.37554800	-0.11217400	6.64289200
H	3.60400600	-3.22106800	-2.25078900	H	4.11747500	-0.76966200	5.14426500
H	-0.62579200	-2.40853100	-2.53080700				

IM2b

N	-1.89229500	-3.49443400	-2.01328600	C	-3.22432700	-2.60791000	1.75635400
C	-2.51265800	-2.43111100	-1.52338600	C	-3.51168700	-3.96404700	1.86425200
C	-1.89956700	-1.18736000	-1.26002500	C	-2.48454000	-4.88389700	2.06160400
C	-0.53615300	-1.09483400	-1.60324400	C	-1.16789700	-4.44720000	2.17426900
C	0.11735500	-2.19168800	-2.15494000	C	-0.87709400	-3.09081900	2.07775400
C	-0.61052400	-3.37363400	-2.32549200	Rh	0.65000700	0.58508700	0.99756700
C	-2.74250900	-0.09442700	-0.72004100	C	1.86732800	-0.97682500	0.83134900
C	-3.66811100	0.54921700	-1.56189600	C	2.41931000	1.53797900	0.70747800
N	-4.46576200	1.53720600	-1.18106800	C	3.50075500	0.73724400	0.77258600
C	-4.40968000	1.93311200	0.08305800	C	3.19304900	-0.69914100	0.84917700
C	-3.58127100	1.34168100	1.04365300	C	2.44777800	3.00049700	0.78072800
C	-2.74625300	0.31545000	0.62201500	O	3.36848800	3.74115700	0.53091600
P	-1.48538100	-0.39717700	1.74327900	O	1.22692600	3.44457200	1.19050100
P	0.39777500	0.44480300	-1.23168300	C	1.08187200	4.86227400	1.26976400
O	-3.81584500	-2.52283900	-1.24359700	H	1.14761800	-2.17263600	-2.48662600
C	-4.45294900	-3.76657400	-1.49642200	H	-3.61041200	1.69704600	2.07069900
O	-3.70845000	0.14319400	-2.83568100	H	-5.49416600	-3.62803400	-1.19537600
C	-4.66363500	0.75993200	-3.68557200	H	-4.39750700	-4.02732900	-2.55937200
O	-5.17187200	2.95349700	0.48379900	H	-3.98958700	-4.56903200	-0.90890100
C	-5.99291800	3.57638300	-0.49342100	H	-4.52660300	0.29818300	-4.66611500
O	0.04802100	-4.41522100	-2.83433200	H	-5.68268200	0.58299700	-3.32268100
C	-0.67785200	-5.62274300	-3.02137400	H	-4.49527500	1.84252600	-3.74474900
C	-0.65445000	1.80247200	-1.83949400	H	-6.50595800	4.38798600	0.02647500
C	-1.03532800	2.86566700	-1.01867600	H	-5.38609900	3.97353400	-1.31720500
C	-1.81632600	3.89611500	-1.53277400	H	-6.72216700	2.86896400	-0.90470600
C	-2.20460800	3.87443600	-2.86869600	H	0.03706900	-6.33291600	-3.44117900
C	-1.81662600	2.82013300	-3.69372100	H	-1.06885600	-5.99540700	-2.06732700
C	-1.05084200	1.78022000	-3.18267900	H	-1.51525500	-5.47331400	-3.71262200
C	-1.90120700	-2.16481200	1.85839500	H	-0.72473600	2.89322500	0.02546000

H	-2.12123700	4.71707600	-0.88543100
H	-2.80816300	4.68544400	-3.27274800
H	-2.11538700	2.80544000	-4.74052800
H	-0.75966700	0.95050300	-3.82786900
H	-4.02831400	-1.89268000	1.58070800
H	-4.54380300	-4.30420800	1.79303700
H	-2.71348800	-5.94511300	2.13976900
H	-0.36618600	-5.16347300	2.34249800
H	0.15101600	-2.74623800	2.19248700
H	0.07542200	5.04134600	1.65762200
H	1.83304800	5.28970900	1.94121300
H	1.19406100	5.31185400	0.27631000
C	-1.83095100	0.32157500	3.38365900
C	-2.56785400	-0.33546500	4.37129800
C	-1.29407100	1.58761400	3.65447100
C	-2.76813800	0.27015600	5.60792600
H	-2.98043900	-1.32503600	4.17754100
C	-1.50116800	2.19282400	4.88793300
H	-0.71901700	2.11869200	2.88733700
C	-2.23779000	1.53049500	5.86641100
H	-3.34129000	-0.24711300	6.37479700
H	-1.08165100	3.17649600	5.08907200
H	-2.39505300	1.99768100	6.83648200
C	1.92692100	0.57276200	-2.22100300
C	2.22023600	1.81523300	-2.80067100
C	2.88851600	-0.44449000	-2.28857800
C	3.42406300	2.01860800	-3.46539300
H	1.51235100	2.63909900	-2.73038900
C	4.07985900	-0.24133400	-2.97242400
H	2.74406000	-1.38996500	-1.77297100
C	4.35022700	0.98800000	-3.56556000
H	3.63361300	2.99116800	-3.90537600
H	4.80575100	-1.05092100	-3.02608500
H	5.28907700	1.14536700	-4.09309500
C	4.88934500	1.28693900	0.88089900
O	5.49075000	1.38692800	1.91951500
O	5.35289000	1.64516400	-0.32018800
C	6.61133700	2.32027200	-0.29534800
H	6.85884300	2.52715100	-1.33831100
H	6.51715000	3.25394100	0.26969500
H	7.37822000	1.69118400	0.16942700
C	4.21431800	-1.76112600	0.89463600
C	5.38631100	-1.66470500	0.13227900
C	4.00683100	-2.90773400	1.67095000
C	6.31662300	-2.69648700	0.13973700
H	5.56083900	-0.78337300	-0.48484500
C	4.93859400	-3.93737700	1.67561400
H	3.11904300	-2.97034000	2.30078100
C	6.09560500	-3.83522300	0.90869900
H	7.22263900	-2.60944700	-0.45728300
H	4.76921700	-4.81748000	2.29313500
H	6.83015800	-4.63804900	0.91907300
H	1.43323600	-1.97678600	0.75002300

IM2c

P	0.71620100	-0.43373400	1.27604800
C	2.18114800	-0.26625700	2.34215600
C	2.63078800	-1.38966900	3.04842700
C	2.95085900	0.90642200	2.36051800
C	3.82489400	-1.33798100	3.75938900
C	4.14470100	0.94623900	3.06782100
C	4.58685000	-0.17540700	3.76409300
C	-0.55113800	0.81826700	1.72726900
C	-1.88111300	0.66922800	1.28383000
C	-0.17031400	1.94946700	2.43823500
C	-2.75175500	1.73244000	1.60402900
C	-1.13974100	2.93179900	2.66627300
C	-2.21283500	1.96682700	-1.80750500
C	-3.39304400	1.90231900	-2.55623200
C	-4.24568500	2.99841600	-2.59979600
C	-2.76936500	4.21414700	-1.12643500
C	-3.93162000	4.15556200	-1.88914700
C	-0.04537500	-2.02349200	1.73475500
C	-0.63030200	-2.12433600	3.00382000
C	-0.01907900	-3.13608200	0.89149000
C	-1.17749600	-3.33152600	3.41827900
C	-0.58417000	-4.33759100	1.30674200
C	-1.16203200	-4.43569100	2.56828400
C	-2.26035000	-0.70435500	-0.83242400
C	-2.86416100	-1.78013300	-1.46860800
C	-2.43899600	-0.49166800	0.54441200
C	-3.65202400	-2.62909200	-0.68311100
C	-3.29947400	-1.39413400	1.19657200
P	-1.15149200	0.49499500	-1.65954200
C	-0.79535900	-0.10239700	-3.34132700
C	-0.29989700	-1.41060300	-3.50069900
C	-0.72734700	0.78628100	-4.42029800
C	0.21414300	-1.82556400	-4.72694400
C	-0.21189400	0.36386300	-5.63970600
C	0.25713300	-0.93873800	-5.79698200
Rh	1.05194100	-0.34430500	-0.96105000
C	2.86704300	-1.30758100	-0.75020500
C	3.90167800	-0.45189100	-0.69257300
C	2.24084400	1.26130300	-0.79297800
C	3.55427000	0.96055400	-0.73634600
H	2.05938900	-2.31592900	3.03694200
H	2.64184100	1.78491100	1.79803100
H	4.16192900	-2.21689000	4.30554400
H	4.74219400	1.85547300	3.05887200
H	5.52831700	-0.14194700	4.30852800
H	0.83426500	2.10677700	2.81535700
H	-3.64474500	0.99289700	-3.10361900
H	-5.15948700	2.95027200	-3.18871000
H	-2.52860800	5.11179600	-0.55734800
H	-4.60110600	5.01310200	-1.92772800
H	-0.65853900	-1.25973800	3.66783900
H	0.46308000	-3.08472600	-0.08442100
H	-1.62079600	-3.40883900	4.40975200
H	-0.55783000	-5.19971500	0.64295100
H	-1.59457800	-5.37910700	2.89685500
H	-2.76107500	-1.97584300	-2.53324400
H	-0.36623300	-2.12976800	-2.67896000
H	-1.07444000	1.81187100	-4.30150000
H	0.57088000	-2.84743500	-4.84643600
H	-0.17273600	1.05966100	-6.47540200
H	0.65796400	-1.26119600	-6.75556300
C	-1.91166500	3.12038600	-1.08590300
H	-1.00875400	3.15118600	-0.48082700
C	4.64232500	1.97143800	-0.66320000
O	5.73937400	1.72294700	-0.22056700
O	4.27474100	3.17067000	-1.13484200
C	1.63134300	2.60423700	-0.91508700
O	1.08887800	3.02953000	-1.90798500

O	1.72328300	3.27950100	0.24450400	N	-3.87284000	-2.43625800	0.61079800
C	5.28585600	4.17770800	-1.07762400	O	-0.73909300	4.03237100	3.31055100
H	6.18097200	3.84915300	-1.61491600	O	-4.01011100	1.61826100	1.17699800
H	4.85641600	5.06046800	-1.55445400	O	-3.52714800	-1.17797800	2.49651700
H	5.55589800	4.39164800	-0.03777400	O	-4.20610500	-3.67855100	-1.29403000
C	1.34004500	4.65350500	0.20470000	C	-1.70417600	5.05468300	3.51546000
H	1.38002800	5.00440400	1.23799900	H	-2.54702200	4.68774800	4.11183600
H	2.04150300	5.21268000	-0.42570600	H	-1.18171500	5.85117600	4.04900100
H	0.32895800	4.77246400	-0.20142200	H	-2.08795500	5.42665000	2.55663500
C	2.92197300	-2.76412300	-0.74386600	C	-4.90899900	2.67620600	1.47666200
C	2.32134900	-3.51132000	-1.76996800	H	-5.85730500	2.39194500	1.01536100
C	3.52187600	-3.44897500	0.32371800	H	-5.03194000	2.79150000	2.55983100
C	2.30251600	-4.89965200	-1.71948800	H	-4.55213700	3.62292700	1.05381600
H	1.88718400	-2.98402500	-2.62142300	C	-4.40479400	-2.07320900	3.16215400
C	3.50191600	-4.83775400	0.37016900	H	-4.01750200	-3.09954800	3.12764400
H	3.99552100	-2.87073000	1.11772900	H	-4.45614600	-1.72077700	4.19508500
C	2.88443600	-5.56674500	-0.64338100	H	-5.40134200	-2.05756400	2.70641000
H	1.84382700	-5.46641400	-2.52820100	C	-5.00837800	-4.54640000	-0.50640600
H	3.97321200	-5.35694800	1.20299800	H	-5.35326900	-5.32722200	-1.18719000
H	2.86885700	-6.65416100	-0.60216900	H	-4.42350500	-4.98547400	0.31116600
H	4.94921100	-0.76017400	-0.66352800	H	-5.86391000	-4.01117100	-0.07859300
N	-2.39712400	2.82795100	2.26568300				

IM2d

P	-0.75707600	-0.45455500	-1.23669500	C	-2.71472300	2.53129300	-0.04579800
C	-2.04297700	-0.03443200	-2.45541100	H	-2.89461800	-2.02620800	-2.43506900
C	-2.99320000	-1.00525100	-2.79698600	H	-1.53765000	2.06647500	-2.61245100
C	-2.22559100	1.27933500	-2.90822900	H	-4.82028000	-1.43320200	-3.83709200
C	-4.08974600	-0.66819400	-3.58205400	H	-3.45477100	2.63723400	-4.02472600
C	-3.32213300	1.61033900	-3.69148800	H	-5.12509200	0.90175200	-4.63038500
C	-4.25859300	0.63719000	-4.02798100	H	0.23369200	1.21800800	-3.48694000
C	0.92395900	-0.08894500	-1.88429800	H	3.83802800	0.05345900	3.04790900
C	2.05772700	-0.61735900	-1.23263300	H	6.06588900	1.13228700	2.90956400
C	1.07175000	0.81362800	-2.92726100	H	4.65676000	3.64665500	-0.28852300
C	3.30149500	-0.14739700	-1.69713400	H	6.47819500	2.92568900	1.24554800
C	2.37092300	1.21993500	-3.25667300	H	-0.08268400	-2.50399800	-3.14749500
C	2.99009500	1.26627000	1.46883600	H	-1.51861000	-2.38271600	0.92513100
C	4.01773600	0.84918700	2.32325100	H	-0.16172400	-4.97246800	-3.08776800
C	5.26725800	1.45023500	2.24224200	H	-1.52194800	-4.83940600	0.99382600
C	4.47972100	2.86301800	0.44739700	H	-0.86119500	-6.15007800	-1.01452500
C	5.49717400	2.45750000	1.30531100	H	1.53137400	-2.10216800	3.22342900
C	-0.79247500	-2.27508700	-1.11451300	H	-0.84905900	-0.93395100	2.82514200
C	-0.41023900	-3.01478600	-2.24104000	H	1.93431000	2.23977900	3.82729000
C	-1.20260900	-2.93918700	0.04301400	H	-2.35387000	-0.17571900	4.58091400
C	-0.44864900	-4.40281500	-2.20534200	H	0.47481100	2.86452500	5.72557000
C	-1.21551700	-4.33050200	0.08104400	H	-1.68741700	1.70820700	6.08492800
C	-0.84198600	-5.06178900	-1.04208500	C	3.22502600	2.26673900	0.52709300
C	1.77169500	-1.31643000	1.20242900	H	2.42740200	2.57509900	-0.14853500
C	1.74322900	-2.30497400	2.17583700	C	-3.59426800	-0.96372400	1.12498300
C	2.03447400	-1.62555400	-0.14139700	O	-3.35048700	-1.46716600	2.20717600
C	1.99336400	-3.61587200	1.75291900	O	-4.34445500	-1.55491800	0.18799600
C	2.34461100	-2.97161200	-0.40565100	C	-5.05878000	1.62453500	0.05712800
P	1.37754600	0.43502800	1.51073100	O	-5.50849900	2.44616500	-0.70995200
C	0.65165300	0.63271900	3.16712100	O	-5.80527500	0.82065900	0.82350600
C	-0.57262700	-0.03873700	3.39442800	C	-7.21320300	0.94311000	0.63258000
C	1.00980900	1.69152200	4.00869200	H	-7.48305500	0.69726800	-0.40019600
C	-1.41444800	0.35341800	4.43629100	H	-7.67254400	0.23801900	1.32777800
C	0.17824600	2.05361900	5.06263900	H	-7.54208200	1.96475500	0.84925100
C	-1.03738500	1.40025400	5.26873400	C	-4.95904100	-2.77999600	0.58167300
Rh	-0.95090000	0.49210900	0.81277800	H	-5.62270600	-2.61584100	1.43756800
C	-2.98580300	0.29327400	0.66898500	H	-5.52963100	-3.11955600	-0.28477000
C	-3.60286200	1.41856400	0.24930000	H	-4.20132000	-3.52322700	0.85613900
C	-1.39300500	2.34253100	0.08466100	H	-3.12239600	3.50712200	-0.32178400

C	-0.31642500	3.32603600	0.06984400
C	0.32706400	3.71543400	-1.11525100
C	0.05161100	3.95013100	1.27465400
C	1.28905600	4.71895500	-1.09923100
H	0.06361700	3.24091500	-2.05953900
C	1.02677400	4.93826600	1.28990900
H	-0.45691500	3.65645700	2.19474800
C	1.64179800	5.33030000	0.10207300
H	1.76085000	5.02603900	-2.03153900
H	1.29525200	5.42000100	2.22837500
H	2.39342600	6.11762000	0.11295100
N	3.45734300	0.75283300	-2.66302300
N	2.30840100	-3.93523800	0.50452000
O	2.48220400	2.14985600	-4.20845400
O	1.90480100	-4.57934500	2.67226400
O	2.66545200	-3.28294600	-1.66546100
O	4.38488400	-0.63257400	-1.08838700
C	3.79185400	2.58887900	-4.54205400
H	4.40417200	1.75685700	-4.90769100
H	3.66112400	3.33605700	-5.32731100
H	4.28803700	3.03396900	-3.67048500
C	5.65287500	-0.16640000	-1.52609100
H	5.72402100	0.92318700	-1.42749400
H	6.38299000	-0.65311200	-0.87574600
H	5.83202300	-0.44193300	-2.57192500
C	3.01008900	-4.63369400	-1.93415500
H	3.22518000	-4.67511300	-3.00443800
H	3.89317300	-4.93410900	-1.35855700
H	2.18078100	-5.30697000	-1.68326300
C	2.13294100	-5.91386900	2.24262600
H	3.15349200	-6.03592500	1.86175200
H	1.98217800	-6.53745000	3.12609600
H	1.42668800	-6.19496800	1.45114600

(*R*)-H_8-BINAP / Rh^+

P	-1.39491100	-1.07379700	0.48607300
C	-2.31062500	-2.23162900	1.55914700
C	-3.69283900	-2.42568700	1.45851800
C	-1.56660700	-3.04005200	2.43569900
C	-4.31900600	-3.39167100	2.23901100
H	-4.28309800	-1.82481800	0.76891100
C	-2.19898900	-4.00032000	3.21515800
H	-0.48558300	-2.90476000	2.50869400
C	-3.57697500	-4.17582800	3.11695500
H	-5.39481600	-3.53322100	2.15792500
H	-1.61523900	-4.61555400	3.89679200
H	-4.07312700	-4.93110300	3.72313300
C	-0.78883400	0.32195100	1.49236500
C	-0.09070100	1.40117300	0.89603500
C	-0.89590200	0.23964500	2.88169700
C	0.52266900	2.35334700	1.72553800
C	-0.31095900	1.20571000	3.68825300
H	-1.44398200	-0.58077700	3.34163800
C	0.40070300	2.25614400	3.12372200
H	-0.40821000	1.14189100	4.77195700
C	2.56755600	-0.25433100	0.60337400
C	3.52114500	0.74797400	0.39784200
C	2.53793700	-0.94343600	1.81594300
C	4.44469700	1.04133600	1.39329100
H	3.55019700	1.29741000	-0.54445500
C	3.45192300	-0.63445800	2.81837600
H	1.79272700	-1.72733100	1.96274000
C	4.41056200	0.35046600	2.60362000
H	5.19935900	1.80798000	1.22405600
H	3.42342000	-1.17290400	3.76361500
H	5.13861000	0.58035300	3.37965900
C	2.65653400	-1.41483700	-2.01215900
C	3.43836000	-0.63230700	-2.86807400
C	2.81903600	-2.80515000	-2.02185200
C	4.34728600	-1.23766500	-3.72835900
H	3.33405900	0.45228700	-2.87326300
C	3.72783900	-3.41004600	-2.88197800
H	2.24774200	-3.43323600	-1.32693900
C	4.49009900	-2.62286900	-3.73912200
H	4.94984300	-0.62324800	-4.39438000
H	3.84398200	-4.49170700	-2.87979100
H	5.20329000	-3.09008500	-4.41510400
C	-2.60889400	-0.41465300	-0.69327800
C	-3.60955700	0.45820800	-0.25117900
C	-2.60350200	-0.85706100	-2.01694700
C	-4.61641600	0.85259800	-1.12427800
H	-3.60736600	0.81590500	0.78089900
C	-3.59823400	-0.43734900	-2.89477300
H	-1.81038900	-1.52953100	-2.35042200
C	-4.60946700	0.40585100	-2.44513900
H	-5.40927800	1.51327700	-0.77645200
H	-3.58998400	-0.77918100	-3.92774700
H	-5.39879300	0.71958500	-3.12610800
C	0.64707300	0.70121100	-1.43859700
C	0.55506700	0.83593800	-2.82630900
C	-0.07617900	1.57308700	-0.59226000
C	-0.18567300	1.86654300	-3.38438800
H	1.06270700	0.12813100	-3.47950200
C	-0.83303000	2.60773300	-1.17251200
C	-0.85861000	2.76792500	-2.56907800
H	-0.23831300	1.97560600	-4.46759800
P	1.48275000	-0.77058900	-0.77117500
Rh	0.11472500	-2.45333700	-0.34849300
C	1.26149000	3.55520200	1.20052200
H	0.56633700	4.41043000	1.25644600
H	1.49682000	3.43649200	0.13502700
C	2.53518300	3.92761400	1.98783100
H	3.41355200	3.50062600	1.48660100
H	2.65654000	5.01776400	1.93502500
C	2.51552500	3.47114300	3.44959500
H	3.02558000	2.49965600	3.54172600
H	3.06610100	4.17876700	4.08206000
C	1.08580200	3.29583300	3.95739300
H	0.54724100	4.25646100	3.88326300
H	1.06940100	3.01001200	5.01728400
C	-1.67082800	3.89657000	-3.12674000
H	-1.17812300	4.85855900	-2.90431600
H	-1.73155300	3.82333600	-4.22050900
C	-1.60614500	3.63012400	-0.38277100
H	-1.00328700	4.55477400	-0.37303100
H	-1.70784100	3.33109100	0.66802000
C	-3.06200000	3.87615200	-2.49481400
H	-3.54477200	2.93060000	-2.78445500
H	-3.67996500	4.68361600	-2.90704200
C	-2.99100700	3.97165300	-0.96516300
H	-3.74310600	3.30386600	-0.52421100
H	-3.25393300	4.98423900	-0.63210900

IM1a’

C	-2.15310600	1.99699800	1.69185500	C	6.08621100	-2.98912700	-0.52855000
C	-2.85654500	2.12920100	0.68805600	C	5.88084400	-2.30166000	0.81225500
C	-3.78079200	2.23559200	-0.38929000	C	2.38862900	2.89235200	-4.10836600
C	-5.10962700	1.83020100	-0.17893900	C	3.94633800	1.87684800	-1.80400000
C	-3.39076100	2.71930200	-1.64695000	C	3.57277000	3.64189700	-3.52006900
C	-6.03038400	1.91474300	-1.21414300	C	4.57290400	2.64722700	-2.95665000
C	-4.31721600	2.79851500	-2.67425100	H	-1.74045100	2.25488300	2.65244000
C	-5.63517600	2.39810300	-2.45976300	H	-5.39678300	1.44591500	0.79907000
C	-2.91230400	-0.92934300	1.20503000	H	-2.35661200	3.01882500	-1.81254400
C	-3.14894700	-0.73054900	-0.00658600	H	-7.06000800	1.60370400	-1.04810800
C	-3.81332500	-0.85068400	-1.30042300	H	-4.01326800	3.16953200	-3.65050600
O	-3.35551500	-0.45009600	-2.34586100	H	-6.35937400	2.46586800	-3.26966400
O	-4.98961900	-1.46677900	-1.16548600	H	-6.61161500	-2.20057800	-2.11855300
C	-5.70466700	-1.65705500	-2.38774900	H	-5.95303200	-0.68703100	-2.83516100
C	-2.90798400	-1.33553600	2.60513900	H	-5.09916800	-2.23668900	-3.09365900
O	-2.13706400	-2.14571900	3.06869700	H	-4.70082500	-0.35957400	5.08878400
O	-3.83380200	-0.66789900	3.28999700	H	-4.05889800	-2.02307900	4.85619900
C	-3.87915700	-0.95624400	4.69128200	H	-2.93217900	-0.67064700	5.16225700
P	0.82912600	1.17212900	1.30703300	H	1.14100100	3.52018300	3.17421800
C	0.63684000	1.41815700	3.11703500	H	-0.00826000	-0.63013500	3.39772100
C	0.80972600	2.65419900	3.74473900	H	0.67723400	3.76653000	5.57999100
C	0.17572700	0.33252700	3.87538300	H	-0.40919600	-0.38847100	5.80999700
C	0.54272700	2.79616300	5.10590600	H	-0.09397100	1.82237400	6.91675300
C	-0.07208800	0.47256900	5.23430900	H	2.80769900	0.17463500	3.23928800
C	0.10850600	1.70837600	5.85359300	H	4.80006900	-1.24919300	3.00630100
C	2.38308900	0.20923100	1.11842800	H	1.66513400	-3.44507600	-1.71595900
C	2.80685500	-0.22757900	-0.15271800	H	0.41043700	-2.23821100	2.21719300
C	3.11437100	-0.16029200	2.24985700	H	3.04341100	-5.24489000	-0.72697200
C	3.94062300	-1.05260800	-0.27744200	H	1.79824200	-4.03811800	3.20919000
C	4.23371500	-0.96341400	2.11881400	H	3.12521000	-5.53604700	1.74033700
C	4.65123300	-1.42998500	0.87308100	H	-0.31206700	-2.09267400	-3.54352100
C	1.00859800	-2.67963700	0.19740800	H	-2.03140300	-3.37829100	0.19637800
C	1.72309300	-3.55271400	-0.63141800	H	-1.73904200	-3.69963000	-4.74346300
C	1.02499300	-2.87001000	1.57751300	H	-3.47040000	-4.97119500	-1.01943900
C	2.48970000	-4.56837500	-0.07724000	H	-3.34657700	-5.12496100	-3.49853000
C	1.79091500	-3.89323900	2.13064500	H	3.10567700	2.99549400	1.72716300
C	2.53116500	-4.73338300	1.30684100	H	-0.48981100	2.88874000	-0.62370400
C	-1.10939700	-2.58595000	-1.59462600	H	3.63763900	5.23682600	0.81608100
C	-1.01412000	-2.70629500	-2.98230100	H	0.07203400	5.11348600	-1.58765900
C	-1.98540500	-3.42463200	-0.89269400	H	2.12712800	6.29596900	-0.84520400
C	-1.81935800	-3.61569600	-3.66133900	H	-0.97944000	-0.05668800	-2.89983800
C	-2.78995300	-4.32877500	-1.57499700	H	-0.00758400	1.73388000	-4.27989600
C	-2.71709400	-4.41633900	-2.96317400	H	3.73690200	-2.42350700	-1.89828100
C	1.25223600	2.81430600	0.62673800	H	4.18400300	-0.81196200	-2.40994600
C	2.42577400	3.47159100	1.01815300	H	6.06538800	-2.45054400	-2.63393800
C	0.41191700	3.41449300	-0.31095900	H	6.48896800	-1.12829700	-1.54276600
C	2.73075800	4.72489800	0.49955400	H	5.38497800	-3.83380800	-0.62376300
C	0.72963200	4.65759400	-0.84886500	H	7.09803600	-3.41062300	-0.58347100
C	1.88306000	5.31735000	-0.43533700	H	5.83488900	-3.04004700	1.62680300
C	0.80189700	-0.30953600	-1.68647600	H	6.75804100	-1.67037400	1.03469300
C	0.04431100	0.26980700	-2.71697600	H	1.56741100	3.58189900	-4.35569100
C	2.06499400	0.22592300	-1.36980000	H	2.69066700	2.43945700	-5.06854500
C	0.58155300	1.29545000	-3.47294800	H	3.80167600	2.56254100	-0.95087000
C	2.60471000	1.27134100	-2.15061500	H	4.64024300	1.10888700	-1.43937100
C	1.86008600	1.79440000	-3.22017900	H	4.02937700	4.28353200	-4.28461700
P	-0.09923400	-1.44872800	-0.57391600	H	3.22888600	4.30371500	-2.70806800
Rh	-1.26095300	0.21318900	0.55579500	H	5.48421600	3.15191800	-2.61037200
C	4.37467500	-1.56246700	-1.63150500	H	4.88234500	1.94574300	-3.74997200
C	5.82981900	-2.00569100	-1.65845800				

IM1b'

P	0.57305600	-1.64323000	-0.70707600
C	-0.18564700	-2.64142400	-2.04069000
C	-0.09817800	-4.03362800	-2.09746400
C	-1.00647500	-1.97193700	-2.96091400
C	-0.79867600	-4.74334400	-3.07014200
C	-1.68987600	-2.68292600	-3.93853800
C	-1.58916100	-4.07177800	-3.99426700
C	1.86016300	-0.61983600	-1.52211000
C	2.68544900	0.22434400	-0.75720400
C	1.92023100	-0.55536700	-2.91764500
C	3.53405100	1.15703000	-1.39217700
C	2.75516400	0.35924700	-3.53423100
C	3.54728700	1.23583300	-2.79344100
C	0.60608500	2.57427300	-0.36947100
C	1.32270400	3.70310100	0.04833500
C	1.67271000	4.68726700	-0.86893200
C	0.64242000	3.40964700	-2.63714400
C	1.33560900	4.53939900	-2.21347100
C	1.46179500	-2.80815900	0.38172100
C	2.46531800	-3.64540900	-0.12119300
C	1.15684600	-2.84489800	1.74503400
C	3.13535000	-4.52011500	0.72658400
C	1.84178300	-3.70671200	2.59553400
C	2.82552200	-4.54849000	2.08521400
C	1.74518600	0.69121700	1.55296300
C	1.92501800	0.67990500	2.94032200
C	2.73772300	0.11562500	0.73343800
C	3.02271600	0.05092300	3.49927300
C	3.85336800	-0.53033500	1.31105900
C	3.97619700	-0.58674000	2.70806000
P	0.15220100	1.28101000	0.83863700
C	4.41214900	2.08081600	-0.57758600
C	5.54930300	2.69002600	-1.38392500
C	5.00562600	3.30804200	-2.66048300
C	4.41503500	2.21825000	-3.54013700
C	5.12301300	-1.28775000	3.39185400
C	4.90557600	-1.17645100	0.43630400
C	5.90839100	-2.20783100	2.47204700
C	6.20260400	-1.47927900	1.17195800
C	-0.74052600	2.14973100	2.18185600
C	-1.13176700	1.41631600	3.31194400
C	-1.19917400	3.46214200	2.04013600
C	-1.89976700	2.00708900	4.30712400
C	-1.99387800	4.04102100	3.02580000
C	-2.33393500	3.32275200	4.16587600
Rh	-1.23494300	-0.45692100	0.22812500
C	-2.10596400	-2.57746500	0.43855300
C	-3.16700300	-2.21668800	-0.07353200
C	-2.71931800	1.01228200	-0.25151900
C	-3.07297500	0.62931600	0.88445200
H	0.50646200	-4.57927700	-1.37501700
H	-1.13558700	-0.88874400	-2.90028400
H	-0.72350100	-5.82862700	-3.10087400
H	-2.31368000	-2.14874800	-4.65392200
H	-2.13361600	-4.62865500	-4.75435100
H	1.30905900	-1.21647000	-3.52768200
H	2.79297300	0.40507200	-4.62360500
H	1.60742400	3.81130000	1.09649000
H	2.22061000	5.56734900	-0.53623900
H	0.39788000	3.27647300	-3.69046300
H	1.62194100	5.30592600	-2.93191200
H	2.72717000	-3.60511000	-1.18027400
H	0.39606100	-2.17007600	2.14392800
H	3.90955000	-5.17430900	0.32916800
H	1.60778500	-3.71921000	3.65849200
H	3.35703300	-5.22927800	2.74806000
H	1.20979400	1.16946700	3.59486500
H	3.14514000	0.04557800	4.58331500
H	3.78216100	2.89067500	-0.17172300
H	4.81107400	1.56183100	0.30348100
H	6.07816200	3.43363900	-0.77373700
H	6.28576400	1.90820000	-1.63765500
H	4.22428500	4.04020000	-2.39827700
H	5.78404400	3.85409900	-3.20858800
H	3.83067700	2.65283400	-4.36557400
H	5.23400100	1.65784500	-4.02245200
H	4.73612100	-1.83566100	4.26428000
H	5.80254500	-0.52183100	3.80322500
H	4.49104400	-2.11639200	0.03470900
H	5.11076500	-0.55687100	-0.44630300
H	6.83066600	-2.53804800	2.96688000
H	5.31770600	-3.11416800	2.25455400
H	6.86437600	-2.06775900	0.52330500
H	6.73400400	-0.53767100	1.39274000
H	-0.85495400	0.36539000	3.40469000
H	-0.95067200	4.03889000	1.15138400
H	-2.17024500	1.43128400	5.19117500
H	-2.34956600	5.06112100	2.89677600
H	-2.94900000	3.78150300	4.93731300
C	0.26753500	2.43607000	-1.71594600
H	-0.26516900	1.54732800	-2.05327200
C	-3.96539100	0.59093300	2.04005500
O	-4.86916600	1.37479600	2.19540400
O	-3.67054200	-0.41251600	2.87576100
C	-2.80819300	1.69303700	-1.54246900
O	-2.68635300	1.14054900	-2.61393100
O	-3.03580500	2.99150900	-1.37036600
C	-4.52787100	-0.49520200	4.01372400
H	-5.56900400	-0.63664600	3.70161000
H	-4.17853500	-1.35308000	4.59051200
H	-4.46425100	0.42483500	4.60632600
C	-3.14916000	3.75737900	-2.57330200
H	-3.38413700	4.77328600	-2.25399900
H	-2.20066700	3.73527200	-3.12220400
H	-3.94551100	3.35630300	-3.20778400
C	-4.42978800	-1.77609200	-0.55117700
C	-4.59489900	-1.33835200	-1.87377800
C	-5.51911100	-1.74968700	0.33545500
C	-5.83089400	-0.86834300	-2.29175600
H	-3.74154300	-1.34517900	-2.54934000
C	-6.75213900	-1.28307200	-0.09490300
H	-5.37670800	-2.10265500	1.35578400
C	-6.90696100	-0.83895200	-1.40620300
H	-5.95673000	-0.51966200	-3.31477800
H	-7.59440900	-1.26019500	0.59318400
H	-7.87347500	-0.46673400	-1.74041600
H	-1.41402200	-3.31513600	0.80693800

IM1c'

P	0.21428800	1.43967100	0.48292900
C	-0.57496600	2.36876500	1.85144700
C	-0.35839700	3.72150400	2.12642200
C	-1.42084900	1.63279300	2.69198200
C	-0.95702800	4.31489000	3.23348500
C	-2.01328100	2.22750300	3.79988200
C	-1.77567800	3.57009500	4.07723500
C	1.68103200	0.66535700	1.27082000
C	2.53444900	-0.16574900	0.52375800
C	1.87598800	0.78830800	2.64994300
C	3.56519500	-0.88780900	1.16429800
C	2.88503400	0.07559500	3.27236000
C	3.72417100	-0.77582300	2.55433900
C	0.70546200	-2.65869800	0.84735300
C	1.56929200	-3.72850200	0.58081500
C	2.11590000	-4.46108900	1.62763600
C	0.97021900	-3.05179400	3.21589000
C	1.82104900	-4.11914900	2.94631200
C	0.90683000	2.64532500	-0.70375100
C	1.83317100	3.61333900	-0.29597800
C	0.52819200	2.58552700	-2.04731000
C	2.32379300	4.54304400	-1.20695500
C	1.04754200	3.49284000	-2.96572700
C	1.92921200	4.48413300	-2.54197700
C	1.40146800	-1.10168400	-1.54142600
C	1.39708800	-1.28904900	-2.92752700
C	2.39561900	-0.28505900	-0.96126700
C	2.31744300	-0.63166900	-3.72380900
C	3.32326800	0.39061000	-1.78212000
C	3.26176400	0.23470500	-3.17684300
P	-0.01174000	-1.72248900	-0.54504700
C	4.47937600	-1.79737500	0.37407800
C	5.76971700	-2.13032100	1.10836800
C	5.45552900	-2.63292000	2.50701400
C	4.79435300	-1.52432700	3.31002200
C	4.20377900	0.95169600	-4.11068200
C	4.36298300	1.30401600	-1.17177400
C	4.94455800	2.10570000	-3.45616700
C	5.50224800	1.64602300	-2.12009000
C	-0.90900000	-2.96158100	-1.55314300
C	-1.54596400	-2.54162500	-2.73054000
C	-1.14045000	-4.25972500	-1.08950800
C	-2.34896400	-3.41503000	-3.45231800
C	-1.95706200	-5.12833000	-1.80817500
C	-2.55427500	-4.71360300	-2.99259900
Rh	-1.50197100	-0.01792200	-0.26158300
C	-2.77276800	2.49252500	-0.88485900
C	-2.75962800	1.45065800	-1.54036500
C	-2.84649500	-1.38967200	0.60856100
C	-3.32342900	-1.18677200	-0.53730300
H	0.25909800	4.33108700	1.47047200
H	-1.62566800	0.58095500	2.48890400
H	-0.78404000	5.37145300	3.43207700
H	-2.66086200	1.63069500	4.44004200
H	-2.23541000	4.04019700	4.94495100
H	1.23031400	1.43302400	3.24333500
H	3.02784700	0.17209900	4.34976300
H	1.81263900	-3.98934000	-0.45093100
H	2.78124400	-5.29645500	1.41442000

H	0.74683200	-2.77167500	4.24444300
H	2.26082000	-4.68656900	3.76501500
H	2.18828000	3.62820100	0.73560700
H	-0.17312000	1.81706200	-2.37433800
H	3.03099100	5.30258000	-0.87858700
H	0.75674300	3.42967200	-4.01284000
H	2.32160400	5.20571300	-3.25663900
H	0.67788000	-1.95679000	-3.39331800
H	2.30070300	-0.78385200	-4.80378900
H	3.93705200	-2.73407700	0.15580000
H	4.70602300	-1.36402600	-0.60865200
H	6.34009600	-2.87332500	0.53591900
H	6.40246100	-1.22870700	1.17440700
H	4.77483400	-3.49673300	2.43260600
H	6.35898400	-2.98361500	3.02207600
H	4.36163800	-1.91910700	4.24217800
H	5.56311000	-0.79905000	3.62681300
H	3.64126000	1.29142200	-4.99312100
H	4.93734500	0.22098500	-4.49236700
H	3.86019800	2.23700900	-0.86578600
H	4.75927300	0.87468300	-0.24217800
H	5.73786300	2.47019900	-4.12120300
H	4.25189000	2.94829600	-3.28880000
H	6.14023600	2.41528700	-1.66601800
H	6.13973700	0.75873300	-2.27501100
H	-1.44746700	-1.51034200	-3.07127500
H	-0.69770900	-4.59895700	-0.15543500
H	-2.83404400	-3.06700000	-4.36267400
H	-2.12801600	-6.13557800	-1.43339300
H	-3.18978200	-5.39739900	-3.55253500
C	0.40436100	-2.32946000	2.16889400
H	-0.25654600	-1.49079200	2.38636600
C	-4.27462100	-1.42916300	-1.61755600
O	-4.26913300	-0.86563600	-2.69280000
O	-5.14271200	-2.37744900	-1.27113900
C	-2.79618500	-2.01007100	1.93603400
O	-2.74694300	-1.39706400	2.97813400
O	-2.79981400	-3.33700300	1.82660600
C	-6.09889200	-2.72009000	-2.27791900
H	-5.58794900	-3.10740100	-3.16654000
H	-6.73298800	-3.48781600	-1.83284700
H	-6.69072200	-1.84303800	-2.55877600
C	-2.73367200	-4.05014800	3.06626600
H	-2.81244200	-5.10559700	2.80274900
H	-1.77799200	-3.84730300	3.56371700
H	-3.55642800	-3.75155100	3.72290700
C	-2.79115500	3.73422500	-0.19796000
C	-1.99844500	4.80055500	-0.65722500
C	-3.66221000	3.92812600	0.88572300
C	-2.09013300	6.04168100	-0.04528700
H	-1.32388900	4.64031700	-1.49829600
C	-3.74006500	5.17142300	1.49424800
H	-4.26630000	3.09328700	1.23743300
C	-2.96164100	6.22853500	1.02613900
H	-1.48534200	6.87002900	-0.40905900
H	-4.41489900	5.32075400	2.33479000
H	-3.03611300	7.20615600	1.49932000
H	-3.11896300	0.80292400	-2.33208600

IM1d’

P	-0.03384200	1.29175800	1.01566000
C	-1.08511700	1.96889200	2.35637900
C	-1.20274800	3.33895800	2.60381500
C	-1.90921800	1.08195000	3.06673700
C	-2.08652400	3.80663700	3.57297300
C	-2.76730500	1.54964200	4.05351600
C	-2.85571900	2.91579300	4.31111300
C	1.15842600	0.14417700	1.82329900
C	2.20534300	-0.44428000	1.08628500
C	0.94377600	-0.26951800	3.14281600
C	2.98617500	-1.47564600	1.65531800
C	1.70252500	-1.28799400	3.68871500
C	2.70258900	-1.92327900	2.95440500
C	0.52961300	-2.72996400	-0.32420300
C	1.48421500	-3.65015600	-0.77399500
C	1.71348700	-4.82216500	-0.06276900
C	0.06769700	-4.15598700	1.57185700
C	1.00868500	-5.07267800	1.11350700
C	0.95862000	2.69172500	0.39057500
C	1.72507700	3.46055900	1.27545900
C	0.99550800	2.97978200	-0.97523700
C	2.49192200	4.51845800	0.80023500
C	1.78573700	4.02066500	-1.45179900
C	2.52424100	4.79780800	-0.56460700
C	1.84524500	-0.35839000	-1.41976000
C	2.30340800	0.02315800	-2.68472100
C	2.57245300	0.03837200	-0.28086300
C	3.43380100	0.81088900	-2.80957700
C	3.72161200	0.84859700	-0.41425400
C	4.14078300	1.25093200	-1.69162000
P	0.21066000	-1.19071600	-1.25037200
C	4.11853600	-2.10823800	0.87637000
C	5.09967800	-2.86863300	1.75628900
C	4.34866400	-3.83324200	2.65790600
C	3.46448800	-3.04663400	3.61195600
C	5.36154900	2.10948700	-1.91170900
C	4.49555400	1.29960700	0.80478500
C	5.89761000	2.75711200	-0.64534600
C	5.91596000	1.73742900	0.48029800
C	-0.25718400	-1.70543400	-2.94569800
C	-0.59988500	-0.70320700	-3.86592800
C	-0.41180700	-3.04282700	-3.31600000
C	-1.05483800	-1.03534100	-5.13470900
C	-0.88045300	-3.37260000	-4.58655100
C	-1.19801300	-2.37315300	-5.49778700
Rh	-1.49423900	0.27872200	-0.47178600
C	-2.39773800	2.18325800	-0.84827400
C	-3.09411300	1.74211600	0.09023900
C	-3.19169400	-1.31129500	-1.28947800
C	-3.06973000	-0.36937000	-2.06721500
H	-0.61699600	4.05354000	2.02854100
H	-1.88451200	0.01467100	2.83937200
H	-2.17341000	4.87704000	3.74670900
H	-3.37753400	0.84440000	4.61631000
H	-3.53801200	3.28526200	5.07390400
H	0.18317600	0.20593300	3.75541300
H	1.51719900	-1.60632900	4.71566500
H	2.05348700	-3.44402800	-1.68243800
H	2.45302500	-5.53733600	-0.41989000
H	-0.48539900	-4.34718500	2.49008800
H	1.19725300	-5.98727400	1.67366900
H	1.72387200	3.22720400	2.34166300
H	0.43622300	2.35879900	-1.67644900
H	3.07636400	5.11878700	1.49535200
H	1.83362600	4.21504200	-2.52274400
H	3.13551500	5.61870400	-0.93612700
H	1.77930900	-0.29715500	-3.58165100
H	3.78187500	1.09898000	-3.80265400
H	3.68879700	-2.80118300	0.13395400
H	4.65224000	-1.35324900	0.28526700
H	5.82832000	-3.39502800	1.12624900
H	5.67317600	-2.15699800	2.37495800
H	3.72888600	-4.49994700	2.03507400
H	5.03588600	-4.47443400	3.22459800
H	2.74729200	-3.70929600	4.12017700
H	4.08819100	-2.61650500	4.41403600
H	5.13211100	2.86847800	-2.67517300
H	6.14872700	1.47636800	-2.35558800
H	3.95442500	2.14365000	1.26618000
H	4.50809100	0.51515500	1.57206300
H	6.89587800	3.17438200	-0.82943000
H	5.24840500	3.59787000	-0.35254600
H	6.39195000	2.14659900	1.38088300
H	6.51490600	0.86159700	0.17631500
H	-0.53752000	0.34916900	-3.58120500
H	-0.17948300	-3.83976800	-2.61142700
H	-1.31053500	-0.24537100	-5.83842100
H	-0.99428300	-4.41975500	-4.86161700
H	-1.56321700	-2.63368900	-6.48928200
C	-0.17718900	-2.99270800	0.84987900
H	-0.91975600	-2.27629600	1.20430000
C	-2.02388200	3.00599500	-1.99666600
O	-1.80994900	2.55772100	-3.10312500
O	-1.95607600	4.28751200	-1.65990900
C	-4.21784000	1.81226900	1.02208300
O	-4.69478000	2.85109200	1.40361000
O	-4.66141500	0.59578400	1.35991400
C	-5.80000400	0.59360200	2.22321500
H	-6.63979800	1.11177900	1.74962400
H	-6.04371100	-0.45798600	2.38956000
H	-5.56019900	1.09416400	3.16831500
C	-1.60070000	5.18166700	-2.71946200
H	-2.32565300	5.11199300	-3.53624900
H	-1.60942700	6.17853000	-2.27785400
H	-0.60263600	4.93508400	-3.09881200
H	-3.16425900	0.37003400	-2.84169300
C	-3.32081900	-2.50672700	-0.51771500
C	-3.65194600	-2.46327300	0.84517700
C	-3.11301100	-3.74496800	-1.14533600
C	-3.76445600	-3.64459000	1.56515500
H	-3.81756300	-1.49482200	1.31394200
C	-3.23084200	-4.91970800	-0.41570900
H	-2.86420500	-3.76456600	-2.20570100
C	-3.55432800	-4.87161500	0.93822300
H	-4.02647700	-3.61181800	2.62135400
H	-3.07317200	-5.87847700	-0.90533700
H	-3.64943600	-5.79520200	1.50634000

TS1a'

C	-1.97311300	1.90564900	1.57516300
C	-3.03154700	1.68415200	0.88082400
C	-4.14686000	2.16832800	0.10236700
C	-5.46386500	1.93195400	0.51827500
C	-3.90182800	2.84490200	-1.09948600
C	-6.52275700	2.38522100	-0.25599900
C	-4.96487800	3.28504200	-1.87349400
C	-6.27319600	3.05390700	-1.45324800
C	-2.42871400	-0.98086900	1.42997000
C	-3.16805000	-0.31340200	0.59520400
C	-4.13589700	-0.63423600	-0.47116400
O	-3.97563800	-0.30739700	-1.62341900
O	-5.17428500	-1.31822900	0.00332000
C	-6.16365000	-1.64780600	-0.97625500
C	-2.30908600	-2.15872500	2.29022600
O	-2.87172000	-3.19982000	2.03983700
O	-1.49347300	-1.95095400	3.32081800
C	-1.30808500	-3.08194000	4.17696900
P	1.07515400	0.93413100	1.49319800
C	1.11206800	0.81163100	3.31983200
C	0.90635100	1.94901500	4.10739800
C	1.21496300	-0.43434800	3.95133400
C	0.82580000	1.84622100	5.49269900
C	1.15714900	-0.53030800	5.33614900
C	0.95861400	0.60883200	6.11133500
C	2.57168100	0.06707400	0.85965500
C	2.74461600	-0.11189100	-0.53275300
C	3.53157100	-0.43476000	1.74005300
C	3.85295900	-0.83105700	-1.00379500
C	4.64033700	-1.12006200	1.26171300
C	4.81039900	-1.32260200	-0.10181200
C	0.94333200	-2.66346900	-0.40518100
C	1.52655800	-3.46522000	-1.39060800
C	1.11252000	-2.98881700	0.94077300
C	2.29723500	-4.56138300	-1.02507100
C	1.89197600	-4.08227300	1.30560700
C	2.48646800	-4.86615500	0.32258000
C	-1.37192900	-2.21398200	-1.94176800
C	-1.36052600	-2.12112200	-3.33589800
C	-2.28145900	-3.07608700	-1.31718900
C	-2.27405100	-2.84954000	-4.09053900
C	-3.18588300	-3.80804200	-2.07723600
C	-3.19259100	-3.68566200	-3.46395300
C	1.45836700	2.69544300	1.14794700
C	2.62495800	3.27045600	1.66919700
C	0.62548400	3.46266500	0.33203200
C	2.93183300	4.59924300	1.40294900
C	0.93983900	4.79060000	0.05536600
C	2.08700900	5.36136200	0.59728800
C	0.54075400	0.04833400	-1.78208500
C	-0.34616100	0.79945400	-2.57494300
C	1.82498300	0.55941900	-1.50430200
C	0.08050100	1.97904200	-3.15786600
C	2.24832300	1.76327900	-2.10729500
C	1.37310000	2.46387400	-2.95520400
P	-0.21218900	-1.32711900	-0.84683400
Rh	-1.07664400	0.27522300	0.66105900
C	4.13750400	-1.04764300	-2.46599000
C	4.66551800	-2.45595200	-2.79859400
C	5.52086600	-3.08249400	-1.68654900
C	5.99415700	-2.04372600	-0.67164200
C	1.77860100	3.73533300	-3.65813000
C	3.60773100	2.34576300	-1.79382000
C	3.01900500	4.39724300	-3.07854500
C	4.08853100	3.34695900	-2.83374600
H	-1.62415300	2.71934100	2.19995900
H	-5.64109800	1.39012600	1.44659700
H	-2.87066100	2.99083200	-1.42297300
H	-7.54647800	2.21665900	0.07216800
H	-4.77759200	3.80399700	-2.81118800
H	-7.10576300	3.39844500	-2.06370900
H	-6.93662400	-2.20162600	-0.44176400
H	-6.57358500	-0.73112300	-1.41610500
H	-5.72194800	-2.26078400	-1.76996200
H	-0.69415200	-2.72422700	5.00521300
H	-2.27123100	-3.45093400	4.54237600
H	-0.80105500	-3.88671900	3.63062800
H	0.81810300	2.92950400	3.64143100
H	1.36180700	-1.33920000	3.36047400
H	0.66895900	2.74262500	6.08949600
H	1.27671100	-1.50130500	5.81559100
H	0.91020200	0.53071400	7.19557300
H	3.42401000	-0.28371600	2.81242100
H	5.39021100	-1.49378500	1.95923600
H	1.36060900	-3.23939000	-2.44573700
H	0.61333800	-2.38607200	1.70235500
H	2.74657600	-5.18872300	-1.79404900
H	2.02570100	-4.32757100	2.35853000
H	3.08774200	-5.72895400	0.60350100
H	-0.64004400	-1.47825500	-3.84022800
H	-2.29275800	-3.19055600	-0.23200800
H	-2.26242300	-2.76672300	-5.17569800
H	-3.88584900	-4.47700200	-1.57897900
H	-3.90437900	-4.25470900	-4.05921400
H	3.29361900	2.67440200	2.29191300
H	-0.27515300	3.01800500	-0.09481600
H	3.83397900	5.04138600	1.82131900
H	0.28498200	5.37985700	-0.58511500
H	2.32820500	6.40231900	0.38871800
H	-1.37450200	0.46527100	-2.71929100
H	-0.60607600	2.54671600	-3.78778800
H	3.25409000	-0.82273700	-3.07793100
H	4.90273500	-0.31261900	-2.77392000
H	3.81589200	-3.11657600	-3.01712000
H	5.24006300	-2.38940300	-3.73169000
H	4.92721300	-3.82887200	-1.13890900
H	6.37942200	-3.61685300	-2.11219600
H	6.58257200	-2.51328700	0.12772900
H	6.66424500	-1.32037000	-1.16758100
H	0.92581700	4.43106900	-3.66358900
H	1.96364400	3.49136400	-4.71851300
H	3.54705700	2.84842600	-0.81180200
H	4.34989900	1.55019700	-1.66092500
H	3.37508400	5.18301600	-3.75710300
H	2.77159200	4.88686900	-2.12206500
H	5.02592700	3.80711400	-2.49536700
H	4.31575300	2.82391800	-3.77791200

TS1b'

P	0.34731100	-1.65817300	-0.57637900
C	-0.64540100	-2.57284700	-1.81207800
C	-0.87299500	-3.94778600	-1.72359400
C	-1.35383400	-1.82622100	-2.76719200
C	-1.77526700	-4.56734400	-2.58536400
C	-2.24258100	-2.45003500	-3.63161500
C	-2.45751500	-3.82356800	-3.54000600
C	1.75504200	-0.89861400	-1.46954700
C	2.73567000	-0.16934700	-0.76963800
C	1.78260800	-0.92242600	-2.86705500
C	3.71454800	0.56170200	-1.47483500
C	2.74348800	-0.20021200	-3.55196800
C	3.69777500	0.56204100	-2.87882400
C	1.09659100	2.51763400	-0.57081600
C	2.04933100	3.49172100	-0.24566500
C	2.52188000	4.35954000	-1.22403900
C	1.13944400	3.26346600	-2.87112500
C	2.06805900	4.24518400	-2.53721200
C	1.06634100	-2.90320900	0.54673000
C	1.88498400	-3.91652700	0.03132600
C	0.82543800	-2.84412300	1.92081700
C	2.43189800	-4.87130500	0.88086700
C	1.39363100	-3.78701100	2.77148800
C	2.18753200	-4.80501300	2.25157700
C	1.87176000	0.57370100	1.48754200
C	1.97440500	0.56787100	2.88201000
C	2.77901900	-0.18811300	0.72493000
C	2.94303800	-0.20067100	3.50309700
C	3.76010600	-0.97431200	1.36567900
C	3.82804200	-0.98835800	2.76826000
P	0.42979800	1.39730200	0.70479800
C	4.75916100	1.36511800	-0.73264000
C	5.95551000	1.73674100	-1.59618400
C	5.48270100	2.36526700	-2.89615600
C	4.69868900	1.33858500	-3.69753500
C	4.84394600	-1.81288600	3.51834500
C	4.70912600	-1.83077300	0.55690200
C	5.51669700	-2.87699100	2.66687900
C	5.93767000	-2.26978200	1.33952900
C	-0.32427000	2.43174700	2.01520700
C	-1.12476700	1.78055200	2.96496200
C	-0.20714300	3.82199100	2.06965800
C	-1.77626800	2.50389600	3.95574200
C	-0.86395400	4.54441400	3.06066800
C	-1.64611700	3.88939700	4.00508000
Rh	-1.22808100	-0.13914100	0.18460300
C	-2.22763700	-1.83511700	0.88761300
C	-3.22791900	-1.28220900	0.31563900
C	-2.45902100	1.44374700	-0.38765100
C	-3.26001400	0.76995700	0.35610800
H	-0.35769500	-4.54516200	-0.97324300
H	-1.22595600	-0.74285300	-2.82110600
H	-1.94483700	-5.63922200	-2.50382800
H	-2.78278000	-1.85827100	-4.36854000
H	-3.16400600	-4.31058100	-4.20907600
H	1.05056600	-1.50233800	-3.42468400
H	2.75523900	-0.21833800	-4.64260900
H	2.43240600	3.56322000	0.77394000
H	3.25682500	5.11980900	-0.96399500
H	0.78625800	3.16383100	-3.89582000
H	2.44540000	4.92205100	-3.30204300
H	2.09562500	-3.95562200	-1.03918400
H	0.21050900	-2.03867700	2.32564000
H	3.05824400	-5.66345400	0.47455000
H	1.21426600	-3.72571200	3.84346800
H	2.62202700	-5.54993600	2.91615200
H	1.29359100	1.16190500	3.48781900
H	3.01510100	-0.19960800	4.59161000
H	4.28575000	2.28884300	-0.35876700
H	5.09284300	0.83187800	0.16702000
H	6.61512300	2.41564400	-1.04040800
H	6.54852300	0.83338300	-1.81955500
H	4.83969100	3.23179600	-2.66680300
H	6.32478500	2.74232100	-3.49044800
H	4.17621600	1.81284500	-4.54206100
H	5.40236500	0.62069300	-4.15250000
H	4.35988800	-2.26027100	4.39967200
H	5.61312900	-1.13093400	3.91947400
H	4.16008500	-2.72610600	0.21783500
H	5.01101900	-1.31593000	-0.36427200
H	6.37257400	-3.30426700	3.20474200
H	4.81144900	-3.70333200	2.47659900
H	6.52099400	-2.98178800	0.74147500
H	6.59274600	-1.40104500	1.52357100
H	-1.25165900	0.69622900	2.91724200
H	0.37489700	4.35639100	1.32164800
H	-2.39303200	1.98298700	4.68596900
H	-0.76692100	5.62819400	3.08905100
H	-2.16117600	4.45894300	4.77623100
C	0.65228900	2.40620800	-1.88948300
H	-0.07352900	1.63689100	-2.15287700
C	-4.44897100	1.06511000	1.17785500
O	-5.25183300	1.90276000	0.85190000
O	-4.50145600	0.33119400	2.28999200
C	-2.36715700	2.68675300	-1.15472700
O	-2.54098300	2.71317400	-2.35014900
O	-2.06003100	3.72710300	-0.38514200
C	-5.65590000	0.56752700	3.09938300
H	-6.56562700	0.34277200	2.53185600
H	-5.56084600	-0.10217500	3.95551700
H	-5.68766900	1.61214900	3.42599300
C	-1.86583200	4.96635300	-1.07294900
H	-1.71536700	5.71380400	-0.29153000
H	-0.98145000	4.89865900	-1.71966200
H	-2.74350200	5.21183600	-1.67801000
C	-4.51190600	-1.50302600	-0.33124300
C	-4.82201200	-0.95049000	-1.57922800
C	-5.44772500	-2.31179700	0.32517500
C	-6.04288600	-1.23629600	-2.17447700
H	-4.10654500	-0.29396500	-2.07459600
C	-6.66913300	-2.58663100	-0.27668900
H	-5.20462700	-2.71488800	1.30770700
C	-6.96736700	-2.05233300	-1.52687200
H	-6.27952100	-0.80823000	-3.14650900
H	-7.39269600	-3.21760300	0.23580200
H	-7.92711100	-2.26399000	-1.99400200
H	-1.86709100	-2.81730300	1.16334000

TS1c'

P	0.56697900	1.47876800	0.56591400
C	0.12559700	2.64428100	1.90357200
C	0.63690300	3.93768200	2.02157000
C	-0.78157900	2.17693000	2.86430000
C	0.26793000	4.73930600	3.09831900
C	-1.13309900	2.97477700	3.94586100
C	-0.60304500	4.25676500	4.06837900
C	1.80179200	0.34289100	1.31867400
C	2.41642200	-0.67657200	0.56494500
C	2.03396000	0.40963100	2.69633200
C	3.24162400	-1.63074700	1.20211200
C	2.83864900	-0.53079000	3.31319300
C	3.43465800	-1.56329900	2.59068900
C	0.07527800	-2.72723400	0.83197000
C	0.65255400	-3.97748500	0.57590100
C	1.01021500	-4.81147400	1.62879000
C	0.25139300	-3.15091600	3.20548900
C	0.81343600	-4.39676300	2.94472500
C	1.47583800	2.39829800	-0.72458500
C	2.63122800	3.13123300	-0.43008000
C	1.02208400	2.34417800	-2.04523900
C	3.28902600	3.83603400	-1.43291100
C	1.69629400	3.02378300	-3.05380100
C	2.82130200	3.78339100	-2.74405600
C	1.13402700	-1.33252900	-1.52927000
C	1.10772300	-1.49429400	-2.91890700
C	2.27252200	-0.75849800	-0.92362000
C	2.16492100	-1.05339000	-3.69279000
C	3.35226000	-0.32035000	-1.72213100
C	3.27929100	-0.44289000	-3.11898800
P	-0.39165500	-1.63634800	-0.55463000
C	3.89843600	-2.73890900	0.40811600
C	5.06808100	-3.38548800	1.13556000
C	4.64699100	-3.79438000	2.53664400
C	4.29121700	-2.55392300	3.34004000
C	4.37905500	0.03741600	-4.03228200
C	4.58198300	0.28828300	-1.08483500
C	5.39127600	0.94001500	-3.34700400
C	5.78593600	0.32825300	-2.01408100
C	-1.53800200	-2.62724300	-1.58927900
C	-2.05886700	-2.04668700	-2.75655200
C	-2.02378500	-3.87544300	-1.18909800
C	-2.98563600	-2.72616000	-3.53646700
C	-2.96004100	-4.55121000	-1.96768800
C	-3.42961900	-3.98841300	-3.14852600
Rh	-1.43437400	0.48302600	-0.30973300
C	-2.37339400	2.42848700	-0.59883900
C	-3.11827300	1.61193800	-1.23715800
C	-2.96794800	-0.51154700	0.56961900
C	-3.51430800	-0.16835500	-0.54609700
H	1.30293600	4.34385400	1.26385100
H	-1.22930800	1.18617900	2.76812100
H	0.66358500	5.75085200	3.17164700
H	-1.83231500	2.59326500	4.68766400
H	-0.88144900	4.88493100	4.91268400
H	1.57720000	1.19548500	3.29403800
H	3.00977700	-0.47096200	4.38907200
H	0.82471500	-4.29736800	-0.45342900
H	1.45398200	-5.78427300	1.42227600
H	0.11210000	-2.81357400	4.23176100
H	1.10594200	-5.04682400	3.76785400
H	3.02819900	3.13792500	0.58627200
H	0.13362400	1.75502200	-2.29019300
H	4.17893700	4.41535400	-1.19311700
H	1.33946100	2.96505700	-4.08055700
H	3.34261500	4.32925500	-3.52869700
H	0.26162200	-1.97489000	-3.40315100
H	2.13085000	-1.18199400	-4.77552300
H	3.13915200	-3.51055000	0.19277100
H	4.22232600	-2.37582800	-0.57569700
H	5.42992900	-4.24720200	0.55971600
H	5.90770300	-2.67196900	1.19714700
H	3.77132200	-4.46141400	2.46905800
H	5.43806300	-4.35891400	3.04662900
H	3.78405600	-2.82457200	4.27886200
H	5.22010900	-2.04251400	3.64517000
H	3.92572400	0.54013200	-4.90013600
H	4.89889800	-0.84550000	-4.44185500
H	4.33714100	1.31565700	-0.76896900
H	4.83908800	-0.24054300	-0.15791600
H	6.26258100	1.09096900	-3.99695700
H	4.94625100	1.93344400	-3.16900100
H	6.59887900	0.89253200	-1.53907800
H	6.16559300	-0.69502100	-2.17734100
H	-1.75484400	-1.04072000	-3.05178300
H	-1.68268500	-4.32590600	-0.25932400
H	-3.37648500	-2.25675300	-4.43710700
H	-3.32127700	-5.52569400	-1.64459100
H	-4.15240400	-4.52498700	-3.76107200
C	-0.12529100	-2.32219200	2.15258700
H	-0.54982000	-1.34180600	2.36682700
C	-4.59839800	-0.66176500	-1.42624600
O	-4.85147900	-0.22359300	-2.52560500
O	-5.25219700	-1.65371900	-0.83144700
C	-3.08433100	-1.09504300	1.91124800
O	-2.82152300	-0.49068800	2.92632600
O	-3.48625100	-2.35879100	1.84998600
C	-6.33026800	-2.20814500	-1.59202800
H	-5.94940700	-2.62974800	-2.52881900
H	-6.76057000	-2.98946200	-0.96433500
H	-7.07431700	-1.43774300	-1.81860300
C	-3.62250400	-3.01853800	3.11468100
H	-4.00889300	-4.01239800	2.88655200
H	-2.64571400	-3.08757500	3.60685500
H	-4.31689400	-2.46937100	3.75767100
C	-2.09591400	3.80247100	-0.24824800
C	-1.27861500	4.61541800	-1.04438300
C	-2.68978900	4.34126300	0.90101000
C	-1.06508800	5.94306400	-0.69590500
H	-0.82569200	4.20602500	-1.94544000
C	-2.47652200	5.67103300	1.23720000
H	-3.31660900	3.70486400	1.52425200
C	-1.66339200	6.47543800	0.44285300
H	-0.43588600	6.56792100	-1.32743000
H	-2.94627300	6.08013100	2.13006600
H	-1.49921000	7.51786000	0.70942900
H	-3.81688200	1.47169400	-2.06121700

TS1d’

P	-0.45728200	-1.06121700	-1.11681500
C	-1.46822700	-1.03901100	-2.64141800
C	-2.14910300	-2.15408200	-3.13534100
C	-1.65306000	0.20179800	-3.26995400
C	-2.97659200	-2.03395400	-4.24773800
C	-2.47408200	0.31731500	-4.38320700
C	-3.13876300	-0.80381400	-4.87448400
C	1.27488000	-0.73371400	-1.62002600
C	2.30790000	-0.75435500	-0.66238500
C	1.55686800	-0.35050300	-2.93417400
C	3.60292000	-0.31971100	-1.01673000
C	2.83934700	0.03735900	-3.28132800
C	3.86406000	0.08218200	-2.33707000
C	1.89015300	2.13812300	0.72171200
C	2.83851800	2.53669300	1.67329400
C	3.88587100	3.37418900	1.30587200
C	3.10495600	3.35032100	-0.97703200
C	4.01426100	3.78772000	-0.01881600
C	-0.41090300	-2.78850800	-0.51850700
C	-0.01371000	-3.81057000	-1.39161700
C	-0.70656800	-3.09914900	0.80751400
C	0.03411800	-5.12821400	-0.94967600
C	-0.63951800	-4.41529500	1.25480800
C	-0.28018100	-5.43178700	0.37436600
C	1.38262700	-0.51565200	1.69033700
C	1.28454800	-0.99235600	3.00063500
C	2.07929200	-1.26973200	0.72584400
C	1.83150200	-2.21858000	3.33419100
C	2.61562200	-2.52993500	1.07035500
C	2.47492900	-3.00892400	2.38273500
P	0.52199700	1.03300400	1.21645900
C	4.69460600	-0.23044300	0.02626000
C	6.09376500	-0.15267700	-0.56591100
C	6.14933500	0.92650900	-1.63346400
C	5.22885300	0.54646500	-2.78190500
C	3.01356500	-4.35034600	2.81402700
C	3.30059700	-3.38485700	0.02679900
C	3.35749400	-5.27086700	1.65468100
C	4.15389300	-4.49641500	0.61886600
C	-0.21306500	1.72935600	2.74284000
C	-1.25350600	1.01276900	3.35708300
C	0.09903600	3.00534800	3.22115400
C	-1.94492000	1.55367300	4.43254900
C	-0.60319500	3.54749100	4.29436000
C	-1.62328100	2.82519200	4.90200400
Rh	-1.44082900	0.65685200	0.06048600
C	-3.07330100	-0.60043700	0.43122400
C	-3.67172300	0.52350200	0.31819100
C	-1.62832800	2.84641700	-0.24677200
C	-2.75989400	2.57052000	0.23223900
H	-2.04970400	-3.12012600	-2.64568600
H	-1.14371100	1.08325800	-2.87686300
H	-3.50011200	-2.91090300	-4.62388300
H	-2.60146900	1.28545800	-4.86401800
H	-3.78734000	-0.71551000	-5.74380500
H	0.77502500	-0.35587600	-3.69059400
H	3.05469800	0.32835500	-4.31035500
H	2.76105800	2.18861200	2.70425500
H	4.61033600	3.69395600	2.05245400

H	3.21558500	3.64848200	-2.01888100
H	4.83622200	4.44178400	-0.30557000
H	0.26641000	-3.57476300	-2.41957800
H	-0.97616900	-2.30794600	1.50512900
H	0.33050800	-5.91860100	-1.63687400
H	-0.87305700	-4.64000700	2.29434700
H	-0.23711700	-6.46348100	0.71993200
H	0.77741000	-0.40761700	3.76476600
H	1.74862200	-2.58495600	4.35829000
H	4.50728700	0.67304500	0.63353200
H	4.62975100	-1.06737400	0.73317100
H	6.82165300	0.03835700	0.23316700
H	6.36349400	-1.12523200	-1.01246000
H	5.82498200	1.88484900	-1.19417000
H	7.17179000	1.07405500	-2.00406000
H	5.11121300	1.38230500	-3.48778000
H	5.69627200	-0.26601200	-3.36433300
H	2.28971800	-4.82413300	3.49409100
H	3.91940700	-4.18062000	3.42092500
H	2.51973600	-3.82933000	-0.61458100
H	3.90556500	-2.76653400	-0.64850200
H	3.91253000	-6.14463800	2.01926800
H	2.43157200	-5.64952600	1.18963500
H	4.51166100	-5.15352100	-0.18437000
H	5.05048800	-4.06319400	1.09458300
H	-1.53646600	0.02635700	2.98604600
H	0.88816500	3.59099500	2.75333700
H	-2.74282400	0.98093700	4.90088400
H	-0.34703000	4.54169700	4.65566900
H	-2.17043100	3.25202600	5.74016500
C	2.04292100	2.53287700	-0.60620200
H	1.33570400	2.18668400	-1.35830500
C	-3.45590400	-1.99366000	0.68014500
O	-3.59191600	-2.44782300	1.79039700
O	-3.63959400	-2.65371000	-0.46191100
C	-5.08357300	0.95977300	0.32024000
O	-5.50277600	2.08922500	0.43640700
O	-5.86104100	-0.11699500	0.20560500
C	-7.26851800	0.14266900	0.25602800
H	-7.56133400	0.80711000	-0.56288100
H	-7.74922600	-0.83106700	0.15459000
H	-7.53502900	0.60832700	1.21008700
C	-4.00124000	-4.02987600	-0.32345500
H	-4.90561100	-4.13003200	0.28449800
H	-4.17974200	-4.39070700	-1.33861600
H	-3.18248700	-4.58801900	0.14695300
H	-3.75915200	2.86580000	0.53046200
C	-0.75428800	3.90668200	-0.70465400
C	-0.46002500	4.03920100	-2.06709800
C	-0.25539300	4.85046100	0.20155900
C	0.32708400	5.09271300	-2.51198900
H	-0.87029100	3.31919300	-2.77530000
C	0.54923600	5.88953500	-0.24706900
H	-0.51755600	4.76097000	1.25488900
C	0.84342700	6.01299200	-1.60233300
H	0.53626600	5.19907500	-3.57492700
H	0.93574400	6.61738000	0.46384100
H	1.46427100	6.83542300	-1.95245600

IM2a'

C	-2.24389100	0.95872800	-1.10002000
C	-3.44197100	0.48543300	-0.69553200
C	-4.72428600	1.15201600	-1.01618900
C	-5.67778700	1.42741300	-0.02727600
C	-4.97800100	1.56127700	-2.33015700
C	-6.85268300	2.09605400	-0.35275600
C	-6.15273200	2.22948700	-2.65182400
C	-7.09449200	2.49798300	-1.66300400
C	-2.11735700	-1.33006100	0.14209600
C	-3.35201600	-0.78939000	0.02794000
C	-4.60448000	-1.40661800	0.57630200
O	-5.05799100	-1.15253200	1.66563800
O	-5.17856600	-2.19698100	-0.32854000
C	-6.42350600	-2.76608900	0.07943300
C	-1.81787900	-2.61993800	0.78943000
O	-0.74677300	-3.19947300	0.78449000
O	-2.88973800	-3.11859800	1.42688300
C	-2.69494500	-4.36517100	2.08655500
P	-0.23150400	1.04028500	0.96635000
C	-1.41788100	0.76229400	2.30932600
C	-2.48074700	1.65108600	2.49525100
C	-1.39515300	-0.44216400	3.02631100
C	-3.48513700	1.35393600	3.41004300
C	-2.40086900	-0.73200500	3.93750800
C	-3.44692800	0.16581600	4.13020200
C	1.45531200	0.68715000	1.59736800
C	2.55978000	0.65877600	0.72071000
C	1.66252500	0.49668800	2.96822400
C	3.85614000	0.41940600	1.21720800
C	2.93786900	0.25525800	3.44605400
C	4.03917600	0.19848100	2.59105700
C	2.54994200	-2.45131200	0.09971700
C	3.79348300	-2.84242000	-0.40911900
C	2.24276000	-2.70365800	1.43849500
C	4.72763000	-3.45093000	0.42041300
C	3.18411600	-3.30269000	2.26846900
C	4.42573500	-3.67430800	1.76229900
C	1.21694500	-2.77643800	-2.45203600
C	2.18007500	-2.78557200	-3.46958200
C	0.13865900	-3.66583000	-2.51975100
C	2.06230400	-3.66874800	-4.53503400
C	0.02832800	-4.55273200	-3.58704000
C	0.98712500	-4.55334800	-4.59369700
C	-0.21394100	2.84180900	0.65952300
C	-0.06476900	3.69099600	1.76430700
C	-0.26206300	3.38730200	-0.62371000
C	-0.00759500	5.06781200	1.58462700
C	-0.20837200	4.76644600	-0.79911900
C	-0.08880900	5.60751400	0.30295500
C	1.85310200	-0.04713500	-1.60138600
C	1.52994800	0.32265000	-2.92111600
C	2.36752400	0.94726900	-0.73204500
C	1.83130800	1.59277500	-3.38698900
C	2.69141400	2.22922100	-1.22027300
C	2.44408100	2.54162200	-2.56903500
P	1.29130700	-1.69621100	-0.98468600
Rh	-0.65476400	-0.28736000	-0.79612600
C	5.03998500	0.37505300	0.27883900
C	6.37025900	0.56525500	0.99226800
C	6.46997700	-0.39756300	2.16352600
C	5.39681800	-0.06930300	3.18962100
C	2.82845300	3.86906700	-3.17481800
C	3.25681000	3.29046500	-0.30403600
C	3.20155100	4.94477700	-2.16446800
C	4.05189600	4.34809200	-1.05619200
H	-2.18475400	1.88859600	-1.67222500
H	-5.49106900	1.12637500	1.00271200
H	-4.24853000	1.32979900	-3.10641200
H	-7.58298300	2.30930900	0.42607300
H	-6.33818000	2.53366600	-3.68049000
H	-8.01740500	3.01775000	-1.91363300
H	-6.76612100	-3.37154600	-0.76082300
H	-6.28136300	-3.38572100	0.97184400
H	-7.14553700	-1.97341800	0.30421300
H	-3.64877100	-4.60017400	2.56291000
H	-2.41897500	-5.14560900	1.36956100
H	-1.90148200	-4.28103100	2.83795700
H	-2.53904300	2.57016600	1.91351200
H	-0.59616600	-1.16668300	2.85940500
H	-4.30928700	2.05117400	3.54756500
H	-2.37628400	-1.66958400	4.48956400
H	-4.24501400	-0.07174000	4.82997100
H	0.82880000	0.54375100	3.66557300
H	3.09180900	0.10480300	4.51543400
H	4.03390800	-2.67942600	-1.45979400
H	1.25760900	-2.44346900	1.81932000
H	5.69227800	-3.75582100	0.01793800
H	2.94129900	-3.48956600	3.31322200
H	5.15832700	-4.15237500	2.41078500
H	3.01598900	-2.08585400	-3.44212500
H	-0.60078500	-3.67406300	-1.71864400
H	2.81202200	-3.66783400	-5.32397800
H	-0.81139000	-5.24340100	-3.63113000
H	0.89807100	-5.24402700	-5.42997800
H	-0.00264900	3.27651100	2.77091600
H	-0.32436700	2.73529700	-1.49249700
H	0.09773800	5.72081600	2.44852700
H	-0.26048200	5.18326900	-1.80416700
H	-0.05345400	6.68637800	0.16386100
H	1.09032400	-0.40751800	-3.59961300
H	1.60583500	1.85162700	-4.42228700
H	5.04016500	-0.60391300	-0.23114700
H	4.92803000	1.11870800	-0.52146400
H	7.19496500	0.41656000	0.28348000
H	6.45112300	1.60321400	1.35829400
H	6.32988000	-1.42664600	1.79473300
H	7.46116900	-0.35887200	2.63278400
H	5.30554900	-0.87388500	3.93499200
H	5.69983400	0.82598000	3.75912500
H	2.01844500	4.21332000	-3.83583400
H	3.68886900	3.68892300	-3.84186500
H	2.41926300	3.77609500	0.22771500
H	3.87336800	2.83930800	0.48304600
H	3.72326800	5.76562700	-2.67269900
H	2.29216100	5.37656600	-1.71758600
H	4.38858800	5.12429400	-0.35715300
H	4.96085900	3.89315600	-1.48575900

IM2b’

P	1.18948600	-1.51036300	-1.32823700
C	0.07009700	-1.71190200	-2.74826400
C	-0.50243100	-2.96542700	-3.01206700
C	-0.59935000	-0.54417700	-3.20446300
C	-1.69646000	-3.05135500	-3.71253500
C	-1.81700400	-0.65091300	-3.88833000
C	-2.36769900	-1.89871900	-4.13192600
C	2.34562400	-0.14311800	-1.60792000
C	2.83403100	0.49552500	-0.44880800
C	2.59169400	0.40130200	-2.87080900
C	3.56743800	1.69229000	-0.56649700
C	3.29359100	1.58957800	-2.97383300
C	3.77192700	2.25155200	-1.84021200
C	0.47195400	2.58099600	0.98598500
C	0.79052700	3.34822100	2.11420600
C	1.14657100	4.68346400	1.96924700
C	0.90717600	4.49130300	-0.42553800
C	1.20058400	5.25640200	0.69921800
C	2.06114500	-3.05589500	-0.98277500
C	2.92183500	-3.62415500	-1.92913100
C	1.91327400	-3.64101800	0.27718100
C	3.62779500	-4.77783700	-1.61119600
C	2.62624100	-4.79342800	0.59158900
C	3.48012100	-5.35879500	-0.35199300
C	1.62735700	0.03985100	1.77836000
C	1.70916300	-0.44485900	3.08601700
C	2.72082800	-0.15337500	0.90199700
C	2.80391800	-1.19121700	3.49041000
C	3.82241000	-0.93204500	1.31804700
C	3.84349400	-1.48318100	2.61170500
P	0.06335700	0.81584600	1.20037400
C	4.12641200	2.36939700	0.66309200
C	5.20890400	3.39158500	0.34723600
C	4.73672300	4.33079400	-0.74932100
C	4.52352700	3.54509900	-2.03370000
C	4.97065100	-2.36175300	3.09373700
C	4.96820600	-1.19841900	0.36588000
C	5.85184900	-2.88593200	1.97179700
C	6.21679600	-1.73760200	1.04785300
C	-1.19521900	0.73576000	2.50939600
C	-1.46908400	-0.46633100	3.17553000
C	-2.06053700	1.82160700	2.67590800
C	-2.55223500	-0.55710100	4.03907700
C	-3.15699400	1.71726300	3.52564300
C	-3.39961300	0.53426600	4.21314700
Rh	-0.63920000	-0.11921000	-0.73098100
C	-1.92985600	-1.34736200	0.14051600
C	-3.21817500	-0.94766900	0.12949400
C	-2.31840900	1.05597500	-0.79482400
C	-3.43139500	0.41402500	-0.38279200
H	-0.01666400	-3.87048400	-2.64780700
H	-0.13993400	0.44671300	-3.13330500
H	-2.12404100	-4.03030300	-3.92144800
H	-2.31242700	0.26092400	-4.21628300
H	-3.31659200	-1.98386300	-4.65686100
H	2.21907600	-0.09613400	-3.76618500
H	3.47972300	2.02553300	-3.95608400
H	0.75057100	2.90138100	3.10875100
H	1.38239300	5.27921600	2.84876600
H	0.95879200	4.93008200	-1.42129800
H	1.47706900	6.30358400	0.58828200
H	3.03799900	-3.15873400	-2.90947300
H	1.25239800	-3.17829500	1.01282900
H	4.29584500	-5.22574300	-2.34400600
H	2.51656800	-5.25034600	1.57345500
H	4.03647300	-6.26139700	-0.10596300
H	0.90815300	-0.24950900	3.79500800
H	2.84594400	-1.57719100	4.50969600
H	3.29859600	2.86807800	1.19753600
H	4.50955500	1.61547500	1.36536100
H	5.47230400	3.94290300	1.25897400
H	6.12469700	2.87360600	0.01607400
H	3.79155100	4.80421200	-0.43598000
H	5.45681700	5.14070000	-0.92191700
H	3.99983900	4.15335200	-2.78647300
H	5.50441900	3.30777100	-2.48000300
H	4.54997900	-3.18780600	3.68647500
H	5.58920900	-1.77953200	3.79787100
H	4.62634300	-1.93069800	-0.38941800
H	5.21197000	-0.29068500	-0.20312200
H	6.74522100	-3.36788200	2.38860100
H	5.31048400	-3.65548200	1.39587500
H	6.94342300	-2.05028600	0.28707900
H	6.69817900	-0.93653500	1.63319900
H	-0.84401000	-1.34403600	3.00653300
H	-1.89676800	2.74648900	2.12436500
H	-2.74691300	-1.48963100	4.56594400
H	-3.82887100	2.56518900	3.64003900
H	-4.25688900	0.45578800	4.87854300
C	0.54600600	3.15450900	-0.28380900
H	0.31547900	2.56939500	-1.17458200
C	-4.78534900	1.02857600	-0.49552200
O	-5.23756300	1.51497800	-1.50648900
O	-5.44272000	0.96297900	0.66807000
C	-2.28334800	2.35798800	-1.47035900
O	-1.68363400	2.54781700	-2.51447600
O	-2.91295300	3.32447200	-0.79171000
C	-6.79411600	1.41491100	0.62424700
H	-7.37754200	0.79330100	-0.06560800
H	-7.17738600	1.31464600	1.64113500
H	-6.84405800	2.45648100	0.29083800
C	-2.98421200	4.58706200	-1.45280900
H	-3.50939800	5.25442400	-0.76745900
H	-1.97872200	4.96720600	-1.66552600
H	-3.53922000	4.48938800	-2.39177800
C	-4.30779100	-1.87793800	0.48713700
C	-5.45743100	-1.96401500	-0.30961800
C	-4.18894200	-2.72980900	1.58927000
C	-6.46155000	-2.87246500	-0.00375200
H	-5.55721800	-1.32408400	-1.18815400
C	-5.19360200	-3.64193200	1.89216500
H	-3.30522900	-2.65603700	2.22295300
C	-6.33348800	-3.71470700	1.09858200
H	-7.34734400	-2.92952000	-0.63406000
H	-5.08917300	-4.29416200	2.75765800
H	-7.12229200	-4.42535700	1.33780200
H	-1.55355200	-2.33095500	0.43688900

IM2c’

P	-0.59174600	0.78490200	1.20744800
C	-1.88273500	0.57143100	2.47501600
C	-2.76811800	1.62574400	2.73320700
C	-2.14018000	-0.68859700	3.03319200
C	-3.87874000	1.42726500	3.54487400
C	-3.24653000	-0.87480700	3.85358200
C	-4.12133700	0.17762700	4.10535900
C	1.02713800	0.17333900	1.81340400
C	2.19945400	0.27692800	1.02645600
C	1.08506600	-0.47677600	3.04808600
C	3.39561900	-0.32637700	1.47210600
C	2.26683400	-1.05246400	3.48014700
C	3.41960700	-1.01081500	2.70062100
C	2.32509400	-2.46591400	-1.13974400
C	3.29197400	-2.71216100	-2.12269700
C	4.27509800	-3.66921000	-1.90231600
C	3.34792100	-4.12313500	0.28304000
C	4.30097700	-4.37491500	-0.69988300
C	-0.37479900	2.58808300	1.02925200
C	-0.15731200	3.35141600	2.18478300
C	-0.39940500	3.21653800	-0.21437700
C	-0.01145900	4.73016700	2.09134100
C	-0.24520800	4.59579200	-0.30746700
C	-0.06305900	5.35316400	0.84524300
C	1.92989700	0.43547600	-1.48625800
C	2.14552400	1.11127100	-2.69125000
C	2.27408100	1.04572000	-0.26202100
C	2.66644600	2.39277200	-2.67350700
C	2.83505900	2.33899000	-0.25717800
C	3.00327700	3.02296900	-1.47293500
P	1.07406100	-1.17028100	-1.38728100
C	4.64823300	-0.26637700	0.62476400
C	5.91985600	-0.56883400	1.40359100
C	5.75789300	-1.85937100	2.18700100
C	4.66323500	-1.68314700	3.22704600
C	3.57103100	4.41924900	-1.53664600
C	3.25548200	2.98615000	1.04155900
C	3.64691500	5.11604300	-0.18732800
C	4.20863500	4.15598700	0.84727600
C	0.23134500	-1.49778700	-2.96971000
C	-0.62010300	-0.49344100	-3.48287200
C	0.08439900	-2.80686200	-3.44400600
C	-1.59029400	-0.80242300	-4.43740000
C	-0.86989800	-3.09968700	-4.40962700
C	-1.71723900	-2.10583200	-4.89865000
Rh	-1.06955000	-0.19085100	-0.78623600
C	-2.96236100	0.67353900	-0.74155600
C	-3.86985300	-0.18478500	-0.23649000
C	-2.04948600	-1.68676200	0.09297600
C	-3.37413500	-1.47422300	0.21428200
H	-2.60874800	2.60482000	2.28506800
H	-1.50015400	-1.53979000	2.80214200
H	-4.56029800	2.25452600	3.73369000
H	-3.43246300	-1.85773300	4.28319100
H	-4.99577200	0.02236900	4.73411100
H	0.20388300	-0.53737300	3.68064700
H	2.29555200	-1.56199300	4.44383700
H	3.27218400	-2.15284100	-3.05966700
H	5.02407500	-3.86449500	-2.66720400
H	3.37430100	-4.67050800	1.22428900
H	5.07215000	-5.12408300	-0.52940600
H	-0.11544300	2.86640000	3.16116200
H	-0.54994300	2.63491000	-1.12296500
H	0.14270300	5.31976100	2.99285800
H	-0.27830000	5.07537600	-1.28432700
H	0.04324700	6.43441200	0.77490200
H	1.90156900	0.63359600	-3.64005200
H	2.82945700	2.92522500	-3.61159000
H	4.54542200	-0.99674800	-0.19875300
H	4.73573500	0.71083800	0.13179200
H	6.76950800	-0.62590400	0.71111500
H	6.13472200	0.25980400	2.09964900
H	5.49091600	-2.67131100	1.49037500
H	6.69523300	-2.15422200	2.67570600
H	4.38427600	-2.64817000	3.67632300
H	5.05262100	-1.07439200	4.06100700
H	2.98267000	5.01169400	-2.25374300
H	4.58391200	4.35706100	-1.97000000
H	2.35193900	3.33788900	1.56805700
H	3.70437200	2.23706200	1.70862600
H	4.25809000	6.02385100	-0.26868400
H	2.63944600	5.43485500	0.12795000
H	4.37697000	4.65844500	1.80841500
H	5.19085600	3.78429200	0.50890100
H	-0.46577500	0.55402500	-3.20731100
H	0.71395100	-3.60121000	-3.04358900
H	-2.23271000	-0.01498300	-4.82807300
H	-0.96242500	-4.11895500	-4.78002700
H	-2.47034800	-2.34984000	-5.64453500
C	2.35852800	-3.16644700	0.06904700
H	1.61323200	-2.96599000	0.83998600
C	-4.32081100	-2.44232200	0.81971800
O	-5.50728100	-2.24335800	0.92814100
O	-3.70489400	-3.55229900	1.26358400
C	-1.26560900	-2.89836600	0.42742400
O	-0.63152500	-3.06220800	1.44955500
O	-1.31150300	-3.78270500	-0.57368300
C	-4.56412800	-4.51470000	1.87171600
H	-5.30684300	-4.87533500	1.15242400
H	-3.91732900	-5.33120500	2.19810400
H	-5.08822400	-4.07431300	2.72666100
C	-0.70429800	-5.04725700	-0.30974800
H	-0.92226400	-5.66814000	-1.18083300
H	0.37912900	-4.94059500	-0.17709900
H	-1.13682700	-5.48973800	0.59474200
C	-3.27073300	2.01837800	-1.22211700
C	-2.77438500	2.50037000	-2.44257500
C	-4.07383000	2.87513300	-0.45207300
C	-3.06289700	3.78732300	-2.87766300
H	-2.16744200	1.84153400	-3.06555200
C	-4.36160800	4.16333800	-0.88631300
H	-4.46755600	2.51168600	0.49766800
C	-3.85375900	4.62717200	-2.09677900
H	-2.67867300	4.13486200	-3.83555300
H	-4.98838400	4.81079100	-0.27526700
H	-4.08039000	5.63645100	-2.43492500
H	-4.94105200	0.02540400	-0.18647200

IM2d'

P	0.66877300	-0.79045800	1.14798800
C	1.81766700	-0.48815600	2.52387700
C	3.00972000	-1.21897300	2.59300100
C	1.65227900	0.62232500	3.35905700
C	3.99881000	-0.85716300	3.50005300
C	2.63816400	0.97495900	4.27043900
C	3.81659800	0.23733000	4.33857800
C	-1.06666000	-0.75311100	1.74197300
C	-2.15450400	-0.97647800	0.86556300
C	-1.32326100	-0.44301100	3.08194500
C	-3.47607300	-0.79015600	1.32855600
C	-2.62179300	-0.26508900	3.52347800
C	-3.70435300	-0.39219700	2.65671900
C	-2.74883400	1.96074300	-0.67214900
C	-3.89924500	2.00559100	-1.46997200
C	-5.03357300	2.66770300	-1.01501500
C	-3.89035600	3.21547300	1.04299800
C	-5.02783900	3.27093500	0.24247100
C	0.95578200	-2.51351600	0.61676600
C	1.00055400	-3.51976400	1.59100800
C	1.07574800	-2.85278600	-0.73074600
C	1.20369700	-4.84266200	1.21655900
C	1.26796300	-4.17943300	-1.10330700
C	1.34313100	-5.17203400	-0.13076700
C	-1.74692000	-0.63187200	-1.61601000
C	-1.72747200	-1.13420100	-2.92002300
C	-1.99424900	-1.49843800	-0.53265000
C	-1.93817400	-2.48430300	-3.13942900
C	-2.21804300	-2.87109800	-0.76646800
C	-2.17328800	-3.36399900	-2.08123600
P	-1.26045800	1.08822300	-1.24186800
C	-4.64858400	-0.99878400	0.39554100
C	-5.98581600	-1.11452200	1.11186900
C	-6.14219100	0.01918800	2.10976300
C	-5.08733300	-0.12403100	3.19377400
C	-2.39940500	-4.82119500	-2.40048900
C	-2.48571100	-3.81146900	0.38634400
C	-2.32527600	-5.73568800	-1.18803400
C	-3.11848500	-5.12634400	-0.04486100
C	-0.73497500	1.86602700	-2.80847400
C	0.45135800	1.38069000	-3.39529500
C	-1.27106300	3.07199800	-3.26768500
C	1.08158800	2.09505800	-4.41033600
C	-0.64397800	3.77353600	-4.29298800
C	0.53455900	3.29376700	-4.85870800
Rh	1.01792100	0.63915700	-0.56885900
C	3.01155000	0.12389300	-0.47986800
C	3.77755900	1.03294100	0.16087900
C	1.72300900	2.21655500	0.51851000
C	3.04929500	2.16241900	0.71501500
H	3.18612600	-2.05336700	1.91657900
H	0.75491600	1.23326300	3.27738800
H	4.92467800	-1.42694000	3.53951600
H	2.49392900	1.84017300	4.91538100
H	4.59910700	0.52387800	5.03790800
H	-0.50761000	-0.34883000	3.79255400
H	-2.80504400	-0.01039700	4.56805900
H	-3.90379300	1.51715700	-2.44657900
H	-5.92513300	2.70971800	-1.63775100
H	-3.88534400	3.68676000	2.02471700
H	-5.91797600	3.78739000	0.59798400
H	0.88724100	-3.26630800	2.64597100
H	1.02770200	-2.08659900	-1.50507600
H	1.25161700	-5.61850500	1.97804400
H	1.35801000	-4.43166800	-2.15921400
H	1.50447200	-6.20876800	-0.42169000
H	-1.54135800	-0.46770900	-3.76139200
H	-1.92280500	-2.87580400	-4.15746700
H	-4.68747900	-0.14551400	-0.30433600
H	-4.48406500	-1.88169300	-0.23660200
H	-6.79771300	-1.11362700	0.37327400
H	-6.04344300	-2.08002800	1.64243000
H	-6.01340400	0.98163700	1.58634200
H	-7.14399100	0.02861700	2.55756700
H	-5.05164900	0.77103000	3.83284800
H	-5.36620600	-0.95434200	3.86486900
H	-1.68351200	-5.13395400	-3.17552900
H	-3.39519900	-4.91913000	-2.86574200
H	-1.52833600	-4.01925400	0.89611000
H	-3.11288800	-3.32036500	1.14297400
H	-2.69741800	-6.73472200	-1.44848000
H	-1.27556500	-5.85697000	-0.87243900
H	-3.17167100	-5.80916600	0.81288200
H	-4.15727000	-4.94916500	-0.37203200
H	0.88585900	0.41952100	-3.09497700
H	-2.17168700	3.47765400	-2.80791700
H	1.99724100	1.70464200	-4.84959200
H	-1.07644300	4.70706200	-4.64852100
H	1.02544300	3.85349100	-5.65212700
C	-2.74848300	2.56561900	0.58513200
H	-1.85205300	2.52460900	1.20659400
C	3.47291700	-0.99916200	-1.30204700
O	3.14390800	-1.13238700	-2.46687300
O	4.21134300	-1.89198100	-0.62976500
C	5.25539300	1.02584200	0.30330700
O	5.82866100	1.55919100	1.22642100
O	5.87474000	0.39288000	-0.70092700
C	7.29447000	0.32477000	-0.58607400
H	7.58080600	-0.22915300	0.31462200
H	7.64345100	-0.19258900	-1.48169700
H	7.72390500	1.33049400	-0.53250400
C	4.76288400	-2.94151600	-1.42132300
H	5.43115900	-2.52496300	-2.18383500
H	5.32020700	-3.57750800	-0.73107800
H	3.96978200	-3.51434700	-1.91506000
H	3.58265900	2.98881000	1.19235500
C	0.82130400	3.34037100	0.74872800
C	0.47497700	3.75612800	2.04305300
C	0.34110800	4.08079200	-0.34506800
C	-0.34454000	4.86409900	2.23402700
H	0.89347300	3.23224900	2.90073900
C	-0.48782200	5.17604800	-0.15129100
H	0.64636800	3.79800000	-1.35369700
C	-0.83927100	5.56758800	1.13947900
H	-0.58798700	5.18636100	3.24500800
H	-0.84673900	5.73860400	-1.01203600
H	-1.48183600	6.43269000	1.29176700

6.5. Mechanistic Considerations of Oxidative Dimerization

The compound **6ea** exhibits a high HOMO energy, making it susceptible to one-electron oxidation by DDQ, resulting in the formation of a radical cation species (Table S18). In its neutral state, **6ea** possesses a significant negative charge on the C1 carbon, yet there is no notable difference in the spin density of C1 among the radical cation species of **6aa**–**6fa**. Additionally, the molecular orbitals of the HOMO and SOMO for **6aa**–**6fa** (Figure S37) are similarly distributed among their respective orbitals, including C1, with no observable differences. On the basis of these findings, the dimerization reaction leading from **6ea** to **9** is attributed to the high energy level of the HOMO in **6ea** and proceeds via the mechanism illustrated in Figure S38.

Table S18. Structural analyses for the azulene-embedded PAHs [B3LYP/6-31G(d) or UB3LYP/6-31G(d)]

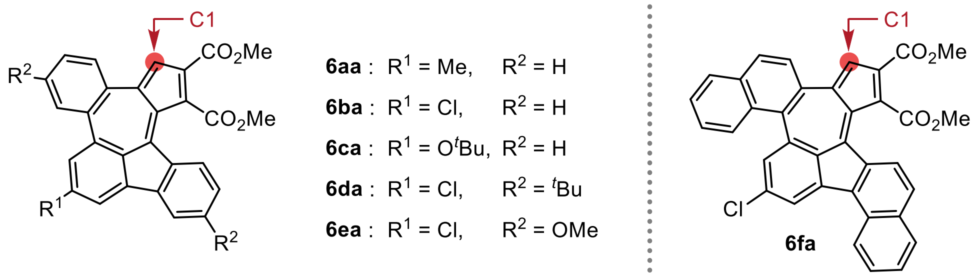

	C1		HOMO (eV)	LUMO (eV)
	charge	spin density		
**6aa** / **6aa**$^{+\cdot}$	−0.209	0.265	−5.491	−2.867
**6ba** / **6ba**$^{+\cdot}$	−0.205	0.256	−5.647	−3.070
**6ca** / **6ca**$^{+\cdot}$	−0.208	0.247	−5.486	−2.858
**6da** / **6da**$^{+\cdot}$	−0.207	0.256	−5.526	−2.968
**6ea** / **6ea**$^{+\cdot}$	−0.216	0.255	−5.322	−2.876
**6fa** / **6fa**$^{+\cdot}$	−0.205	0.273	−5.513	−3.095

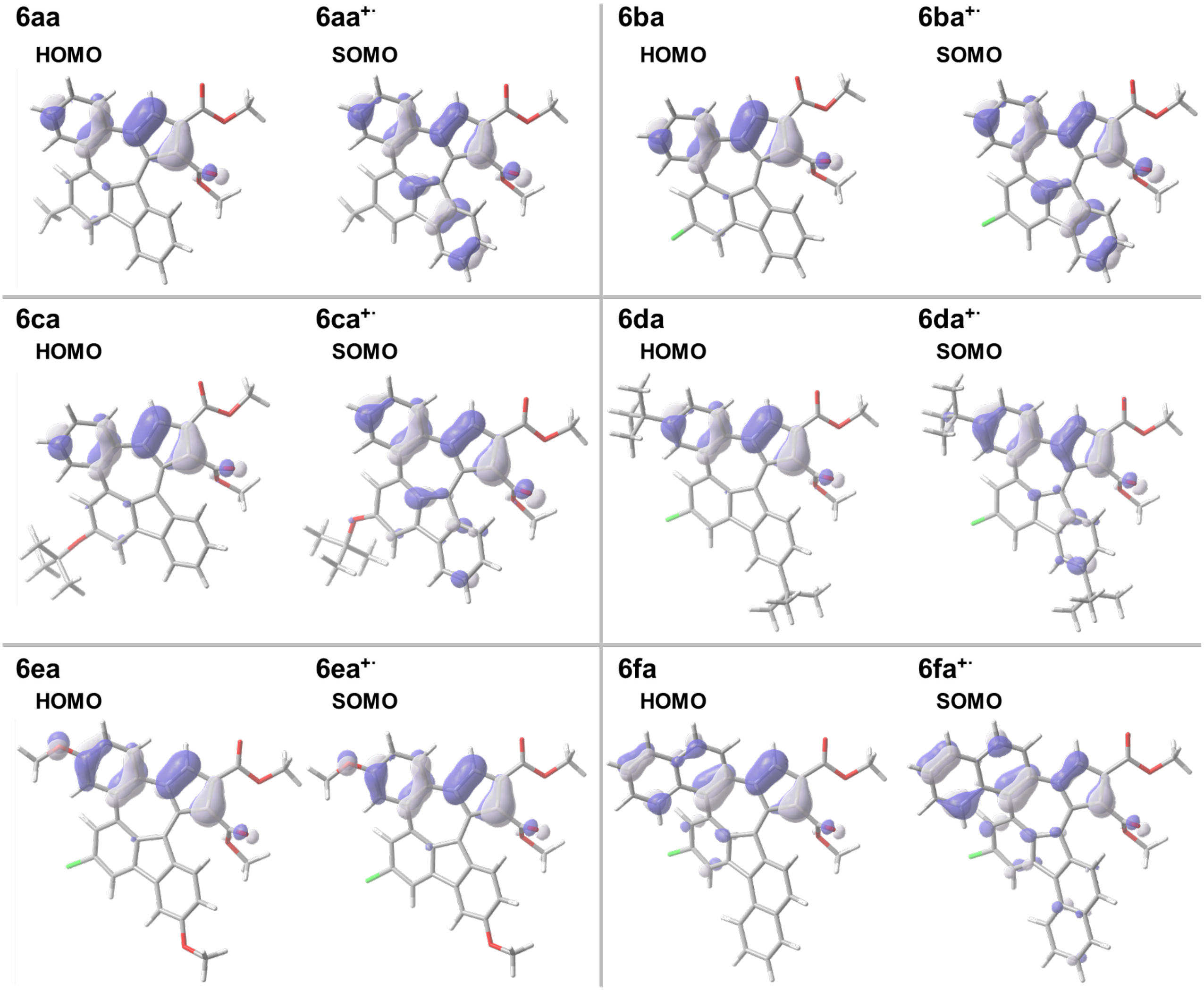

Figure S37. Molecular orbitals of azulene-embedded PAHs

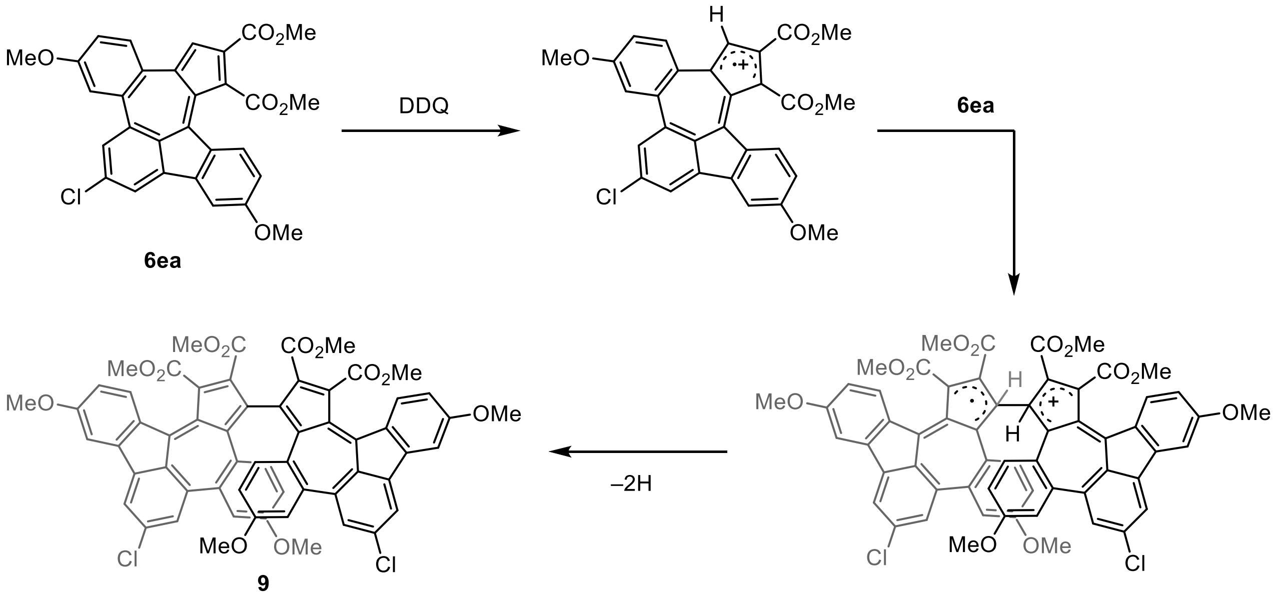

Figure S38. Plausible mechanism of dimerization of **6ea** to **9**.

Cartesian Coordinates of the Computed Structures

Structures of **6ba** and **6fa** were shown in section 6.2.

6aa

C	-2.32158700	-2.10107700	-0.16847000
C	-2.64172700	-0.73638800	0.30040300
C	-1.87402900	0.40231100	-0.00494900
C	-0.48799100	0.56347800	-0.45859700
C	0.48760200	-0.39640800	-0.30417100
C	0.23727500	-1.85409700	-0.17782900
C	-1.00718100	-2.58941600	-0.41838700
C	-0.87405300	-3.91059300	-0.90866500
C	-1.95961300	-4.73979800	-1.13222000
C	-3.24996900	-4.25686500	-0.89671900
C	-3.41280300	-2.96092800	-0.43588500
C	-3.86251700	-0.50250900	0.98403800
C	-4.35419900	0.76694600	1.27341400
C	-3.62891300	1.89269400	0.83440100
C	-2.41485600	1.70417900	0.20448400
C	-1.48248300	2.68620100	-0.36436300
C	-0.33333100	1.99146000	-0.80917100
C	-1.61903400	4.05243000	-0.57838500
C	-0.61170800	4.72644100	-1.28024300
C	0.49485000	4.03291800	-1.77735200
C	0.63953900	2.65786100	-1.55416200
C	1.93074800	-0.20320700	-0.15364000
C	2.48754900	-1.44687800	0.07395300
C	1.45646800	-2.44894400	0.02496500
C	-5.64661500	0.94568600	2.03284200
C	2.68184200	1.07827600	-0.02867700
O	3.54135100	1.46443200	-0.79326100
O	2.29172100	1.76595700	1.06657400
C	2.96275700	3.01903900	1.27497500
C	3.88012800	-1.82085900	0.40189500
O	4.29703300	-2.96334700	0.34585000
O	4.63656000	-0.77312400	0.79052800
C	6.00921700	-1.07757900	1.07595200
H	0.12013000	-4.27184600	-1.14572000
H	-1.80486000	-5.74368500	-1.51792300
H	-4.12035200	-4.87436900	-1.09990600
H	-4.42014800	-2.57895700	-0.31758400
H	-4.44217700	-1.35757400	1.31437100
H	-4.01524700	2.89353200	1.01278000
H	-2.49911100	4.58798800	-0.23237900
H	-0.70652300	5.79333000	-1.46347500
H	1.24736200	4.55975500	-2.35710100
H	1.48989600	2.13292900	-1.97205200
H	1.65961900	-3.50140100	0.16460100
H	-6.35189200	1.57399600	1.47500200
H	-5.47339800	1.43838800	2.99807600
H	-6.13346000	-0.01382800	2.23078900
H	2.56922700	3.40627600	2.21538400
H	4.04315500	2.86784300	1.34040600
H	2.74457700	3.70965600	0.45585600
H	6.47097500	-0.12407400	1.33348400
H	6.08136000	-1.78001200	1.91099100
H	6.49535200	-1.51553800	0.20026200

6aa$^{+\cdot}$

C	2.27099400	2.12864500	-0.16652200
C	2.64143600	0.77991600	0.28630700
C	1.90634800	-0.38556300	-0.01724600
C	0.53369100	-0.58723100	-0.44270900
C	-0.46986600	0.37359700	-0.29443300
C	-0.26771800	1.80973700	-0.24603000
C	0.92482900	2.59171600	-0.39864600
C	0.74011000	3.95017700	-0.80066700
C	1.79450200	4.81120400	-1.00994900
C	3.10324700	4.34720300	-0.81374400
C	3.32110900	3.04075200	-0.40284600
C	3.88277300	0.58489400	0.94815300
C	4.41624600	-0.66999300	1.22012800
C	3.71706700	-1.82174800	0.78092600
C	2.49383600	-1.67375700	0.16926800
C	1.57265900	-2.67876200	-0.38418300
C	0.39625100	-2.00566800	-0.79844400
C	1.73561700	-4.03480400	-0.61198000
C	0.72281800	-4.72428500	-1.30038000
C	-0.40741900	-4.05404700	-1.77159600
C	-0.57939500	-2.68434000	-1.53310400
C	-1.89585900	0.14960200	-0.10238300
C	-2.53304600	1.41042600	0.02726900
C	-1.57188400	2.40151500	-0.08712200
C	5.72167000	-0.82024600	1.95534000
C	-2.62614900	-1.14749300	0.05524700
O	-3.40944300	-1.54947100	-0.77653600
O	-2.30605100	-1.75456500	1.19647100
C	-2.99519600	-3.00152600	1.46288800
C	-3.95552400	1.74744300	0.31641000
O	-4.38653100	2.87477300	0.19708900
O	-4.65874100	0.68873200	0.73369300
C	-6.05536000	0.93801400	1.01720800
H	-0.26219900	4.30585600	-1.00060200
H	1.61117800	5.82778800	-1.34225900
H	3.95058100	5.00108400	-0.99600300
H	4.34388500	2.70109100	-0.30307000
H	4.43927200	1.45235000	1.28249900
H	4.14013000	-2.80772200	0.95401300
H	2.63169900	-4.55799400	-0.29157600
H	0.83722500	-5.78679300	-1.49322500
H	-1.15593100	-4.59249100	-2.34422200
H	-1.44293700	-2.17581900	-1.94268700
H	-1.79988300	3.45314400	0.00994800
H	6.44693500	-1.38132000	1.35367600
H	5.58080400	-1.37921100	2.88851400
H	6.16240700	0.14836000	2.20443400
H	-2.67046900	-3.29935200	2.45850200
H	-4.07496900	-2.84489800	1.43274900
H	-2.70928100	-3.74875700	0.71944200

H	-6.46572200	-0.02746700	1.30889200
H	-6.14847700	1.66415400	1.82764400
H	-6.55485700	1.31998200	0.12464000

6ba$^{+\cdot}$

C	3.68532000	0.76615500	0.71894300
C	4.25055200	-0.48053100	0.94340000
C	3.60791600	-1.66877500	0.54322000
C	2.35403400	-1.55492500	-0.01486200
C	1.71246900	-0.28824300	-0.16676100
C	2.41092100	0.90584500	0.11345900
Cl	5.80178300	-0.58648400	1.71971500
C	0.33008700	-0.54114000	-0.53487600
C	-0.70216800	0.37713300	-0.33275200
C	-0.55624900	1.82173100	-0.27559500
C	0.59551900	2.65338000	-0.47449000
C	1.96791700	2.24355200	-0.31141600
C	1.45067700	-2.59155100	-0.53709200
C	0.23357900	-1.96124400	-0.89672800
C	1.65805500	-3.93823400	-0.78178800
C	0.64510600	-4.66233300	-1.43411000
C	-0.52841400	-4.03339600	-1.85300800
C	-0.74419000	-2.67290300	-1.59644200
C	0.33842500	4.00624700	-0.85372200
C	1.34755600	4.91025900	-1.10491000
C	2.68073500	4.49703700	-0.97654700
C	2.96920000	3.19596100	-0.58888600
C	-2.10773900	0.09380400	-0.07977300
C	-2.78796500	1.32653300	0.09392800
C	-1.87358300	2.35736500	-0.05138100
C	-4.20924800	1.60250000	0.44943600
O	-4.68813200	2.71318400	0.36302100
O	-4.84999100	0.51171100	0.88255100
C	-6.24195900	0.70059600	1.23116900
C	-2.77937200	-1.23289300	0.09402500
O	-3.57661700	-1.65760400	-0.71283900
O	-2.39316500	-1.83475500	1.21641400
C	-3.02501000	-3.10850400	1.50089100
H	4.23404400	1.64169200	1.03936600
H	4.08767800	-2.62876700	0.69899900
H	2.58618000	-4.42800800	-0.50254900
H	0.79284000	-5.71810300	-1.64034300
H	-1.27833300	-4.59683800	-2.39907900
H	-1.64314800	-2.19625300	-1.96611200
H	-0.68547100	4.32395800	-1.00132900
H	1.10866600	5.92153100	-1.41741400
H	3.49249900	5.18494000	-1.19206200
H	4.00870800	2.89753200	-0.54131800
H	-2.13984500	3.39768300	0.06808000
H	-6.59972100	-0.28428800	1.52699100
H	-6.32713100	1.41223600	2.05516200
H	-6.79535000	1.07299400	0.36685000
H	-2.65014800	-3.39952700	2.48070000
H	-4.11005200	-2.99127600	1.51448800
H	-2.74149900	-3.84010800	0.74124300

6ca

C	2.94775549	0.94494832	0.61748958
C	3.58233685	-0.27684244	0.83214609
C	2.95201575	-1.47594589	0.45242174
C	1.68077106	-1.40187519	-0.08458587
C	0.99687407	-0.16128278	-0.23718479
C	1.66156011	1.05152445	0.03599096
O	4.78159206	-0.31018299	1.49864007
C	-0.39612066	-0.46259717	-0.58420100
C	-1.45295897	0.38729605	-0.34281767
C	-1.34944456	1.86166665	-0.21235942
C	-0.21006867	2.72793816	-0.52608093
C	1.16324092	2.38143699	-0.37683973
C	0.81244464	-2.47308298	-0.58976642
C	-0.43064036	-1.89754811	-0.94108250
C	1.07144594	-3.81729873	-0.82762811
C	0.08862609	-4.58840096	-1.46117778
C	-1.11558559	-4.00929006	-1.86936828
C	-1.38237499	-2.65666241	-1.62134117
C	-0.51774938	4.03307591	-0.97959827
C	0.45401457	4.97866949	-1.25775595
C	1.80181267	4.63643430	-1.11753681
C	2.13413068	3.35928996	-0.69708835
C	-2.85229420	0.04026937	-0.08871618
C	-3.51910805	1.21582356	0.19757664
C	-2.60608931	2.32210381	0.08830652
C	-4.91483544	1.43730088	0.63266238
O	-5.45297480	2.52937954	0.62436581
O	-5.52538297	0.31248869	1.06058013
C	-6.89702681	0.46764108	1.45144765
C	-3.45327196	-1.31428340	0.07364091
O	-4.32150752	-1.78504229	-0.63139723
O	-2.91386570	-1.96212876	1.12890551
C	-3.43235751	-3.28004014	1.36994569
H	3.46877482	1.83680196	0.94161750
H	3.45009698	-2.42465670	0.62139440
H	2.02453888	-4.26102743	-0.55226954
H	0.27719839	-5.63956995	-1.66185391
H	-1.85141382	-4.60794576	-2.39862685
H	-2.30994404	-2.21991038	-1.97070348
H	-1.55900594	4.28717988	-1.14161848
H	0.16580163	5.96482398	-1.61121402
H	2.58480467	5.34825412	-1.36329414
H	3.18271826	3.08910363	-0.65208728
H	-2.90788734	3.34573974	0.26005663
H	-7.23522018	-0.53125512	1.72812870
H	-6.97956428	1.15248358	2.30002029
H	-7.49107653	0.85785811	0.62089437
H	-2.93184834	-3.62807463	2.27410930
H	-4.51484651	-3.24408852	1.51600798
H	-3.20405051	-3.93967473	0.52834893
C	6.05292872	-0.30062362	0.76714207
C	7.09024530	-0.33179625	1.89094897
C	6.18481784	0.98134946	-0.06285553
C	6.17535721	-1.54345698	-0.12248213
H	7.15988153	-1.56468501	-0.60279234
H	5.41791512	-1.54919566	-0.91286157

H	6.06671876	-2.45644474	0.47258208
H	8.10450832	-0.32753023	1.47793494
H	6.96475128	-1.23237307	2.50025172
H	6.97363738	0.54083525	2.54117469
H	7.16629532	1.01437496	-0.54863701
H	6.09000880	1.86626734	0.57536422
H	5.42161026	1.03214757	-0.84619505

6ca$^{+\cdot}$

C	2.87087745	1.43406764	0.27879546
C	3.68818866	0.29227211	0.38992637
C	3.18731195	-0.96724613	-0.03809562
C	1.88406684	-1.03266272	-0.47207937
C	1.02155325	0.10684008	-0.48524826
C	1.55147981	1.39143769	-0.19055792
O	4.89994230	0.54146982	0.88767357
C	-0.31756482	-0.35577941	-0.71538476
C	-1.46847978	0.37174660	-0.37468113
C	-1.55363926	1.81282335	-0.26664131
C	-0.57464425	2.83058449	-0.53094041
C	0.85404239	2.65160726	-0.50832543
C	1.11349655	-2.19071848	-0.95650842
C	-0.22334762	-1.76504329	-1.14609754
C	1.51258871	-3.46951292	-1.30400803
C	0.56685781	-4.33158419	-1.88719994
C	-0.73332706	-3.89650259	-2.13830133
C	-1.14095374	-2.60231670	-1.77861646
C	-1.08444048	4.13106363	-0.82519593
C	-0.26564604	5.19984137	-1.12138137
C	1.12265893	5.01286614	-1.12757023
C	1.65465135	3.76637944	-0.82817833
C	-2.76860785	-0.14859704	0.00313704
C	-3.62475183	0.95005730	0.30046601
C	-2.91231205	2.11915505	0.11337624
C	-5.02364447	0.97036138	0.80897367
O	-5.69127549	1.98297915	0.82555137
O	-5.42470054	-0.22671540	1.25572121
C	-6.78211030	-0.28561504	1.74978717
C	-3.19396156	-1.57259979	0.17635543
O	-3.99913077	-2.09952786	-0.55947436
O	-2.58968008	-2.14274009	1.21848455
C	-2.97012461	-3.51297209	1.49383855
H	3.32024075	2.36817019	0.59003225
H	3.79623082	-1.85861857	-0.00148056
H	2.53604858	-3.80253313	-1.15730930
H	0.86357489	-5.33679711	-2.17104540
H	-1.43924275	-4.55713260	-2.63180543
H	-2.14412376	-2.27423202	-2.01933115
H	-2.15612307	4.27604941	-0.86800039
H	-0.69630127	6.16550075	-1.36556589
H	1.78696794	5.83369566	-1.37996949
H	2.72858079	3.64208840	-0.88285364
H	-3.32608858	3.09814380	0.30747854
H	-6.93992673	-1.32511039	2.03339628
H	-6.89638666	0.37644447	2.61094548
H	-7.47928164	0.01358525	0.96450662
H	-2.44710341	-3.77524893	2.41214678
H	-4.05156370	-3.58143746	1.62540076
H	-2.65734328	-4.15763804	0.66945679
C	6.00252479	-0.41919598	1.13684353
C	7.08019278	0.49687889	1.72463782
C	6.48990699	-1.02452745	-0.18370420
C	5.57618505	-1.46309051	2.17410027
H	6.45119026	-2.05096739	2.46938325
H	4.82271351	-2.16242141	1.80353817
H	5.18168070	-0.97166119	3.06882413
H	7.97555926	-0.08451053	1.96426246
H	6.72158444	0.97560922	2.64061398
H	7.35485223	1.27763285	1.00918553
H	7.41074033	-1.58755695	-0.00067831
H	6.71578725	-0.23317225	-0.90508521
H	5.77232391	-1.71171556	-0.63882643

6da

C	2.12274100	-2.30094600	1.32567600
C	1.23119400	-3.30927000	1.65776800
C	-0.10962700	-3.27584800	1.25621800
C	-0.53363100	-2.14913200	0.57558400
C	0.34136500	-1.05891200	0.30029400
C	1.71582700	-1.16015000	0.58886800
Cl	1.80350800	-4.68390100	2.59129400
C	-0.48136200	0.05261400	-0.19941100
C	-0.11312700	1.37658800	-0.11706500
C	1.28395500	1.87205100	-0.03146300
C	2.52711400	1.12932000	-0.24974600
C	2.74742900	-0.24219300	0.05850000
C	-1.85251700	-1.83792200	0.00898000
C	-1.80716500	-0.52282900	-0.49806800
C	-2.97864500	-2.63826200	-0.14418000
C	-4.09416100	-2.15460600	-0.85059000
C	-4.00934400	-0.86288100	-1.40436500
C	-2.88535200	-0.04928200	-1.24656200
C	3.61637000	1.84902600	-0.78471500
C	4.86375800	1.28538600	-0.99849600
C	5.09804400	-0.06862400	-0.71519200
C	4.02089600	-0.79148200	-0.20323100
C	-0.98236900	2.55166100	-0.01896900
C	-0.16401600	3.65176200	0.14197600
C	1.21343800	3.23465000	0.10089500
C	-0.51071800	5.06667400	0.39780700
O	0.28528400	5.98071700	0.28470500
O	-1.79008800	5.23987300	0.78953100
C	-2.18813800	6.60126400	1.00696700
C	-2.46604300	2.59273400	0.11993800
O	-3.22972200	3.10999400	-0.66843500
O	-2.86444600	1.98403400	1.25788700
C	-4.28288700	1.97736100	1.48580800
C	-5.36917600	-2.99152600	-1.05765200
C	-6.57981900	-2.23595100	-0.45835600
C	6.44587700	-0.76497800	-0.97521800
C	6.97892900	-1.36933100	0.34571800
H	3.15020300	-2.39094600	1.65319200
H	-0.78000300	-4.09451900	1.49379000
H	-2.98030600	-3.64273200	0.26418800

H	-4.83738400	-0.47760000	-1.99168800
H	-2.86413300	0.92530600	-1.71893700
H	3.46050100	2.88382500	-1.06847300
H	5.64605200	1.90687600	-1.41963500
H	4.15592500	-1.85299300	-0.03337900
H	2.03609900	3.92963900	0.19395100
H	-3.24339600	6.55589300	1.27715700
H	-1.60130000	7.04857500	1.81402600
H	-2.04951900	7.19142400	0.09735300
H	-4.41824200	1.50548100	2.45954600
H	-4.67363200	2.99795300	1.49255800
H	-4.79161000	1.40182900	0.70766100
C	-5.28264100	-4.37209000	-0.37947500
C	-5.60078400	-3.21187900	-2.57212200
C	7.50708400	0.21155300	-1.51738100
C	6.25287700	-1.89478800	-2.01471800
H	-6.21583100	-4.92161100	-0.54491500
H	-5.13784000	-4.28721600	0.70362700
H	-4.46742200	-4.97926300	-0.78918700
H	-7.49870400	-2.81648400	-0.60403800
H	-6.72563500	-1.25847800	-0.92951100
H	-6.44802300	-2.07393300	0.61759400
H	-6.50904100	-3.80484600	-2.73372500
H	-4.75926900	-3.74927800	-3.02373800
H	-5.72092800	-2.26591800	-3.10989800
H	8.45174800	-0.32099100	-1.67389100
H	7.70239900	1.03185900	-0.81726400
H	7.21069000	0.64494300	-2.47931200
H	7.94029800	-1.86869000	0.17474200
H	6.29008900	-2.11127300	0.76330000
H	7.13018200	-0.59025200	1.10147800
H	7.20658200	-2.40096600	-2.20781900
H	5.88190000	-1.49437400	-2.96499100
H	5.53944700	-2.65045500	-1.66939200

6da$^{+\cdot}$

C	2.10916500	-2.37613000	1.26132300
C	1.21712100	-3.38617400	1.58695500
C	-0.13503600	-3.33469500	1.19740800
C	-0.56278900	-2.20077200	0.54279100
C	0.31670500	-1.10699800	0.28256400
C	1.69517900	-1.21845600	0.55377700
Cl	1.78117600	-4.76718800	2.48388300
C	-0.49987500	0.00765300	-0.18072300
C	-0.09532400	1.34217100	-0.10499300
C	1.28108000	1.81696100	-0.10262500
C	2.51389700	1.10355100	-0.23694500
C	2.72187600	-0.29685000	0.03399900
C	-1.88793100	-1.86000500	0.00049600
C	-1.82803500	-0.53035700	-0.47674600
C	-3.02225400	-2.63698300	-0.15768400
C	-4.14262500	-2.11698700	-0.84417400
C	-4.04260200	-0.81553100	-1.37282200
C	-2.90697200	-0.02170500	-1.20761300
C	3.64334300	1.85484100	-0.67777700
C	4.88037600	1.29027900	-0.89520000
C	5.09123900	-0.08631000	-0.67826600
C	3.99538700	-0.82925500	-0.21773700
C	-0.93743000	2.51810600	0.03722900
C	-0.10196200	3.66251100	0.09431600
C	1.21711200	3.25105700	-0.01967900
C	-0.44371400	5.09601000	0.31267500
O	0.36083900	5.98524200	0.13003800
O	-1.69839300	5.26467300	0.74615300
C	-2.10549100	6.63521900	0.96366100
C	-2.42213600	2.59099100	0.21303600
O	-3.15096300	3.06397300	-0.63086700
O	-2.79986600	2.09124000	1.38924600
C	-4.21844700	2.15536100	1.67471300
C	-5.42902200	-2.93001300	-1.05284400
C	-6.62007100	-2.16092600	-0.42859200
C	6.43563500	-0.77627200	-0.93567500
C	6.93062600	-1.43249500	0.37838300
H	3.13761700	-2.47769800	1.58116600
H	-0.80440400	-4.15447500	1.43377500
H	-3.04148600	-3.65020000	0.22630200
H	-4.86787100	-0.40906200	-1.94784600
H	-2.87314600	0.95589500	-1.67173300
H	3.51840200	2.90560000	-0.90520500
H	5.68385700	1.92004600	-1.25668200
H	4.13393300	-1.89360800	-0.08242900
H	2.05012300	3.93743100	0.02588600
H	-3.14638800	6.57908600	1.27850000
H	-1.48608500	7.09094500	1.73924000
H	-2.00910600	7.20509400	0.03730800
H	-4.32094000	1.78825700	2.69468700
H	-4.57035600	3.18526600	1.59143700
H	-4.76618500	1.52033000	0.97491000
C	-5.35713000	-4.32364200	-0.40020300
C	-5.67356500	-3.11467100	-2.57160600
C	7.51321200	0.21103800	-1.42286600
C	6.24369500	-1.86856900	-2.01874700
H	-6.30078900	-4.85239600	-0.56713600
H	-5.20426800	-4.26332600	0.68354500
H	-4.55948200	-4.93960300	-0.83098200
H	-7.54590500	-2.72751300	-0.57639100
H	-6.75919200	-1.17550400	-0.88490300
H	-6.47920500	-2.02002700	0.64904200
H	-6.59017900	-3.69302400	-2.73122000
H	-4.84563700	-3.65570900	-3.04291400
H	-5.79105700	-2.15804500	-3.09052900
H	8.45165700	-0.32786700	-1.58555100
H	7.71279300	0.99848200	-0.68738100
H	7.24003900	0.68301000	-2.37330000
H	7.89311300	-1.92471700	0.20289300
H	6.23552900	-2.19268300	0.75006300
H	7.07170600	-0.68552100	1.16713100
H	7.19970900	-2.36845600	-2.20808400
H	5.89592900	-1.43478000	-2.96258900
H	5.52493300	-2.63576300	-1.71244300

6ea

C	-3.11719200	1.45017300	1.04348400
C	-2.73798000	2.74559300	1.35984300
C	-1.49019900	3.26225000	0.99215500
C	-0.61248200	2.39549300	0.36706000
C	-0.94325800	1.03343600	0.11291600
C	-2.24736400	0.56272800	0.36086300
Cl	-3.86474700	3.77941700	2.22631200
C	0.28365100	0.35187300	-0.32531000
C	0.48930900	-1.00526000	-0.20242200
C	-0.58210300	-2.03174300	-0.14497600
C	-2.01048400	-1.87156300	-0.41842100
C	-2.78751700	-0.71078200	-0.16267600
C	0.73661900	2.64746600	-0.15543100
C	1.26070600	1.41258100	-0.61370700
C	1.43137900	3.83184700	-0.31005400
C	2.67463000	3.80347000	-0.97307800
C	3.17557800	2.59824900	-1.48824400
C	2.46442700	1.40511300	-1.31559200
C	-2.68481500	-2.99803000	-0.95589300
C	-4.03727500	-3.01493300	-1.21736300
C	-4.79998400	-1.86170600	-0.97216500
C	-4.17134400	-0.73178600	-0.46537500
C	1.75919600	-1.71208000	-0.04103300
C	1.46216100	-3.05193500	0.12387000
C	0.04034500	-3.24274600	0.02793500
C	2.35161600	-4.19187700	0.43340800
O	2.01169000	-5.35577000	0.32418200
O	3.57159500	-3.81340800	0.86982100
C	4.48668900	-4.88419700	1.14202200
C	3.11632900	-1.12970900	0.15636100
O	4.06534600	-1.28910200	-0.58350200
O	3.16947800	-0.39382000	1.28859800
C	4.44272500	0.20792100	1.56996900
O	3.29704500	5.00585800	-1.08497900
C	4.54669200	5.06737300	-1.76076000
O	-6.12131700	-1.94353600	-1.29246300
C	-6.94194800	-0.80524200	-1.07770600
H	-4.10069600	1.11281500	1.34368900
H	-1.22796200	4.29129100	1.21214000
H	1.04696100	4.78654000	0.03522700
H	4.10740000	2.57698600	-2.04058100
H	2.86118000	0.49410700	-1.74683700
H	-2.10092500	-3.87809400	-1.19917600
H	-4.52136700	-3.88828000	-1.64262300
H	-4.74193800	0.17824400	-0.34410500
H	-0.42418400	-4.21455100	0.11813600
H	5.41725100	-4.40210300	1.44271800
H	4.10475800	-5.52140400	1.94452900
H	4.64144700	-5.49373600	0.24786100
H	4.31899000	0.71598400	2.52696100
H	5.22095300	-0.55666100	1.63584200
H	4.70827400	0.92464500	0.78794900
H	4.85525200	6.11302000	-1.71852300
H	5.30128400	4.44380300	-1.26503600
H	4.45101500	4.75766200	-2.80901500
H	-7.94794500	-1.10137600	-1.37967600
H	-6.95190800	-0.51010100	-0.02018700
H	-6.61647300	0.04746800	-1.68794900

6ea$^{+\cdot}$

C	-3.14971900	1.45211400	1.00557200
C	-2.79696100	2.75654100	1.31801500
C	-1.54727600	3.28866300	0.95477400
C	-0.64974900	2.43658600	0.34710000
C	-0.95537300	1.06559800	0.10606100
C	-2.25349200	0.57543400	0.34283100
Cl	-3.93498200	3.77107300	2.16075200
C	0.28059000	0.40452200	-0.31062200
C	0.48504100	-0.97273700	-0.19393600
C	-0.55532500	-1.99666800	-0.20777200
C	-1.96658200	-1.88965100	-0.39088200
C	-2.76695900	-0.70914800	-0.17384200
C	0.70593000	2.69488700	-0.16274500
C	1.24601300	1.45214500	-0.59893300
C	1.38981600	3.87596600	-0.32306800
C	2.64879300	3.84654700	-0.97752500
C	3.16334300	2.63530400	-1.47589100
C	2.46185900	1.44375300	-1.29320100
C	-2.64861600	-3.07467500	-0.82668700
C	-3.98926000	-3.11335100	-1.08927300
C	-4.75877300	-1.93846200	-0.92069100
C	-4.13710600	-0.76747000	-0.46801700
C	1.74290100	-1.66447200	0.00440900
C	1.48208100	-3.05498600	0.07956800
C	0.11805900	-3.25770500	-0.07822900
C	2.40068500	-4.19355600	0.35351200
O	2.06278600	-5.34899900	0.20201400
O	3.59933800	-3.79251900	0.79655300
C	4.54982300	-4.84752400	1.06620600
C	3.10273100	-1.08042400	0.23023100
O	4.00098500	-1.17451800	-0.57717200
O	3.17748500	-0.47299400	1.41611500
C	4.46951200	0.08255200	1.75717000
O	3.25165900	5.03794900	-1.09303400
C	4.51640300	5.13191700	-1.75892200
O	-6.05219600	-2.04677800	-1.22853800
C	-6.92642300	-0.91650700	-1.09832700
H	-4.12888300	1.10166700	1.30440400
H	-1.30635300	4.32326100	1.17315100
H	1.00129900	4.83454600	0.00516100
H	4.09641300	2.61769300	-2.02534400
H	2.86381000	0.53379400	-1.72049400
H	-2.07164000	-3.97126600	-1.00983200
H	-4.47568100	-4.01359300	-1.44854500
H	-4.72916800	0.13172700	-0.37871400
H	-0.33499200	-4.23705000	-0.02676100
H	5.46141900	-4.34031000	1.37875600
H	4.17303500	-5.49803700	1.85861400
H	4.72235900	-5.43601200	0.16275000
H	4.35552400	0.47200900	2.76770300
H	5.23285700	-0.69712900	1.72218700
H	4.72825800	0.88148900	1.05861800
H	4.79207800	6.18484100	-1.70967000
H	5.27292300	4.52667400	-1.24781100

H	4.43027200	4.82070000	-2.80558200
H	-7.90898000	-1.27712500	-1.39976500
H	-6.96126500	-0.57075400	-0.05983800
H	-6.61083500	-0.10363500	-1.76058300

6fa$^{+\cdot}$

C	-2.83832200	1.23806700	1.21637800
C	-2.36739500	2.47160600	1.62662000
C	-1.11996300	2.97229700	1.19909100
C	-0.32088700	2.16256000	0.41347500
C	-0.73284200	0.82487800	0.12249000
C	-2.03625900	0.38519000	0.40940500
Cl	-3.34720600	3.45892900	2.67577400
C	0.43250400	0.11716600	-0.38100200
C	0.58565300	-1.25902300	-0.23077100
C	-0.49544300	-2.22951800	-0.06466800
C	-1.91059700	-2.08216400	-0.20496700
C	-2.62851200	-0.83887200	-0.14241500
C	1.00090200	2.37431500	-0.22196900
C	1.42534300	1.13217400	-0.72798600
C	1.76058800	3.55030800	-0.44888700
C	2.97028900	3.41240400	-1.22818700
C	3.33217900	2.14912600	-1.76804000
C	2.57722700	1.02171000	-1.53841800
C	-2.67100700	-3.28666800	-0.42141100
C	-4.00995700	-3.26961300	-0.65260600
C	-4.71564300	-2.03667400	-0.77773000
C	-4.00720000	-0.80824600	-0.56377000
C	1.83320100	-2.00015300	-0.07751500
C	1.51881400	-3.35362600	0.16176300
C	0.13453100	-3.49824100	0.14599800
C	1.40729200	4.85098100	0.01304000
C	2.20680100	5.93871400	-0.24931400
C	3.40329900	5.79370200	-0.99316800
C	3.76935200	4.55837500	-1.47552900
C	-6.07453900	-2.01402900	-1.14646500
C	-6.73001500	-0.81355100	-1.36218000
C	-6.02637500	0.39997200	-1.23978500
C	-4.70085200	0.40479100	-0.85311200
C	2.40424200	-4.51270400	0.46297900
O	1.99234400	-5.65395100	0.46713300
O	3.66030400	-4.14386300	0.74070100
C	4.58069300	-5.22305400	1.02215300
C	3.23979500	-1.48594500	-0.05476500
O	4.02384800	-1.70883800	-0.95017500
O	3.48954500	-0.79615400	1.05801200
C	4.84283600	-0.30049600	1.20074100
H	-3.81792600	0.90910500	1.53801900
H	-0.81126900	3.95235100	1.53538400
H	4.21530300	2.08842800	-2.39786500
H	2.84986500	0.08311500	-2.00291100
H	-2.15619500	-4.23698200	-0.44117700
H	-4.54764200	-4.20023700	-0.80981100
H	-0.34867500	-4.44621900	0.33138600
H	0.49016800	5.00074500	0.56602400
H	1.91654200	6.92018900	0.11320000
H	4.02382200	6.66285900	-1.18912300
H	4.67739800	4.44275800	-2.06088400
H	-6.59655500	-2.95708000	-1.28095000
H	-7.77509700	-0.80840300	-1.65631700
H	-6.52225500	1.33828600	-1.46758500
H	-4.17073900	1.34795900	-0.81601700
H	5.54401000	-4.74311400	1.18789500
H	4.25721000	-5.76859400	1.91133500
H	4.62738800	-5.90597900	0.17150300
H	4.87485800	0.16190600	2.18605100
H	5.55216100	-1.12742300	1.13255100
H	5.05340600	0.43242200	0.41894900

7. Photophysical Properties

7.1. Absorption Spectra of 6ba, 6fa, 7, and 9 (Figure 8a)

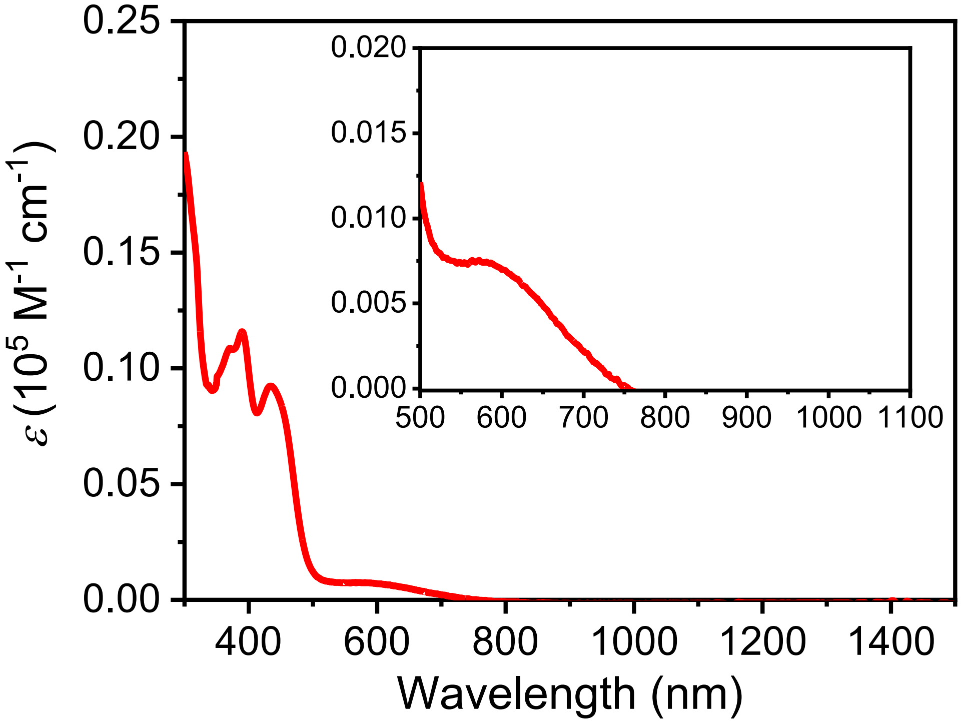

Figure S39. Absorption spectra of **6ba** (1.0 × 10^{-5} M in CH_2Cl_2 at 25 °C).

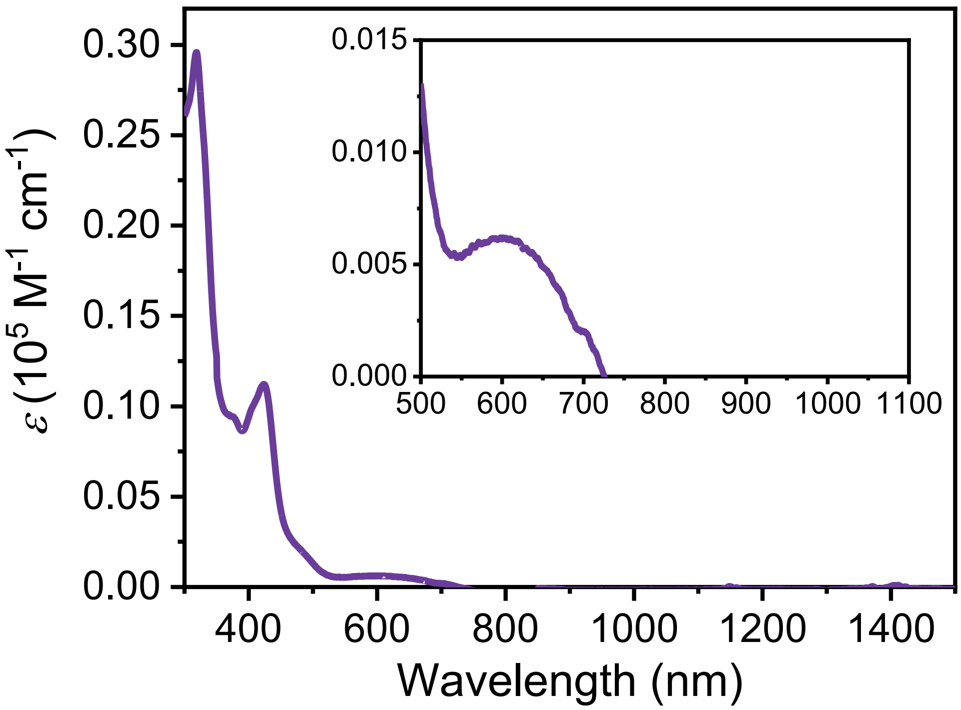

Figure S40. Absorption spectra of **6fa** (1.0 × 10^{-5} M in CH_2Cl_2 at 25 °C).

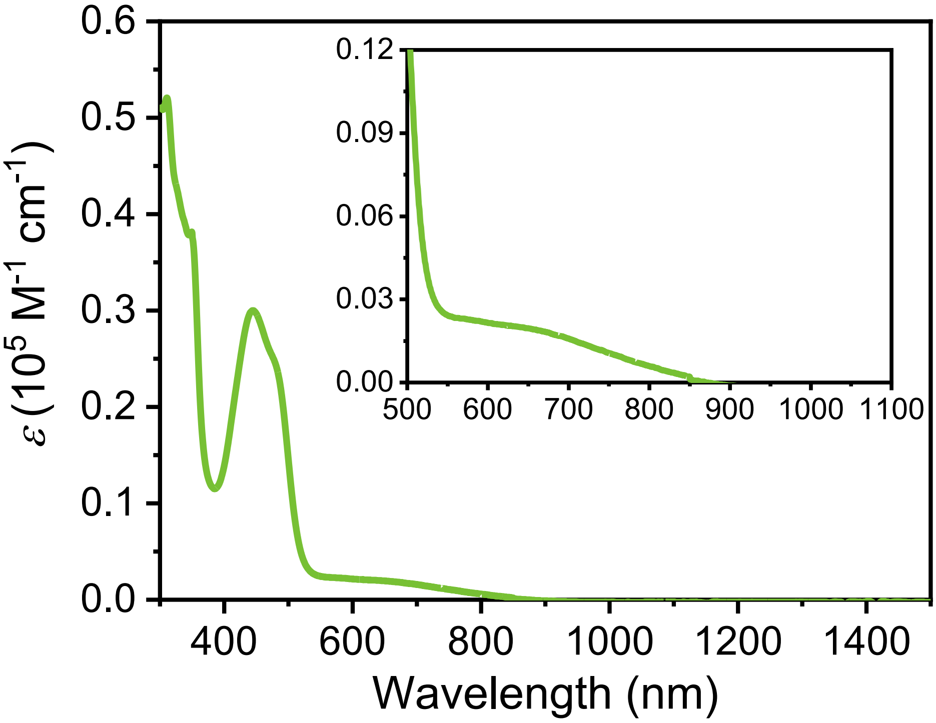

Figure S41. Absorption spectra of **8** (1.0×10^{-5} M in CH_2Cl_2 at 25 °C).

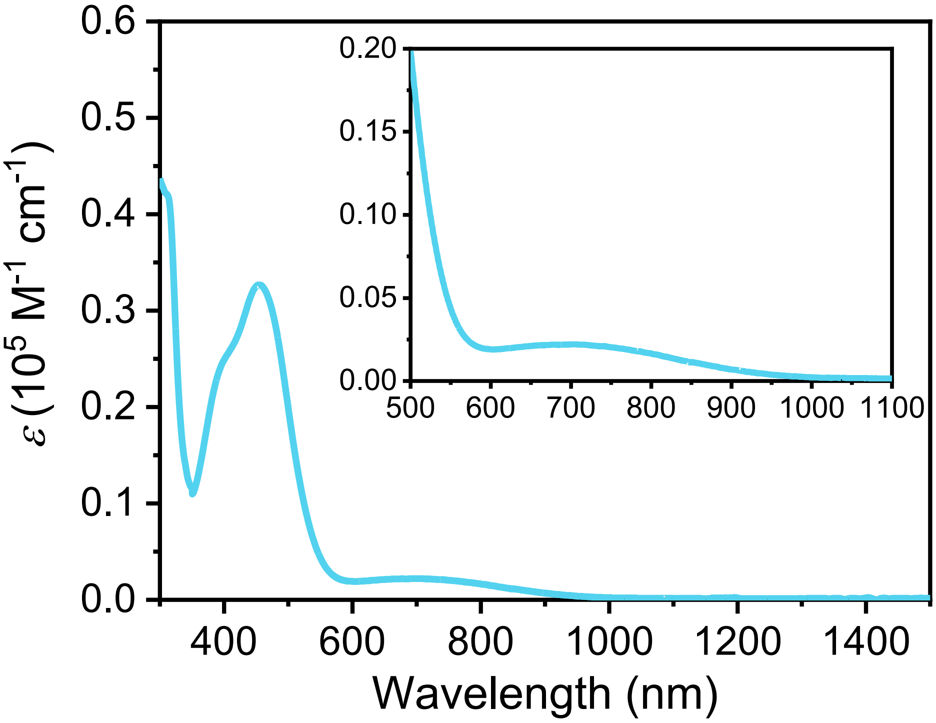

Figure S42. Absorption spectra of **9** (1.0×10^{-5} M in CH_2Cl_2 at 25 °C).

7.2. Absorption Spectra of 6aa

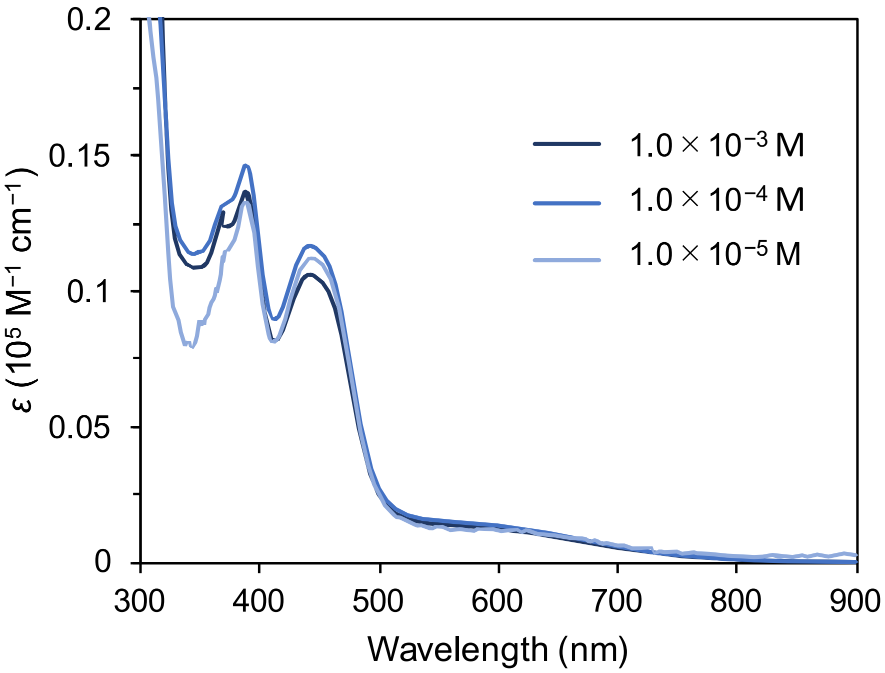

Figure S43. Absorption spectra of **6aa** in variable concentrations (CH_2Cl_2 at 25 °C using 1 mm cuvette).

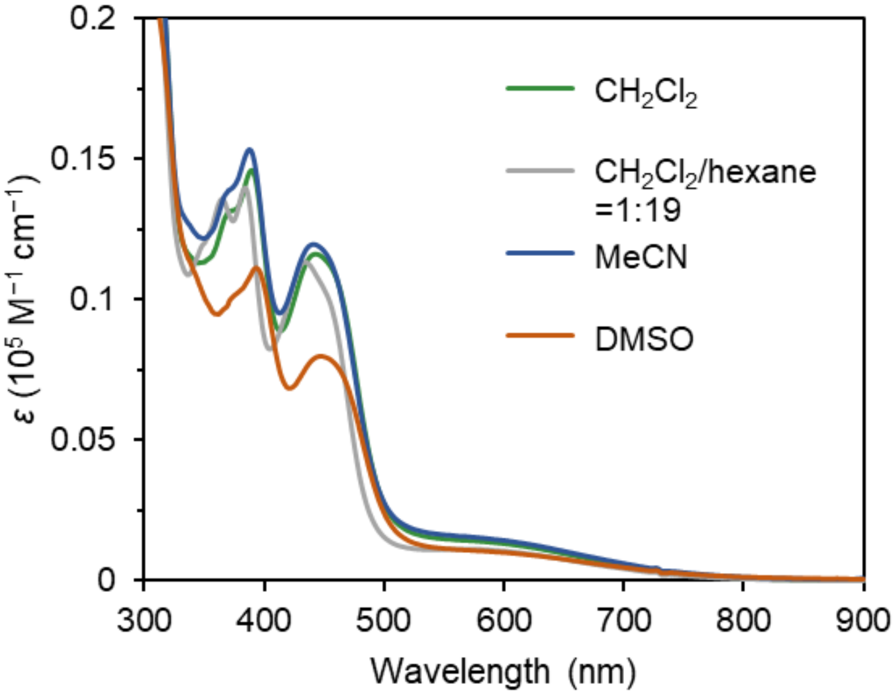

Figure S44. Absorption spectra of **6aa** in variable solvents (1.0×10^{-5} M in CH_2Cl_2 at 25 °C).

Table S19. Absorption spectra of **6aa** in variable solvents.

Solvent	λ_{max1} (nm)	ε ($\times10^5$ M^{-1} cm^{-1})	λ_{max2} (nm)	ε ($\times10^5$ M^{-1} cm^{-1})
CH_2Cl_2	565	0.014	443	0.116
CH_2Cl_2 / hexane =1:19	572	0.011	434	0.114
MeCN	560	0.016	441	0.120
DMSO	572	0.011	447	0.080

8. Electronic Properties (Figure 9)

8.1. Cyclic Voltammetry (CV) Curves (Figure 9)

The cyclic voltammetry measurements were carried out in 0.1 M n-Bu_4NPF_6 solution in dichloromethane using a BAS electrochemical analyzer model 612C at 25 °C using a three-electrode configuration. The working electrode was a glassy carbon electrode. The reference and pseudo-reference electrodes were both Pt wires. The ferrocene/ferrocenium (Fc/Fc^+) couple served as an external reference. For calibration, the redox potential of Fc/Fc^+ was measured under the same conditions. The sample concentrations were 1 mM, and the scanning rate was 100 mV s^{-1}.

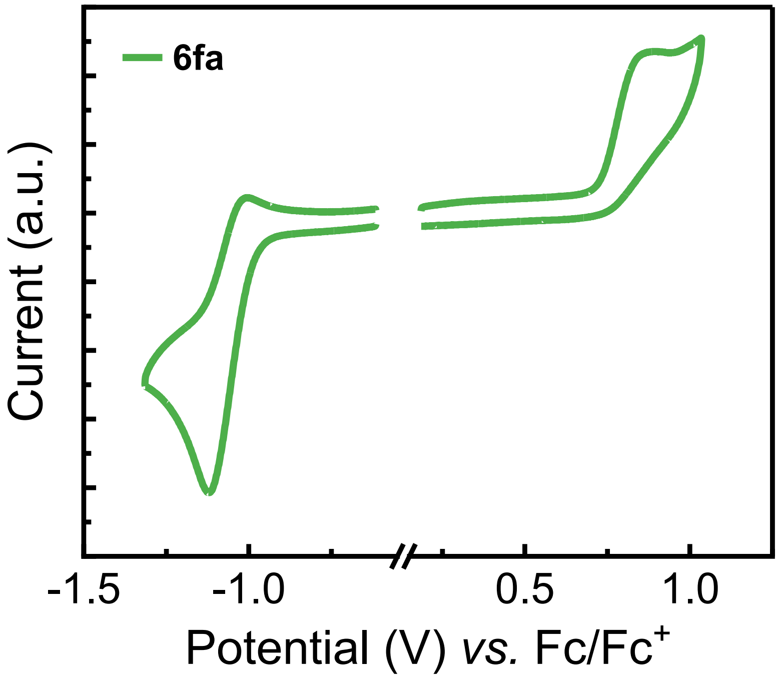

Figure S45. Cyclic voltammogram curves of **6fa**. Positive and negative sweep voltammograms were measured to determine the HOMO and LUMO levels, respectively.

8.2. HOMO and LUMO Energy Levels Based on CV (Table 2)

Assuming that the redox potential of Fc/Fc^+ has an absolute energy level of -4.80 eV to vacuum, HOMO and LUMO energy levels were calculated according to the following equations:

$E_{HOMO} = -(E_{ox,onset} + 4.80)$ eV
$E_{LUMO} = -(E_{red,onset} + 4.80)$ eV

where $E_{ox,onset}$ is the onset of the oxidation peak and $E_{red,onset}$ is the onset of the reduction peak.

Accordingly, the HOMO/LUMO levels were estimated to be –5.52/–3.81 eV for **6fa.** The electrochemical energy gap (E^{EC}_g) of **6fa** was 1.71 eV.

9. ^{1}H and ^{13}C Spectra of New Compounds

5'-Chloro-2,2''-diethynyl-1,1':3',1''-terphenyl (1b)

^{1}H NMR ($CDCl_3$, 400 MHz)

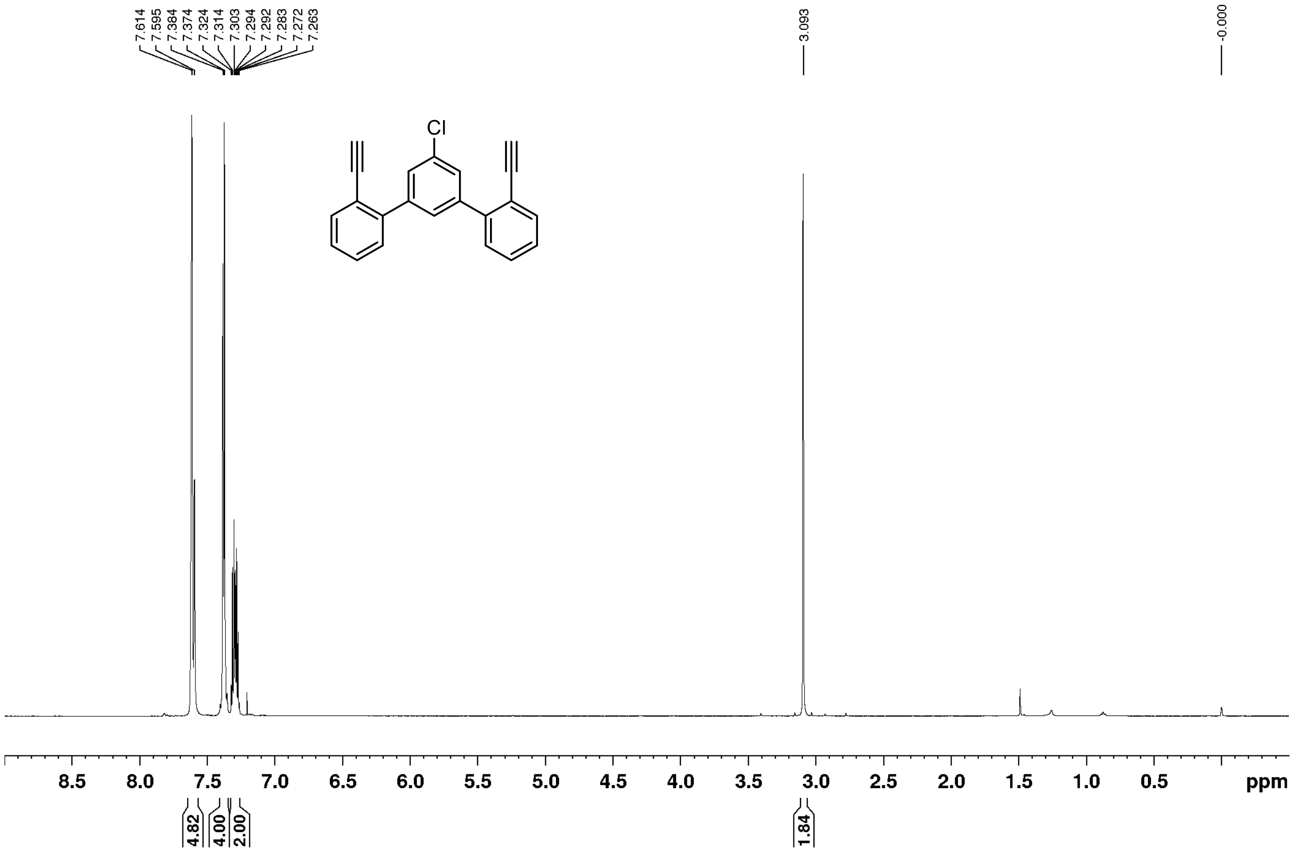

^{13}C NMR ($CDCl_3$, 100 MHz)

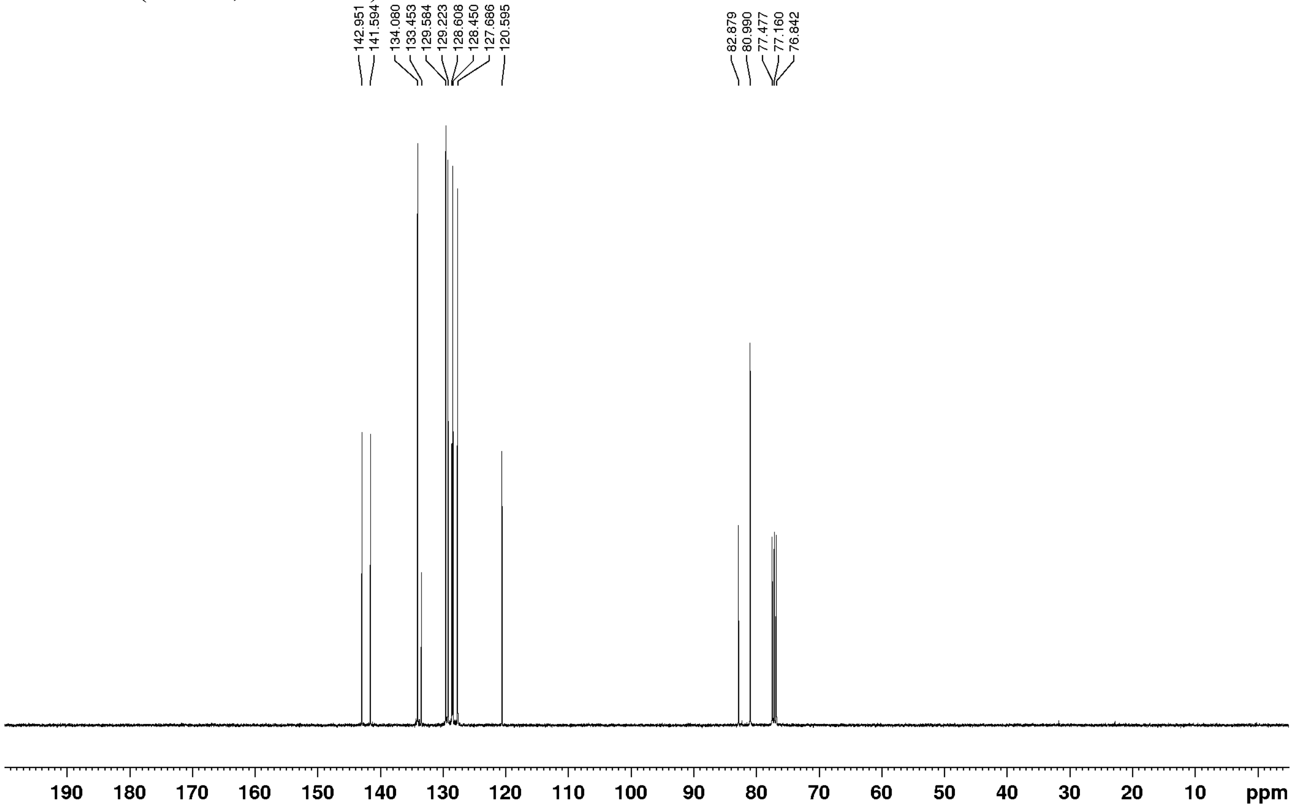

1,3-Dibromo-5-(tert-butoxy)benzene (S5)

^{1}H NMR ($CDCl_3$, 400 MHz)

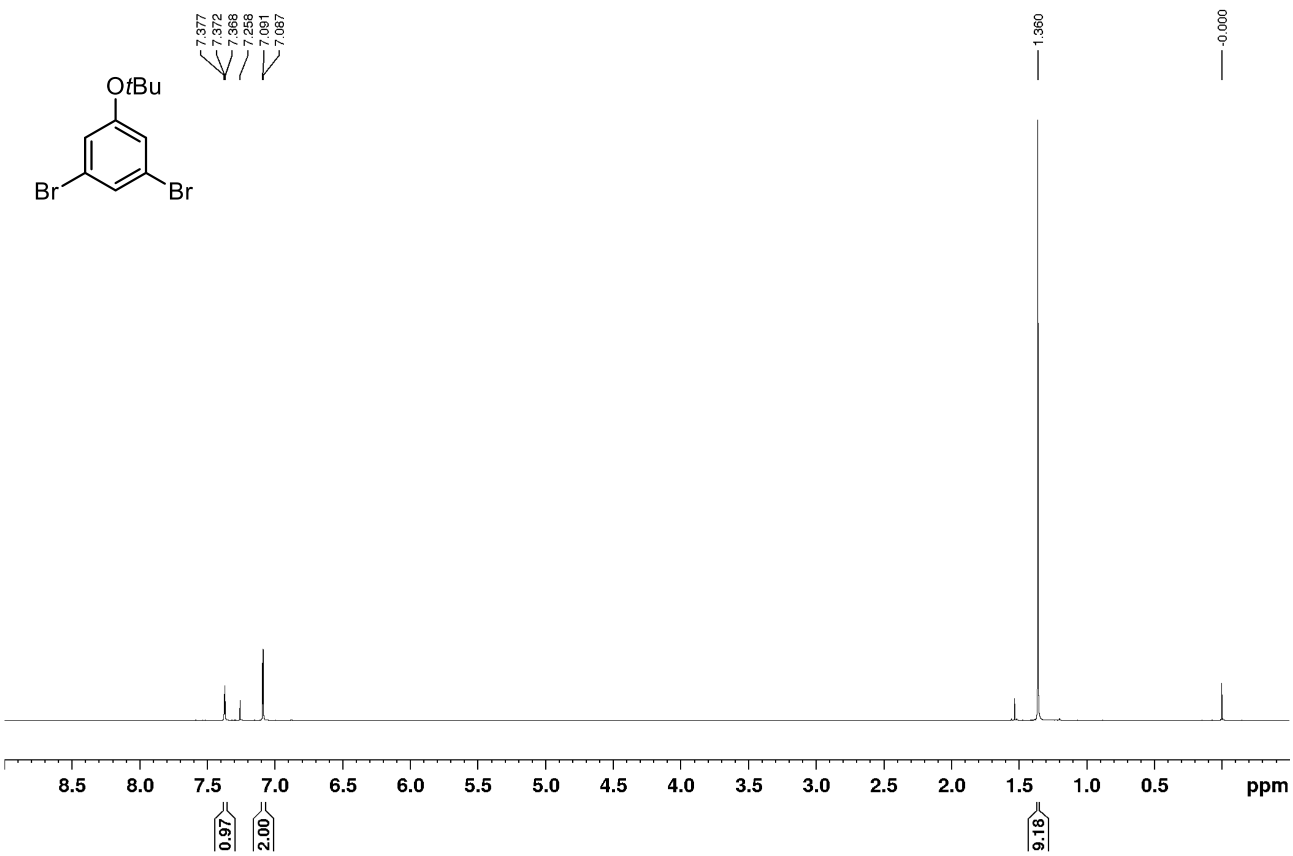

^{13}C NMR ($CDCl_3$, 100 MHz)

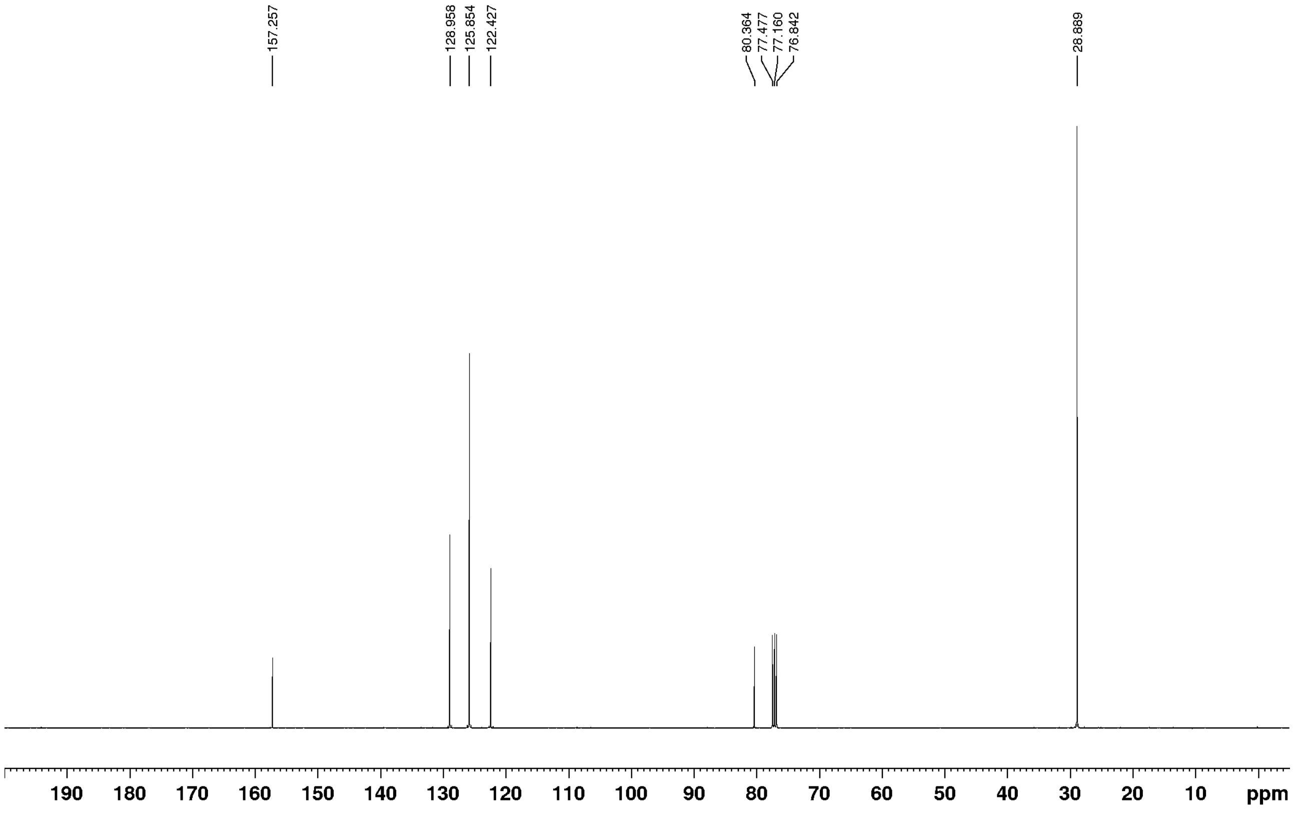

Ethyl 3-(naphthalen-2-yloxy)propiolate (1c)

^{1}H NMR ($CDCl_3$, 400 MHz)

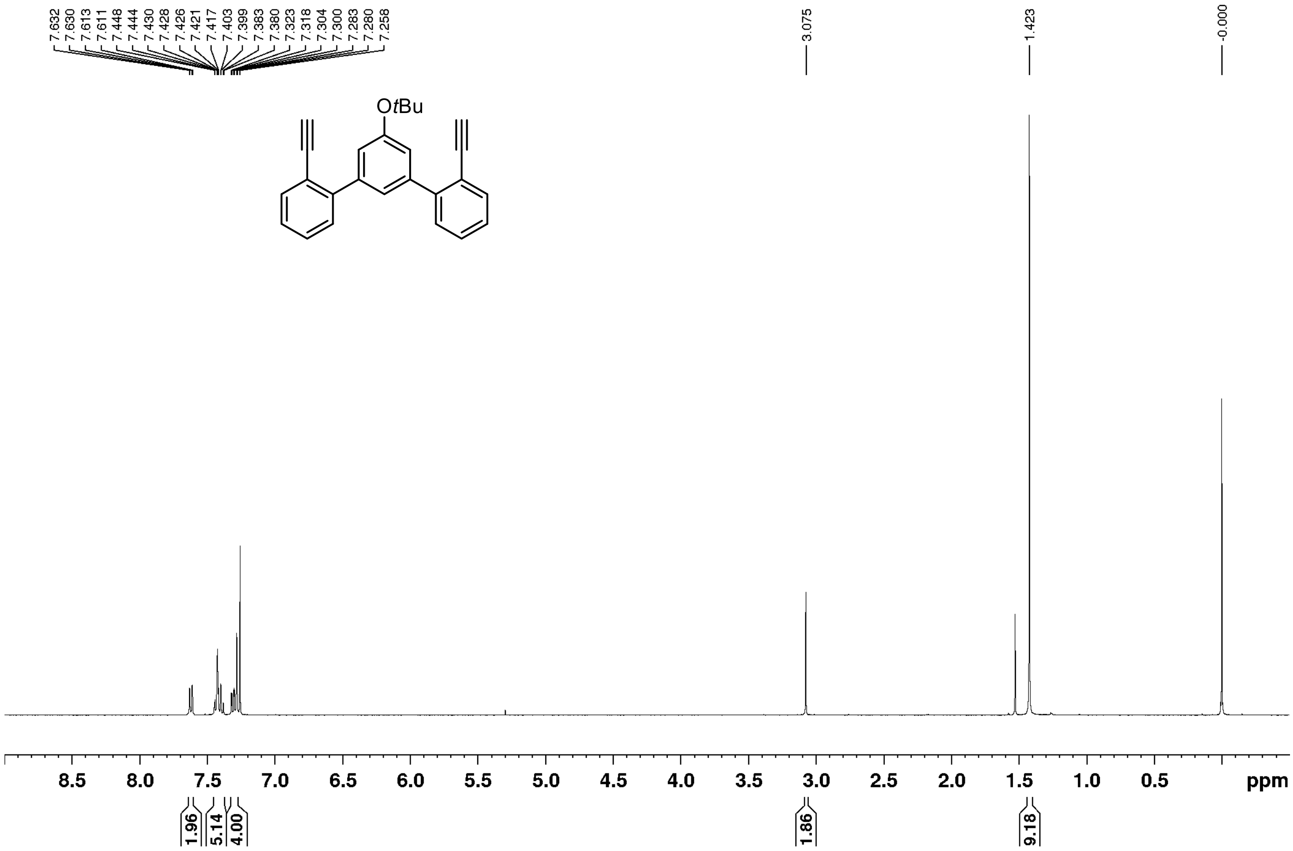

^{13}C NMR ($CDCl_3$, 100 MHz)

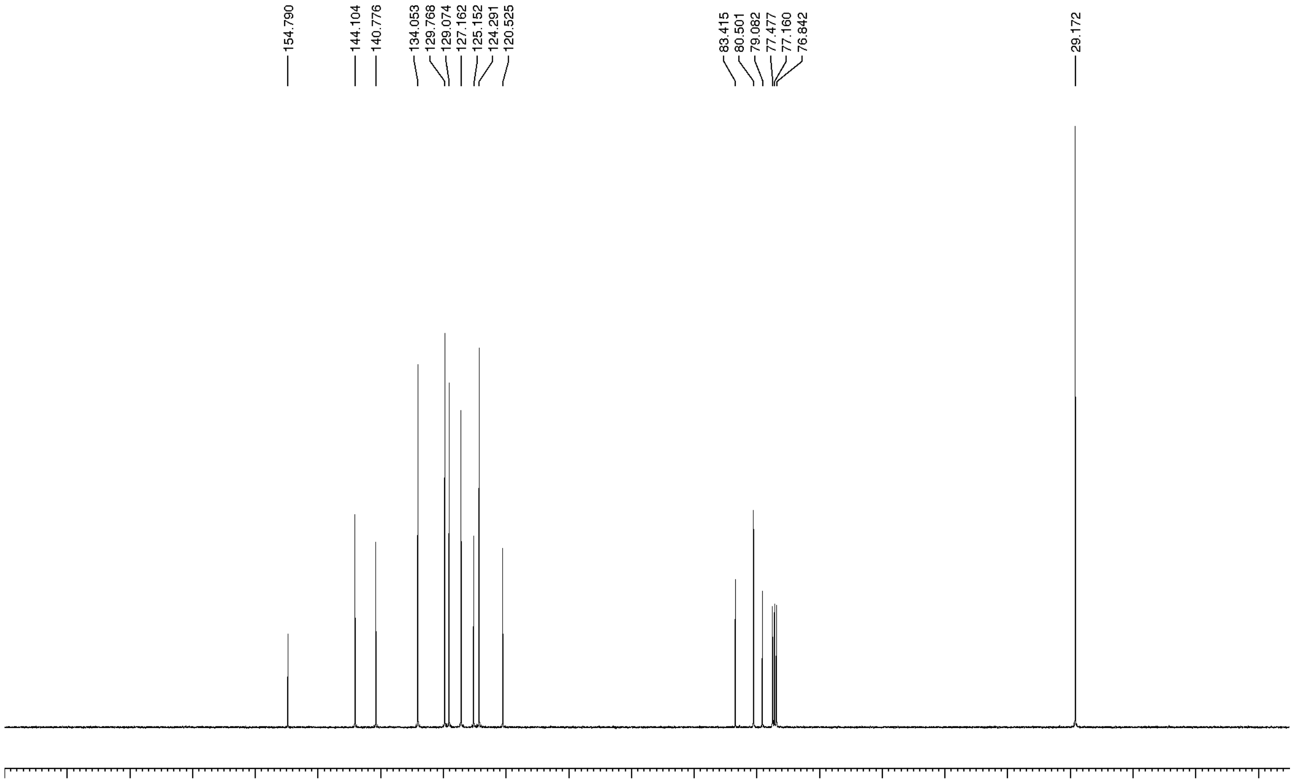

5,5''-Di-*tert*-butyl-5'-chloro-2,2''-diethynyl-1,1':3',1''-terphenyl (1d)

^{1}H NMR ($CDCl_3$, 400 MHz)

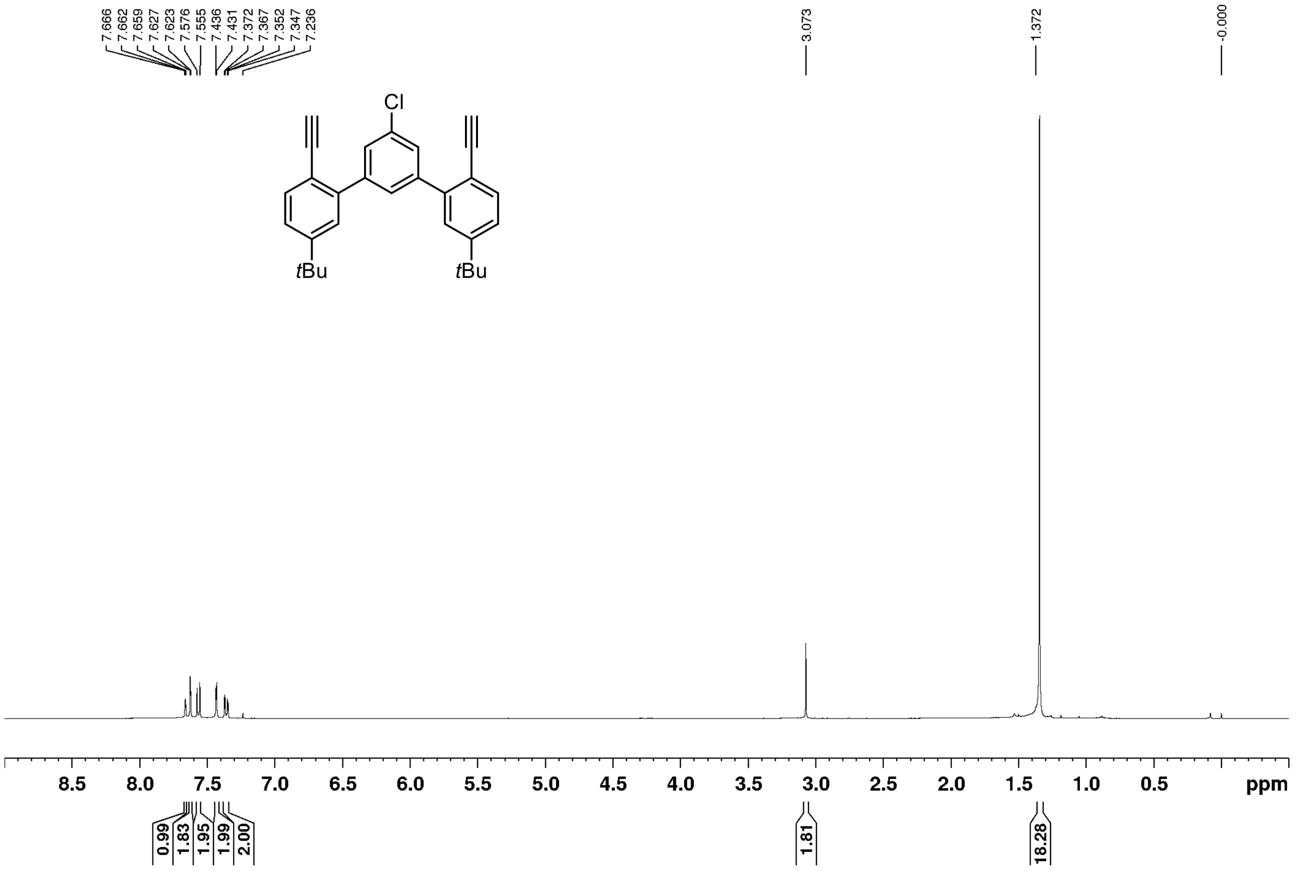

^{13}C NMR ($CDCl_3$, 100 MHz)

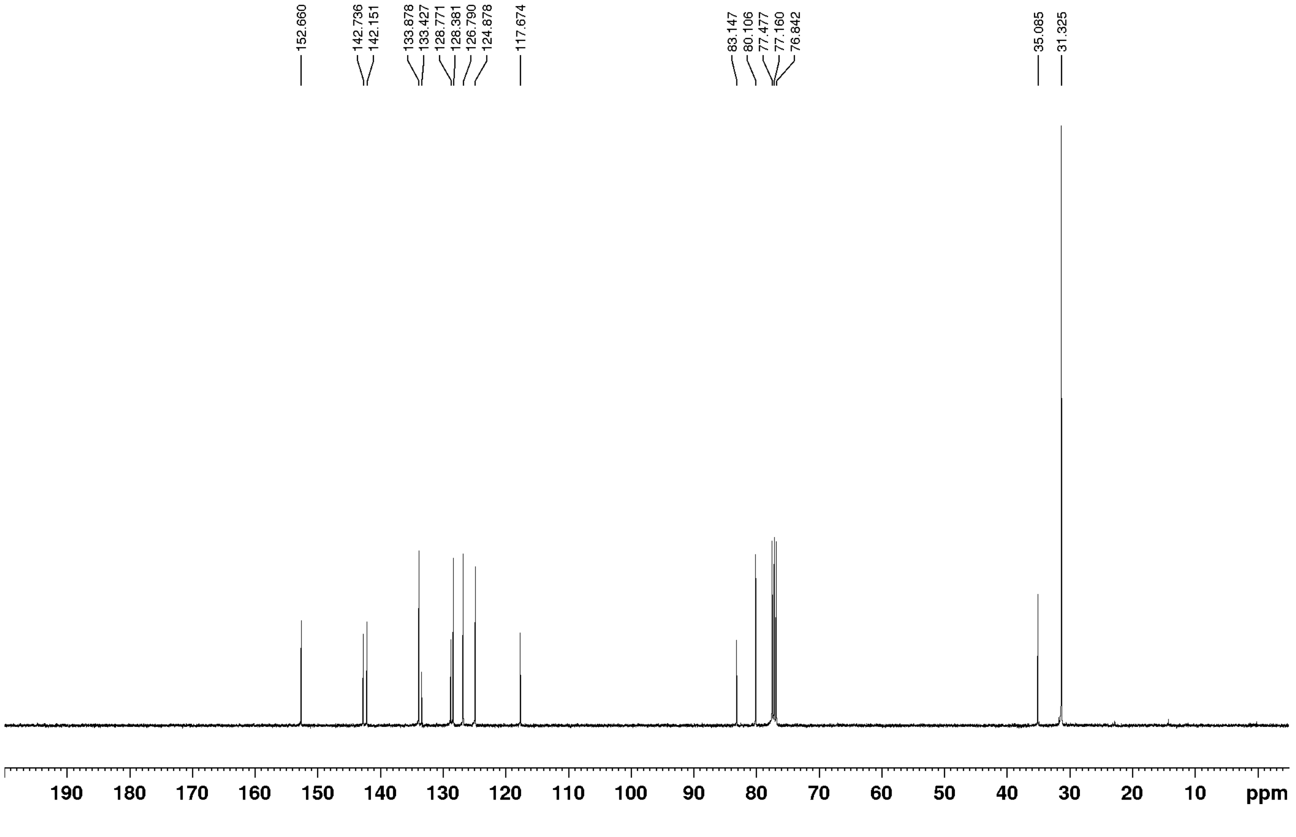

(5-Methoxy-2-((triisopropylsilyl)ethynyl)phenyl)boronic acid (S10)

^{1}H NMR ($CDCl_3$, 400 MHz)

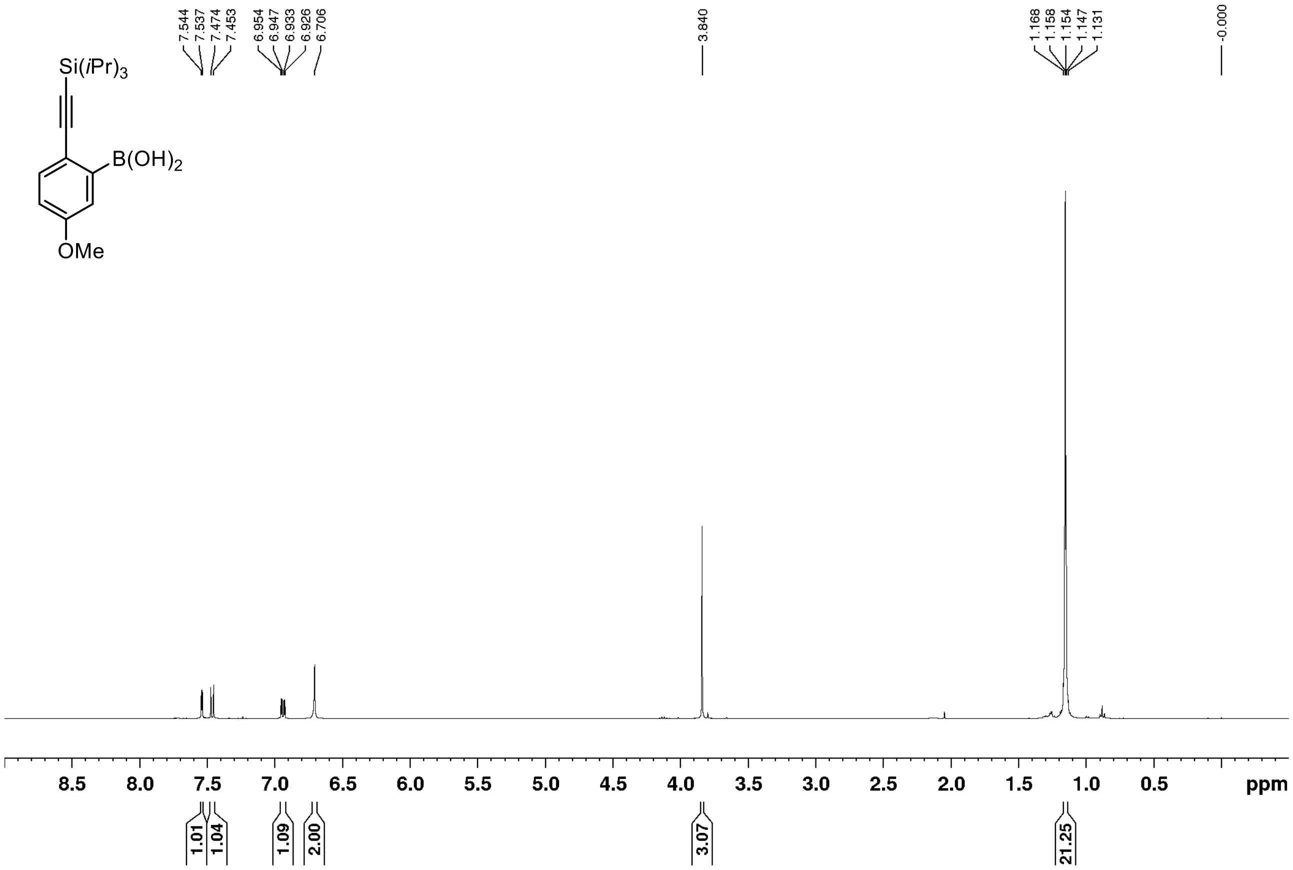

^{13}C NMR ($CDCl_3$, 100 MHz)

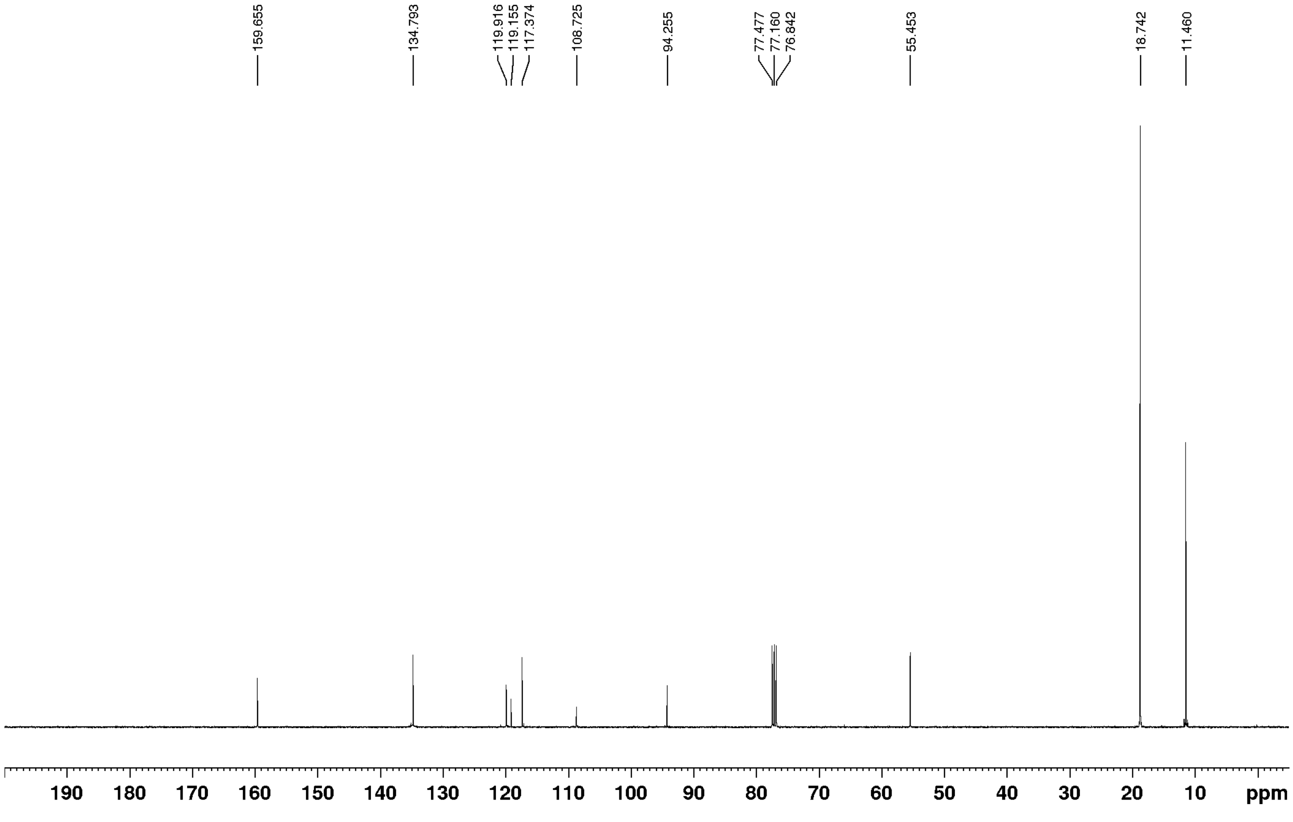

5'-Chloro-2,2''-diethynyl-5,5''-dimethoxy-1,1':3',1''-terphenyl (1e)

^{1}H NMR ($CDCl_3$, 400 MHz)

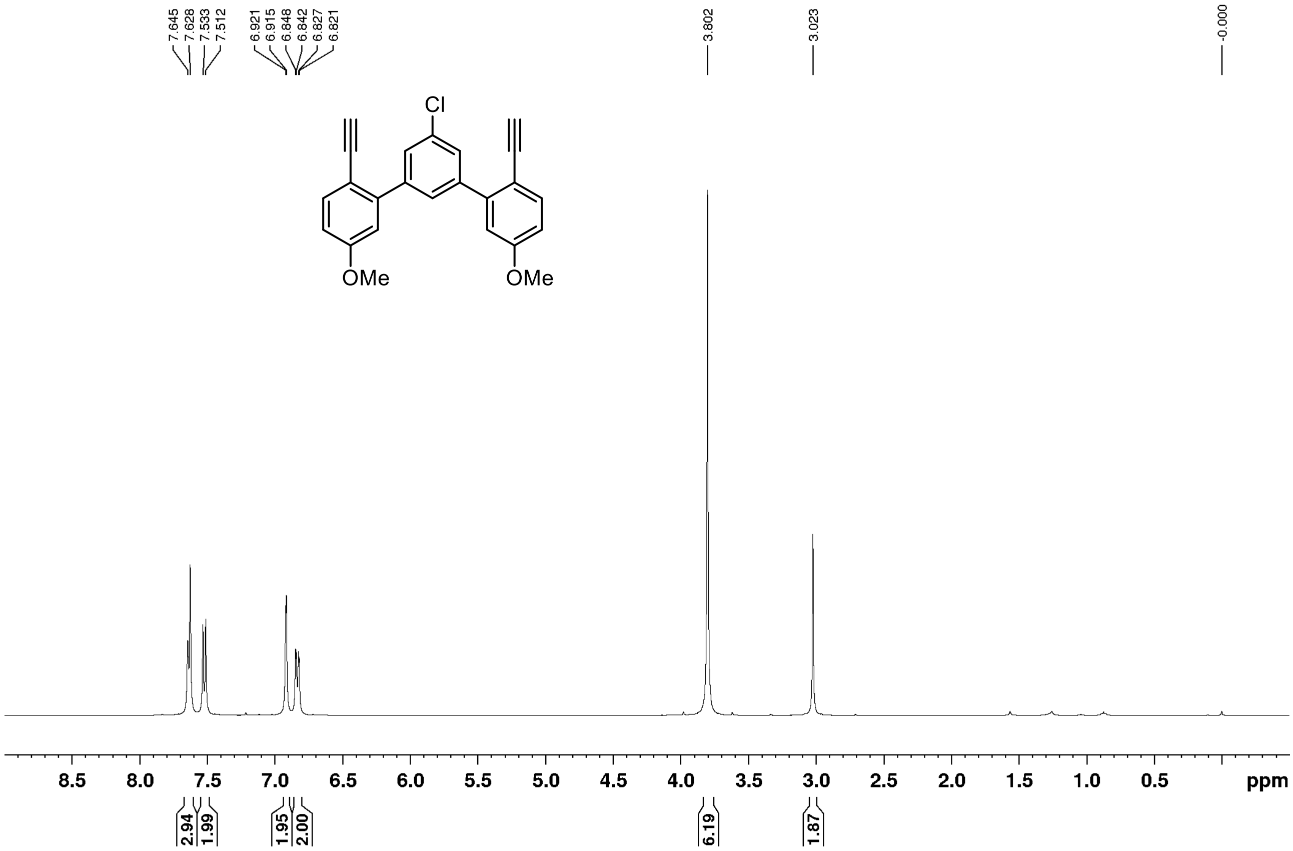

^{13}C NMR ($CDCl_3$, 100 MHz)

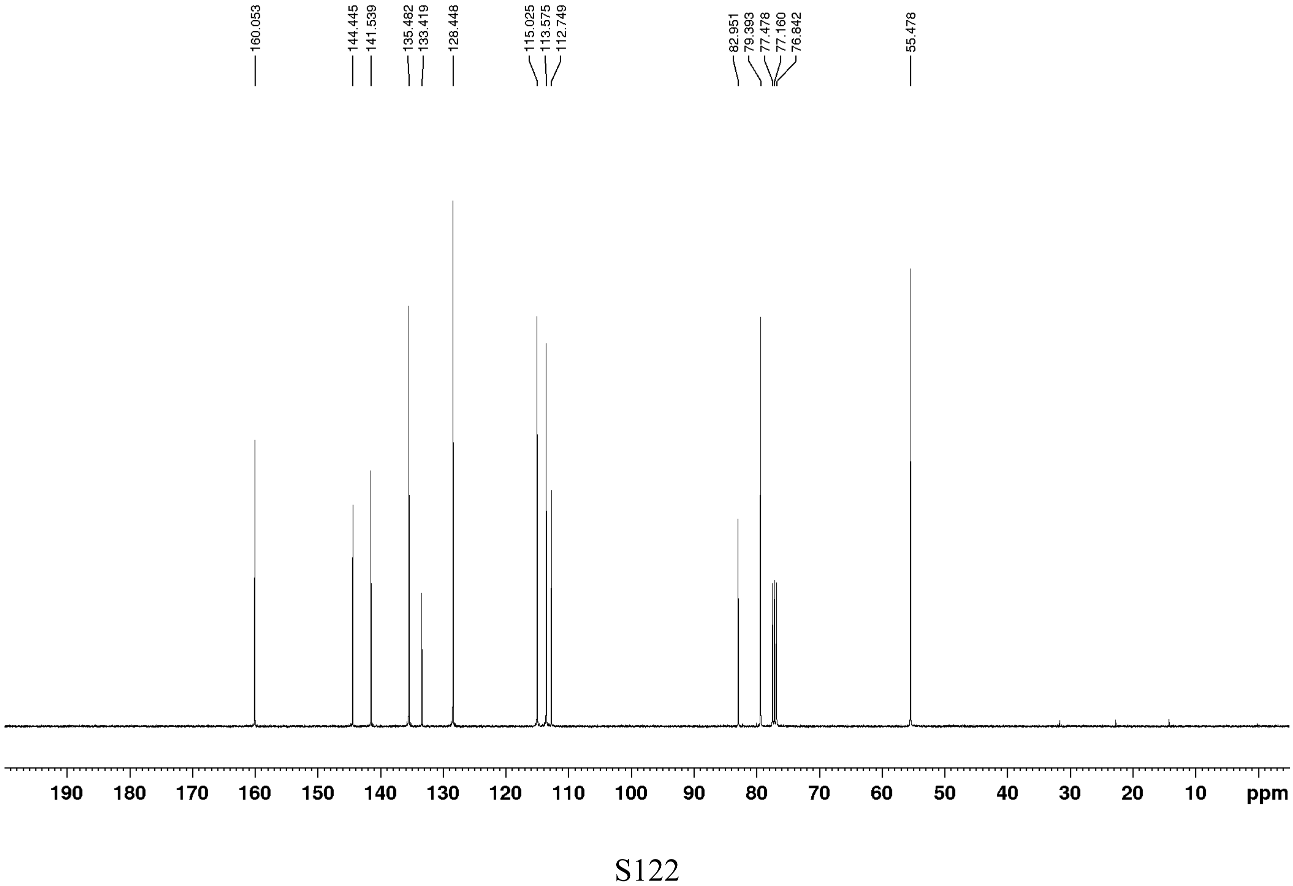

1,1'-(5-Chloro-1,3-phenylene)bis(2-ethynylnaphthalene) (1f)

^{1}H NMR ($CDCl_3$, 400 MHz)

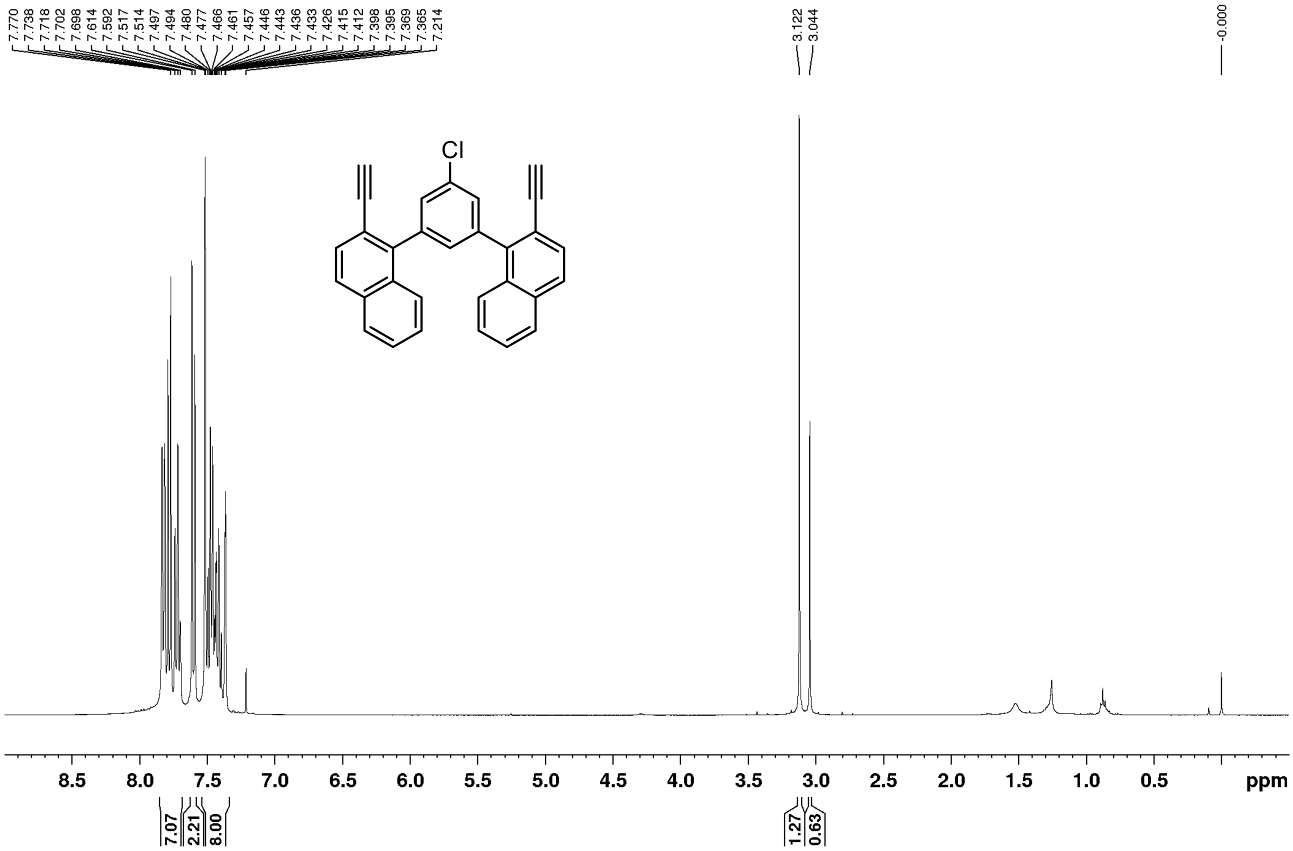

^{13}C NMR ($CDCl_3$, 100 MHz)

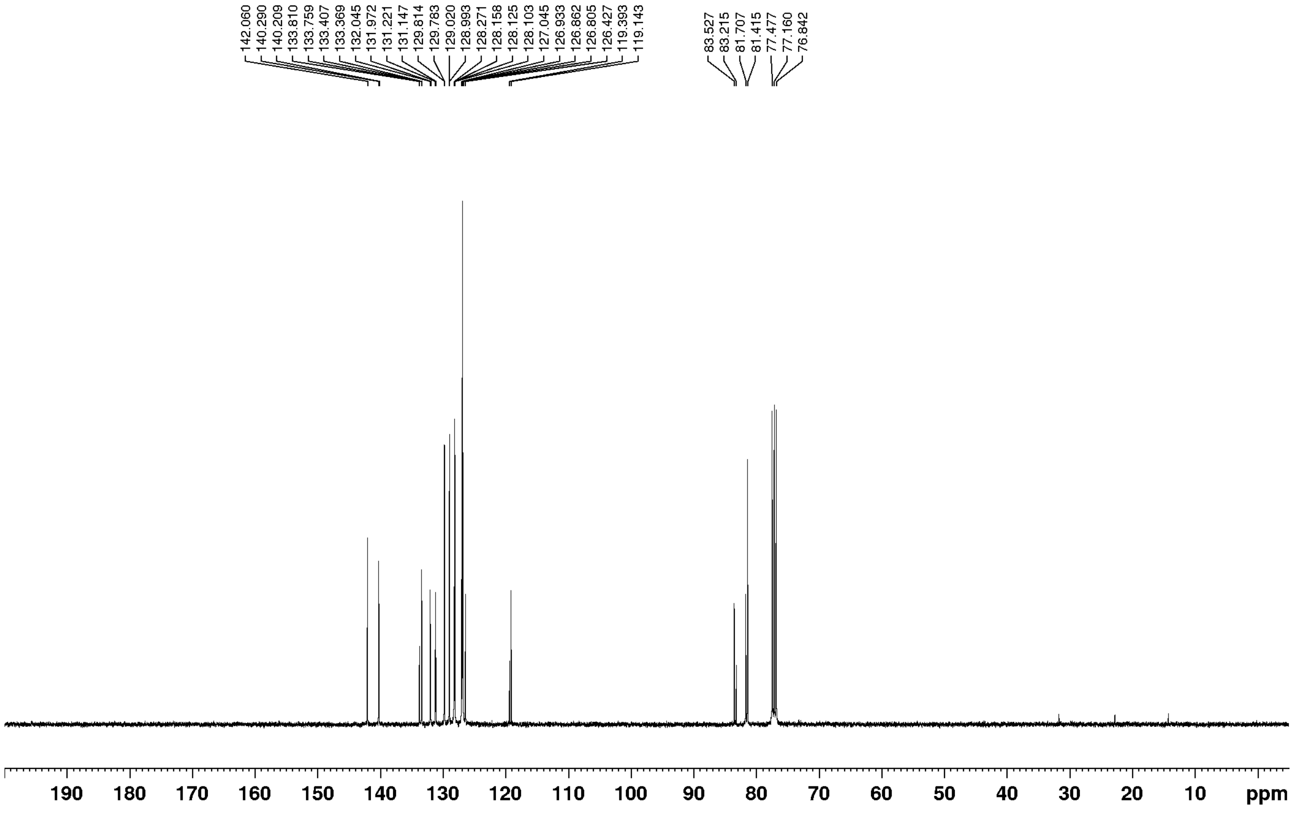

((3'-Bromo-5-(tert-butyl)-5'-chloro-[1,1'-biphenyl]-2-yl)ethynyl)triisopropylsilane (S14)

^{1}H NMR ($CDCl_3$, 400 MHz)

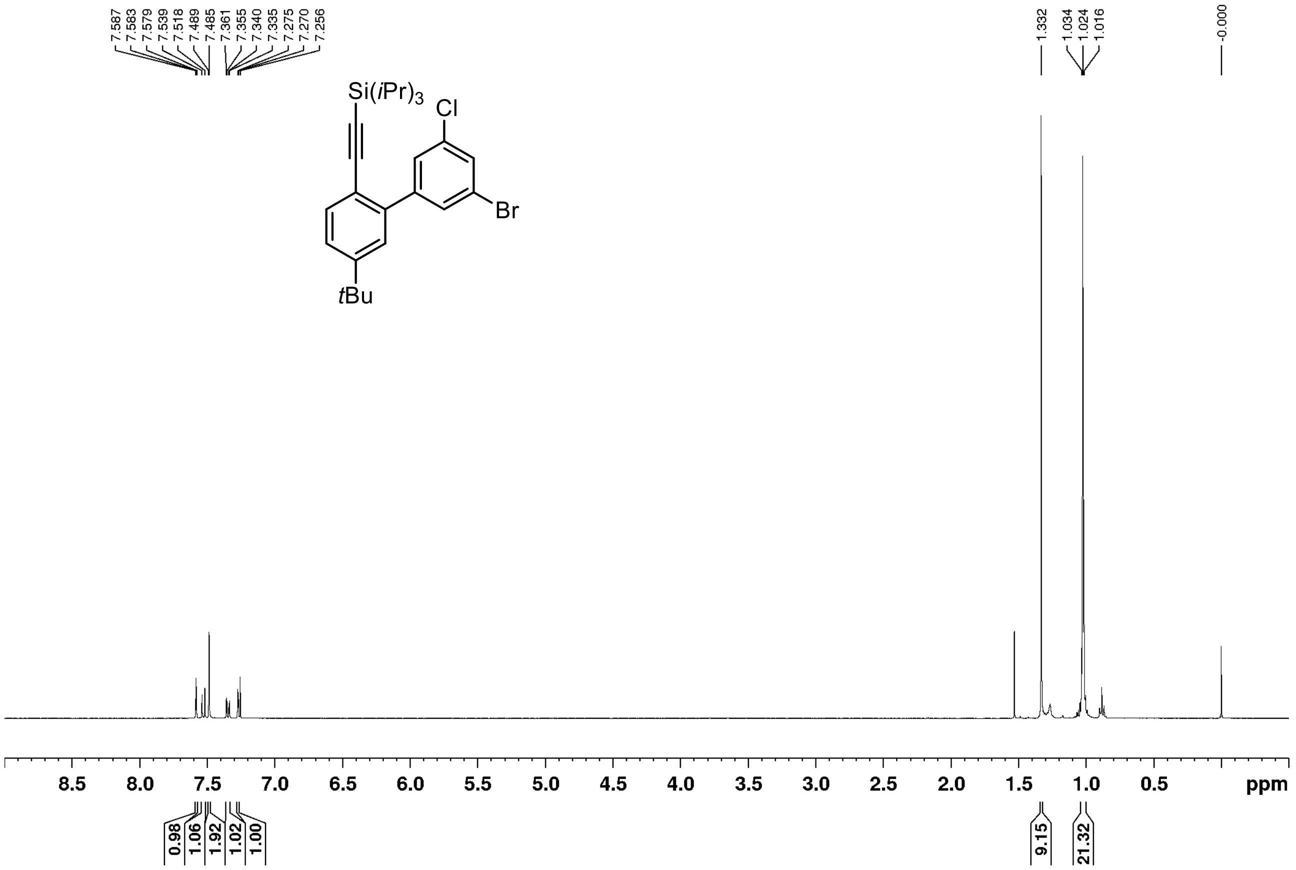

^{13}C NMR ($CDCl_3$, 100 MHz)

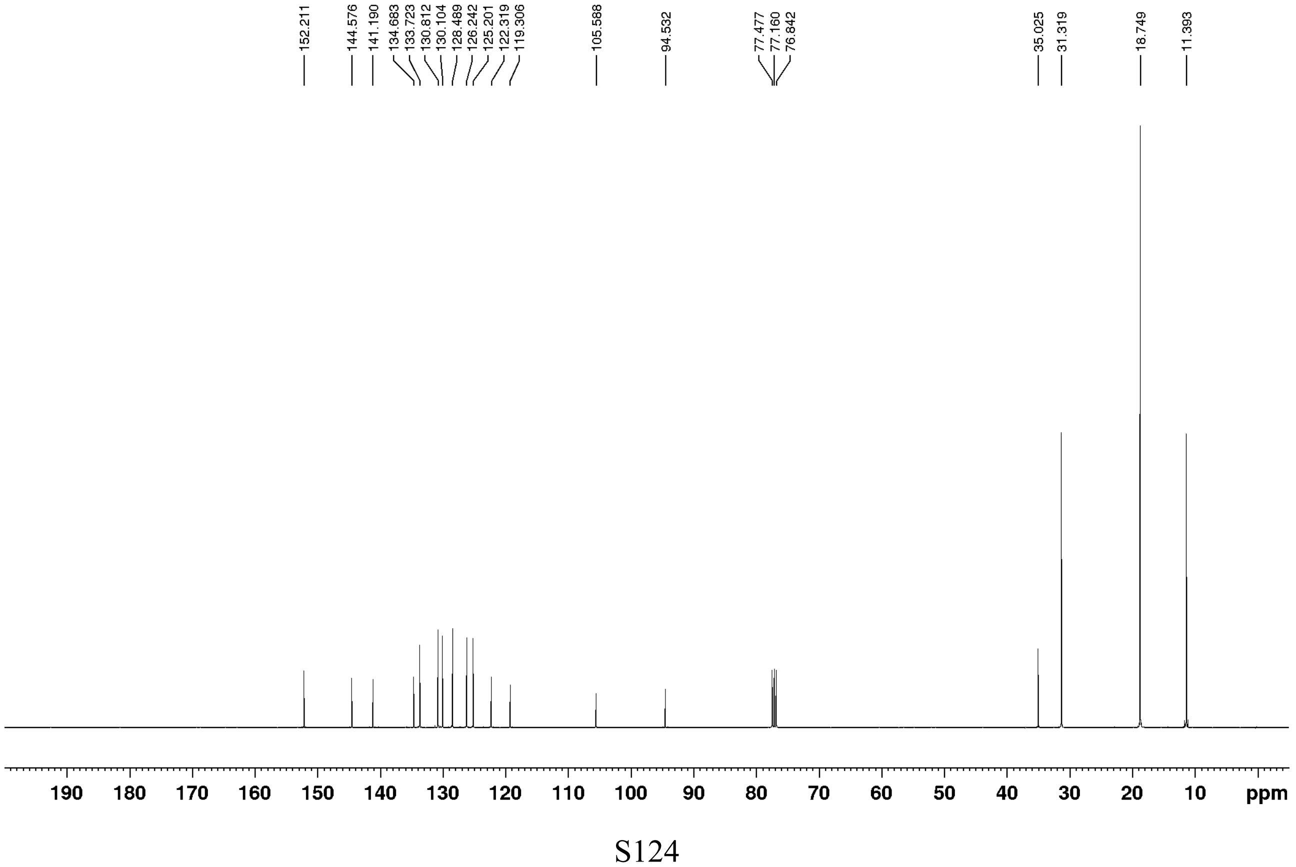

((5-(*tert*-Butyl)-3'-chloro-5'-(4,4,5,5-tetramethyl-1,3,2-dioxaborolan-2-yl)-[1,1'-biphenyl]-2-yl)ethynyl)triisopropylsilane (S15)

^{1}H NMR ($CDCl_3$, 400 MHz)

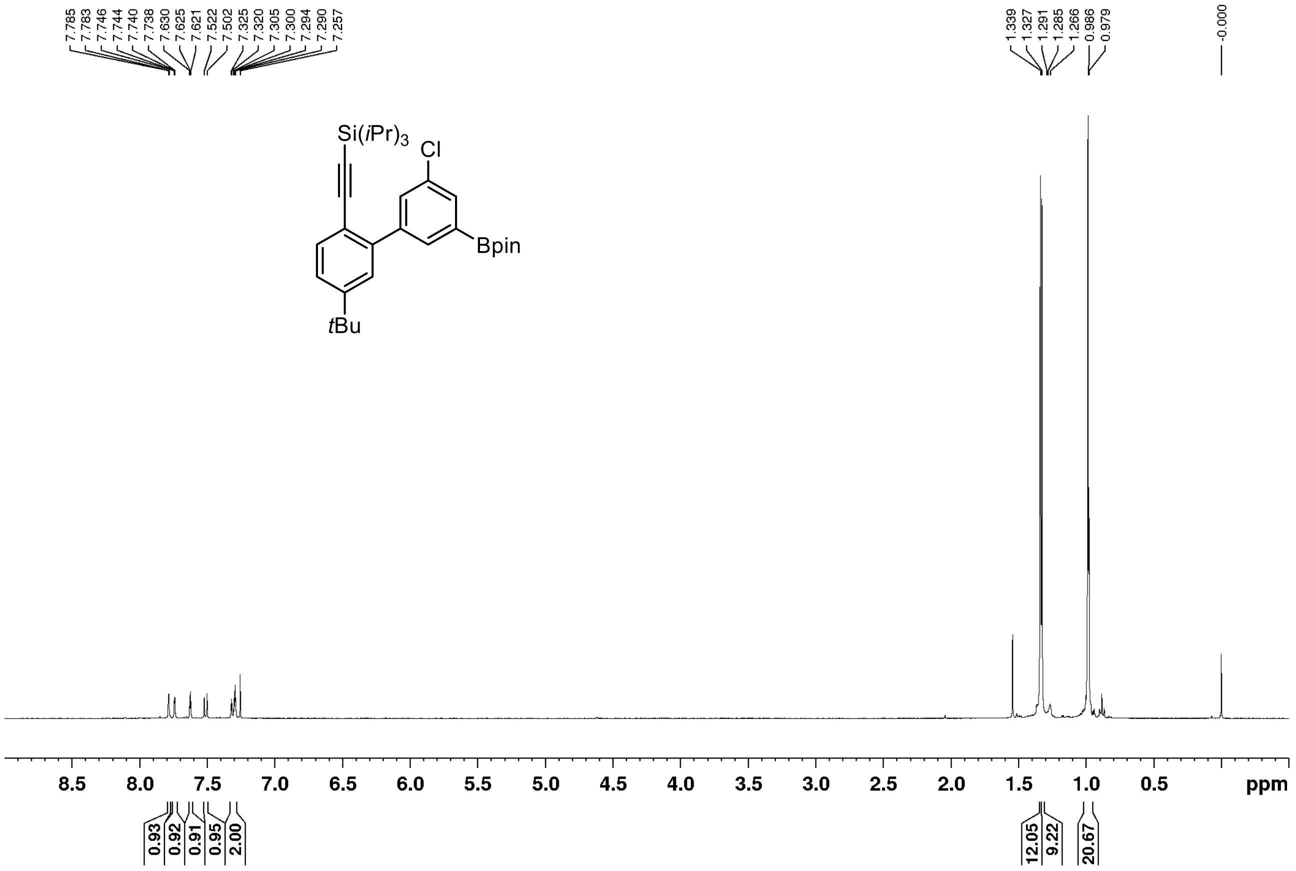

^{13}C NMR ($CDCl_3$, 100 MHz)

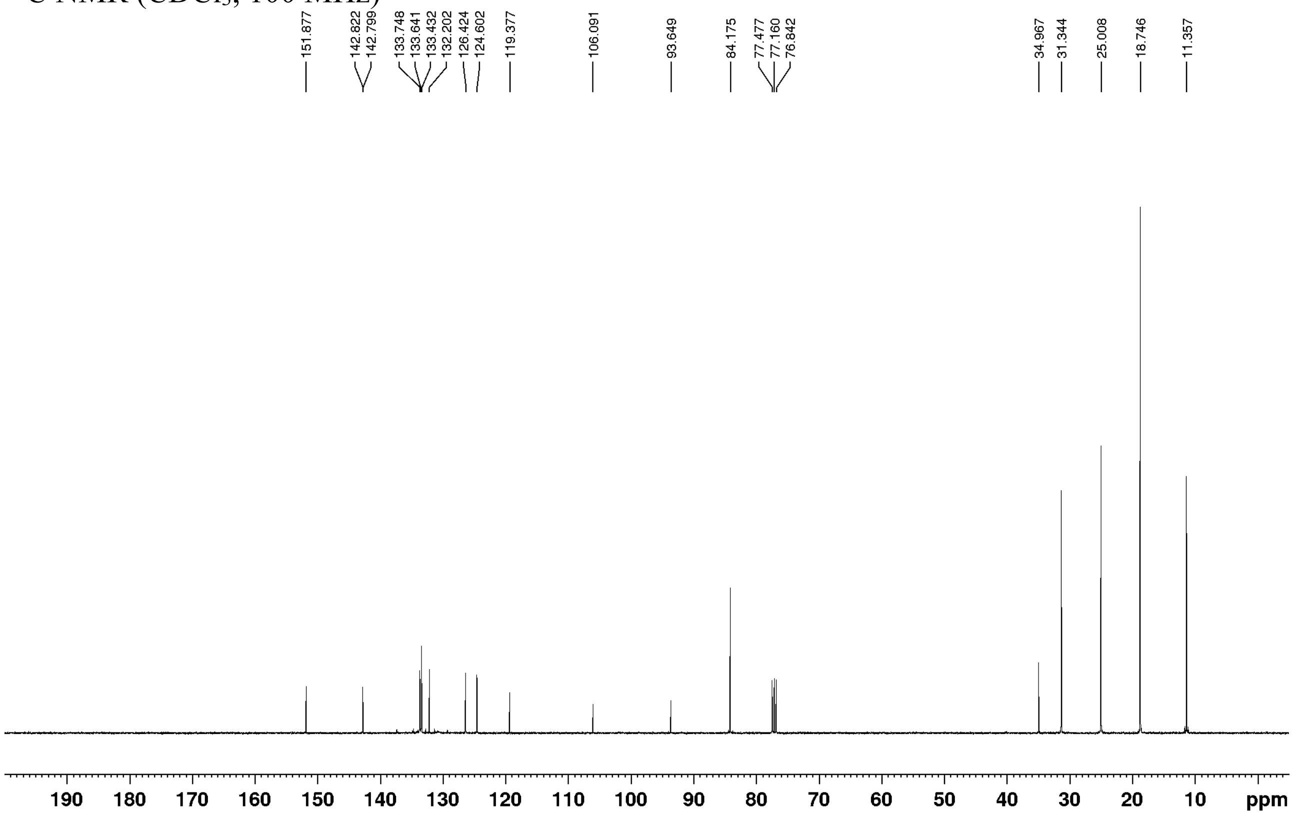

5,5''''-Di-*tert*-butyl-5',5'''-dichloro-2,2'',2'''',5''-tetraethynyl-1,1':3',1'':4'',1''':3''',1''''-quinquephenyl (7)

^{1}H NMR ($CDCl_3$, 400 MHz)

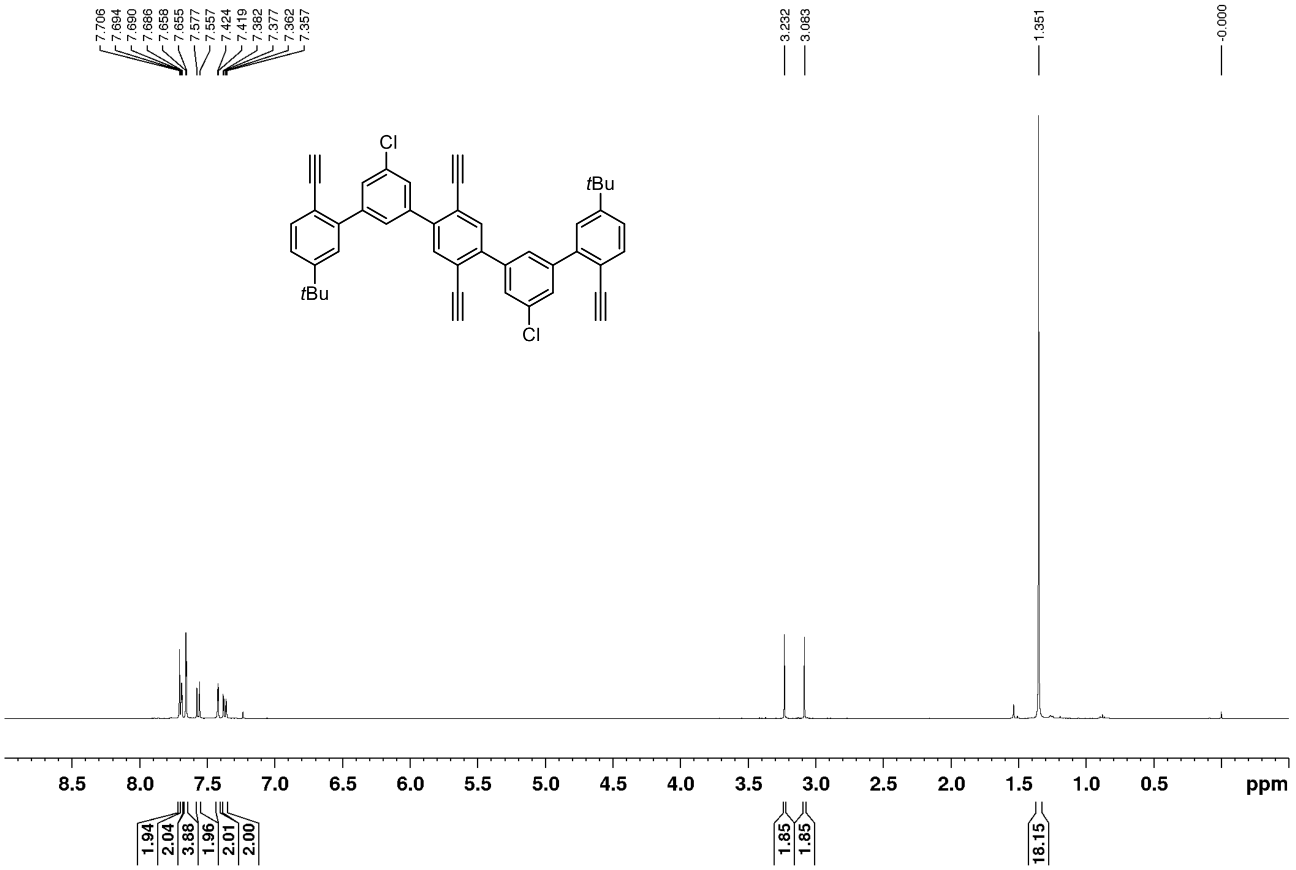

^{13}C NMR ($CDCl_3$, 100 MHz)

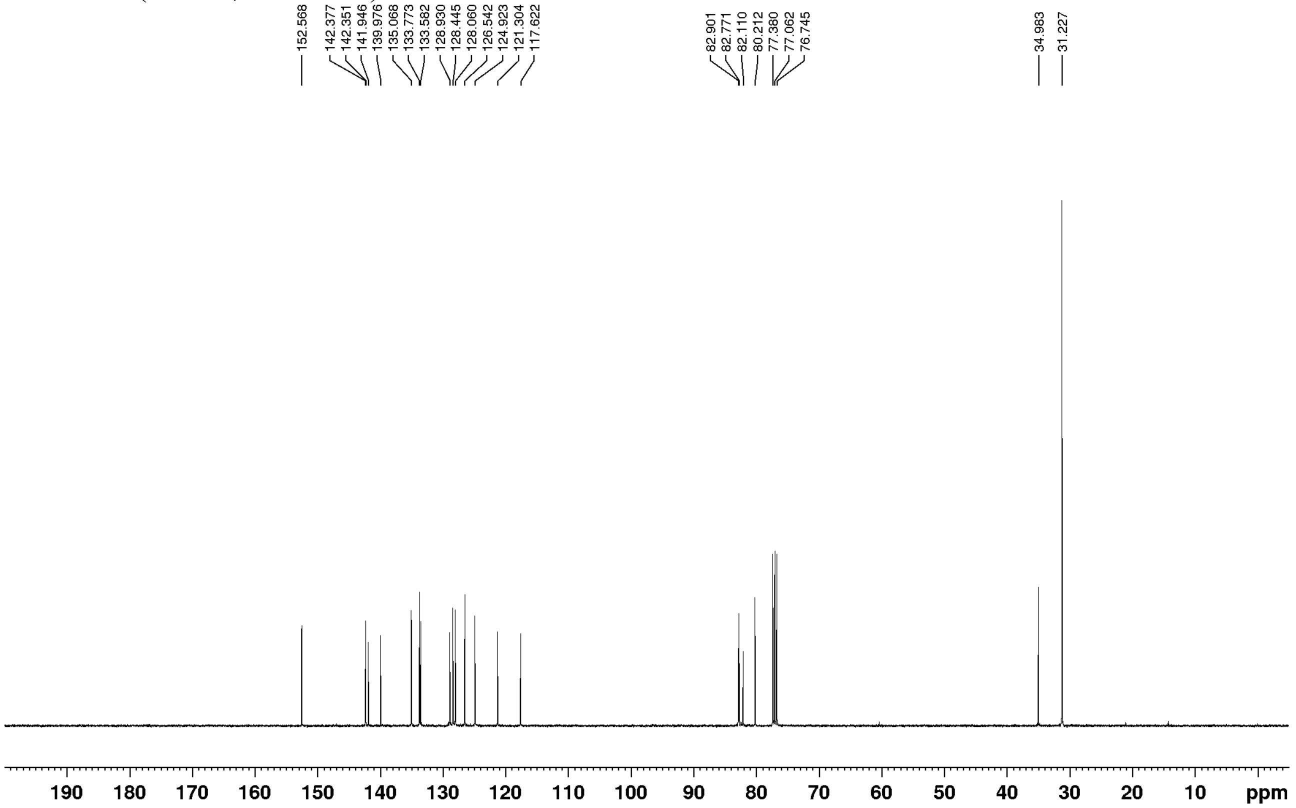

5'-Chloro-2-ethynyl-2''-(*p*-tolylethynyl)-1,1':3',1''-terphenyl (S21)

^{1}H NMR ($CDCl_3$, 400 MHz)

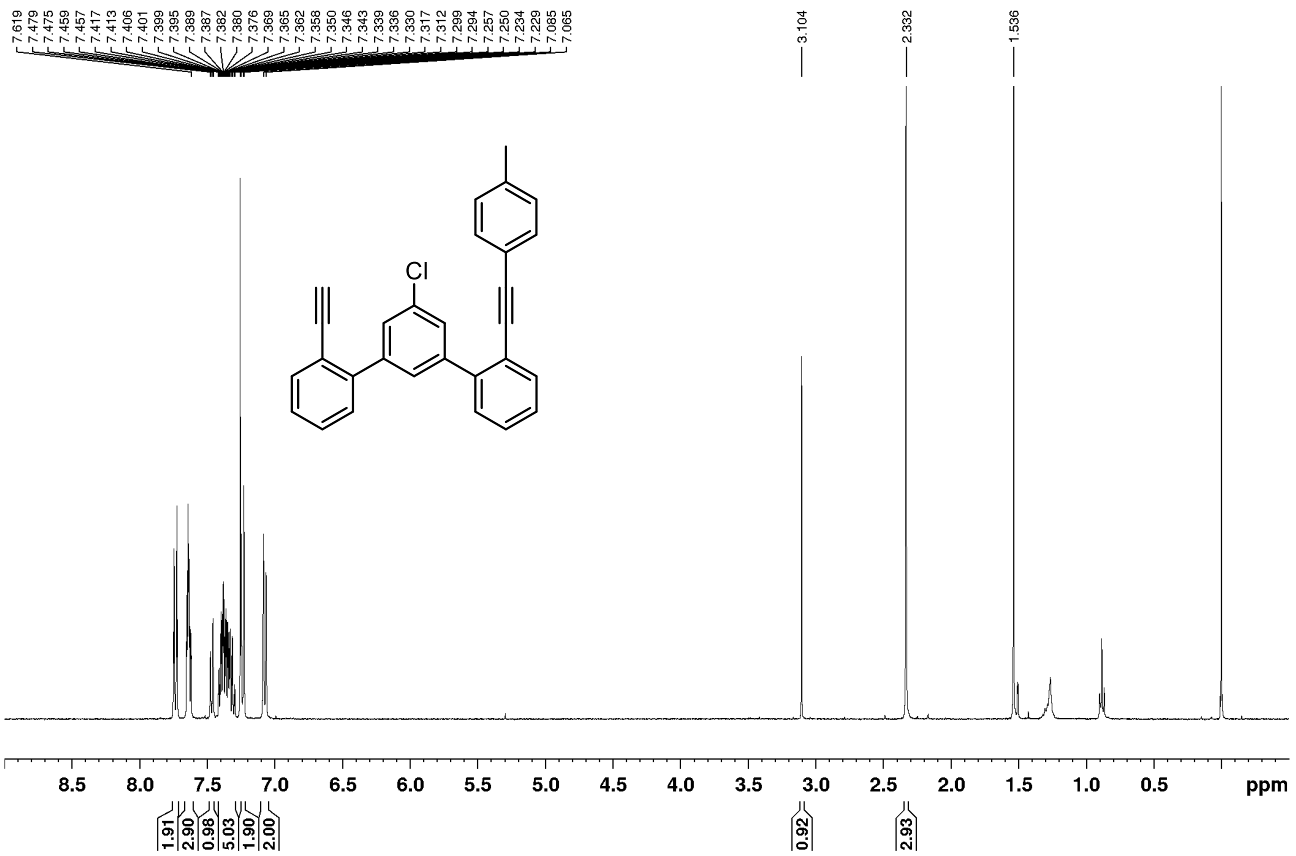

^{13}C NMR ($CDCl_3$, 100 MHz)

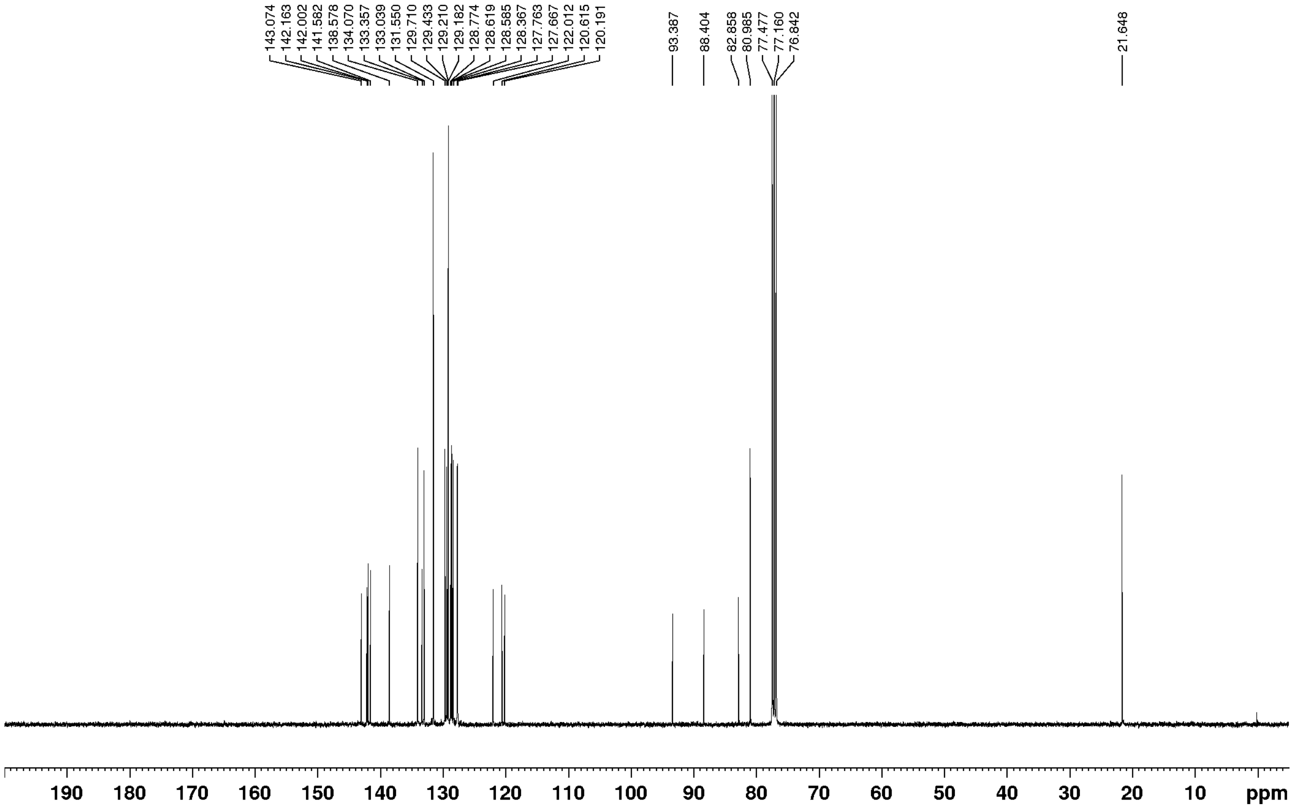

Dimethyl 2-methylbenzo[7,8]azuleno[6,5,4-jk]fluorene-9,10-dicarboxylate (6aa)

^{1}H NMR ($CDCl_3$, 400 MHz)

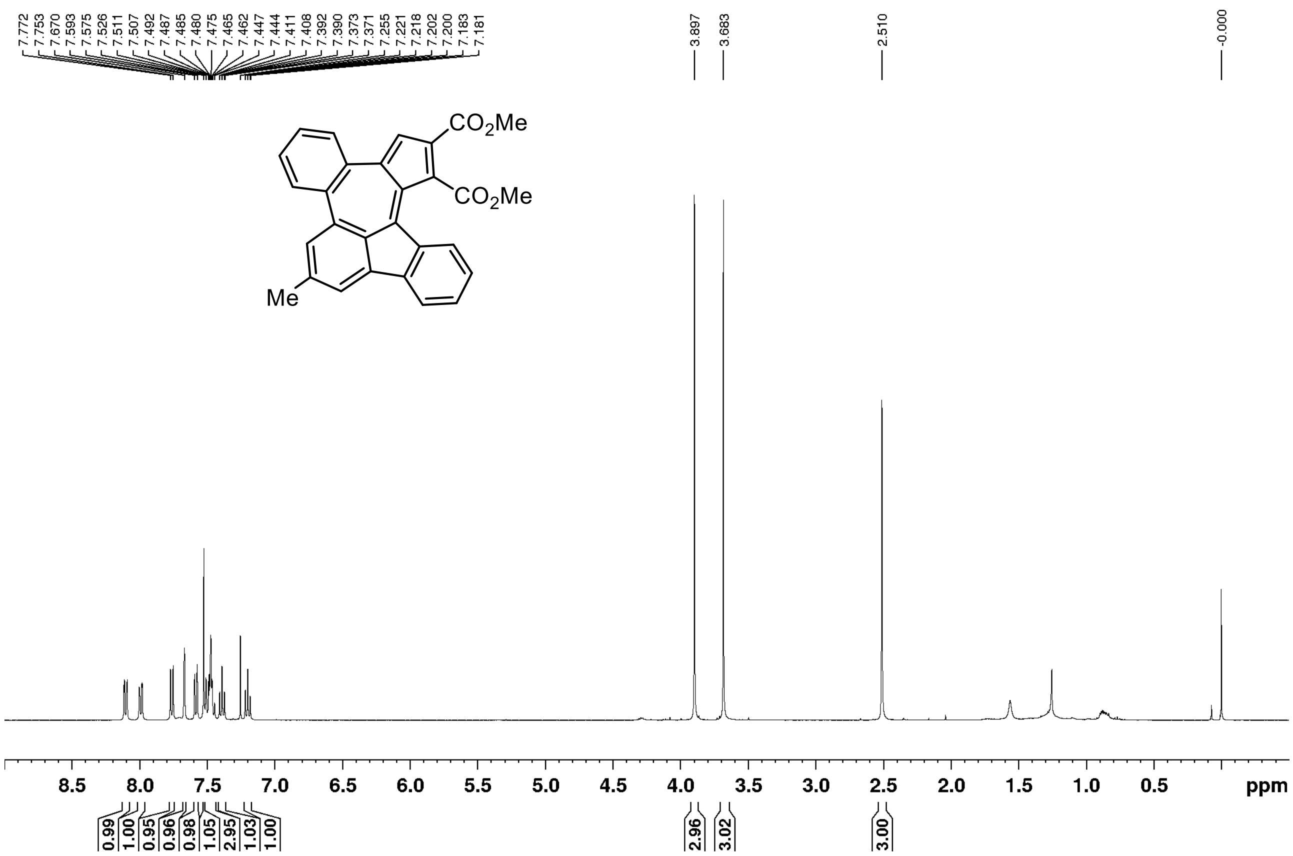

^{13}C NMR ($CDCl_3$, 100 MHz)

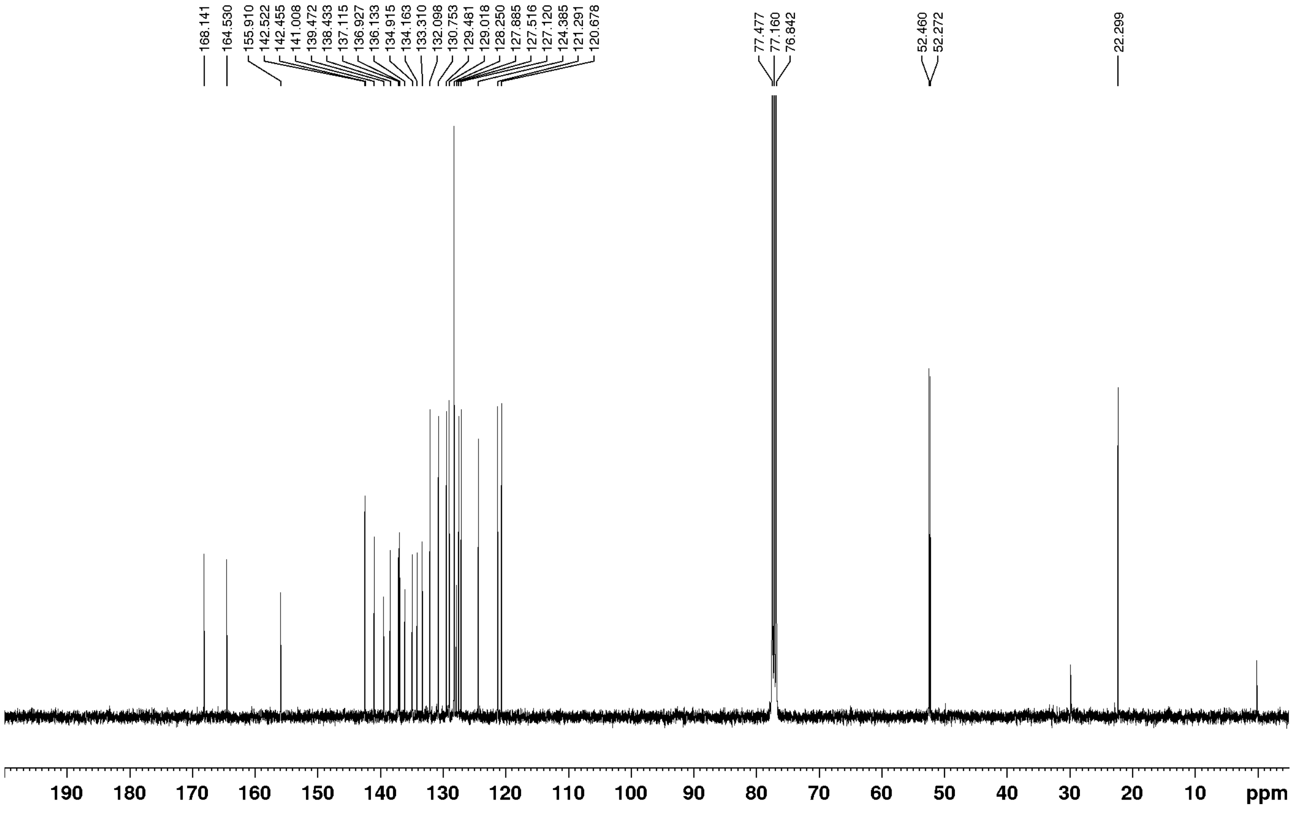

Diethyl 2-methylbenzo[7,8]azuleno[6,5,4-jk]fluorene-9,10-dicarboxylate (6ab)

^{1}H NMR ($CDCl_3$, 400 MHz)

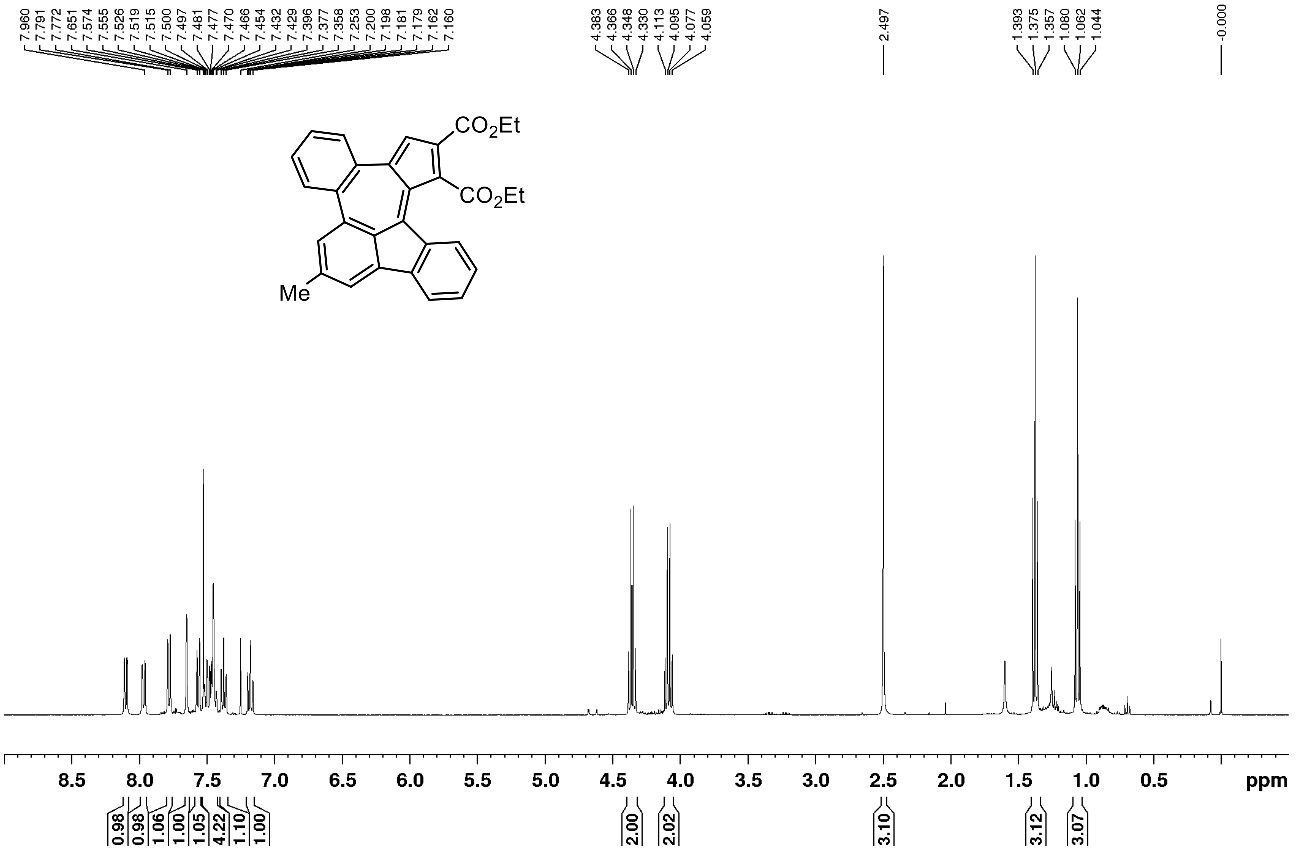

^{13}C NMR ($CDCl_3$, 100 MHz)

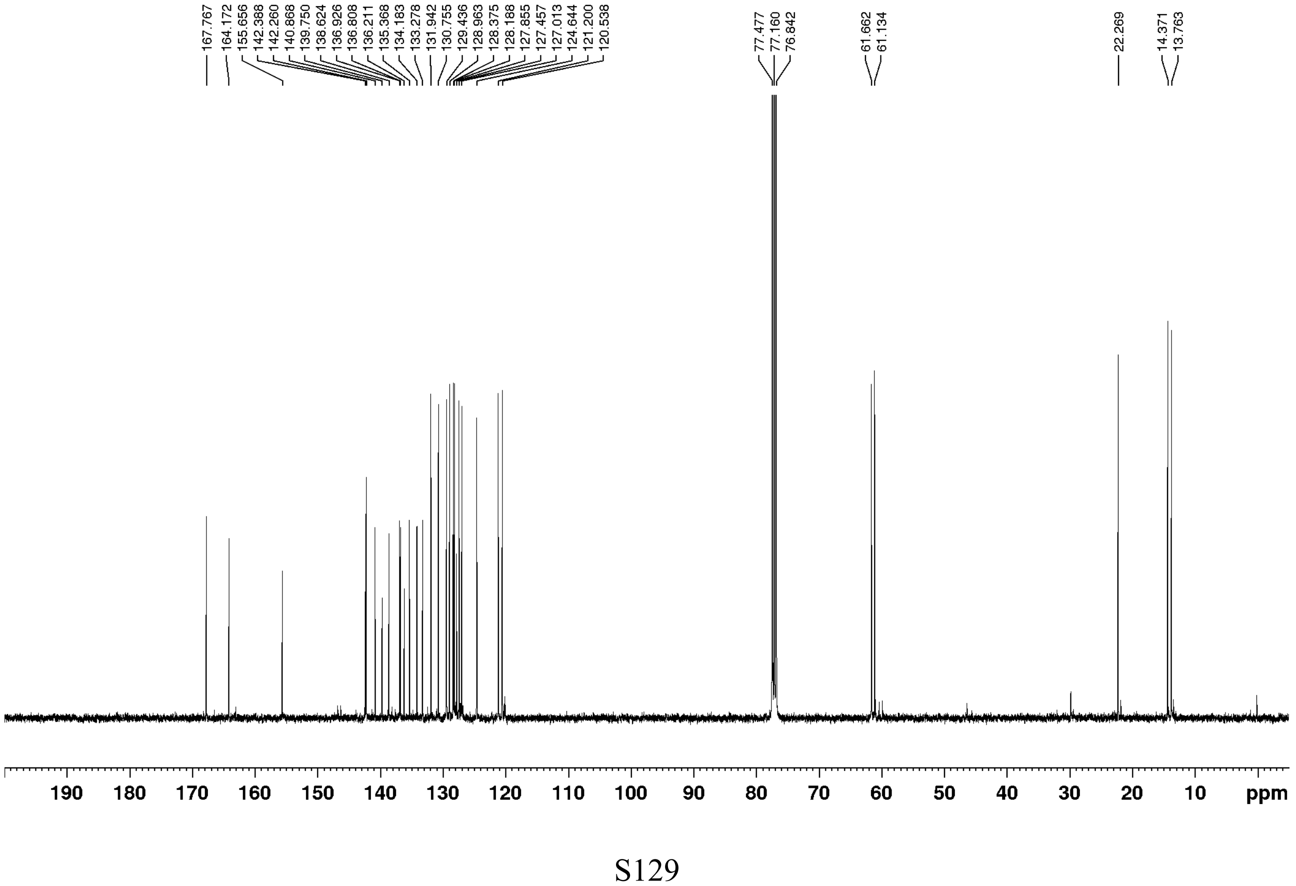

Di-tert-butyl 2-methylbenzo[7,8]azuleno[6,5,4-jk]fluorene-9,10-dicarboxylate (6ac)

^{1}H NMR (CDCl$_3$, 400 MHz)

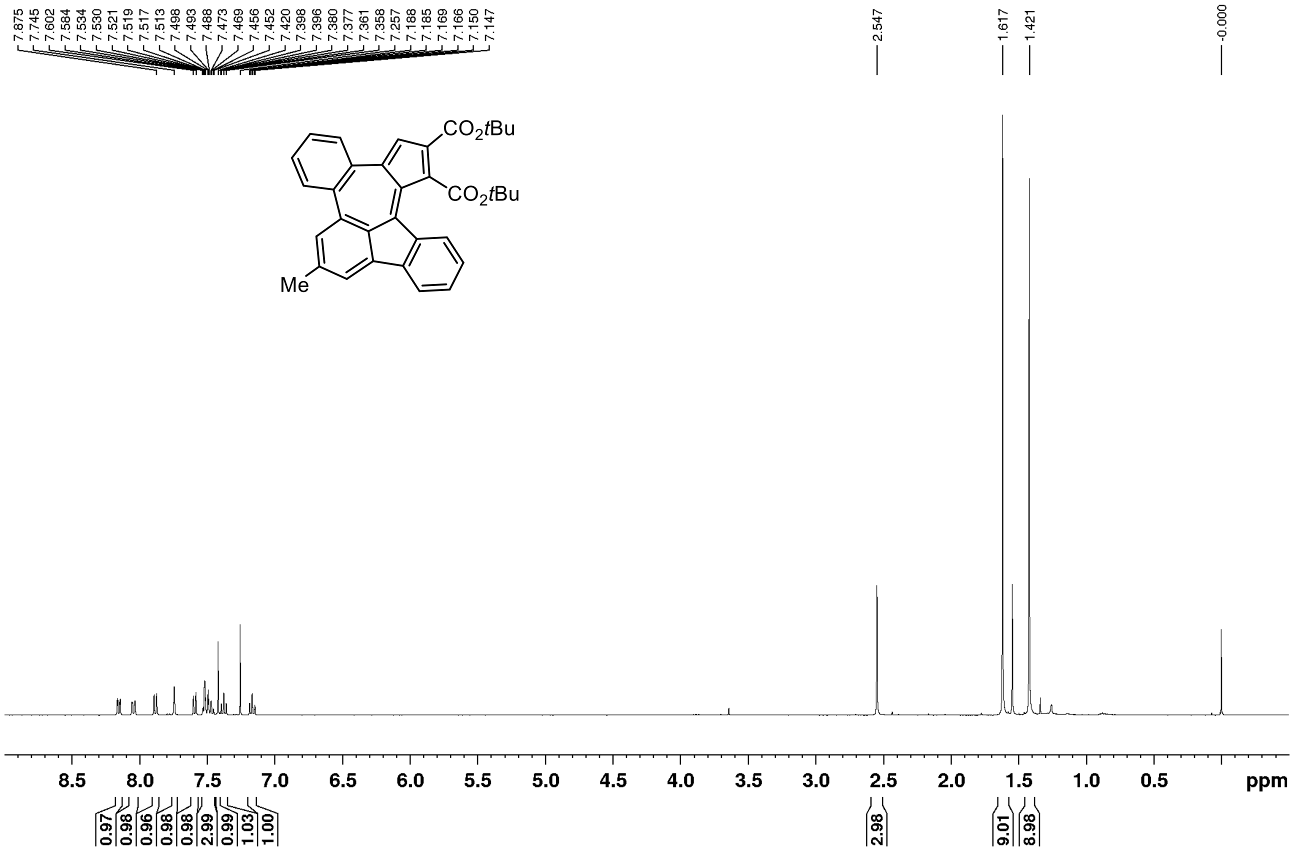

^{13}C NMR (CDCl$_3$, 100 MHz)

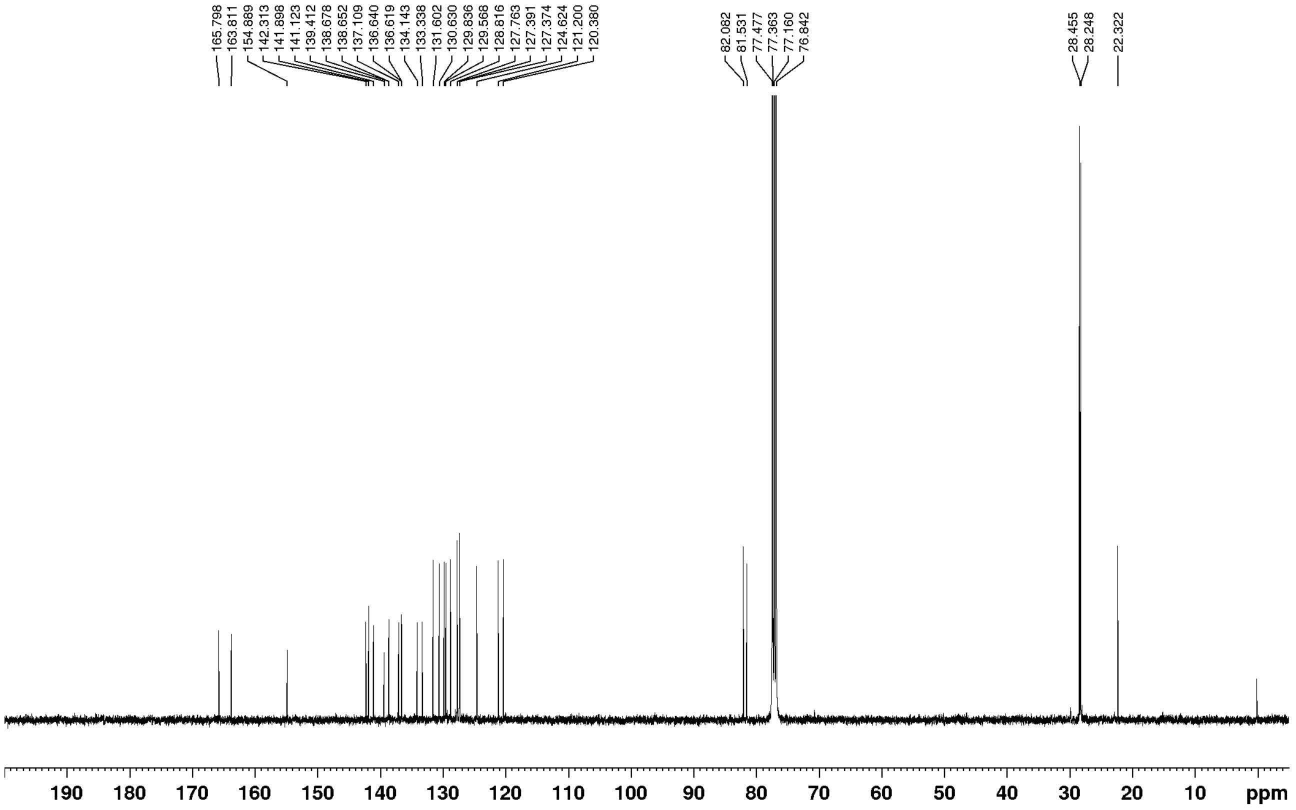

Dimethyl 2-chlorobenzo[7,8]azuleno[6,5,4-jk]fluorene-9,10-dicarboxylate (6ba)

^{1}H NMR ($CDCl_3$, 400 MHz)

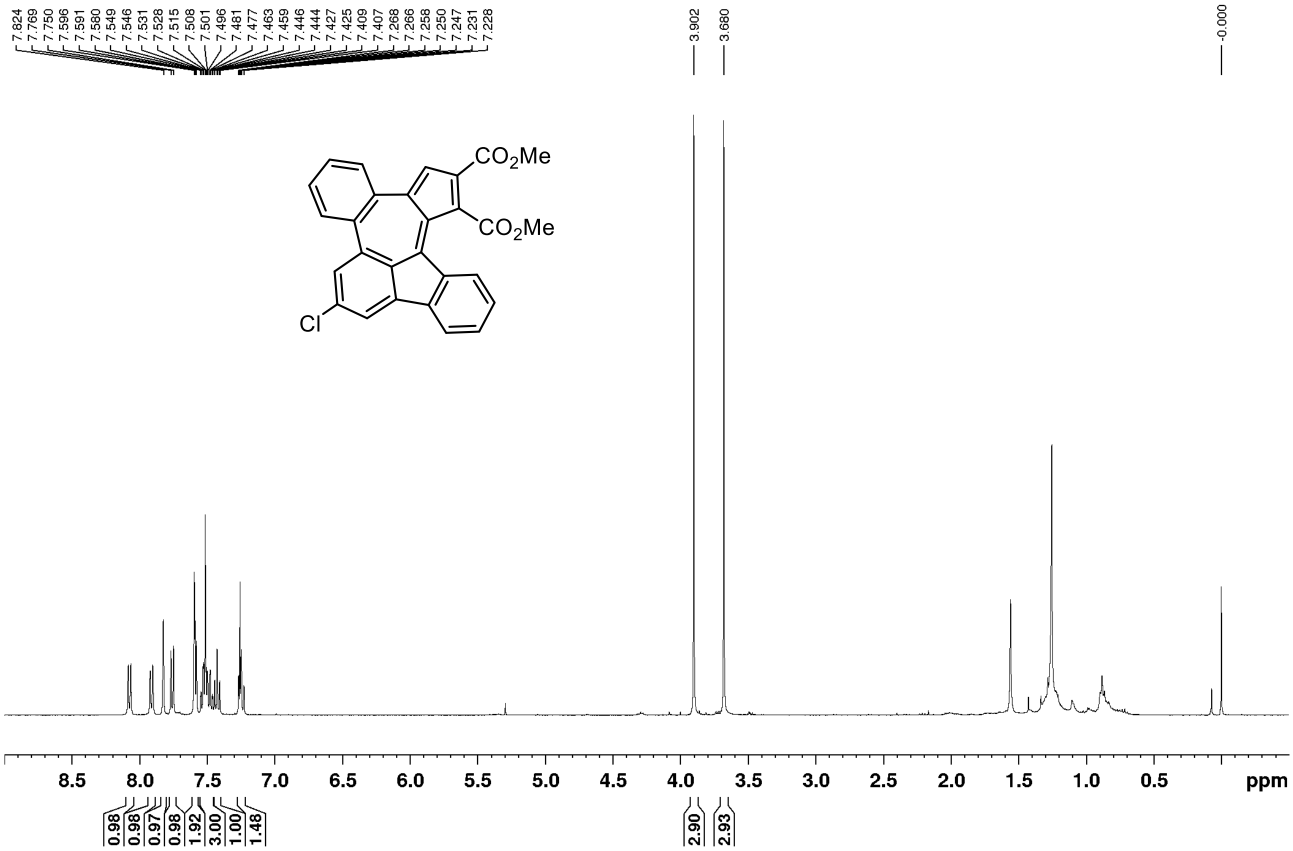

^{13}C NMR ($CDCl_3$, 100 MHz)

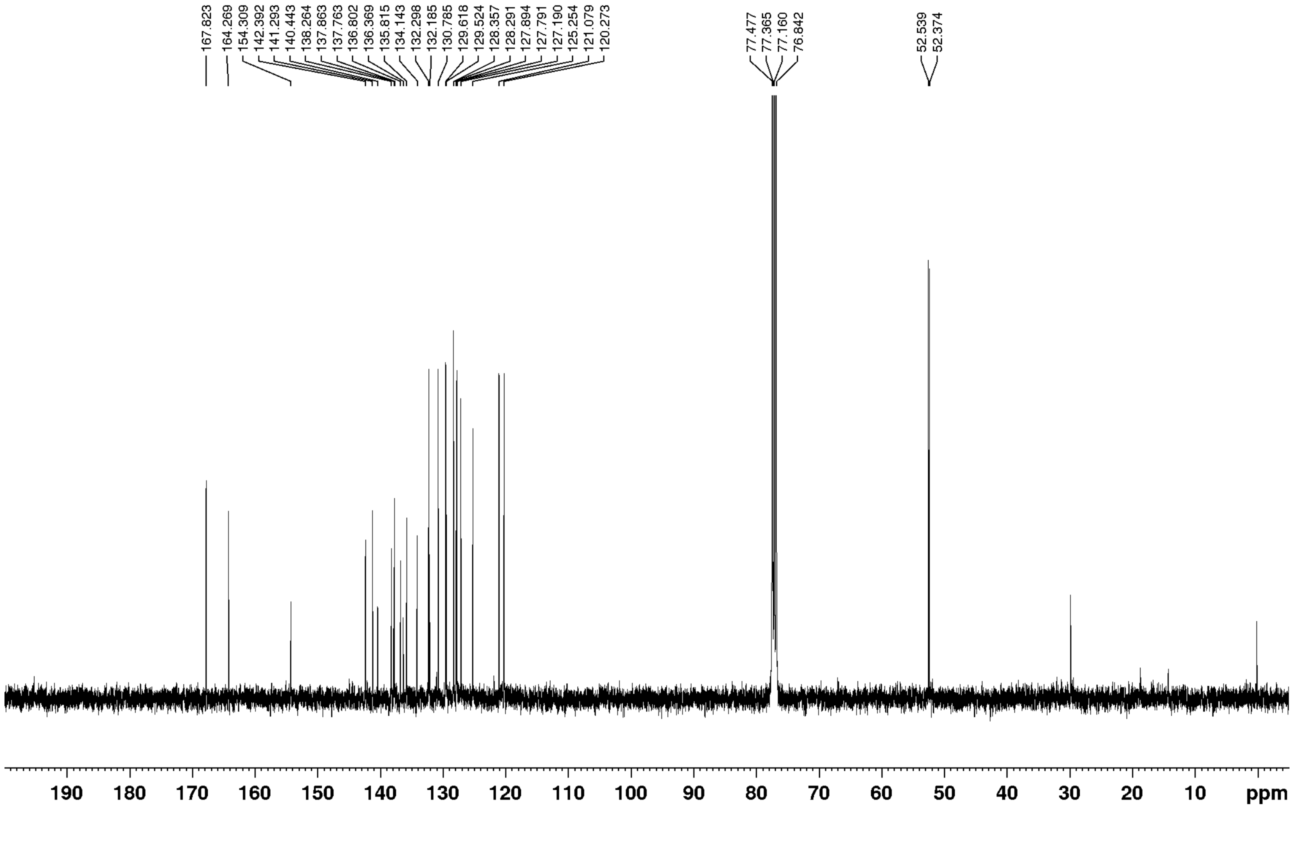

Dimethyl 2-(tert-butoxy)benzo[7,8]azuleno[6,5,4-jk]fluorene-9,10-dicarboxylate (6ca)

^{1}H NMR ($CDCl_3$, 400 MHz)

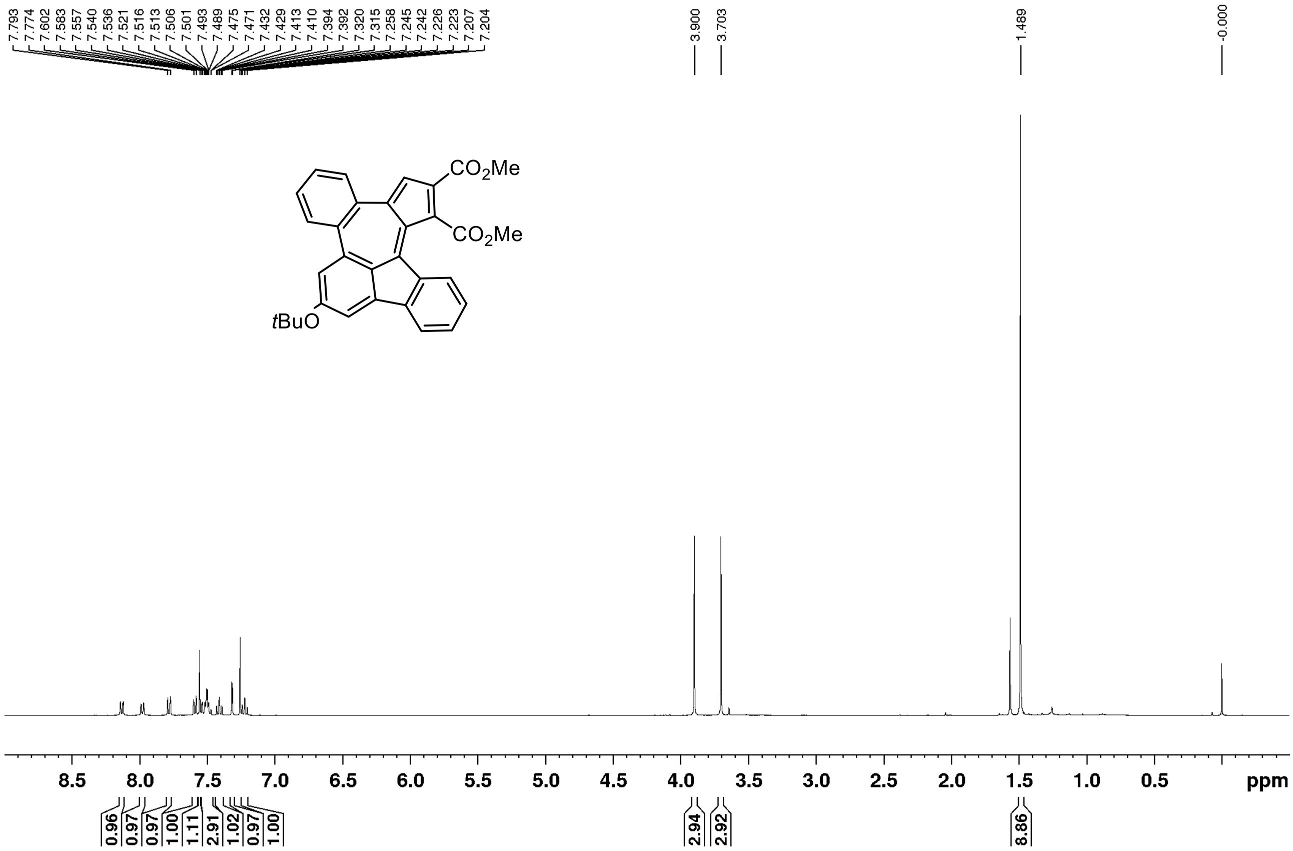

^{13}C NMR ($CDCl_3$, 100 MHz)

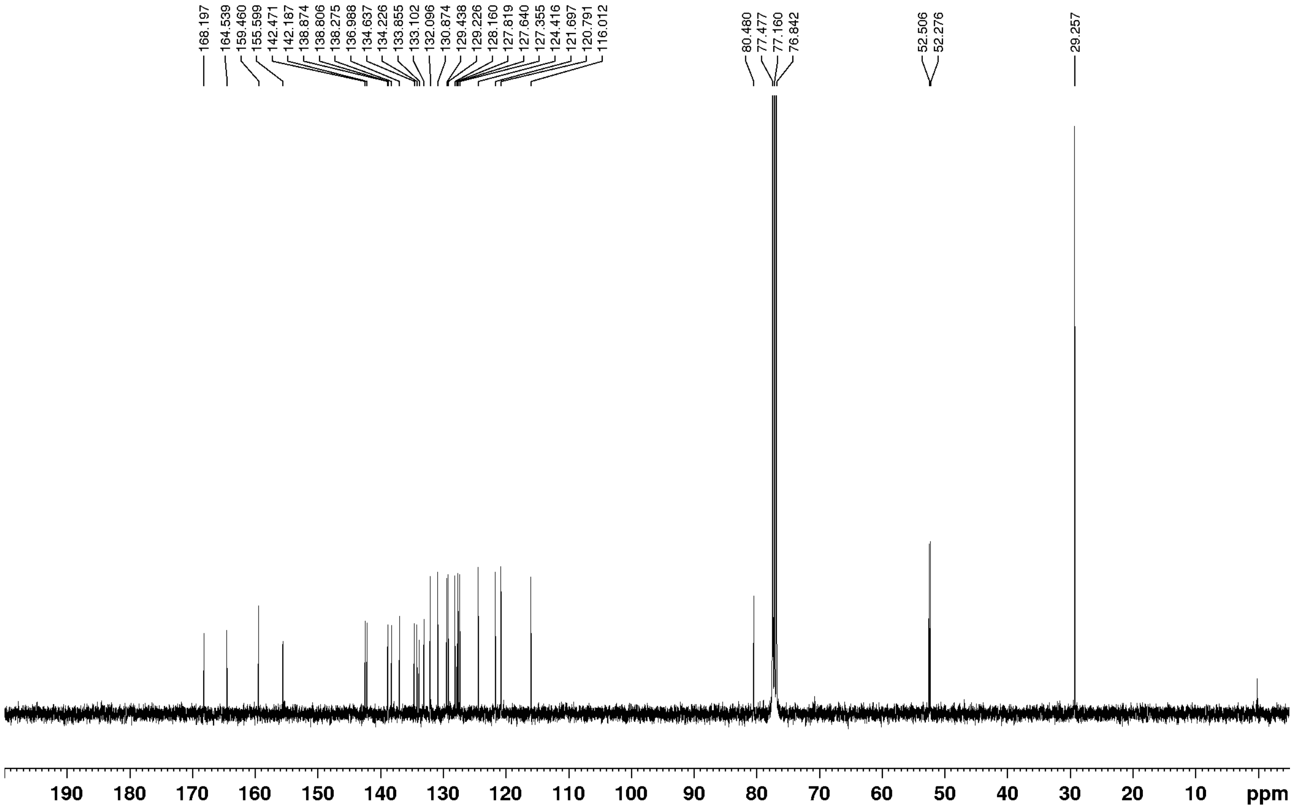

Dimethyl 5,13-di-tert-butyl-2-chlorobenzo[7,8]azuleno[6,5,4-jk]fluorene-9,10-dicarboxylate (6da)

^{1}H NMR ($CDCl_3$, 400 MHz)

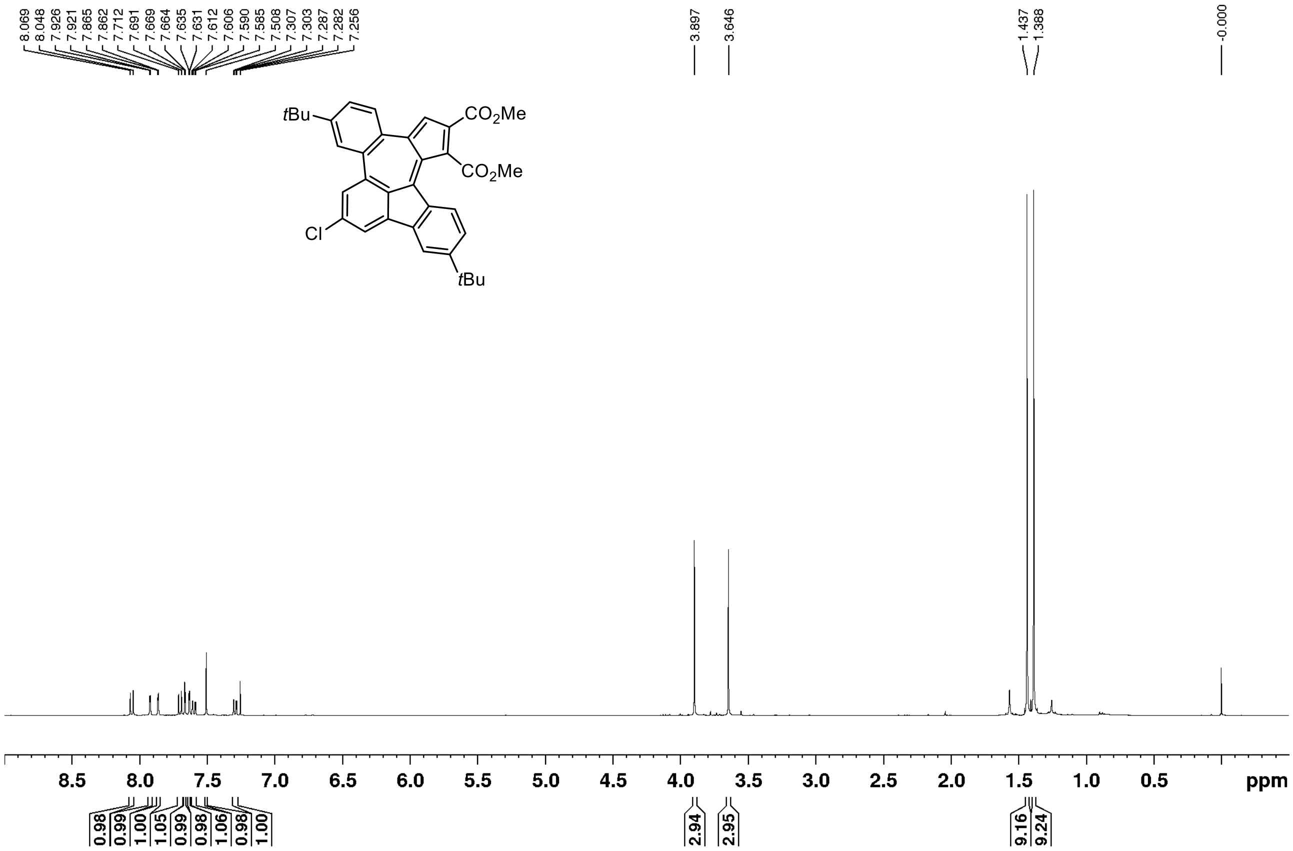

^{13}C NMR ($CDCl_3$, 100 MHz)

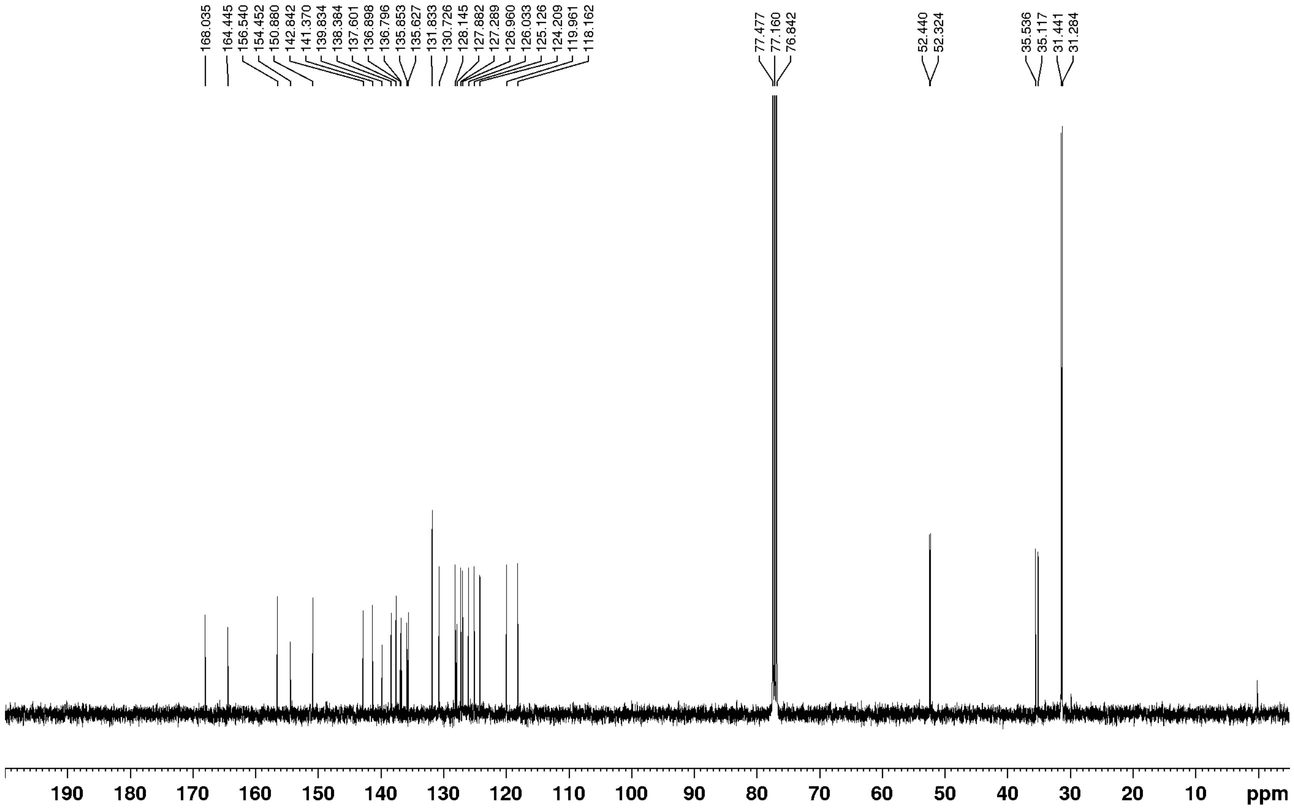

Dimethyl 2-chloro-5,13-dimethoxybenzo[7,8]azuleno[6,5,4-jk]fluorene-9,10-dicarboxylate (6ea)

^{1}H NMR ($CDCl_3$, 400 MHz)

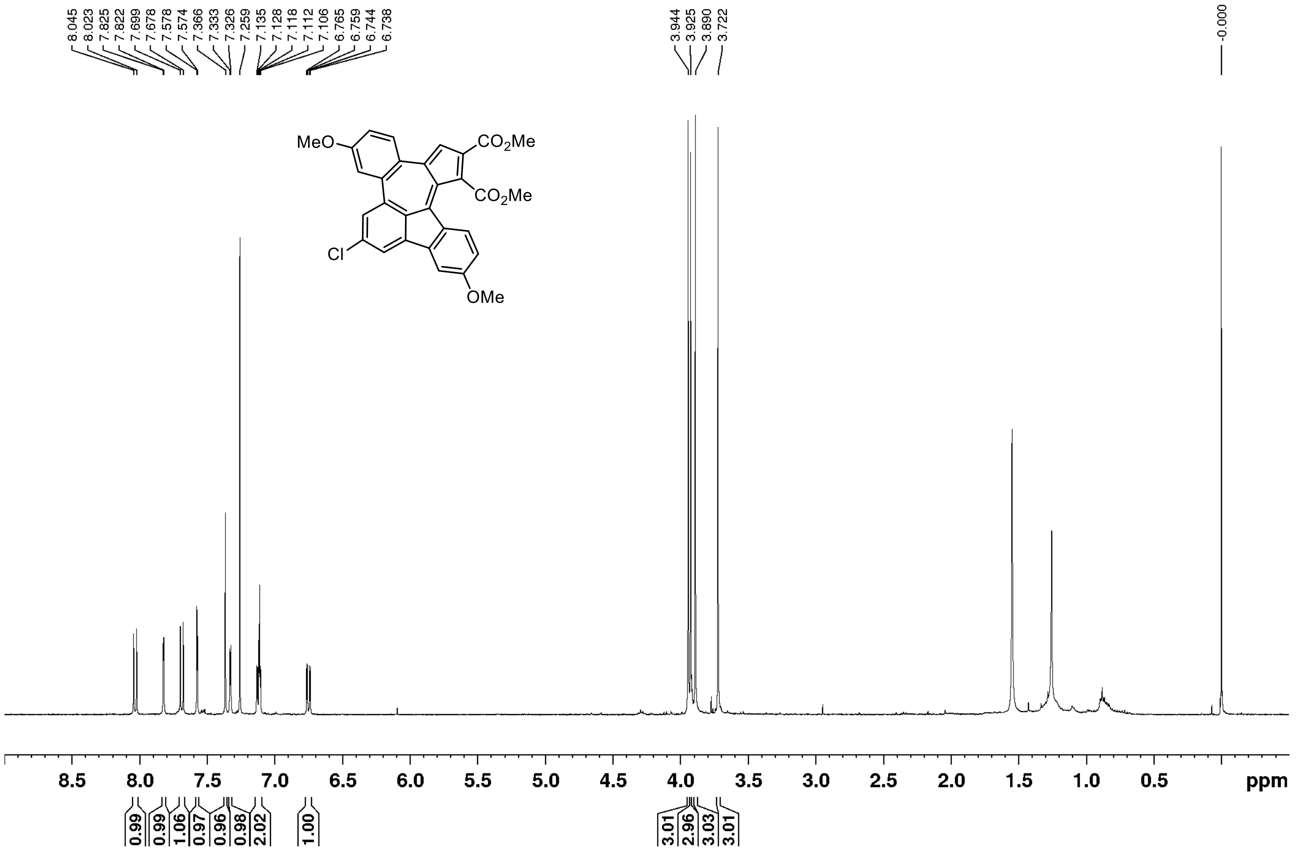

^{13}C NMR ($CDCl_3$, 100 MHz)

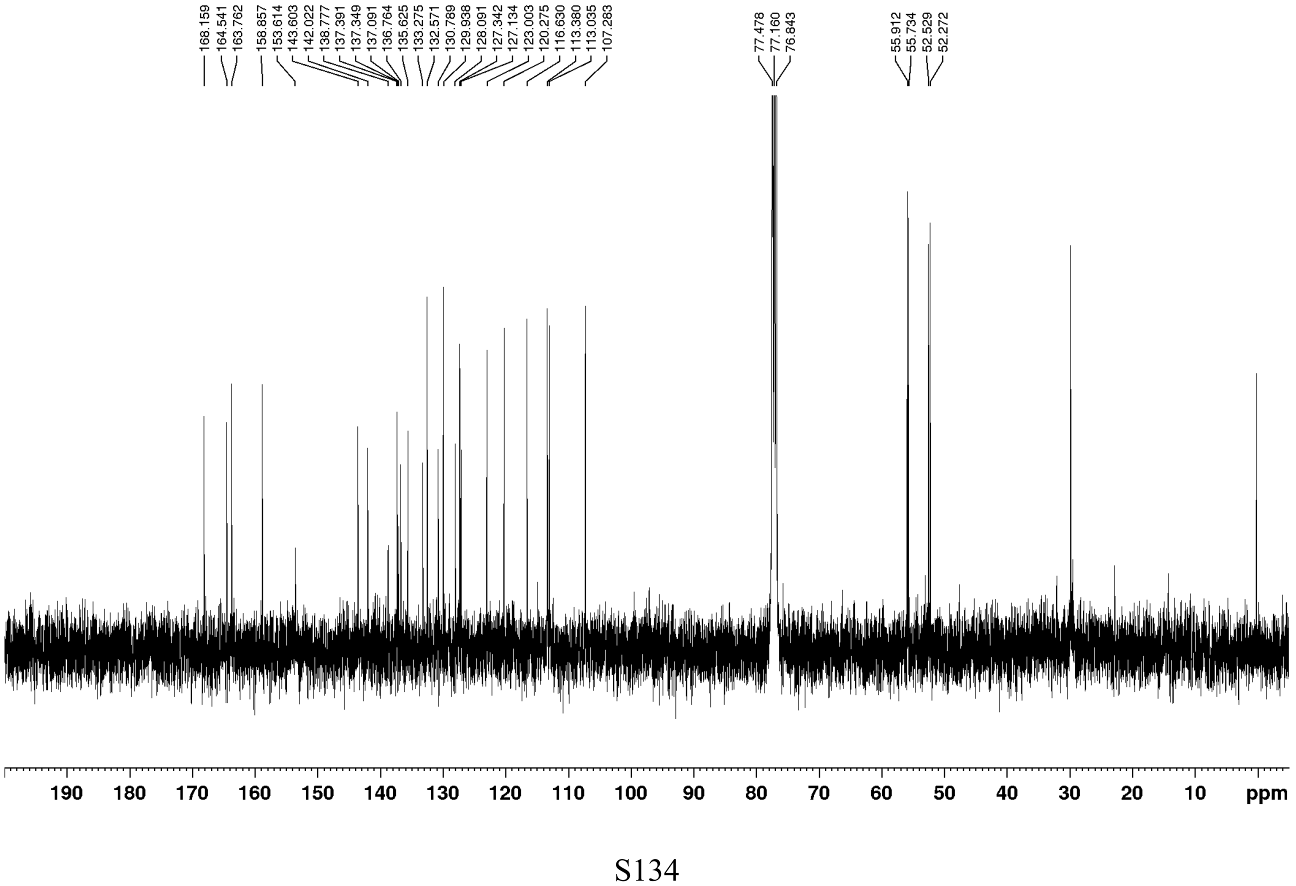

Dimethyl 17-chlorobenzo[c]naphtho[1',2':7,8]azuleno[6,5,4-jk]fluorene-8,9-dicarboxylate (6fa)

^{1}H NMR ($CDCl_3$, 400 MHz)

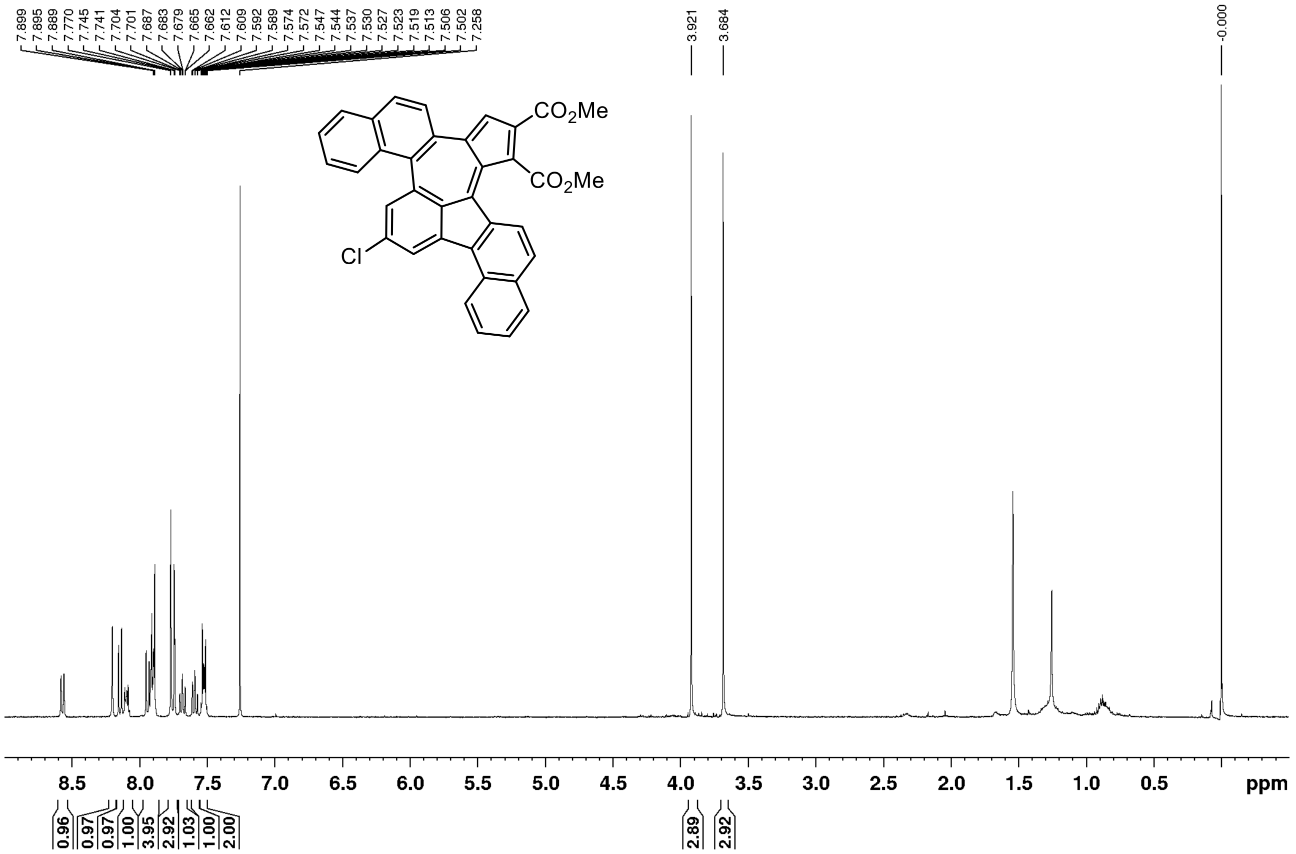

^{13}C NMR ($CDCl_3$, 100 MHz)

Note: This compound has poor solubility and exhibited low ^{13}C NMR peak intensities.

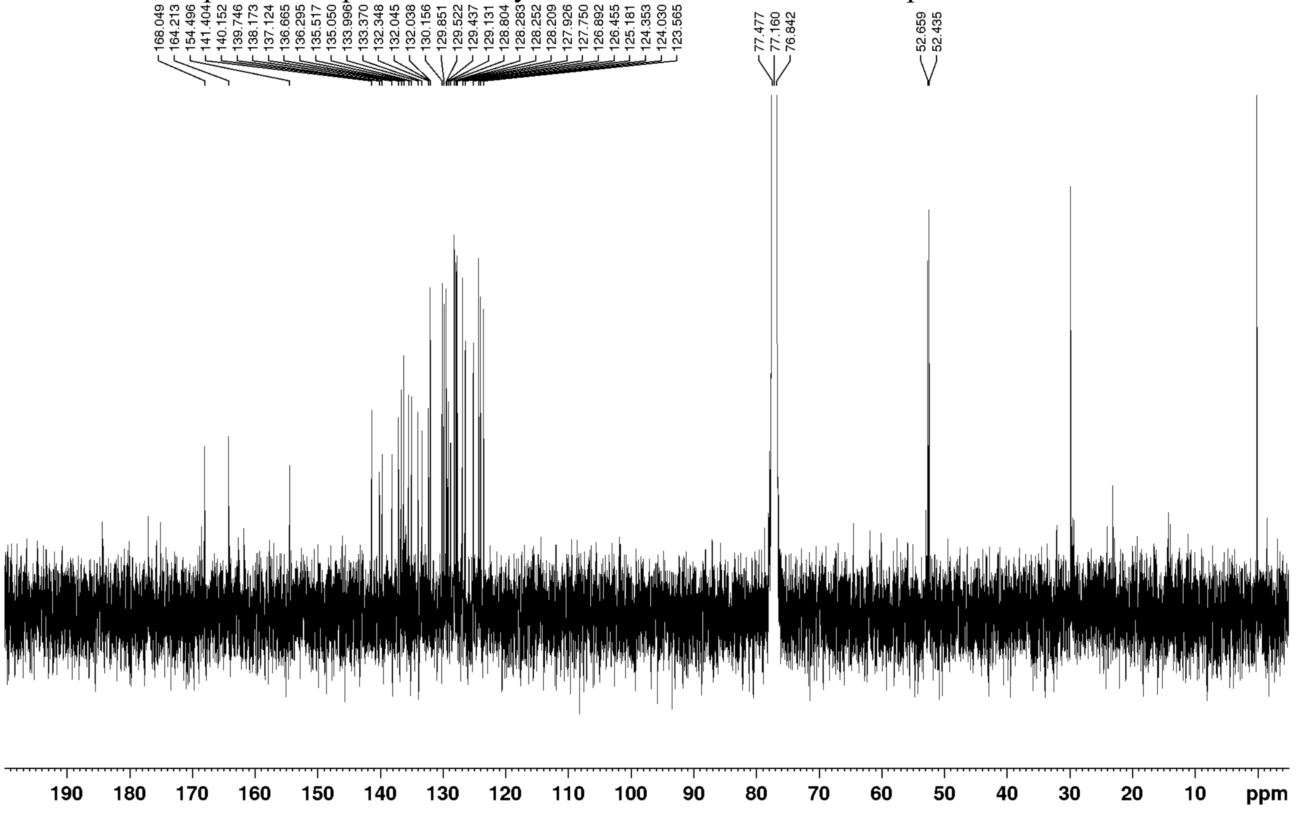

Tetramethyl 5,16-di-tert-butyl-2,13-dichlorodibenzo[3,4:5,6]cyclopenta[7,8]azuleno[2,1-b]benzo[7,8]azuleno[6,5,4-jk]fluorene-9,10,20,21-tetracarboxylate (8)

^{1}H NMR ($CDCl_3$, 400 MHz)

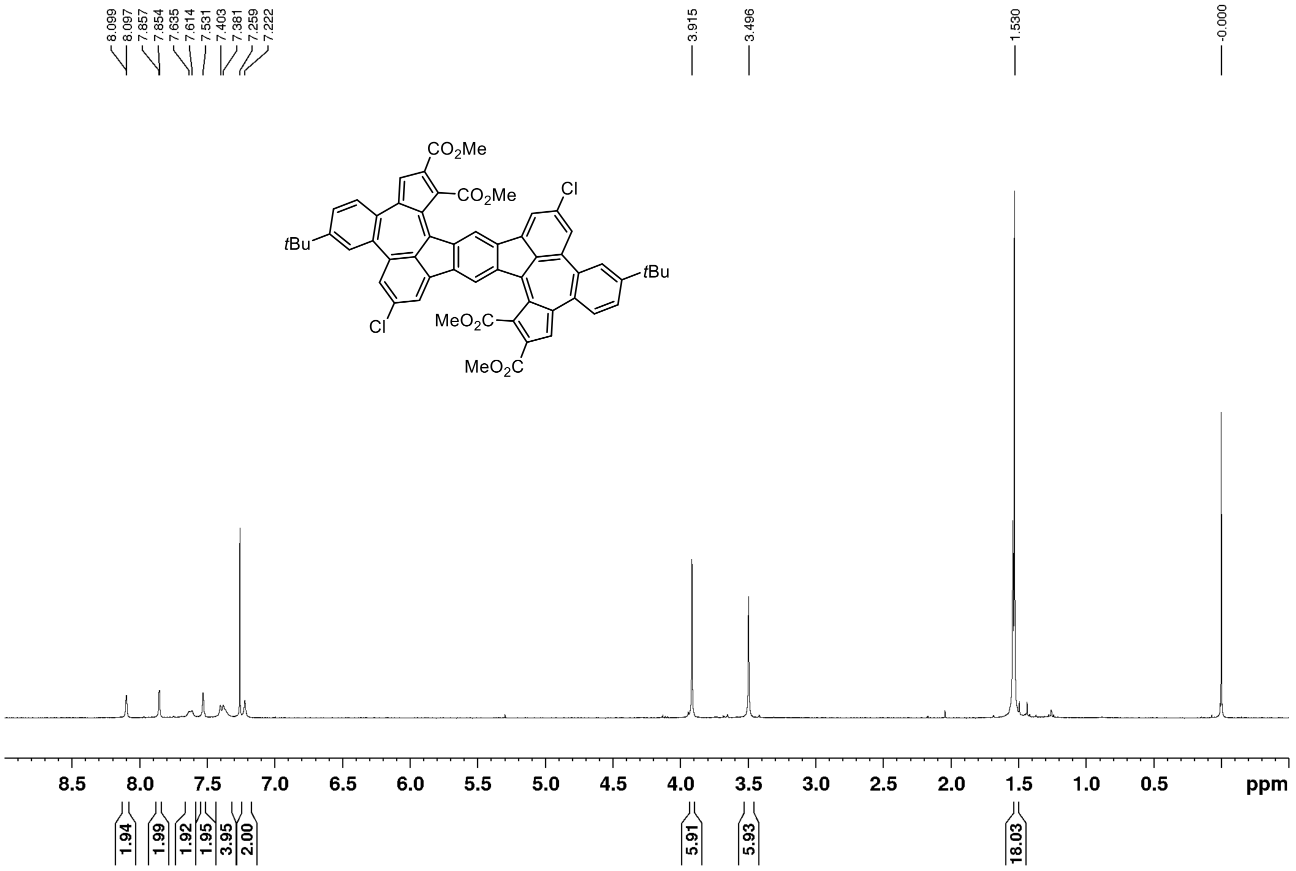

^{13}C NMR ($CDCl_3$, 100 MHz)

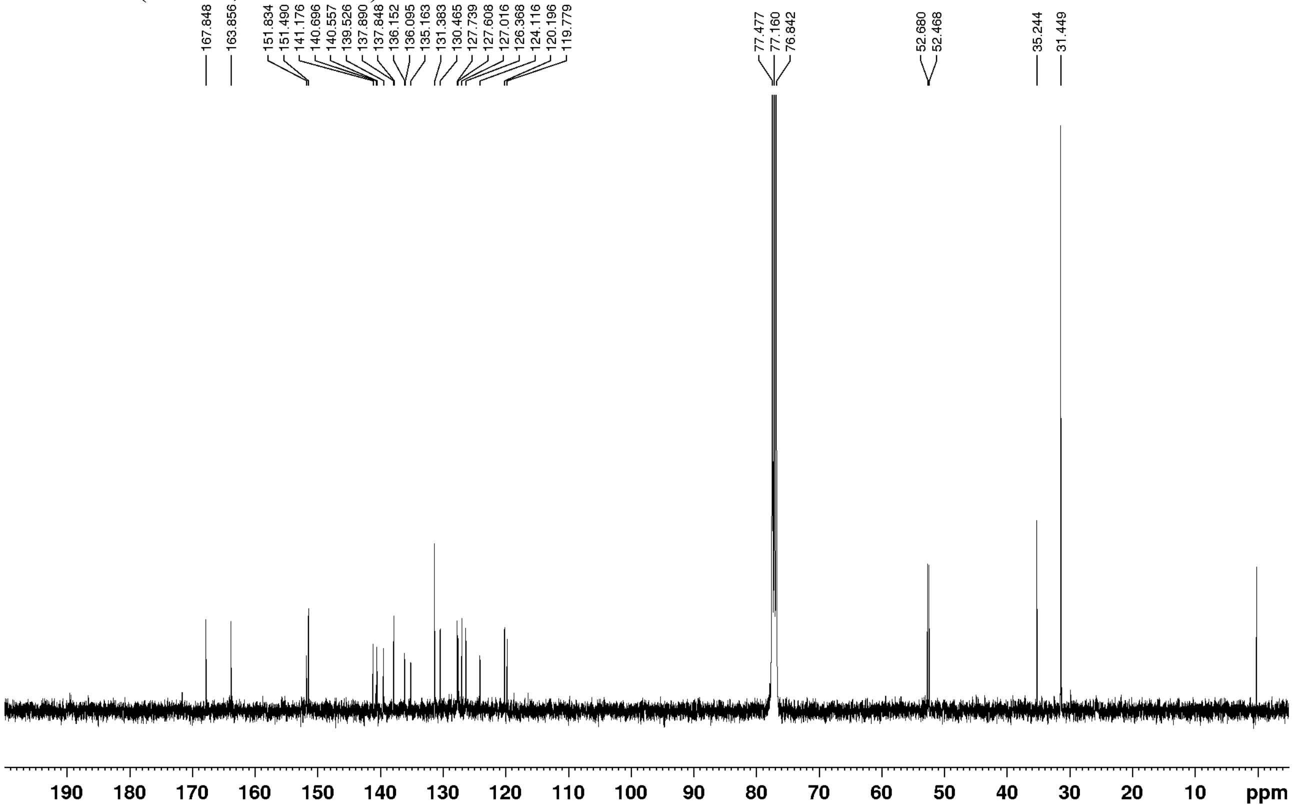

Tetramethyl 2,2'-dichloro-5,5',13,13'-tetramethoxy-[8,8'-bibenzo[7,8]azuleno[6,5,4-jk]fluorene]-9,9',10,10'-tetracarboxylate (9)

^{1}H NMR ($CDCl_3$, 400 MHz)

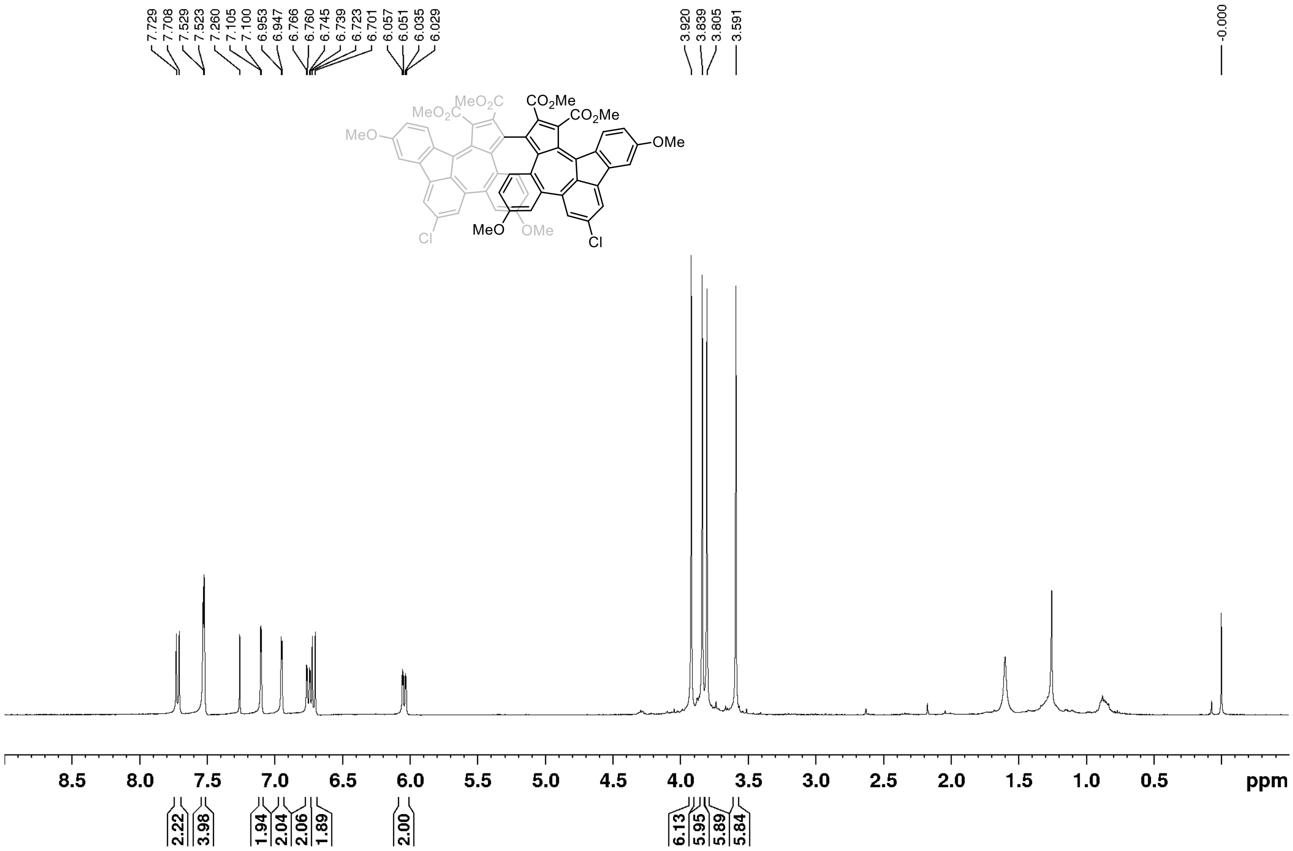

^{13}C NMR ($CDCl_3$, 100 MHz)

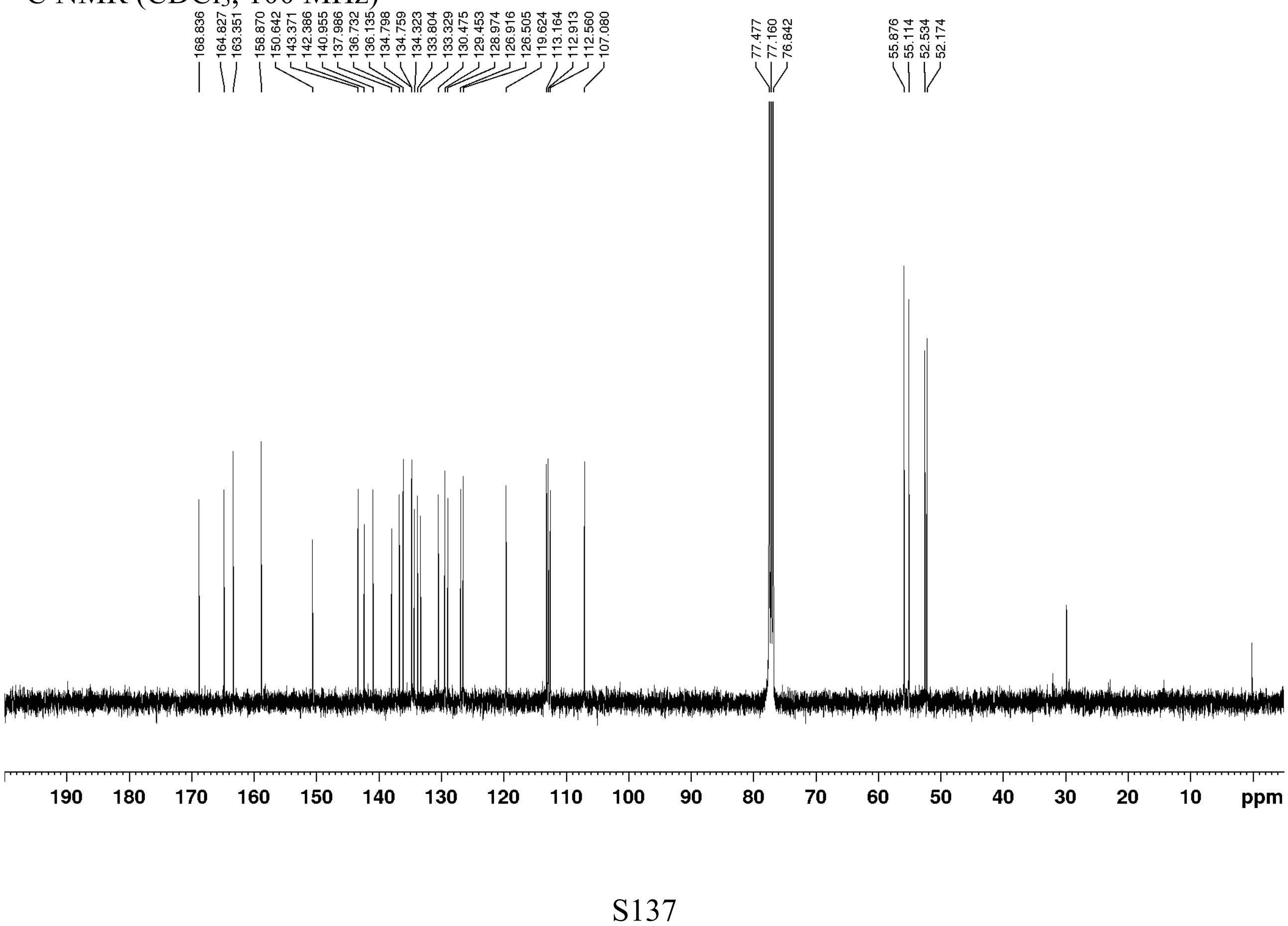

Mixture of 11, 12, and 13

^1H NMR ($CDCl_3$, 400 MHz)

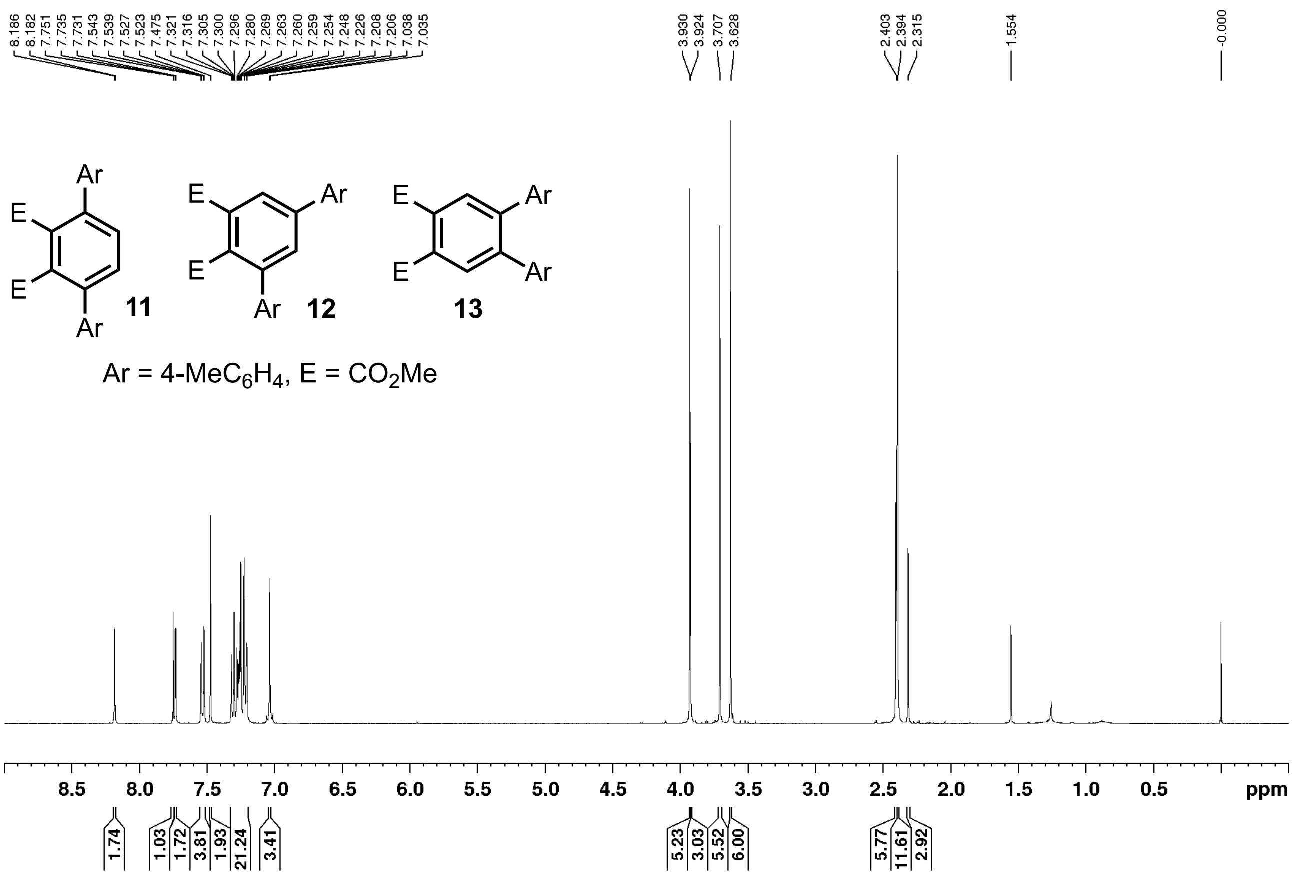

^{13}C NMR ($CDCl_3$, 100 MHz)

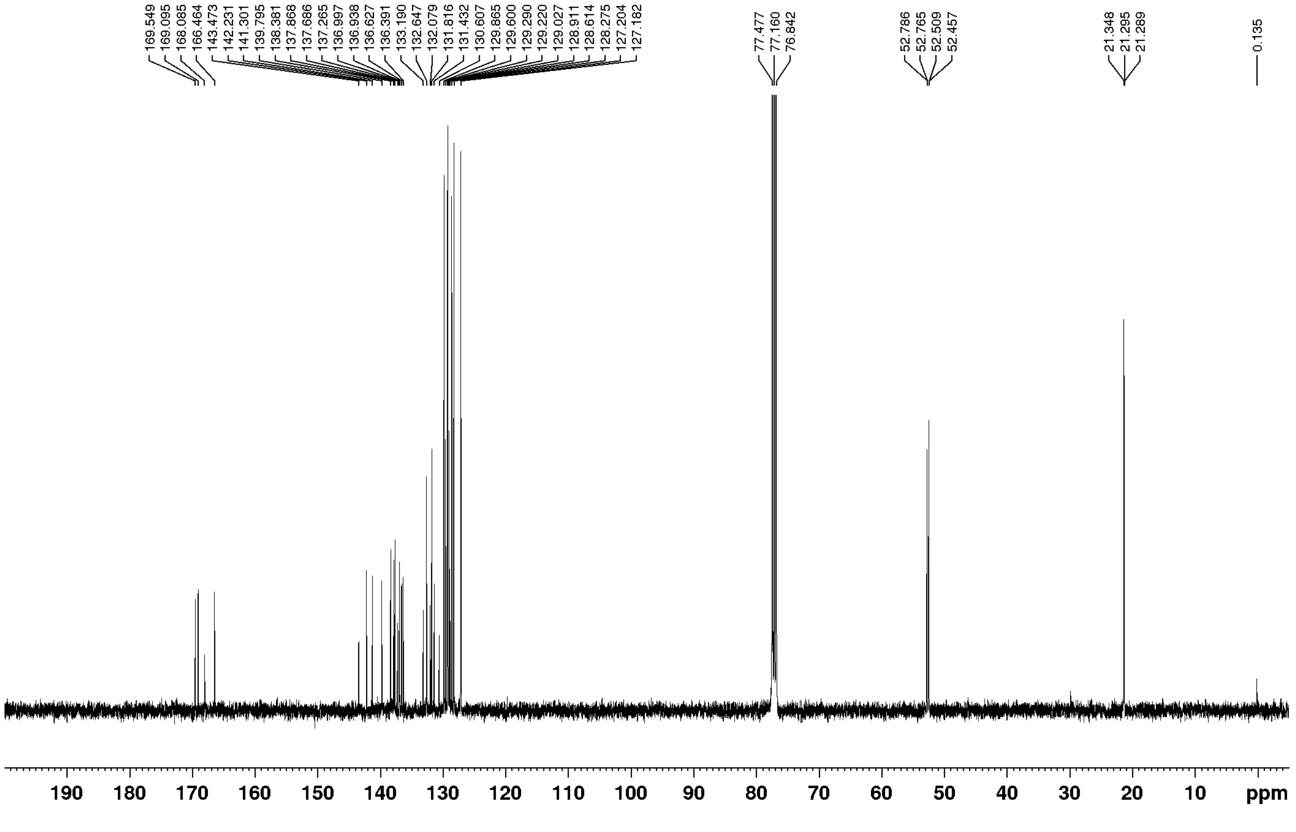

Mixture of 14, 15, and 16

^{1}H NMR ($CDCl_3$, 400 MHz)

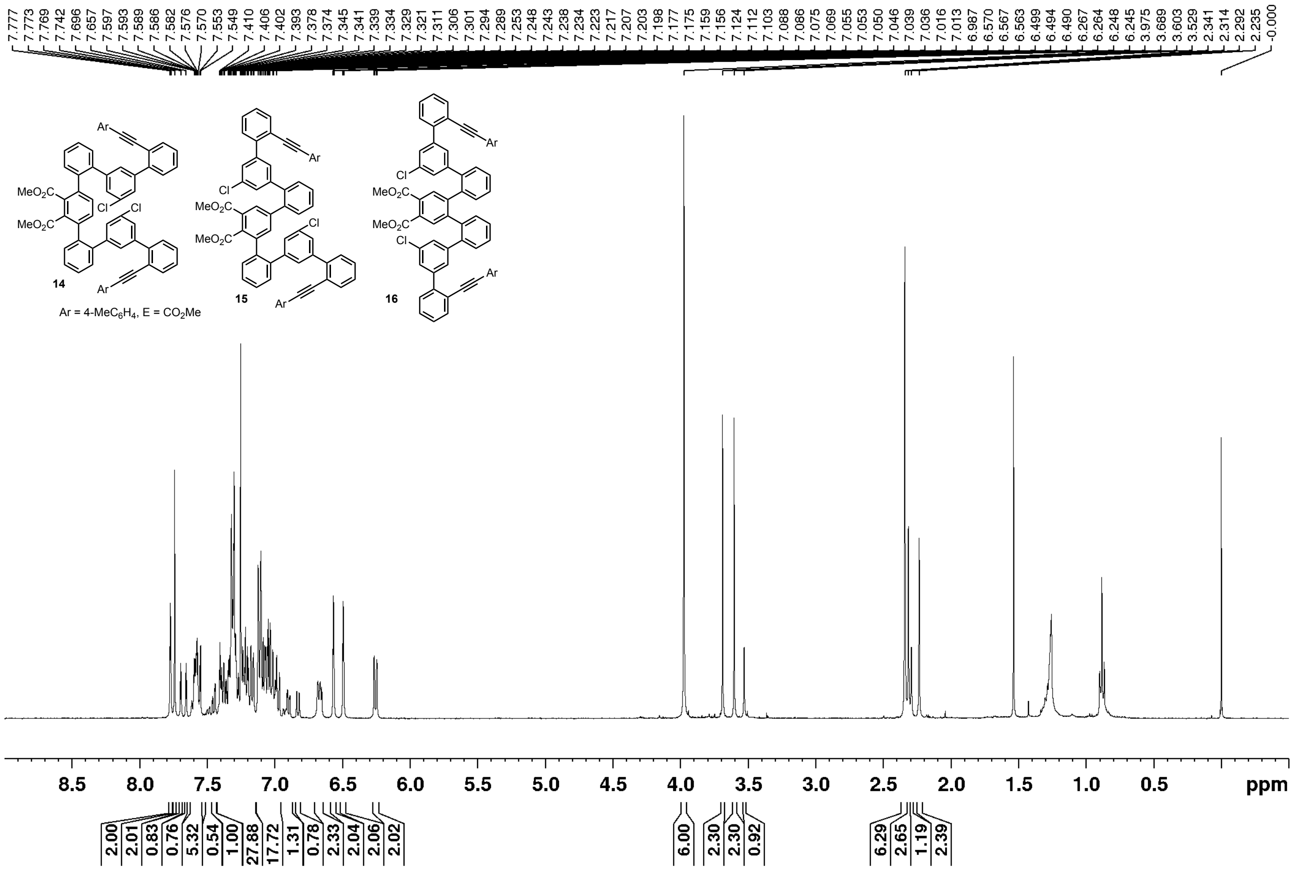

^{13}C NMR ($CDCl_3$, 100 MHz)

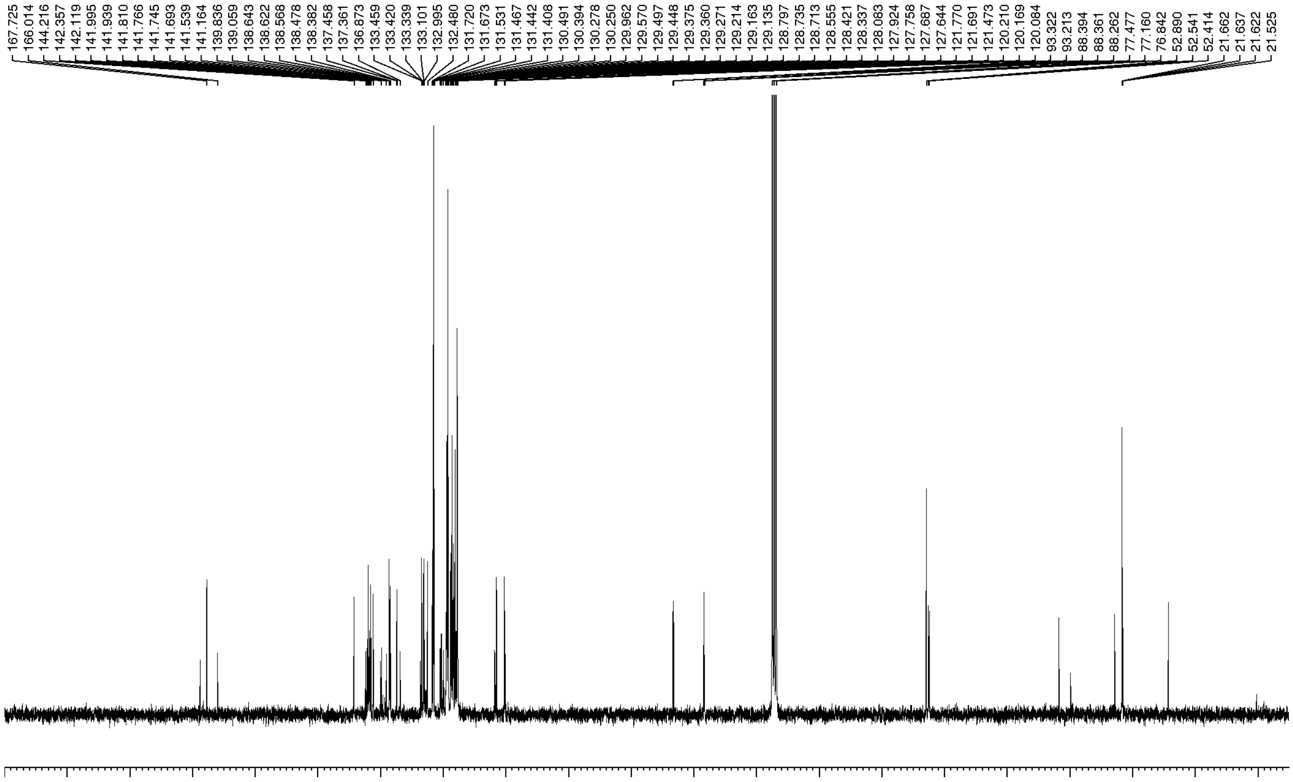

10. References

[1] A. L. Pinardi, G. Otero-Irurueta, I. Palacio, J. I. Martinez, C. Sanchez-Sanchez, M. Tello, C. Rogero, A. Cossaro, A. Preobrajenski, B. Gómez-Lor, A. Jancarik, I. G. Stará, I. Starý, M. F. Lopez, J. Méndez, J. A. Martin-Gago, *ACS Nano* **2013**, *7*, 3676–3684.

[2] E. Jin, Q. Yang, C.-W. Ju, Q. Chen, K. Landfester, M. Bonn, K. Müllen, X. Liu, A. Narita, *J. Am. Chem. Soc.* **2021**, *143*, 10403–10412.

[3] P. Ruffieux, S. Wang, B. Yang, C. Sánchez-Sánchez, J. Liu, T. Dienel, L. Talirz, P. Shinde, C. A. Pignedoli, D. Passerone, T. Dumslaff, X. Feng, K. Müllen, R. Fasel, *Nature* **2016**, *531*, 489–492.

[4] Y. Kawai, T. Oriki, Y. Sato, J. Nogami, Y. Kamiya, S. Suzuki, K. Tanaka, *Org. Lett.* **2024**, *26*, 7869–7874.

[5] P. S. Shinde, A. C. Shaikh, N. T. Patil, *Chem. Commun.* **2016**, *52*, 8152–8155.

[6] G. M. Sheldrick, *Acta Cryst.* **2015**, *A71*, 3–8.

[7] G. M. Sheldrick, *Acta Cryst.* **2015**, *C71*, 3–8.

[8] Gaussian 16, Revision C.02, M. J. Frisch, G. W. Trucks, H. B. Schlegel, G. E. Scuseria, M. A. Robb, J. R. Cheeseman, G. Scalmani, V. Barone, G. A. Petersson, H. Nakatsuji, X. Li, M. Caricato, A. V. Marenich, J. Bloino, B. G. Janesko, R. Gomperts, B. Mennucci, H. P. Hratchian, J. V. Ortiz, A. F. Izmaylov, J. L. Sonnenberg, D. Williams-Young, F. Ding, F. Lipparini, F. Egidi, J. Goings, B. Peng, A. Petrone, T. Henderson, D. Ranasinghe, V. G. Zakrzewski, J. Gao, N. Rega, G. Zheng, W. Liang, M. Hada, M. Ehara, K. Toyota, R. Fukuda, J. Hasegawa, M. Ishida, T. Nakajima, Y. Honda, O. Kitao, H. Nakai, T. Vreven, K. Throssell, J. A. Montgomery, Jr., J. E. Peralta, F. Ogliaro, M. J. Bearpark, J. J. Heyd, E. N. Brothers, K. N. Kudin, V. N. Staroverov, T. A. Keith, R. Kobayashi, J. Normand, K. Raghavachari, A. P. Rendell, J. C. Burant, S. S. Iyengar, J. Tomasi, M. Cossi, J. M. Millam, M. Klene, C. Adamo, R. Cammi, J. W. Ochterski, R. L. Martin, K. Morokuma, O. Farkas, J. B. Foresman, and D. J. Fox, Gaussian, Inc., Wallingford CT, **2016**.

[9] a) A. D. Beche, *Phys. Rev.* **1988**, *A38*, 3098–3100; b) A. D. Beche, *J. Chem. Phys.* **1993**, *98*, 1372–1377; c) A. D. Beche, *J. Chem. Phys.* **1993**, *98*, 5648–5652; d) C. Lee, W. Yang, R. G. Parr, *Phys. Rev.* **1988**, *B37*, 785–788.

[10] a) R. Krishnan, J. S. Binkley, R. Seeger, J. A. Pople, *J. Chem. Phys.* **1980**, *72*, 650–654; b) A. D. McLean, G. S. Chandler, *J. Chem. Phys.* **1980**, *72*, 5639–5648; c) M. J. Frisch, J. A. Pople, J. S. Binkley, *J. Chem. Phys.* **1984**, *80*, 3265–3269.

[11] a) P. von R. Schleyer, C. Maerker, A. Dransfeld, H. Jiao, N. J. R. van E. Hommes, *J. Am. Chem. Soc.* **1996**, *118*, 6317–6318; b) Z. Chen, C. S. Wannere, C. Corminboeuf, R. Puchta, P. von R. Schleyer, *Chem. Rev.* **2005**, *105*, 3842–3888.

[12] T. Lu, F. Chen, *J. Comput. Chem.* **2012**, *33*, 580–592.

[13] a) R. Herges, D. Geuenich, *J. Phys. Chem. A* **2001**, *105*, 3214–3220; b) D. Geuenich, K. Hess, F. Köhler, R. Herges, *Chem. Rev.* **2005**, *105*, 3758–3772.

[14] E. D. Glendening, J. K. Badenhoop, A. E. Reed, J. E. Carpenter, J. A. Bohmann, C. M. Morales, P. Karafiloglou, C. R. Landis, F. Weinhold, *NBO 7.0.*, Theoretical Chemistry Institute, University of Wisconsin, Madison, WI, 2018.

[15] Y. Zhao, D. Truhlar, *Theor. Chem. Acc.* **2008**, *120*, 215–241.

[16] a) J. Tomasi, M. Persico, *Chem. Rev.* **1994**, *94*, 2027–2094; b) S. Miertuš, E. Scrocco, J. Tomasi, *Chem. Phys.* **1981**, *55*, 117–129.

[17] a) K. Fukui, *Acc. Chem. Res.* **1981**, *14*, 363–368; b) K. Ishida, K. Morokuma, A. Ko-mornicki, *J. Chem. Phys.* **1977**, *66*, 2153–2156; c) C. Gonzalez, H. B. Schlegel, *J. Chem. Phys.* **1989**, *90*, 2154–2161; d) H. B. Schlegel, C. Gonzalez, *J. Phys. Chem.* **1990**, *94*, 5523–5527.

[18] T. Lu, F. Chen, *J. Comput. Chem.* **2012**, *33*, 580–592.

[19] T. Lu, *J. Chem. Phys.* **2024**, *161*, 082503.

[20] W. Humphrey, A. Dalke, K. Schulten, *J. Molec. Graphics*, **1996**, *14*, 33–38.

[21] Ò. Torres, M. Fernàndez, À. Díaz-Jiménez, A. Pla-Quintana, A. Roglans, M. Solà, *Organometallics* **2019**, *38*, 2853–2862.
